# Halide Bond Assisted Double Desymmetrization of *Meso*‐Dicarboxylic Acids with Symmetrical Olefins via Asymmetric Halogenation

**DOI:** 10.1002/anie.202520866

**Published:** 2026-01-02

**Authors:** Qingyu Zhang, Haihui Wang, Ying‐Lung Steve Tse, Ying‐Yeung Yeung

**Affiliations:** ^1^ Department of Chemistry and State Key Laboratory of Synthetic Chemistry The Chinese University of Hong Kong Shatin, NT Hong Kong China

**Keywords:** Asymmetric catalysis, Desymmetrization, Halide bonds, Halogenation, Homogeneous catalysis

## Abstract

The efficient synthesis of complex chiral molecules from achiral precursors is essential for sustainable pharmaceutical development. Among available strategies, the desymmetrization of *meso* compounds provides a powerful route to construct multiple stereocenters in a single step. Although *meso*‐dicarboxylic acids are promising feedstocks, their catalytic asymmetric desymmetrization remains a significant challenge. Herein we report a catalytic asymmetric halolactonization of *meso*‐dicarboxylic acids with electronically unbiased olefins, achieving simultaneous desymmetrization of both the *meso*‐dicarboxylic acid and haliranium intermediate. This method efficiently constructs chiral caged polycyclic lactones bearing six stereocenters in a single operation. Furthermore, the reaction is extended to exocyclic olefins, granting access to valuable terpenoid scaffolds. Its synthetic utility is highlighted by the enantioselective formal synthesis of (–)‐longifolene, establishing a versatile platform for accessing norbornane‐based natural products. Mechanistic studies reveal that the brominated amino‐urea catalyst operates as a biomimetic hydrogen‐bonding activator. Unexpectedly, the bromine substituent on the catalysts functions as an unconventional Brønsted base to stabilize a key intermediate via halide bond instead of a typical halogen bond. This work marks a substantial advancement in terpenoid synthesis, merging operational simplicity with the efficient generation of multiple stereocenters in a single transformation.

## Graphical abstract

This work demonstrates a double desymmetrization of *meso*‐dicarboxylic acids with symmetrical olefins, constructing complex chiral lactones with six stereocenters in one step. Unexpectedly, the halide bond was found to be crucial in achieving high stereoselectivity. This method enables the formal synthesis of (–)‐longifolene and provides a versatile platform for accessing valuable terpenoid and norbornane‐based natural products.

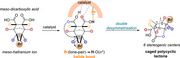

## Introduction

The efficient synthesis of complex chiral molecules from achiral precursors has gained significant attention in pharmaceutical research, driven by the increasing demand for sustainable development and modern manufacturing practices. Simplifying synthetic routes for these intricate chiral compounds offers a direct path to greener chemistry by reducing energy consumption and minimizing waste emissions.^[^ ^1^ ^]^ Among available strategies, the desymmetrization of *meso* compounds is particularly compelling, as it allows the efficient construction of molecules with multiple stereocenters in a single step.^[^ ^2^, ^3^, ^4^, ^5^, ^6^ ^]^ This approach dramatically shortens synthetic sequences, streamlining the production of valuable chiral compounds while adhering to sustainable chemistry principles.

**Scheme 1:**
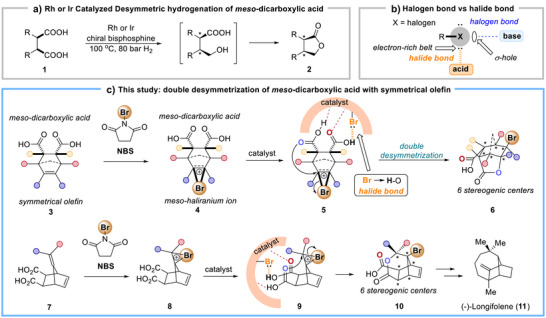
Desymmetrizing asymmetric reactions with *meso*‐dicarboxylic acids. a) A literature report on desymmetric hydrogenation of *meso*‐dicarboxylic acid. b) Halogen bond vs. halide bond. c) Our study on enantioselective halolactonization of *meso*‐dicarboxylic acid with symmetrical olefin via double desymmetrization.

*Meso*‐dicarboxylic acids are abundant, readily available, and cost‐effective feedstocks. They can be converted into high‐value intermediates for the synthesis of complex functional molecules through various strategies.^[^ ^7^, ^8^ ^]^ Although desymmetrization offers a promising route to generate useful chiral derivatives for different applications, catalytic desymmetrizing asymmetric transformations involving carboxylic acids remain a significant challenge. The nucleophilicity of carboxylates is inherently low due to resonance stabilization and the presence of an acidic proton, limiting their reactivity compared to other oxygen‐based nucleophiles. While some biological systems^[^ ^9^ ^]^ and artificial catalysts^[^ ^10^, ^11^, ^12^ ^]^ employ hydrogen bond networks to facilitate asymmetric reactions using carboxylate, there was no catalytic method for the desymmetrization of *meso*‐dicarboxylic acids until recently. Zhang, Wen, and coworkers reported a seminal work on the catalytic asymmetric hydrogenation of *meso*‐dicarboxylic acids **1** using chiral Rh/Ir complexes.^[^ ^13^ ^]^ In this transformation, one of the carboxylate groups is selectively reduced to an alcohol, which then undergoes intramolecular esterification with the remaining carboxylic acid to furnish enantioenriched lactones **2** (Scheme 1a).

The asymmetric catalytic halolactonization of olefinic acids has emerged as a highly valuable synthetic strategy for constructing lactones, privileged heterocyclic motifs in organic synthesis.^[^ ^14^, ^15^, ^16^, ^17^, ^18^, ^19^, ^20^, ^21^, ^22^, ^23^, ^24^, ^25^, ^26^, ^27^, ^28^, ^29^ ^]^ Among its variants, desymmetrizing asymmetric halolactonization has garnered particular attention, as it enables the simultaneous introduction of multiple stereocenters and versatile halogen functionalities in a single operation. In addition, the halogen handles embedded within these lactones provide convenient sites for further functionalization, enabling rapid access to structurally diverse molecules. Despite its potential, the development of desymmetrizing halolactonization remains underexplored. To date, relevant reported examples are scarce and predominantly focus on using substrates featuring electronically biased olefins.^[^ ^30^, ^31^, ^32^, ^33^, ^34^, ^35^, ^36^, ^37^, ^38^, ^39^, ^40^ ^]^ In these cases, the reaction proceeds via the formation of a chiral haliranium intermediate, with subsequent nucleophilic attack favoring the Markovnikov products. While this pathway simplifies stereochemical control, it also highlights the current constraints in substrate scope. In stark contrast to conventional halolactonization strategies, catalytic desymmetrization of the carboxylate nucleophiles remains exceptionally rare, despite offering a promising alternative to expand the methodology's applicability. This approach holds particular potential for symmetrical, electronically unbiased olefins, where typical asymmetric halogenation methods are not applicable due to the formation of *meso*‐haliranium intermediates. A seminal work was reported by Johnston and coworkers on the desymmetrizing iodolactonization of substrates bearing a carboxylic acid and a symmetrical olefin.^[^ ^41^ ^]^ However, the desymmetrizing halolactonization of *meso*‐diacids with electronically unbiased olefins, a transformation that could provide direct access to structurally complex, multifunctional chiral building blocks has not been reported. The desymmetrization of *meso*‐diacids is non‐trivial due to multiple competing hydrogen bond donor sites, complicating the design of an effective catalyst.

In organohalogen compounds (R–X), the electron density around the halogen atom (X) is anisotropically distributed (Scheme 1b). This creates two distinct regions: an electron‐deficient area (commonly known as σ‐hole) located along the bond axis opposite the R–X bond, and an electron‐rich belt (the non‐bonding electrons) encircling the halogen.^[^ ^42^ ^]^ The σ‐hole can form noncovalent interactions with Lewis bases via halogen bonding.^[^ ^43^ ^]^ Conversely, the electron‐rich belt can act as a Lewis base to interact with an acid,^[^ ^44^, ^45^, ^46^, ^47^ ^]^ and the resulting R–X···acid interaction has been termed as halide bonds.^[^ ^48^ ^]^ While halogen bonds have been applied to various Lewis acid‐type catalytic reactions,^[^ ^49^ ^]^ catalysis involving halide bonds remains underexplored.^[^ ^50^ ^]^

As an ongoing interest in asymmetric halogenation in our research team,^[^ ^51^, ^52^, ^53^, ^54^, ^55^, ^56^ ^]^ here we report a catalytic asymmetric halolactonization of electronically unbiased olefins in *meso*‐dicarboxylic acid derivative **3** with *N*‐bromosuccinimide (NBS), achieving simultaneous desymmetrization of both the *meso*‐dicarboxylic acid group and the *meso*‐haliranium ion intermediate **4** (Scheme 1c). This strategy efficiently constructs chiral caged polycyclic lactone **6** with six stereocenters in a single step. Mechanistic studies suggest a catalyst‐substrate complex **5** facilitates double desymmetrization through multiple non‐covalent interactions. Unexpectedly, the bromine substituent on the catalyst operates as an unconventional Brønsted base using its electron‐rich belt, engaging the substrate's acidic proton via halide bond, which is a critical feature for effective enantiodifferentiation. The methodology extends to exocyclic olefin **7**, yielding valuable bridged polycyclic building block **10**, and its utility is showcased in the enantioselective formal synthesis of (−)‐longifolene (**11**).

Bridged polycyclic terpenoids represent an important class of natural products, widely distributed in biologically active compounds and characterized by their intricate topological architectures.^[^ ^57^, ^58^, ^59^, ^60^ ^]^ The structural complexity of these molecules, exemplified by prominent targets such as longifolene, longiborneol, and culmorone featuring the bicyclo[2.2.1]heptane framework, has long captivated the synthetic chemistry community, presenting formidable challenges for chemical synthesis. While significant progress has been made in developing synthetic approaches to these architecturally complex molecules,^[^ ^61^, ^62^, ^63^, ^64^, ^65^, ^66^, ^67^, ^68^, ^69^ ^]^ the scarcity of catalytic asymmetric methods limits the scope of application. By enabling rapid access to privileged bicyclo[2.2.1]heptane frameworks with operational simplicity and stereochemical control, the newly developed catalytic protocol offers a powerful platform for synthesizing complex terpenoid natural products.

## Results and Discussion

**Scheme 2:**
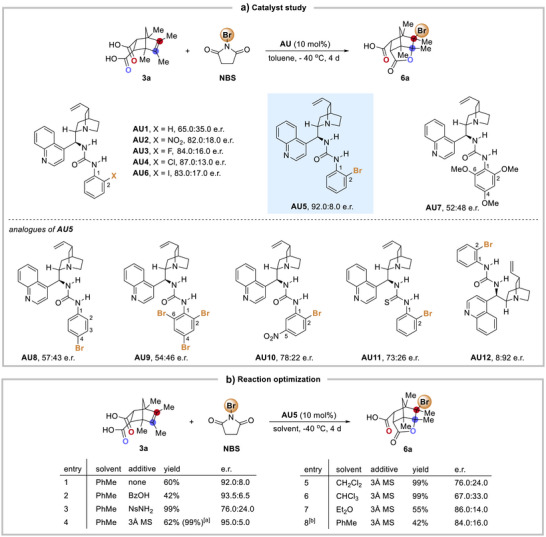
Desymmetrizing asymmetric bromolactonization of olefinic *meso*‐diacid **3a**. a) Catalyst study. b) Reaction optimization. Reactions were carried out with diacid **3a** (0.1 mmol), catalyst **AU** (0.01 mmol), additive, and NBS (0.105 mmol) in solvent (4 mL) at −40 °C for 4 d. The yields were measured using ^1^H NMR spectroscopy with CH_2_Br_2_ as the internal standard. ^[a]^The reaction time was 7 d. ^[b]^The reaction was performed at −78 °C.

Our study commenced with an investigation to identify a suitable catalyst that could perform this double desymmetrization process. We envisioned that effectively controlling the stereo‐conformation of the two carboxylic acids by chiral hydrogen bond catalysts could achieve our goal. Olefinic *meso*‐diacid **3a** was chosen as a starting material in the study (Scheme 2a). NBS was used as the brominating agent, and the reaction was conducted in toluene at −40 °C. After a survey of different chiral hydrogen bond catalysts (Figure S1), amino‐urea **AU1** was identified to be a suitable candidate to catalyze the bromolactonization of **3a**, giving chiral norbornane lactone **6a** in appreciable enantioselectivity (e.r. 65.0:35.0). Interestingly, further studies revealed that substituent at C(2) position of the aryl urea has a remarkable impact on the enantioselectivity; when catalysts **AU2** and **AU3** bearing nitro and fluorine were used, the e.r. was significantly improved. Other catalysts with different halogen substituents at C(2) were investigated. Although bromine is not the most electron‐withdrawing substituent among the catalysts that were studied, the C(2)‐Br catalyst **AU5** was found to be optimal, giving **6a** in e.r. 92.0:8.0. However, urea catalyst **AU7** with electron‐rich 2,4,6‐trimethoxyphenyl group gave negligible enantioselectivity. Analogues of catalyst **AU5** were then studied. When using catalyst **AU8** bearing C(4)‐Br substituent, the enantioselectivity dropped dramatically. When catalyst **AU9** bearing more Br atoms was used, the enantioselectivity was also far inferior to that of **AU5**. Catalyst **AU10** bearing a nitro group at the para‐position of the C(2)‐Br gave poorer e.r. compared with **AU5**, indicating that the C(2)‐Br in **AU5** might not serve as a typical halogen bond^[^ ^42^, ^43^ ^]^ donor. Changing the catalyst to thiourea **AU11** also caused a decrease in enantioselectivity. These results suggest that the urea's acidic N–H and the C(2)‐Br aryl substituent in **AU5** have unique roles in the desymmetrization of *meso*‐diacid **3a**. The antipode of **6a** was obtained in good enantioselectivity by using the pseudoenantiomeric quinidine catalyst **AU12**.

After identifying the suitable catalyst **AU5**, we examined the effect of additives on the reaction. Acid additives aiming at activating NBS by protonation of the carbonyl group of succinimide were examined. While BzOH has a slight improvement on the e.r., the addition of NsNH_2_ had a deteriorating effect (Scheme 2b, entries 2–3). Since the hydrogen bond network might be involved in the catalyst‐substrate complexation and too much moisture could interrupt the interaction and so molecular sieves (MS) were added. To our satisfaction, adding 3 Å MS gave a 62% yield and good e.r. (95:5) of product **6a** (Scheme 2b, entry 4). The *meso*‐diacid **3a** was completely consumed after extending the reaction time and an excellent yield (99%) of the desired product **6a** was obtained. Various solvents were examined and the performance with toluene was found to be superior (Scheme 2b, entries 4–7). In addition, −40 °C was found to be the optimal reaction temperature (Scheme 2b, entry 4 vs 8).

**Scheme 3:**
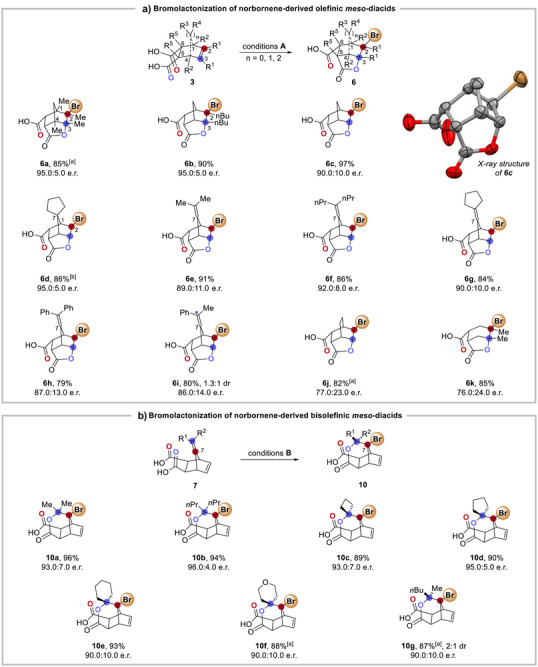
Substrate scope. a) Bromolactonization of norbornene‐derived olefinic *meso*‐diacids **3**. b) Bromolactonization of norbornene‐derived bisolefinic *meso*‐diacids **7**. Conditions **A**: Reactions were conducted using *meso*‐diacid **3** (0.1 mmol), catalyst **AU5** (0.01 mmol), 3 Å MS (15 mg), and NBS (0.105 mmol) in toluene at − 40 °C for 3 d. Conditions **B**: Reactions were conducted using *meso*‐diacid **7** (0.1 mmol), catalyst **AU5** (0.03 mmol), 3 Å MS (30 mg), and NBS (0.105 mmol) in toluene at −40 °C for 2 d. The yield and e.r. were determined on the ester of **6** and **10**, see Supporting Information for details. ^[a]^The reaction time was 7 d. ^[b]^The reaction was carried out in toluene/Et_2_O (9:1) for 9 d.

With the optimized conditions established, we explored the bromolactonization of various norbornene‐derived olefinic *meso*‐diacids **3** (Scheme 3a). The reaction proved broadly applicable, delivering high conversions and excellent stereoselectivity across diverse substrates. Substrates **3a** and **3b** bearing substituents at the bridgehead [C(1) and C(4)] and the cyclohexene ring [C(2) and C(3)] underwent smooth conversion to the corresponding norbornane lactones **6a** and **6b** in good yields with enantiomeric ratios (95:5 e.r.). The unsubstituted norbornene derivative **3c** also performed well under the optimal conditions, affording **6c** efficiently. While the C(7)‐substituted norbornene **3d** exhibited reduced reaction efficiency and an extended reaction time was required, the polycyclic product **6d** was still obtained in good yield and enantioselectivity. Further evaluation of substrates **3e–3i**, featuring exocyclic aliphatic and cyclic alkyl olefins at the C(7) position of bicyclo[2.2.1]heptane scaffold, demonstrated excellent functional group tolerance. These substrates underwent efficient desymmetrizing asymmetric halogenation at the endocyclic double bond, furnishing the corresponding lactones **6e–6i** in good yields (up to 91%) while maintaining high enantioselectivity. The reaction scope extended beyond norbornene frameworks, accommodating bicyclo[2.2.2]octene **3j** and cyclohexene **3k** substrates, furnishing **6j** and **6k** smoothly. The absolute configuration of **6c** was unambiguously determined by X‐ray crystallographic analysis,^[^ ^70^ ^]^ with the stereochemistry of other products assigned by analogy.

Next, we implemented the catalytic protocol for the desymmetrization of diolefinic *meso*‐diacids **7**, which was anticipated to favor the cyclization olefins at the C(7) position of norbornane (Scheme 3b). Gratefully, good yields and enantioselectivity of **10** were generally observed. Notably, the four‐membered ring substrate **7c**, characterized by significant ring strain successfully endured the reaction conditions to give **10c**. Among the substrates with cycloalkane substituents (**7c**‐**7e**), substrate **7d** bearing a cyclopentane, exhibited the least ring tension and yielded **10d** with the most favorable enantiomeric ratio (95:5). Furthermore, substrate **7f** containing a tetrahydropyran demonstrated compatibility with our catalytic protocol to give **10f** smoothly. The reaction with substrate **7g**, which possesses unsymmetrical substituents (methyl and *n*‐butyl) also yielded **10g** having six stereogenic centers with promising stereoselectivity.

**Scheme 4:**
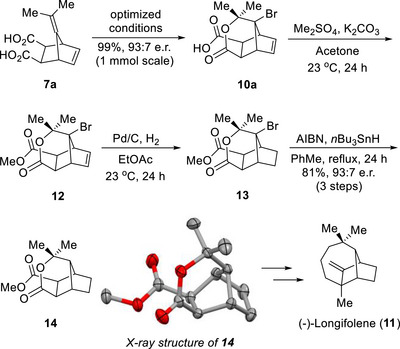
Formal synthesis of (–)‐Longifolene (**11**).

The synthetic utility of this catalytic protocol was exemplified through the formal synthesis of (–)‐longifolene (**11**) (Scheme 4). The preparation of compound **10a** from **7a** via our catalytic system demonstrated excellent scalability while maintaining both efficiency and enantioselectivity. Following the processes of methylation, hydrogenation, and free radical‐mediated debromination (**10a→12→13→14**), the polycyclic compound **14** was obtained as an advanced intermediate for the total synthesis of (–)‐longifolene (**11**),^[^ ^71^, ^72^, ^73^ ^]^ achieving a smooth yield of 81% with an enantiomeric ratio of 93:7 over three steps. The configuration of compound **14** was validated through X‐ray crystallographic analysis.^[^ ^70^ ^]^ This finding paves the way for the catalytic asymmetric total synthesis of structurally relevant compounds, facilitating the preparation of other topologically intricate natural products that feature the bicyclo[2.2.1]heptane framework, a significant category of bioactive compounds.^[^ ^60^, ^61^, ^62^, ^63^, ^64^, ^65^, ^66^, ^67^, ^68^ ^]^

The 2,4,6‐trimethoxy‐substituted catalyst **AU7** exhibited poor enantioselectivity, while urea **AU5** outperformed thiourea **AU11** (Scheme 2a). These observations suggest that the reaction may not follow a Lewis base mechanism and should proceed via dihydrogen bond activation. Kinetic analysis revealed that the reaction is 1.6 order with respect to the *meso*‐diacid substrate **3a** and near first‐order with respect to NBS and the urea catalyst **AU5** (Figure S2–S4).

**Figure 1:**
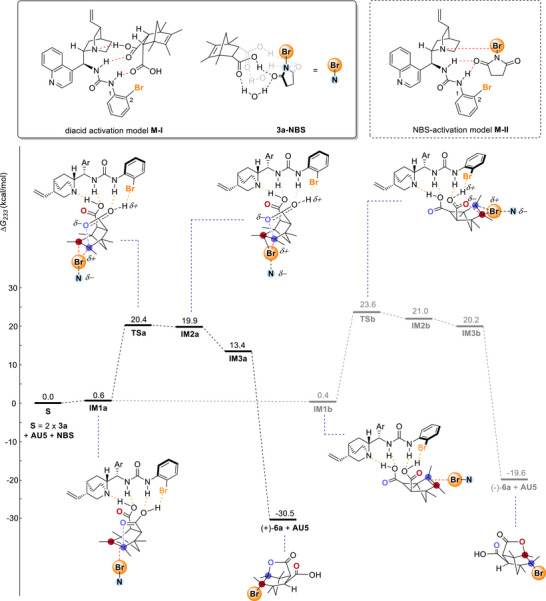
Calculated free energy profile for the reaction at 233 K by DFT at the calculation level ωB97X‐2 D3(BJ) SMD(Toluene)/def2‐TZVPP // M06‐2X‐D3(0) CPCM(Toluene) /def2‐SVP.

Density functional theory (DFT) at the calculation level ωB97X‐2 D3(BJ) SMD(toluene)/def2‐TZVPP // M06‐2X‐D3(0) CPCM(toluene) /def2‐SVP^[^ ^74^, ^75^, ^76^, ^77^, ^78^, ^79^, ^80^, ^81^, ^82^ ^]^ was carried out to gain deeper mechanistic insights into the reaction (Figure 1). Guided by prior studies in asymmetric halolactonization for the common activation modes,^[^ ^14^, ^15^, ^16^, ^17^, ^18^, ^19^, ^20^, ^21^, ^22^, ^23^, ^24^, ^25^, ^26^, ^27^, ^28^, ^29^ ^]^ two initial complexes were evaluated: 1) a urea catalyst bound to *meso*‐diacid (**M‐I**); 2) NBS activation by urea (**M‐II**). Computational results indicated that the formation of **M‐I** is exergonic by 4.5 kcal/mol relative to its starting materials, whereas the formation of **M‐II** is endergonic by 2.7 kcal/mol. These results suggest that **M‐I** is a more favorable species (Figure S6). These findings were further supported by NMR studies of the catalyst/*meso*‐diacid mixture, which revealed significant chemical shifts not only for the quinuclidine group but also for the urea N–H protons, supporting their role in substrate binding (Figure S5).

Subsequent calculations on the bromination of species **M‐I** by NBS indicated that direct NBS involvement resulted in species that are improbable in terms of the free energy (Figure S7). Given the near second‐order dependence on *meso*‐diacid (1.6 order experimentally), further calculations were performed using two diacid molecules. According to a literature report,^[^ ^83^ ^]^ we allowed one acid molecule to hydrogen‐bond with NBS to give **3a‐NBS** (Figure S6). After optimization, species **M‐I** was found to interact with **3a‐NBS** to form the halogen bond complex **IM1a**, with a free energy increase of 0.6 kcal/mol with respect to the starting status **S** (Figure 1). In **IM1a**, the catalyst **AU5** engaged the *meso*‐diacid **3a** via multiple noncovalent interactions. The bromolactonization of **IM1a** proceeded to give **IM2a** via **TSa** (Figure S11, S12) with a calculated barrier of 20.4 kcal/mol (relative to **S**) in the presence of two explicit water molecules (Figure S18). Subsequently, a conformational change via **IM3a** (Figure S13) followed by nucleophilic ring‐opening of the bromiranium species led to the cyclization product (+)‐**6a**. For the pathway leading to the minor enantiomer (‐)‐**6a**, we concluded that **IM1a** could undergo a conformational change to give **IM1b** (Figure S14‐S16). Upon halogenation at the olefin, **IM1b** was converted into **IM2b** via **TSb** with a free energy barrier of 23.6 kcal/mol (relative to **S**) with two explicit water molecules. Cyclization of **IM2b** via **IM3b** gave the minor enantiomer (‐)‐**6a**. Thus, **TSa** was both the rate and enantio‐determining step. In addition, the free energy of **TSa** was 3.2 kcal/mol lower than that of **TSb**, in agreement with the experimental enantiomeric ratio.

**Figure 2:**
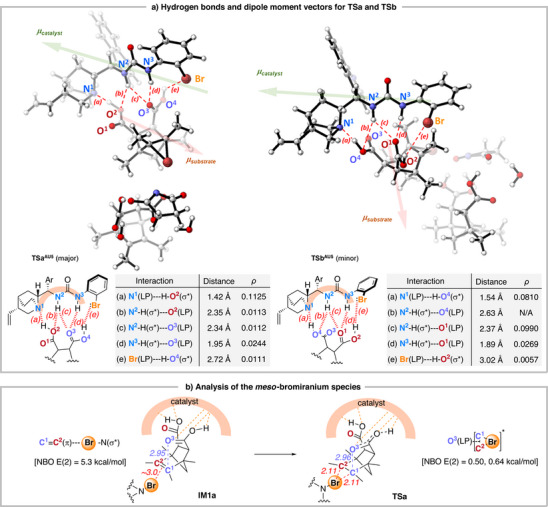
Structural and electronic structure analyses by calculation. a) Hydrogen bonds and dipole moment vectors for **TSa** and **TSb**. The green and red arrows show the directions of the dipole moment vectors (lengths not drawn to scale) of the catalyst and diacid substrate, respectively. b) Analysis of the *meso*‐bromiranium species.

Atom In Molecule (AIM)^[^ ^84^ ^]^ and Natural Bond Orbital (NBO)^[^ ^85^ ^]^ analysis were performed using Multiwfn 3.8 (dev)^[^ ^86^, ^87^ ^]^ and visualized with VMD 1.9.4^[^ ^88^ ^]^ to gain a better understanding of the enantiodetermining step that differentiates the major and minor transition states, **TSa** and **TSb** (Figure 2a). **TSa** was found to have considerably stronger hydrogen bond interactions (based on the electron density *ρ*) that contribute to its stabilization than **TSb**. These include: (*a*) quinuclidine N(LP)···H‐O Brønsted interaction; (*b‐d*) urea N–H···O═C hydrogen bonding. These interactions are orchestrated to control the *meso*‐diacid conformation, enabling effective enantiodifferentiation in the desymmetrization process. In **TSa**, the quinuclidine nitrogen forms a strong hydrogen bond with one of the carboxylic acid groups (O^2^ of **TSa** in Figure 2a), reflected by the short N^1^∙∙∙H‐O^2^ bond distance (1.42 Å) and the relatively large *ρ* value in the AIM analysis. On the other hand, the other carboxylic acid oxygen O^3^ is positioned near the olefin in substrate **3a**. This binding mode desymmetrizes the *meso*‐dicarboxylic acid. A dipole moment analysis^[^ ^90^, ^91^ ^]^ of **TSa** and **TSb** revealed that **TSa** exhibits a more favorable alignment, where the dipole vectors of the substrate **3a** (***µ***_s__ubstrate_, red arrow) and catalyst **AU5** (***μ***_catalyst_, green arrow) are roughly antiparallel, with their positive ends near each other's negative ends, enhancing stability through a more favorable dipole‐dipole interaction.

NBO analysis of **IM1a** indicated a notable C^1^═C^2^(π)→Br‐N(σ*) halogen bond, ultimately leading to the generation of the *meso*‐bromiranium species in **TSa** (Figure 2b).^[^ ^89^ ^]^ Subsequently, the proximity of the carboxylate oxygen O^3^ to the bromiranium three‐membered ring species, due to the interaction between a lone pair of O^3^ and a three‐center antibonding orbital of the bromiranium [denoted by O^3^(LP)→3c^*^(C^1^‐C^2^‐Br)], favors the formation of the O^3^–C^1^ bond in **TSa**. This results in the weakening of the C^1^‐Br bond and ring‐opening of the bromiranium intermediate. Thus, the *meso*‐bromiranium intermediate was desymmetrized to yield bromolactone **6a**.

Unexpectedly, the C(2)‐Br at the phenyl group in the catalyst **AU5** plays a crucial role in governing the enantioselectivity. Its influence extends beyond that of a simple electron‐withdrawing group, as replacing it with the more electron‐withdrawing nitro group (i.e., catalyst **AU2** in Scheme 2a) gave inferior asymmetric performance. In addition, the position of the Br in **AU5** is important because the performance of C(4)‐Br catalyst **AU8** dropped significantly, although they have similar electronic demand (Scheme 2a).

**Figure 3:**
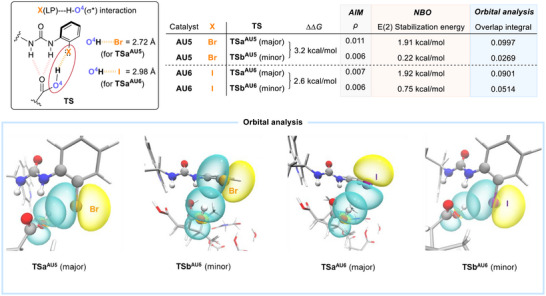
Analyses on **TSa** and **TSb** of the brominated catalyst **AU5** and iodinated catalyst **AU6**.

To explore further, calculations on **TSa** of the catalyst analogues **AU1** (non‐halogenated catalyst) and **AU5** (Br‐substituted catalyst) were carried out. A similar urea hydrogen‐bond system was observed for both **AU1** and **AU5** (Figure S10). However, in catalyst **AU5**, the distance between O^4^‐H of the diacid **3a** and the Br on the catalyst (2.72 Å) is shorter than the sum of their van der Waals radii (2.95 Å) (Figure 2a). AIM, NBO E(2), and NCI (Figure S8, S9) analyses on **TSa^AU5^** indicate that there is an unconventional halogen‐hydrogen interaction. Instead of the typical halogen‐bond involving σ‐hole Lewis acid‐type interaction,^[^ ^42^, ^43^ ^]^ the bromine's electron‐rich belt (the non‐bonding electrons) functions as a Brønsted base,^[^ ^44^, ^45^, ^46^, ^47^, ^48^, ^49^ ^]^ serving as a hydrogen‐bond acceptor to form a significant Br(LP)→H‐O^4^(σ*) interaction in **TSa^AU5^** of the brominated catalyst **AU5** (Figure 3). In comparison, such interaction was found to be much less significant in **TSb^AU5^**. This rare example of halogen‐centered basicity (halide bond)^[^ ^48^, ^50^ ^]^ enhances structural stability and conformational rigidity, dictating the enantiodifferentiation.

Iodine is a softer halogen compared to bromine, and its more polarizable electron cloud is expected to act as a better hydrogen bond acceptor. However, the iodinated catalyst **AU6** exhibits lower enantioselectivity than its brominated counterpart **AU5** (Scheme 2a). To elucidate this difference, further electronic structure analysis was performed to compare **AU5** and the iodine‐substituted catalyst **AU6**. In the calculated **TSa^AU6^** (major) of the iodinated catalyst **AU6**, a significant I(LP)→H‐O^4^(σ*) interaction was observed by NBO analysis (Figure 3). Nevertheless, such interaction remains significant in **TSb^AU6^** (minor). Orbital analysis showed a greater difference in the orbital overlap of the pre‐orthogonal NBOs between **TSa^AU5^** and **TSb^AU5^**, stemming from significantly weaker orbital overlap in **TSb^AU5^**. Conversely, there remains substantial orbital overlap in the minor **TSb^AU6^**, resulting in a comparatively smaller difference in the overlap integral from that of **TSa^AU6^**. This distinction likely arises from iodine's larger lone‐pair orbital volume [35.8 Å^3^ (iodine) vs. 28.9 Å^3^ (bromine) enclosed by the isosurfaces at ± 0.02 a.u. of the orbital], a result of the softer iodine that enhances its availability for interactions in the **TSb^AU6^** system.

Overall, the relatively smaller differences observed in the ρ value at the critical bond points from AIM, E(2) stabilization energy, and orbital integrals between **TSa^AU6^/TSb^AU6^** (compared with **TSa^AU5^/TSb^AU5^**) all align with the reduced ΔΔ*G* value (2.6 kcal/mol) for the iodinated catalyst **AU6** (compared with the ΔΔ*G* = 3.2 kcal/mol with **AU5**). The diminished ΔΔ*G* of **TS** with **AU6** likely explains its lower enantioselectivity in the bromolactonization of **3a** (Scheme 2a). These results underscore the critical influence of the halogen substituent in the catalyst on the enantioselectivity.

## Conclusion

In summary, we have developed a highly efficient and enantioselective desymmetrizing halocyclization of olefinic diacids using a cinchonidine‐derived urea catalyst. Mechanistic studies support that multiple non‐covalent interactions enable a dual desymmetrization of both the *meso*‐dicarboxylic acid and the *meso*‐bromiranium intermediate in a single step. In addition, we have realized the first instance where the halogen's electron‐belt serves as a tunable handle for stereocontrol. This strategy provides access to intricate bridged polycyclic architectures with up to six stereocenters and has been demonstrated in the formal synthesis of (–)‐longifolene.

## Conflict of Interests

The authors declare no conflict of interest.

## Supporting information



Supporting Information

Supporting Information

## Data Availability

The data that support the findings of this study are available in the Supporting Information of this article.

## References

[1] K. R. Campos , P. J. Coleman , J. C. Alvarez , S. D. Dreher , R. M. Garbaccio , N. K. Terrett , R. D. Tillyer , M. D. Truppo , E. R. Parmee , Science 2019, 363, eaat0805, 10.1126/science.aat0805. 30655413

[2] U. Piarulli , P. Daubos , C. Claverie , M. Roux , C. Gennari , Angew. Chem. Int. Ed. 2003, 42, 234–236, 10.1002/anie.200390088. 12532360

[3] J. Merad , M. Candy , J.‐M. Pons , C. Bressy , Synthesis 2017, 49, 1938–1954.

[4] R. Jacques , R. D. C. Pullin , S. P. Fletcher , Nat. Commun. 2019, 10, 21, 10.1038/s41467-018-07871-x. 30604753 PMC6318275

[5] C. Nájera , F. Foubelo , J. M. Sansano , M. Yus , Tetrahedron 2022, 106‐107, 132629, 10.1016/j.tet.2022.132629.

[6] C.‐J. Yang , L. Liu , Q.‐S. Gu , X.‐Y. Liu , CCS Chem 2024, 6, 1612–1627, 10.31635/ccschem.024.202403839.

[7] J. J. R. Kamal in Rodd's Chemistry of Carbon Compounds, 2nd ed. (Ed.: M. Sainsbury ), Elsevier, Amsterdam 1993, vol. ID, Ch. p. 17.

[8] N. Majumdar , ACS Catal. 2022, 12, 8291–8324, 10.1021/acscatal.2c02410.

[9] W. Huang , J. Jia , K. J. Gibson , W. S. Taylor , A. R. Rendina , G. Schneider , Y. Lindqvist , Biochemistry 1995, 34, 10985–10995, 10.1021/bi00035a004. 7669756

[10] M. R. Monaco , B. Poladura , M. Diaz de Los Bernardos , M. Leutzsch , R. Goddard , B. List , Angew. Chem. Int. Ed. 2014, 53, 7063–7067, 10.1002/anie.201400169. 24888674

[11] J. Zhang , S.‐X. Lin , D.‐J. Cheng , X.‐Y. Liu , B. Tan , J. Am. Chem. Soc. 2015, 137, 14039–14042, 10.1021/jacs.5b09117. 26488384

[12] M. R. Monaco , D. Fazzi , N. Tsuji , M. Leutzsch , S. Liao , W. Thiel , B. List , J. Am. Chem. Soc. 2016, 138, 14740–14749, 10.1021/jacs.6b09179. 27779872

[13] L. Yang , T. Yang , Y. Qian , X. Zhang , J. Am. Chem. Soc. 2024, 146, 15908–15916, 10.1021/jacs.4c02538. 38809425

[14] K. D. Ashtekar , A. Jaganathan , B. Borhan , D. C. Whitehead , Org. React. 2004, 105, 1–100.

[15] A. Castellanos , S. P. Fletcher , Chem. ‐ Eur. J. 2011, 17, 5766–5776, 10.1002/chem.201100105. 21509840

[16] C. K. Tan , L. Zhou , Y.‐Y. Yeung , Synlett 2011, 1335–1339.

[17] S. E. Denmark , W. E. Kuester , M. T. Burk , Angew. Chem. Int. Ed. 2012, 51, 10938–10953, 10.1002/anie.201204347. PMC3529098 23011853

[18] U. Hennecke , Chem. Asian J. 2012, 7, 456–465, 10.1002/asia.201100856. 22315237

[19] S. R. Chemler , M. T. Bovino , ACS Catal. 2013, 3, 1076–1091, 10.1021/cs400138b. 23828735 PMC3697159

[20] K. Murai , H. Fujioka , Heterocycles 2013, 87, 763–805.

[21] C. K. Tan , Y.‐Y. Yeung , Chem. Commun. 2013, 49, 7985, 10.1039/c3cc43950j. 23903206

[22] Y. A. Cheng , W. Z. Yu , Y.‐Y. Yeung , Org. Biomol. Chem. 2014, 12, 2333–2343, 10.1039/C3OB42335B. 24595745

[23] C. K. Tan , W. Z. Yu , Y.‐Y. Yeung , Chirality 2014, 26, 328–343, 10.1002/chir.22272. 24339201

[24] S. Zheng , C. M. Schienebeck , W. Zhang , H.‐Y. Wang , W. Tang , Asian J. Org. Chem. 2014, 3, 366–376, 10.1002/ajoc.201400030.

[25] J. R. Wolstenhulme , V. Gouverneur , Acc. Chem. Res. 2014, 47, 3560–3570, 10.1021/ar500282z. 25379791

[26] A. Sakakura , K. Ishihara , Chem. Rec. 2015, 15, 728–742, 10.1002/tcr.201500005. 26147781

[27] M. H. Gieuw , Z. Ke , Y.‐Y. Yeung , Chem. Rec. 2017, 17, 287–311, 10.1002/tcr.201600088. 27701807

[28] Y. Cai , X. Liu , P. Zhou , X. Feng , J. Org. Chem. 2019, 84, 1–13, 10.1021/acs.joc.8b01951. 30339377

[29] S. Liu , B.‐Q. Zhang , W.‐Y. Xiao , Y. L. Li , J. Deng , Adv. Synth. Catal. 2022, 364, 3974–4005, 10.1002/adsc.202200611.

[30] D. H. Paull , C. Fang , J. R. Donald , A. D. Pansick , S. F. Martin , J. Am. Chem. Soc. 2012, 134, 11128–11131, 10.1021/ja305117m. 22726214 PMC3397382

[31] K. Ikeuchi , S. Ido , S. Yoshimura , T. Asakawa , M. Inai , Y. Hamashima , T. Kan , Org. Lett. 2012, 14, 6016–6019, 10.1021/ol302908a. 23148461

[32] M. Wilking , C. Mück‐Lichtenfeld , C. G. Daniliuc , U. Hennecke , J. Am. Chem. Soc. 2013, 135, 8133–8136, 10.1021/ja402910d. 23679927

[33] D. W. Tay , G. Y. C. Leung , Y.‐Y. Yeung , Angew. Chem. Int. Ed. 2014, 53, 5161–5164, 10.1002/anie.201310136. 24706539

[34] K. Murai , J. Nakajima , A. Nakamura , N. Hyogo , H. Fujioka , Chem. Asian J. 2014, 9, 3511–3517, 10.1002/asia.201402865. 25256170

[35] Z. Ke , C. K. Tan , F. Chen , Y.‐Y. Yeung , J. Am. Chem. Soc. 2014, 136, 5627–5630, 10.1021/ja5029155. 24697791

[36] Y. Nagao , T. Hisanaga , H. Egami , Y. Kawato , Y. Hamashima , Chem. ‐ Eur. J. 2017, 23, 16758–16762, 10.1002/chem.201704847. 29044749

[37] T. Arai , K. Horigane , O. Watanabe , J. Kakino , N. Sugiyama , H. Makino , Y. Kamei , S. Yabe , M. Yamanaka , iScience 2019, 12, 280–292, 10.1016/j.isci.2019.01.029. 30731356 PMC6365408

[38] M. Hiraki , K. Okuno , R. Nishiyori , A. A. Noser , S. Shirakawa , Chem. Commun. 2021, 57, 10907–10910, 10.1039/D1CC03874E. 34590630

[39] C. H. Müller , C. Rösner , U. Hennecke , Chem. Asian J. 2014, 9, 2162–2169. 24840391 10.1002/asia.201402229

[40] U. Hennecke , C. H. Müller , R. Fröhlich , Org. Lett. 2011, 13, 860–863, 10.1021/ol1028805. 21302896

[41] M. T. Knowe , M. W. Danneman , S. Sun , M. Pink , J. N. Johnston , J. Am. Chem. Soc. 2018, 140, 1998–2001, 10.1021/jacs.7b12185. 29400455 PMC5814305

[42] G. Cavallo , P. Metrangolo , R. Milani , T. Pilati , A. Priimagi , G. Resnati , G. Terraneo , Chem. Rev. 2016, 116, 2478–2601, 10.1021/acs.chemrev.5b00484. 26812185 PMC4768247

[43] Halogen Bonding in Solution (Ed: S. M. Huber ), Wiley‐VCH, Weinheim 2021, 10.1002/9783527825738.

[44] G. K. S. Prakash , F. Wang , M. Rahm , J. Shen , C. Ni , R. Haiges , G. A. Olah , Angew. Chem. Int. Ed. 2011, 50, 11761–11764, 10.1002/anie.201105288. PMC3439535 21984045

[45] C. Bartolomé , P. Espinet , J. M. Martín‐Alvarez , Chem. Commun. 2007, 4384, 10.1039/b710304b. 17957294

[46] M. Wang , N. Garrison , P. M. Nguyen , Y.‐L. S. Tse , J. A. Golen , D. R. Manke , J. Org. Chem. 2024, 89, 9681–9685, 10.1021/acs.joc.4c00873. 38965938

[47] S. J. Grabowski , Molecules 2021, 26, 5175, 10.3390/molecules26175175. 34500610 PMC8434224

[48] S. J. Grabowski , J. Phys. Chem. A 2011, 115, 12340–12347, 10.1021/jp205019s. 21970363

[49] R. L. Sutar , S. M. Huber , ACS Catal. 2019, 9, 9622–9639, 10.1021/acscatal.9b02894.

[50] C. E. Jakobsche , G. Peris , S. J. Miller , Angew. Chem. Int. Ed. 2008, 47, 6707–6711, 10.1002/anie.200802223. PMC3479438 18646230

[51] J. Huang , H. Yang , X. Chen , R. Liang , F.‐Y. Kwong , Z. Huang , M. W. Wong , Y.‐Y. Yeung , Chem 2025, 11, 102439, 10.1016/j.chempr.2025.102439.

[52] R. Chen , Y. Liu , H. Wang , Y.‐L. S. Tse , Y.‐Y. Yeung , Chem Catal 2025, 5, 101512.

[53] T. Zheng , R. Chen , J. Huang , T. P. Gonçalves , K.‐W. Huang , Y.‐Y. Yeung , Chem 2023, 9, 1255–1269, 10.1016/j.chempr.2023.01.016.

[54] Y.‐C. Chan , X. Wang , Y.‐P. Lam , J. Wong , Y.‐L. S. Tse , Y.‐Y. Yeung , J. Am. Chem. Soc. 2021, 143, 12745–12754, 10.1021/jacs.1c05680. 34350758

[55] T. Zheng , X. Wang , W.‐H. Ng , Y.‐L. S. Tse , Y.‐Y. Yeung , Nat. Catal. 2020, 3, 993–1001, 10.1038/s41929-020-00530-9.

[56] X. Xiong , T. Zheng , X. Wang , Y.‐L. S. Tse , Y.‐Y. Yeung , Chem 2020, 6, 919–932, 10.1016/j.chempr.2020.01.009.

[57] L. Yet , Chem. Rev. 2000, 100, 2963–3008, 10.1021/cr990407q. 11749312

[58] K. C. Nicolaou , T. Montagnon , Molecules that Changed the World Wiley‐VCH, Weinheim, 2008.

[59] Y. Yuan , Y. Lei , M. Xu , B. Zhao , S. Xu , Mar. Drugs 2025, 23, 96, 10.3390/md23030096. 40137282 PMC11943499

[60] Y. Zang , R. Sun , R. Feng , H. Zhu , X. Li , Chin. J. Chem. 2025, 43, 443–469, 10.1002/cjoc.202400697.

[61] S. C. Welch , R. L. Walters , Synth. Commun. 1973, 3, 419–423, 10.1080/00397917308065935.

[62] S. C. Welch , R. L. Walters , J. Org. Chem. 1974, 39, 2665–2673, 10.1021/jo00932a001.

[63] E. J. Corey , W. J. Howe , H. W. Orf , D. A. Pensak , G. Petersson , J. Am. Chem. Soc. 1975, 97, 6116–6124, 10.1021/ja00854a026.

[64] J. Hanson , R. Nyfeler , J. Chem. Soc. Perkin Trans. 1 1976, 2471, 10.1039/p19760002471. 1034641

[65] M. Ihara , K. Makita , Y. Fujiwara , Y. Tokunaga , K. Fukumoto , J. Org. Chem. 1996, 61, 6416–6421, 10.1021/jo960698s. 11667485

[66] K. Takasu , S. Mizutani , M. Noguchi , K. Makita , M. Ihara , J. Org. Chem. 2000, 65, 4112–4119, 10.1021/jo000185s. 10866628

[67] C. J. Marth , G. M. Gallego , J. C. Lee , T. P. Lebold , S. Kulyk , K. G. M. Kou , J. Qin , R. Lilien , R. Sarpong , Nature 2015, 528, 493–498, 10.1038/nature16440. 26675722 PMC4688071

[68] R. F. Lusi , G. Sennari , R. Sarpong , J. Am. Chem. Soc. 2022, 144, 17277–17294, 10.1021/jacs.2c08136. 36098550 PMC9721240

[69] R. F. Lusi , G. Sennari , R. Sarpong , Nat. Chem. 2022, 14, 450–456, 10.1038/s41557-021-00870-4. 35165424 PMC9117171

[70] Deposition numbers 2443944 (for **3a**), 2443943 (for **3c**), 2416060 (for **6c**), and 2411170 (for **14**). These data are provided free of charge by the joint Cambridge Crystallographic Data Centre and Fachinformationszentrum Karlsruhe Access Structures service.

[71] E. J. Corey , M. Ohno , P. A. Vatakencherry , R. B. Mitra , J. Am. Chem. Soc. 1961, 83, 1251–1253, 10.1021/ja01466a056.

[72] E. J. Corey , M. Ohno , R. B. Mitra , P. A. Vatakencherry , J. Am. Chem. Soc. 1964, 86, 478–485, 10.1021/ja01057a039.

[73] R. A. Volkmann , G. C. Andrews , W. S. Johnson , J. Am. Chem. Soc. 1975, 97, 4777–4779, 10.1021/ja00849a062.

[74] P. J. Stephens , F. J. Devlin , C. F. Chabalowski , M. J. Frisch , J. Phys. Chem. 1994, 98, 11623–11627, 10.1021/j100096a001.

[75] F. Weigend , R. Ahlrichs , Phys. Chem. Chem. Phys. 2005, 7, 3297, 10.1039/b508541a. 16240044

[76] S. Grimme , J. Antony , S. Ehrlich , H. Krieg , J. Chem. Phys. 2010, 132, 154104, 10.1063/1.3382344. 20423165

[77] S. Grimme , S. Ehrlich , L. Goerigk , J. Comput. Chem. 2011, 32, 1456–1465, 10.1002/jcc.21759. 21370243

[78] J.‐D. Chai , M. Head‐Gordon , J. Chem. Phys. 2009, 131, 174105, 10.1063/1.3244209. 19894996

[79] F. Weigend , F. Furche , R. Ahlrichs , J. Chem. Phys. 2003, 119, 12753–12762, 10.1063/1.1627293.

[80] S. Grimme , S. Ehrlich , L. Goerigk , J. Comput. Chem. 2011, 32, 1456–1465. 21370243 10.1002/jcc.21759

[81] H. X. Hou , D. G. Zhou , R. Li , Comput. Theor. Chem. 2022, 1208, 113545.

[82] A. V. Marenich , C. J. Cramer , D. G. Truhlar , J. Phys. Chem. B 2009, 113, 6378–6396, 10.1021/jp810292n. 19366259

[83] H. X. Hou , D. G. Zhou , R. Li , Comput. Theor. Chem. 2022, 1208, 113545.

[84] R. F. W. Bader , Atoms in Molecules: A Quantum Theory, Clarendon Press, Oxford 1990, 10.1093/oso/9780198551683.001.0001.

[85] E. D. Glendening , C. R. Landis , F. Weinhold , WIREs Comput. Mol. Sci. 2012, 2, 1–42, 10.1002/wcms.51.

[86] T. Lu , F. Chen , J. Comput. Chem. 2012, 33, 580–592, 10.1002/jcc.22885. 22162017

[87] T. Lu , J. Chem. Phys. 2024, 161, 082503, 10.1063/5.0216272. 39189657

[88] W. Humphrey , A. Dalke , K. Schulten , J. Mol. Graph. 1996, 14, 33–38, 10.1016/0263-7855(96)00018-5. 8744570

[89] K. D. Ashtekar , M. Wetticatt , R. Yousefi , J. E. Jackson , B. Borhan , J. Am. Chem. Soc. 2016, 138, 8114–8119, 10.1021/jacs.6b02877. 27284808 PMC5340197

[90] T. Ding , L. Long , M.‐J. Yuan , W. Xu , S.‐Y. Wong , H. Gao , Y.‐H. Wang , Y.‐Y. Yeung , X. Jiang , J. Am. Chem. Soc. 2025, 147, 16225–16236, 10.1021/jacs.5c01224. 40302362

[91] N. Tampellini , B. Q. Mercado , S. J. Miller , J. Am. Chem. Soc. 2025, 147, 4624–4630, 10.1021/jacs.4c17080. 39847512 PMC11815475

