# Supplementary material for: Halide Bond Assisted Double Desymmetrization of Meso‐Dicarboxylic Acids with Symmetrical Olefins via Asymmetric Halogenation
Source: Angew Chem Int Ed Engl. 2026 Jan 2;65(7):e20866. doi: 10.1002/anie.202520866 (PMC12887640; doi:10.1002/anie.202520866)
Supplement: Supplementary file 1 — Supporting Information [file ANIE-65-e20866-s001.pdf]

Supporting Information

Halide Bond Assisted Double Desymmetrization of *meso*-Dicarboxylic acids with Symmetrical Olefins via Asymmetric Halogenation

Qingyu Zhang, Haihui Wang, Ying-Lung Steve Tse,* Ying-Yeung Yeung*

Department of Chemistry and State Key Laboratory of Synthetic Chemistry, The Chinese University of Hong Kong, Shatin, NT, Hong Kong.

Email: stevetse@cuhk.edu.hk; yyyeung@cuhk.edu.hk

Table of Contents

1. General methods and materials 2
2. Preparation of catalysts 3
3. Preparation of diacid substrates 13
4. Desymmetrizing asymmetric halocyclization of diacid substrates 20
5. Formal synthesis of (–)-longifolene 39
6. Catalyst screening 41
7. Kinetic studies 42
8. NMR experiments 45
9. Computational studies 46
10. References 113
11. NMR Spectra 115
12. X-Ray crystallographic data 173

1. General methods and materials

Commercially available reagents were used as received. Halogen sources were purified by recrystallization prior to use. The solvents were dried by distillation over the appropriate drying reagents or solvent purification system (Inert PS-MD-7). NMR spectra were recorded on a Bruker AMX500 (500 MHz) spectrometer or a Bruker AMX400 (400 MHz) spectrometer in $CDCl_3$, acetone-d_6 or methanol-d_4. Chemical shifts are reported as δ values relative to internal chloroform (δ 7.26 for ^{1}H NMR and 77.00 for ^{13}C NMR), acetone-d_6 (δ 2.05 for ^{1}H NMR and 29.84 for ^{13}C NMR), methanol-d_4 (δ 3.31 for ^{1}H NMR and 49.00 for ^{13}C NMR). High resolution mass spectra were obtained on a Finnigan/MAT 95XL-T spectrometer (ionization mode: ESI or APCI). X-ray crystallography data were collected on a Bruker AXS Kappa ApexII Duo Diffractometer. Unless otherwise specified, all melting points were measured with the samples after column chromatography (without recrystallization and correction). Analytical thin layer chromatography (TLC) was performed with Merck pre-coated TLC plates, silica gel 60F-254, layer thickness 0.25 mm. Column chromatography was performed on Merck 60 (0.040-0.063 mm) mesh silica gel. All reactions sensitive to air or moisture were carried out under an argon atmosphere in dry and freshly distilled solvents under anhydrous conditions, unless otherwise noted.

2. Preparation of catalysts[1]

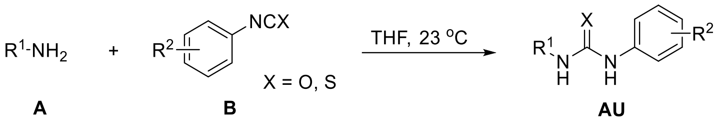

General procedure for the preparation of catalysts

To a solution of amine **A** (1.0 mmol, 1.0 equiv) in anhydrous THF (5.0 mL, 0.2 M) was added isocyanate or isothiocyanate **B** (1.0 mmol, 1.0 equiv) and the mixture was stirred at room temperature. When all starting material was consumed (TLC analysis), the mixture was concentrated under reduced pressure. The residue was purified by flash column chromatography (EtOAc: MeOH = 5:1) to give the product **AU**.

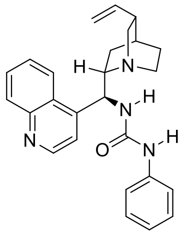

AU1, 1-phenyl-3-((S)-quinolin-4-yl((1S,2S,4S,5R)-5-vinylquinuclidin-2-yl)methyl)urea

Yield: 54%.

Physical state: white foam.

^{1}H NMR (500 MHz, Chloroform-*d*) δ 8.86 (d, *J* = 4.6 Hz, 1H), 8.44 (d, *J* = 8.5 Hz, 1H), 8.13 (dd, *J* = 8.4, 1.2 Hz, 1H), 7.83 (s, 1H), 7.74 – 7.67 (m, 1H), 7.59 (t, *J* = 7.7 Hz, 1H), 7.45 (d, *J* = 4.6 Hz, 1H), 7.23 – 7.13 (m, 4H), 7.00 – 6.92 (m, 1H), 6.67 (d, *J* = 3.7 Hz, 1H), 5.65 – 5.55 (m, 1H), 5.39 (br, 1H), 4.94 – 4.85 (m, 2H), 3.21 – 3.09 (m, 1H), 3.07 – 2.90 (m, 2H), 2.60 – 2.47 (m, 2H), 2.26 – 2.14 (m, 1H), 1.65 – 1.57 (m, 2H), 1.56 – 1.44 (m, 1H), 1.33 – 1.26 (m, 1H), 0.90 (dd, *J* = 13.8, 6.8 Hz, 1H).

^{13}C NMR (126 MHz, Chloroform-*d*) δ 155.8, 150.0, 148.4, 140.8, 138.9, 130.2, 129.2, 128.8, 127.2, 126.8, 123.4, 122.9, 119.7, 114.7, 55.4, 40.8, 39.1, 27.4, 27.1, 25.7.

HRMS (ESI) calcd for $C_{26}H_{28}N_4ONa^+$ $[M+Na]^+$: 435.2155; found: 435.2158.

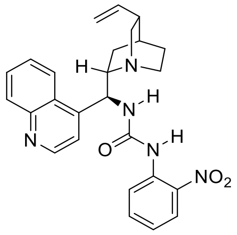

AU2, 1-(2-nitrophenyl)-3-((S)-quinolin-4-yl((1S,2S,4S,5R)-5-vinylquinuclidin-2-yl)methyl)urea

Yield: 40%.

Physical state: yellow foam.

^{1}H NMR (500 MHz, Chloroform-*d*) δ 9.83 (s, 1H), 8.87 (d, *J* = 4.5 Hz, 1H), 8.45 (t, *J* = 9.3 Hz, 2H), 8.11 (dd, *J* = 15.7, 8.5 Hz, 2H), 7.71 (t, *J* = 7.7 Hz, 1H), 7.63 (t, *J* = 7.8 Hz, 1H), 7.57 – 7.47 (m, 2H), 7.42 (t, *J* = 8.0 Hz, 1H), 6.95 (t, *J* = 7.8 Hz, 1H), 5.73 – 5.61 (m, 1H), 5.46 (br, 1H), 5.02 – 4.92 (m, 2H), 3.33 – 3.16 (m, 3H), 2.86 – 2.72 (m, 2H), 2.39 – 2.29 (m, 1H), 1.75 – 1.64 (m, 3H), 1.41 – 1.34 (m, 1H), 1.03 – 0.95 (m, 1H).

^{13}C NMR (126 MHz, Chloroform-*d*) δ 154.1, 150.1, 148.5, 140.4, 136.6, 135.6, 135.4, 130.5, 129.2, 126.9, 125.5, 123.0, 115.1, 55.3, 40.7, 39.0, 27.2, 27.2, 25.4.

HRMS (ESI) calcd for $C_{26}H_{27}N_5O_3Na^+$ $[M+Na]^+$: 480.2006; found: 480.2010.

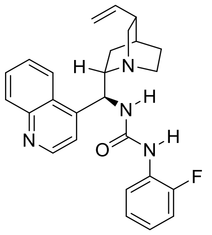

AU3, 1-(2-fluorophenyl)-3-((S)-quinolin-4-yl((1S,2S,4S,5R)-5-vinylquinuclidin-2-yl)methyl)urea

Yield: 73%.

Physical state: pale yellow foam.

^{1}H NMR (500 MHz, Chloroform-*d*) δ 8.86 (d, *J* = 4.5 Hz, 1H), 8.44 (d, *J* = 8.5 Hz, 1H), 8.12 (d, *J* = 8.6 Hz, 1H), 8.02 (t, *J* = 8.2 Hz, 1H), 7.70 (t, *J* = 7.7 Hz, 1H), 7.64 – 7.53 (m, 2H), 7.50 – 7.42 (m, 2H), 7.00 – 6.90 (m, 2H), 6.89 – 6.80 (m, 1H), 5.67 – 5.58 (m, 1H), 5.49 (br, 1H), 4.99 – 4.89 (m, 2H), 3.30 – 3.12 (m, 3H), 2.76 – 2.58 (m, 2H), 2.35 – 2.26 (m, 1H), 1.73 – 1.59 (m, 3H), 1.38 – 1.30 (m, 1H), 0.99 – 0.92 (m, 1H).

^{13}C NMR (126 MHz, Chloroform-*d*) δ 155.0, 153.1, 151.2, 150.1, 148.5, 140.2, 130.3, 129.2, 127.5 (d, *J* = 10.0 Hz), 126.9, 124.3 (d, *J* = 3.3 Hz), 123.2, 122.3 (d, *J* = 7.4 Hz), 120.9, 115.1, 114.5 (d, *J* = 19.2 Hz), 55.1, 40.6, 38.8, 27.1, 27.0, 25.3.

^{19}F NMR (471 MHz, Chloroform-*d*) δ -131.7.

HRMS (ESI) calcd for $C_{26}H_{27}FN_4ONa^+$ $[M+Na]^+$: 453.2061; found: 453.2063.

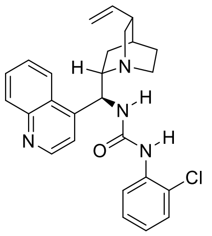

AU4, 1-(2-chlorophenyl)-3-((S)-quinolin-4-yl((1S,2S,4S,5R)-5-vinylquinuclidin-2-yl)methyl)urea

Yield: 69%.

Physical state: pale pink solid.

^{1}H NMR (500 MHz, Chloroform-*d*) δ 8.87 (d, *J* = 4.5 Hz, 1H), 8.44 (dd, *J* = 8.6, 1.3 Hz, 1H), 8.13 (dd, *J* = 8.4, 1.2 Hz, 1H), 8.05 (dd, *J* = 8.4, 1.5 Hz, 1H), 7.73 – 7.67 (m, 1H), 7.59 (t, *J* = 7.7 Hz, 1H), 7.48 (d, *J* = 4.6 Hz, 1H), 7.44 – 7.37 (m, 1H), 7.20 (dt, *J* = 7.9, 1.4 Hz, 1H), 7.14 – 7.06 (m, 1H), 7.05 – 6.99 (m, 1H), 6.84 (td, *J* = 7.7, 1.4 Hz, 1H), 5.67 – 5.57 (m, 1H), 5.37 (br, 1H), 4.94 – 4.84 (m, 2H), 3.18 – 2.98 (m, 3H), 2.69 – 2.58 (m, 2H), 2.28 – 2.19 (m, 1H), 1.64 – 1.52 (m, 3H), 1.33 – 1.24 (m, 1H), 0.98 – 0.85 (m, 1H).

^{13}C NMR (126 MHz, Chloroform-*d*) δ 154.9, 150.0, 148.4, 140.8, 135.6, 130.2, 129.1, 128.8, 127.3, 126.8, 123.2, 122.9, 122.1, 121.0, 114.6, 55.5, 40.7, 39.2, 27.5, 27.2, 25.6.

HRMS (ESI) calcd for $C_{26}H_{27}ClN_4ONa^+$ $[M+Na]^+$: 469.1766; found: 469.1770.

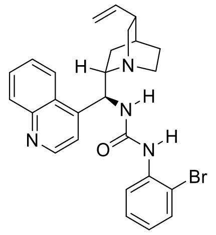

AU5, 1-(2-bromophenyl)-3-((S)-quinolin-4-yl((1S,2S,4S,5R)-5-vinylquinuclidin-2-yl)methyl)urea

Yield: 85%.

Physical state: white foam.

^{1}H NMR (400 MHz, Chloroform-*d*) δ 8.89 (d, *J* = 4.5 Hz, 1H), 8.44 (d, *J* = 8.4 Hz, 1H), 8.14 (d, *J* = 8.3 Hz, 1H), 8.01 (dd, *J* = 8.3, 1.6 Hz, 1H), 7.71 (t, *J* = 7.3 Hz, 1H), 7.61 (t, *J* = 7.5 Hz, 1H), 7.51 (d, *J* = 4.5 Hz, 1H), 7.44 – 7.35 (m, 1H), 7.18 – 7.11 (m, 1H), 7.06 (s, 1H), 6.80 (td, *J* = 7.7, 1.6 Hz, 1H), 6.74 (s, 1H), 5.72 – 5.58 (m, 1H), 5.32 (br, 1H), 4.99 – 4.87 (m, 2H), 3.26 – 3.09 (m, 2H), 3.08 – 2.94 (m, 1H), 2.78 – 2.61 (m, 2H), 2.27 (q, *J* = 9.9, 7.6 Hz, 1H), 1.66 – 1.56 (m, 2H), 1.37 – 1.19 (m, 2H), 0.96 (dd, *J* = 13.7, 6.9 Hz, 1H).

^{13}C NMR (101 MHz, Chloroform-*d*) δ 154.9, 150.1, 148.5, 141.1, 136.6, 132.1, 130.4, 129.1, 128.1, 126.8, 123.7, 123.2, 121.4, 114.6, 112.9, 55.7, 40.8, 39.4, 27.7, 27.2, 25.6.

HRMS (ESI) calcd for $C_{26}H_{26}BrN_4ONa^+$ $[M+Na]^+$: 513.1260 (Br^{79}), 515.1240 (Br^{81}); found: 513.1267 (Br^{79}), 515.1245 (Br^{81}).

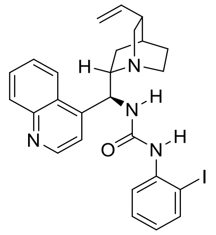

AU6, 1-(2-iodophenyl)-3-((S)-quinolin-4-yl((1S,2S,4S,5R)-5-vinylquinuclidin-2-yl)methyl)urea

Yield: 68%.

Physical state: pale yellow foam.

^{1}H NMR (500 MHz, Chloroform-*d*) δ 8.87 (d, *J* = 4.5 Hz, 1H), 8.44 (d, *J* = 8.5 Hz, 1H), 8.13 (d, *J* = 8.4 Hz, 1H), 7.84 (d, *J* = 8.3 Hz, 1H), 7.70 (t, *J* = 7.7 Hz, 1H), 7.65 (d, *J* = 7.9 Hz, 1H), 7.58 (t, *J* = 7.7 Hz, 1H), 7.49 (d, *J* = 4.5 Hz, 1H), 7.19 (t, *J* = 7.8 Hz, 1H), 6.92 (s, 1H), 6.83 (s, 1H), 6.69 (t, *J* = 7.6 Hz, 1H), 5.72 – 5.57 (m, 1H), 5.33 (br, 1H), 4.98 – 4.85 (m, 2H), 3.23 – 3.09 (m, 2H), 3.08 – 2.93 (m, 1H), 2.74 – 2.61 (m, 2H), 2.32 – 2.20 (m, 1H), 1.62 – 1.48 (m, 2H), 1.36 – 1.20 (m, 2H), 0.93 (dd, *J* = 13.9, 6.8 Hz, 1H).

^{13}C NMR (126 MHz, Chloroform-*d*) δ 155.2, 150.1, 148.5, 141.1, 139.2, 138.7, 130.3, 129.1, 128.9, 127.3, 126.7, 124.8, 123.3, 122.3, 114.5, 90.5, 55.7, 40.8, 39.4, 27.7, 27.2, 25.7.

HRMS (ESI) calcd for $C_{26}H_{27}IN_4ONa^+$ $[M+Na]^+$: 561.1122; found: 561.1124.

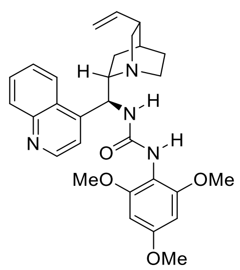

AU7, 1-((S)-quinolin-4-yl((1S,2S,4S,5R)-5-vinylquinuclidin-2-yl)methyl)-3-(3,4,5-trimethoxyphenyl)urea

Yield: 85%.

Physical state: pale yellow foam.

^{1}H NMR (500 MHz, Chloroform-*d*) δ 8.85 (d, *J* = 4.5 Hz, 1H), 8.40 (d, *J* = 8.4 Hz, 1H), 8.11 (dd, *J* = 8.5, 1.3 Hz, 1H), 7.89 (s, 1H), 7.70 (t, *J* = 7.5 Hz, 1H), 7.60 (t, *J* = 7.7 Hz, 1H), 7.47 (d, *J* = 4.6 Hz, 1H), 6.88 (d, *J* = 4.7 Hz, 1H), 6.53 (s, 2H), 5.61 – 5.52 (m, 1H), 5.43 (br, 1H), 4.95 – 4.85 (m, 2H), 3.74 (s, 3H), 3.70 (s, 6H), 3.29 – 3.08 (m, 2H), 2.93 – 2.81 (m, 1H), 2.66 – 2.55 (m, 1H), 2.47 – 2.34 (m, 1H), 2.18 (dt, *J* = 11.4, 6.8 Hz, 1H), 1.65 – 1.56 (m, 2H), 1.54 – 1.44 (m, 1H), 1.33 (td, *J* = 11.7, 10.1, 3.5 Hz, 1H), 0.89 (dd, *J* = 14.0, 6.6 Hz, 1H).

^{13}C NMR (126 MHz, Chloroform-*d*) δ 155.3, 153.2, 150.1, 148.3, 139.9, 135.1, 133.5, 130.2, 129.3, 127.1, 126.9, 123.3, 115.2, 97.3, 60.8, 55.9, 55.0, 40.8, 38.5, 26.9, 26.8, 25.4.

HRMS (ESI) calcd for $C_{29}H_{34}N_4O_4Na^+$ $[M+Na]^+$: 525.2472; found: 525.2479.

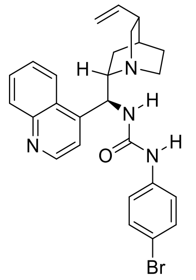

AU8, 1-(4-bromophenyl)-3-((S)-quinolin-4-yl((1S,2S,4S,5R)-5-vinylquinuclidin-2-yl)methyl)urea

Yield: 60%.

Physical state: pale yellow foam.

^{1}H NMR (500 MHz, Chloroform-*d*) δ 8.85 (d, *J* = 4.6 Hz, 1H), 8.64 (s, 1H), 8.36 (d, *J* = 8.5 Hz, 1H), 8.08 (d, *J* = 8.3 Hz, 1H), 7.69 (t, *J* = 7.7 Hz, 1H), 7.62 (t, *J* = 7.7 Hz, 1H), 7.56 (d, *J* = 4.6 Hz, 1H), 7.36 (d, *J* = 6.9 Hz, 1H), 7.25 – 7.22 (m, 3H), 7.11 (br, 1H), 5.68 (br, 1H), 5.58 – 5.45 (m, 1H), 5.03 – 4.91 (m, 2H), 3.75 – 3.42 (m, 2H), 3.10 (dd, *J* = 13.6, 10.4 Hz, 1H), 2.80 – 2.60 (m, 2H), 2.31 (q, *J* = 7.9 Hz, 1H), 1.80 – 1.62 (m, 3H), 1.47 (t, *J* = 12.2 Hz, 1H), 0.92 (dd, *J* = 13.2, 5.0 Hz, 1H).

^{13}C NMR (126 MHz, Chloroform-*d*) δ 155.0, 150.2, 148.4, 145.3, 138.3, 138.1, 131.6, 130.2, 129.5, 127.4, 126.8, 123.0, 120.6, 116.5, 114.8, 54.5, 41.2, 37.6, 26.7, 25.5, 24.7.

HRMS (ESI) calcd for $C_{26}H_{26}BrN_5O_3Na^+$ $[M+Na]^+$: 513.1260 (Br^{79}), 515.1240 (Br^{81}); found: 513.1265 (Br^{79}), 515.1243 (Br^{81}).

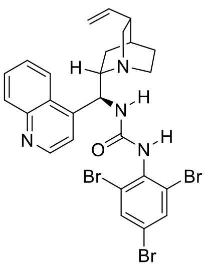

AU9, 1-((S)-quinolin-4-yl((1S,2S,4S,5R)-5-vinylquinuclidin-2-yl)methyl)-3-(2,4,6-tribromophenyl)urea

Yield: 60%.

Physical state: pale yellow solid.

^{1}H NMR (500 MHz, Chloroform-*d*) δ 8.76 (d, *J* = 4.4 Hz, 1H), 8.34 (d, *J* = 8.5 Hz, 1H), 7.94 (d, *J* = 8.0 Hz, 1H), 7.68 – 7.56 (m, 3H), 7.51 (t, *J* = 7.5 Hz, 1H), 7.47 (d, *J* = 4.4 Hz, 1H), 7.18 (br, 1H), 5.62 – 5.51 (m, 1H), 5.35 (br, 1H), 4.95 – 4.84 (m, 2H), 3.29 – 3.19 (m, 1H), 3.09 (s, 1H), 2.97 (dd, *J* = 14.0, 10.2 Hz, 1H), 2.68 – 2.59 (m, 1H), 2.58 – 2.48 (m, 1H), 2.26 – 2.15 (m, 1H), 1.72 – 1.64 (m, 1H), 1.59 – 1.51 (m, 1H), 1.31 – 1.15 (m, 2H), 0.99 – 0.90 (m, 1H).

^{13}C NMR (126 MHz, Chloroform-*d*) δ 156.0, 149.9, 148.2, 140.2, 134.9, 134.6, 130.2, 129.0, 127.0, 126.7, 125.7, 123.3, 121.2, 115.0, 55.0, 40.8, 38.7, 27.0, 25.2.

HRMS (ESI) calcd for $C_{26}H_{25}Br_3N_4ONa^+$ $[M+Na]^+$: 670.9452 (Br^{79}, Br^{79}, Br^{79}), 672.9430 (Br^{79}, Br^{79}, Br^{81}), 674.9409 (Br^{79}, Br^{81}, Br^{81}), 676.9476 (Br^{81}, Br^{81}, Br^{81}); found: 670.9457 (Br^{79}, Br^{79}, Br^{79}), 672.9436 (Br^{79}, Br^{79}, Br^{81}), 674.9413 (Br^{79}, Br^{81}, Br^{81}), 676.9472 (Br^{81}, Br^{81}, Br^{81}).

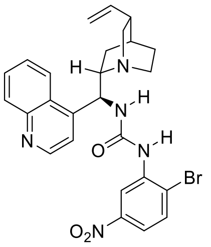

AU10, 1-(2-bromo-5-nitrophenyl)-3-((S)-quinolin-4-yl((1S,2S,4S,5R)-5-vinylquinuclidin-2-yl)methyl)urea

Yield: 51%.

Physical state: yellow solid.

^{1}H NMR (500 MHz, Chloroform-*d*) δ 8.98 (d, *J* = 2.7 Hz, 1H), 8.91 (d, *J* = 4.5 Hz, 1H), 8.44 (d, *J* = 8.4 Hz, 1H), 8.15 (dd, *J* = 8.5, 1.3 Hz, 1H), 7.77 – 7.70 (m, 1H), 7.68 – 7.60 (m, 2H), 7.55 – 7.52 (m, 2H), 7.50 (s, 1H), 7.30 (s, 1H), 5.70 – 5.59 (m, 1H), 5.48 (br, 1H), 4.98 – 4.90 (m, 2H), 3.28 – 3.13 (m, 3H), 2.78 – 2.67 (m, 2H), 2.36 – 2.26 (m, 1H), 1.72 – 1.60 (m, 3H), 1.42 – 1.32 (m, 1H), 0.98 (dd, *J* = 13.9, 6.7 Hz, 1H).

^{13}C NMR (126 MHz, Chloroform-*d*) δ 154.2, 150.1, 148.5, 147.6, 140.4, 138.0, 132.6, 130.4, 129.4, 127.1, 123.1, 118.8, 117.5, 115.3, 115.1, 55.5, 40.9, 39.0, 27.2, 27.1, 25.5.

HRMS (ESI) calcd for $C_{26}H_{26}BrN_5O_3Na^+$ $[M+Na]^+$: 558.1111 (Br^{79}), 560.1091 (Br^{81}); found: 558.1115 (Br^{79}), 560.1092 (Br^{81}).

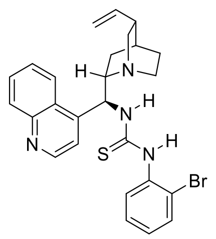

AU11, 1-(2-bromophenyl)-3-((S)-quinolin-4-yl((1S,2S,4S,5R)-5-vinylquinuclidin-2-yl)methyl)thiourea

Yield: 60%.

Physical state: pale yellow foam.

^{1}H NMR (500 MHz, Chloroform-*d*) δ 8.78 (br, 1H), 8.67 (d, *J* = 4.5 Hz, 1H), 8.40 (d, *J* = 8.5 Hz, 1H), 8.09 (d, *J* = 8.3 Hz, 1H), 7.68 (t, *J* = 7.9 Hz, 1H), 7.64 – 7.51 (m, 3H), 7.38 – 7.29 (m, 2H), 7.15 (td, *J* = 7.7, 1.6 Hz, 1H), 5.73 (br, 1H), 5.65 – 5.52 (m, 1H), 4.96 – 4.83 (m, 2H), 3.24 – 3.12 (m, 1H), 3.11 – 2.96 (m, 2H), 2.70 – 2.56 (m, 2H), 2.29 – 2.14 (m, 1H), 1.75 – 1.61 (m, 2H), 1.59 – 1.50 (m, 1H), 1.25 – 1.19 (m, 1H), 0.99 (dd, *J* = 14.0, 7.0 Hz, 1H).

^{13}C NMR (126 MHz, Chloroform-*d*) δ 181.3, 150.0, 148.4, 140.7, 136.3, 133.4, 130.3, 129.0, 128.3, 128.2, 128.0, 127.1, 126.5, 123.7, 120.7, 114.7, 55.1, 41.0, 39.2, 27.5, 27.1, 25.5.

HRMS (ESI) calcd for $C_{26}H_{27}BrN_4SNa^+$ $[M+Na]^+$: 529.1032 (Br^{79}), 531.1014 (Br^{81}); found: 529.1033 (Br^{79}), 531.1015 (Br^{81}).

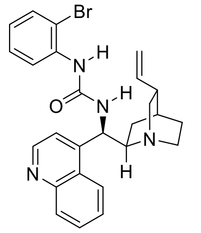

AU12, 1-(2-bromophenyl)-3-((R)-quinolin-4-yl((1S,2R,4S,5R)-5-vinylquinuclidin-2-yl)methyl)urea

Yield: 64%.

Physical state: pale yellow foam.

^{1}H NMR (500 MHz, Chloroform-*d*) δ 8.88 (d, *J* = 4.4 Hz, 1H), 8.42 (d, *J* = 8.5 Hz, 1H), 8.14 (d, *J* = 8.4 Hz, 1H), 8.02 (dd, *J* = 8.4, 1.6 Hz, 1H), 7.76 – 7.66 (m, 1H), 7.59 (t, *J* = 7.6 Hz, 1H), 7.49 (dd, *J* = 4.6, 2.2 Hz, 1H), 7.38 (d, *J* = 8.0 Hz, 1H), 7.20 – 7.10 (m, 2H), 6.84 – 6.72 (m, 2H), 5.95 – 5.84 (m, 1H), 5.31 (br, 1H), 5.19 – 5.07 (m, 2H), 2.98 – 2.83 (m, 5H), 2.27 (q, *J* = 8.2 Hz, 1H), 1.61 (s, 1H), 1.57 – 1.47 (m, 1H), 1.47 – 1.38 (m, 1H), 1.33 – 1.23 (m, 1H), 0.91 – 0.81 (m, 1H).

^{13}C NMR (126 MHz, Chloroform-*d*) δ 155.0, 155.0, 150.0, 148.5, 140.1, 136.7, 136.7, 132.0, 130.3, 129.1, 128.0, 127.3, 126.7, 123.6, 123.3, 121.4, 121.4, 114.9, 112.8, 112.8, 49.0, 46.9, 39.1, 27.3, 26.4, 25.0.

HRMS (ESI) calcd for $C_{26}H_{27}BrN_4ONa^+$ $[M+Na]^+$: 513.1260 (Br^{79}), 515.1240 (Br^{81}); found: 513.1265 (Br^{79}), 515.1243 (Br^{81}).

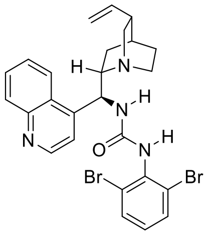

AU13, 1-(2,6-dibromophenyl)-3-((S)-quinolin-4-yl((1S,2S,4S,5R)-5-vinylquinuclidin-2-yl)methyl)urea

Yield: 53%.

Physical state: white solid.

^{1}H NMR (500 MHz, Chloroform-*d*) δ 8.84 – 8.64 (m, 1H), 8.50 – 8.31 (m, 1H), 8.03 – 7.84 (m, 1H), 7.73 – 7.42 (m, 5H), 7.40 – 7.18 (m, 2H), 7.13 – 6.86 (m, 1H), 5.68 – 5.50 (m, 1H), 5.35 (br, 1H), 5.09 – 4.78 (m, 2H), 3.35 – 3.15 (m, 1H), 3.12 – 2.81 (m, 2H), 2.73 – 2.44 (m, 2H), 2.30 – 2.10 (m, 1H), 1.73 – 1.46 (m, 3H), 1.31 – 1.17 (m, 1H), 1.08 – 0.89 (m, 1H).

^{13}C NMR (126 MHz, Chloroform-*d*) δ 156.5, 149.8, 148.1, 140.6, 135.5, 132.2, 130.1, 129.4, 128.8, 127.0, 126.5, 125.5, 123.4, 114.7, 55.1, 40.8, 38.9, 27.3, 27.1, 25.3.

HRMS (ESI) calcd for $C_{26}H_{26}Br_2N_4ONa^+$ $[M+Na]^+$: 593.0347 (Br^{79}, Br^{79}), 595.0325 (Br^{79}, Br^{81}), 597.0392 (Br^{81}, Br^{81}); found: 593.0348 (Br^{79}, Br^{79}), 595.0325 (Br^{79}, Br^{81}), 597.0386 (Br^{81}, Br^{81}).

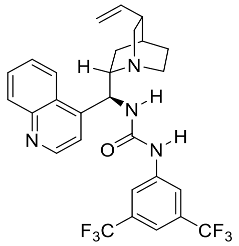

AU14, 1-(3,5-bis(trifluoromethyl)phenyl)-3-((S)-quinolin-4-yl((1S,2S,4S,5R)-5-vinylquinuclidin-2-yl)methyl)urea

Yield: 81%.

Physical state: pale yellow foam.

^{1}H NMR (500 MHz, Chloroform-*d*) δ 8.91 (d, *J* = 4.6 Hz, 2H), 8.44 (d, *J* = 8.5 Hz, 1H), 8.17 (d, *J* = 8.4 Hz, 1H), 7.83 – 7.71 (m, 3H), 7.64 (t, *J* = 7.7 Hz, 1H), 7.50 (d, *J* = 4.5 Hz, 1H), 7.36 (s, 1H), 6.75 (s, 1H), 5.71 – 5.20 (m, 2H), 4.95 – 4.79 (m, 2H), 3.25 – 2.84 (m, 3H), 2.65 – 2.46 (m, 1H), 2.42 – 2.29 (m, 1H), 2.27 – 2.14 (m, 1H), 1.59 – 1.47 (m, 2H), 1.38 – 1.25 (m, 2H), 0.93 (dd, *J* = 13.8, 6.9 Hz, 1H).

^{13}C NMR (126 MHz, Chloroform-*d*) δ 155.0, 149.8, 148.4, 140.8 (d, *J* = 43.1 Hz), 131.9 (q, *J* = 33.2 Hz), 129.8 (d, *J* = 45.1 Hz), 127.2, 126.4, 124.2, 123.3, 122.0, 117.9 (d, *J* = 4.0 Hz), 115.2 (t, *J* = 3.9 Hz), 114.8, 55.3, 40.7, 39.0, 27.4, 27.0, 25.7.

^{19}F NMR (471 MHz, Chloroform-*d*) δ -63.0.

HRMS (ESI) calcd for $C_{28}H_{26}F_6N_4ONa^+$ $[M+Na]^+$: 571.1903; found: 571.1909.

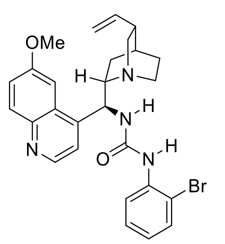

AU15, 1-(2-bromophenyl)-3-((S)-(6-methoxyquinolin-4-yl)((1S,2S,4S,5R)-5-vinylquinuclidin-2-yl)methyl)urea

Yield: 53%.

Physical state: pale yellow foam.

^{1}H NMR (500 MHz, Chloroform-*d*) δ 8.75 – 8.66 (m, 1H), 8.09 – 7.95 (m, 2H), 7.71 (s, 1H), 7.45 – 7.31 (m, 3H), 7.15 (t, *J* = 7.5 Hz, 1H), 7.07 (s, 1H), 6.80 (t, *J* = 7.3 Hz, 1H), 6.72 (s, 1H), 5.75 – 5.62 (m, 1H), 5.28 (br, 1H), 5.02 – 4.88 (m, 2H), 3.96 (s, 3H), 3.28 – 3.16 (m, 2H), 3.08 (s, 1H), 2.91 – 2.57 (m, 2H), 2.28 (s, 1H), 1.72 – 1.53 (m, 3H), 1.39 (t, *J* = 12.0 Hz, 1H), 0.97 (dd, *J* = 14.2, 6.7 Hz, 1H).

^{13}C NMR (126 MHz, Chloroform-*d*) δ 157.8, 154.9, 147.6, 144.7, 141.0, 136.7, 132.1, 131.7, 128.3, 128.0, 123.7, 114.7, 113.0, 101.8, 55.8, 55.6, 40.9, 39.4, 27.8, 27.3, 25.9.

HRMS (ESI) calcd for $C_{27}H_{29}BrN_4O_2Na^+$ $[M+Na]^+$: 543.1366 (Br^{79}), 545.1346 (Br^{81}); found: 543.1372 (Br^{79}), 545.1350 (Br^{81}).

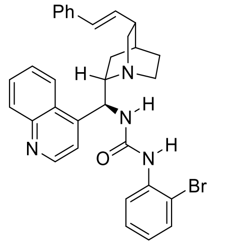

AU16, 1-(2-bromophenyl)-3-((S)-quinolin-4-yl((1S,2S,4S,5S)-5-((E)-styryl)quinuclidin-2-yl)methyl)urea

Yield: 62%.

Physical state: white foam.

^{1}H NMR (500 MHz, Chloroform-*d*) δ 8.91 (d, *J* = 4.6 Hz, 1H), 8.46 (d, *J* = 8.6 Hz, 1H), 8.15 (d, *J* = 8.5 Hz, 1H), 8.03 (d, *J* = 8.2 Hz, 1H), 7.72 (t, *J* = 7.6 Hz, 1H), 7.62 (t, *J* = 7.7 Hz, 1H), 7.56 (d, *J* = 4.6 Hz, 1H), 7.39 (d, *J* = 7.9 Hz, 1H), 7.25 – 7.22 (m, 5H), 7.18 – 7.14 (m, 2H), 7.04 (s, 1H), 6.81 (t, *J* = 7.6 Hz, 1H), 6.33 (d, *J* = 15.8 Hz, 1H), 6.03 (dd, *J* = 15.8, 7.9 Hz, 1H), 5.43 (br, 1H), 3.31 (dd, *J* = 13.9, 10.2 Hz, 1H), 3.27 – 3.19 (m, 2H), 2.82 – 2.70 (m, 2H), 2.53 – 2.41 (m, 1H), 1.74 – 1.59 (m, 3H), 1.49 – 1.39 (m, 1H), 1.03 (dd, *J* = 14.1, 6.4 Hz, 1H).

^{13}C NMR (126 MHz, Chloroform-*d*) δ 154.9, 150.2, 148.5, 136.9, 136.7, 132.2, 132.1, 130.5, 130.4, 129.2, 128.4, 128.0, 127.2, 126.9, 125.9, 123.8, 123.2, 121.6, 113.1, 56.2, 40.8, 38.8, 27.6, 27.3, 25.6.

HRMS (ESI) calcd for $C_{32}H_{31}BrN_4ONa^+$ $[M+Na]^+$: 589.1574 (Br^{79}), 591.1553 (Br^{81}); found: 589.1580 (Br^{79}), 591.1556 (Br^{81}).

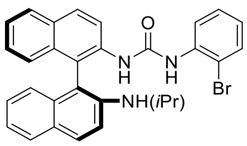

AU17, (S)-1-(2-bromophenyl)-3-(2'-(isopropylamino)-[1,1'-binaphthalen]-2-yl)urea

Yield: 97%.

Physical state: pale yellow solid.

^{1}H NMR (500 MHz, Chloroform-*d*) δ 8.46 (d, *J* = 9.0 Hz, 1H), 8.01 (d, *J* = 9.0 Hz, 1H), 7.92 (d, *J* = 8.1 Hz, 1H), 7.85 (d, *J* = 9.0 Hz, 1H), 7.76 (d, *J* = 7.9 Hz, 1H), 7.45 – 7.33 (m, 3H), 7.26 – 7.23 (m, 1H), 7.23 – 7.17 (m, 2H), 7.17 – 7.12 (m, 2H), 6.98 (td, *J* = 7.7, 1.5 Hz, 1H), 6.87 – 6.81 (m, 2H), 6.72 (br, 1H), 6.67 (br, 1H), 3.78 – 3.67 (m, 1H), 3.31 (br, 1H), 1.01 (d, *J* = 6.2 Hz, 3H), 0.97 (d, *J* = 6.3 Hz, 3H).

^{13}C NMR (126 MHz, Chloroform-*d*) δ 152.6, 135.5, 135.3, 133.7, 132.8, 132.4, 130.9, 130.2, 129.3, 128.2, 128.1, 128.1, 127.1, 126.7, 125.3, 125.2, 124.9, 123.4, 123.2, 122.2, 120.9, 120.4, 115.8, 114.9, 44.6, 23.0, 22.9.

HRMS (ESI) calcd for $C_{30}H_{26}BrN_3ONa^+$ $[M+Na]^+$: 546.1152 (Br^{79}), 548.1131 (Br^{81}); found: 546.1160 (Br^{79}), 548.1136 (Br^{81}).

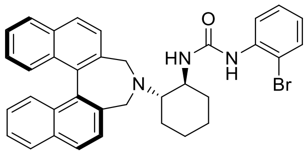

AU18, 1-(2-bromophenyl)-3-((1S,2S)-2-((S)-3,5-dihydro-4H-dinaphtho[2,1-c:1',2'-e]azepin-4-yl)cyclohexyl)urea

Yield: 77%.

Physical state: pale yellow solid.

^{1}H NMR (500 MHz, Chloroform-*d*) δ 8.13 (dd, *J* = 8.3, 1.5 Hz, 1H), 7.98 – 7.87 (m, 4H), 7.54 (t, *J* = 8.7 Hz, 3H), 7.46 (t, *J* = 7.5 Hz, 2H), 7.41 (d, *J* = 8.5 Hz, 2H), 7.28 – 7.18 (m, 5H), 7.00 (s, 1H), 6.88 (td, *J* = 7.8, 1.6 Hz, 1H), 6.15 (d, *J* = 4.1 Hz, 1H), 3.71 – 3.62 (m, 4H), 2.80 (t, *J* = 8.7 Hz, 1H), 2.50 (d, *J* = 12.2 Hz, 1H), 1.91 – 1.59 (m, 3H), 1.37 – 1.10 (m, 5H).

^{13}C NMR (126 MHz, Chloroform-*d*) δ 155.5, 137.1, 134.7, 133.8, 133.0, 132.3, 131.2, 128.9, 128.2, 128.1, 127.7, 127.4, 125.8, 125.5, 123.8, 122.0, 113.8, 69.4, 52.1, 33.6, 27.3, 25.6, 24.5.

HRMS (ESI) calcd for $C_{35}H_{32}BrN_3ONa^+$ $[M+Na]^+$: 612.1621 (Br^{79}), 614.1600 (Br^{81}); found: 612.1627 (Br^{79}), 614.1605 (Br^{81}).

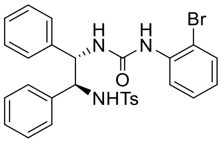

AU19, N-((1S,2S)-2-(3-(2-bromophenyl)ureido)-1,2-diphenylethyl)-4-methylbenzenesulfonamide

Yield: 99%.

Physical state: white solid.

^{1}H NMR (500 MHz, Chloroform-*d*) δ 7.91 (d, *J* = 8.1 Hz, 1H), 7.44 (d, *J* = 7.9 Hz, 2H), 7.37 (d, *J* = 7.9 Hz, 1H), 7.15 – 7.07 (m, 5H), 7.07 – 7.02 (m, 2H), 6.98 (t, *J* = 7.3 Hz, 1H), 6.95 – 6.88 (m, 4H), 6.86 – 6.78 (m, 4H), 6.61 (d, *J* = 6.7 Hz, 1H), 5.09 (t, *J* = 7.7 Hz, 1H), 4.74 – 4.62 (m, 1H), 2.24 (s, 3H).

^{13}C NMR (126 MHz, Chloroform-*d*) δ 155.6, 142.7, 138.7, 137.5, 137.4, 136.4, 132.1, 129.1, 128.4, 128.0, 128.0, 127.6, 127.4, 127.2, 126.8, 124.0, 122.1, 113.8, 63.5, 59.6, 21.3.

HRMS (ESI) calcd for $C_{28}H_{26}BrN_3O_3SNa^+$ $[M+Na]^+$: 586.0770 (Br^{79}), 588.0750 (Br^{81}); found: 586.0778 (Br^{79}), 588.0755 (Br^{81}).

3. Preparation of *meso*-diacid substrates 3 and 7[2,3]

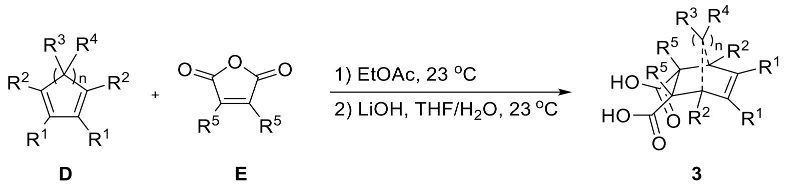

General procedure for the preparation of *meso*-diacid substrates 3

Dienes **D** were prepared according to the literature procedures.[4-9] To a solution of **E** (1.0 equiv) in EtOAc (1.0 M) was added **D** (1.0 equiv). The mixture was stirred at room temperature for 1 hour. The reaction mixture was concentrated under reduced pressure and the crude product was used in the next step directly.

To a solution of the above crude product in THF/H_2O (0.1 M, 1:1 v/v) was added LiOH (4.0 equiv). The mixture was stirred at room temperature for 1 hour. After removal of THF under reduced pressure, the aqueous phase was washed with dichloromethane. 2 N HCl was then added until the pH of the aqueous phase was ca. 2. The aqueous layer was then extracted twice with diethyl ether. The combined organic phase was dried over anhydrous Na_2SO_4, filtered, and concentrated under reduced pressure to give diacid **3**.

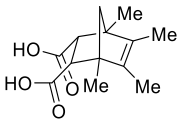

3a, (1S,2R,3S,4S)-1,4,5,6-tetramethylbicyclo[2.2.1]hept-5-ene-2,3-dicarboxylic acid

Yield: 61%.

Physical state: white solid.

^{1}H NMR (500 MHz, Acetone-*d*$_6$) δ 3.14 (s, 2H), 1.63 (s, 6H), 1.33 (d, *J* = 7.8 Hz, 1H), 1.30 (s, 6H), 1.15 (d, *J* = 7.8 Hz, 1H).

^{13}C NMR (126 MHz, Acetone-*d*$_6$) δ 173.5, 138.1, 62.9, 56.4, 54.5, 17.0, 11.8.

HRMS (APCI) calcd for $C_{13}H_{17}O_4^-$ $[M-H]^-$: 237.1132; found: 237.1130.

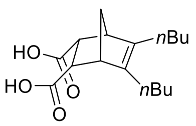

3b, (1R,2R,3S,4S)-5,6-dibutylbicyclo[2.2.1]hept-5-ene-2,3-dicarboxylic acid

Yield: 11%.

Physical state: yellow foam.

^{1}H NMR (500 MHz, Acetone-*d*$_6$) δ 3.30 (s, 2H), 3.05 (s, 2H), 2.24 – 2.14 (m, 2H), 2.12 – 2.07 (m, 2H), 1.55 – 1.44 (m, 2H), 1.39 – 1.27 (m, 8H), 0.90 (t, *J* = 7.2 Hz, 6H).

^{13}C NMR (126 MHz, Acetone-*d*$_6$) δ 174.4, 141.2, 50.0, 49.7, 48.7, 31.6, 27.9, 23.3, 14.3.

HRMS (ESI) calcd for $C_{17}H_{26}O_4Na^+$ $[M+Na]^+$: 317.1723; found: 317.1721.

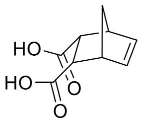

3c, (1R,2S,3R,4S)-bicyclo[2.2.1]hept-5-ene-2,3-dicarboxylic acid

Yield: 53%.

Physical state: white solid.

^{1}H NMR (400 MHz, DMSO-*d*$_6$) δ 11.80 (br, 2H), 6.09 (t, *J* = 1.8 Hz, 2H), 3.21 – 3.13 (m, 2H), 3.03 – 2.94 (m, 2H), 1.30 (d, *J* = 8.2 Hz, 1H), 1.24 (dt, *J* = 8.3, 1.9 Hz, 1H).

^{13}C NMR (101 MHz, DMSO-*d*$_6$) δ 173.5, 134.8, 48.3, 47.8, 45.9.

HRMS (APCI) calcd for $C_9H_9O_4^-$ [M-H]$^-$: 181.0506; found: 181.0506.

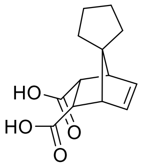

3d, (1R,4S,5R,6S)-spiro[bicyclo[2.2.1]heptane-7,1'-cyclopentan]-2-ene-5,6-dicarboxylic acid

Yield: 25%.

Physical state: white solid.

^{1}H NMR (500 MHz, Acetone-*d*$_6$) δ 6.17 – 6.10 (m, 2H), 3.41 – 3.34 (m, 2H), 2.72 (s, 2H), 1.62 – 1.51 (m, 6H), 1.48 – 1.41 (m, 2H).

^{13}C NMR (126 MHz, Acetone-*d*$_6$) δ 173.7, 136.2, 69.0, 54.8, 48.4, 33.0, 32.3, 26.5, 25.8.

HRMS (APCI) calcd for $C_{13}H_{15}O_4^-$ [M-H]$^-$: 235.0976; found: 235.0976.

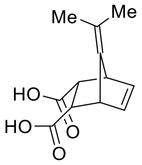

3e, (1R,2S,3R,4S)-7-(propan-2-ylidene)bicyclo[2.2.1]hept-5-ene-2,3-dicarboxylic acid

Yield: 34%.

Physical state: white solid.

^{1}H NMR (500 MHz, Acetone-*d*$_6$) δ 6.27 (t, *J* = 2.0 Hz, 2H), 3.60 (p, *J* = 1.9 Hz, 2H), 3.23 (t, *J* = 1.6 Hz, 2H), 1.56 (s, 6H).

^{13}C NMR (126 MHz, Acetone-*d*$_6$) δ 173.3, 146.2, 135.4, 109.5, 48.5, 46.8, 19.5.

HRMS (APCI) calcd for $C_{12}H_{13}O_4^-$ [M-H]$^-$: 221.0819; found: 221.0825.

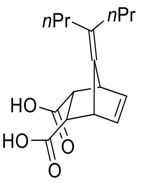

3f, (1R,2S,3R,4S)-7-(heptan-4-ylidene)bicyclo[2.2.1]hept-5-ene-2,3-dicarboxylic acid

Yield: 33%.

Physical state: pale yellow solid.

^{1}H NMR (500 MHz, Acetone-*d*$_6$) δ 6.31 – 6.28 (m, 2H), 3.60 (s, 2H), 3.25 (s, 2H), 1.99 – 1.90 (m, 4H), 1.42 – 1.32 (m, 4H), 0.86 (t, *J* = 7.3 Hz, 6H).

^{13}C NMR (126 MHz, Acetone-*d*$_6$) δ 173.3, 148.2, 135.6, 117.6, 48.6, 46.8, 33.5, 22.4, 14.1.

HRMS (APCI) calcd for $C_{16}H_{21}O_4^-$ [M-H]$^-$: 277.1445; found: 277.1446.

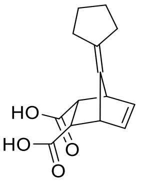

3g, (1R,2S,3R,4S)-7-cyclopentylidenebicyclo[2.2.1]hept-5-ene-2,3-dicarboxylic acid

Yield: 15%.

Physical state: pale yellow solid.

^{1}H NMR (500 MHz, Acetone-*d*$_6$) δ 6.27 (s, 2H), 3.43 (s, 2H), 3.26 (s, 2H), 2.20 – 2.07 (m, 4H), 1.65 – 1.52 (m, 4H).

^{13}C NMR (126 MHz, Acetone-*d*$_6$) δ 173.4, 142.4, 135.4, 120.0, 48.7, 47.9, 29.9, 27.2.

HRMS (APCI) calcd for $C_{14}H_{15}O_4^-$ [M-H]$^-$: 247.0976; found: 246.0975.

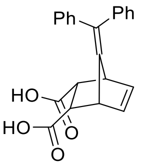

3h, (1R,2S,3R,4S)-7-(diphenylmethylene)bicyclo[2.2.1]hept-5-ene-2,3-dicarboxylic acid

Yield: 46%.

Physical state: pale yellow solid.

^{1}H NMR (400 MHz, Acetone-*d*$_6$) δ 7.39 – 7.30 (m, 4H), 7.29 – 7.23 (m, 2H), 7.16 – 7.09 (m, 4H), 6.42 (t, *J* = 2.0 Hz, 2H), 3.64 – 3.59 (m, 2H), 3.59 – 3.55 (m, 2H).

^{13}C NMR (101 MHz, Acetone-*d*$_6$) δ 172.9, 151.1, 141.7, 135.6, 130.2, 128.9, 127.7, 122.6, 48.6, 48.0.

HRMS (APCI) calcd for $C_{22}H_{17}O_4^-$ [M-H]$^-$: 345.1132; found: 345.1130.

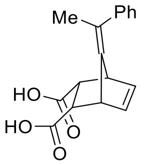

3i, (1R,2S,3R,4S,E)-7-(1-phenylethylidene)bicyclo[2.2.1]hept-5-ene-2,3-dicarboxylic acid

Yield: 7%.

Physical state: yellow solid.

^{1}H NMR (500 MHz, Acetone-*d*$_6$) δ 7.33 (t, *J* = 7.6 Hz, 2H), 7.25 – 7.18 (m, 3H), 6.41 – 6.35 (m, 1H), 6.31 – 6.25 (m, 1H), 3.80 – 3.74 (m, 1H), 3.48 – 3.44 (m, 1H), 3.44 – 3.35 (m, 2H), 1.95 (s, 3H).

^{13}C NMR (126 MHz, Acetone-*d*$_6$) δ 173.0, 172.8, 149.0, 142.9, 135.6, 135.3, 128.9, 128.5, 127.3, 115.6, 48.4, 48.1, 48.0, 47.4, 19.2.

HRMS (APCI) calcd for $C_{17}H_{15}O_4^-$ [M-H]$^-$: 283.0976; found: 283.0974.

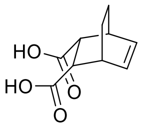

3j, (1R,2S,3R,4S)-bicyclo[2.2.2]oct-5-ene-2,3-dicarboxylic acid

Yield: 52%.

Physical state: white solid.

^{1}H NMR (400 MHz, Acetone-*d*$_6$) δ 6.21 (dd, *J* = 4.7, 3.1 Hz, 2H), 3.05 (s, 2H), 2.86 (s, 2H), 1.70 – 1.57 (m, 2H), 1.32 – 1.18 (m, 2H).

^{13}C NMR (101 MHz, Acetone-*d*$_6$) δ 174.3, 132.9, 47.8, 33.5, 25.2.

HRMS (APCI) calcd for $C_{10}H_{11}O_4^-$ [M-H]$^-$: 195.0663; found: 195.0663.

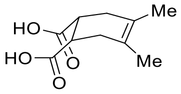

3k, (1R,2S)-4,5-dimethylcyclohex-4-ene-1,2-dicarboxylic acid

Yield: 70%.

Physical state: white solid.

^{1}H NMR (500 MHz, DMSO-*d*$_6$) δ 12.08 (br, 2H), 2.82 (t, *J* = 5.9 Hz, 2H), 2.35 – 2.25 (m, 2H), 2.22 – 2.08 (m, 2H), 1.55 (s, 6H).

^{13}C NMR (126 MHz, DMSO-*d*$_6$) δ 175.0, 124.0, 39.9, 32.3, 19.2.

HRMS (APCI) calcd for $C_{10}H_{13}O_4^-$ [M-H]$^-$: 197.0819; found: 197.0817.

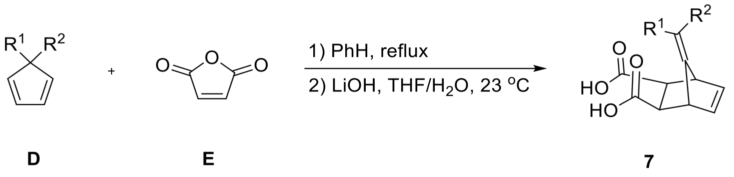

General procedure for the preparation of diacid substrates 7

To a solution of **E** (1.0 equiv) in benzene (1.0 M) was added **D** (1.0 equiv). The mixture was refluxed for 1 hour. The reaction mixture was concentrated under reduced pressure and the crude product was used in the next step directly.

To a solution of the above crude product in THF/H_2O (0.1 M, 1:1 v/v) was added LiOH (4.0 equiv). The mixture was stirred at room temperature for 1 hour. After removal of the THF under reduced pressure, the aqueous phase was washed with dichloromethane. 2 N HCl was then added until the pH of the aqueous phase was ca. 2. The aqueous layer was then extracted twice with diethyl ether. The combined organic phase was dried over anhydrous Na_2SO_4, filtered, and concentrated under reduced pressure to give diacid **7**.

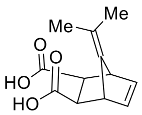

7a, (1R,2R,3S,4S)-7-(propan-2-ylidene)bicyclo[2.2.1]hept-5-ene-2,3-dicarboxylic acid

Yield: 38%.

Physical state: white solid.

^{1}H NMR (500 MHz, Acetone-*d*$_6$) δ 6.36 (t, *J* = 2.0 Hz, 2H), 3.55 (t, *J* = 2.0 Hz, 2H), 2.63 (s, 2H), 1.57 (s, 6H).

^{13}C NMR (126 MHz, Acetone-*d*$_6$) δ 174.3, 145.1, 138.5, 112.6, 47.8, 46.2, 20.3.

HRMS (APCI) calcd for $C_{12}H_{13}O_4^-$ [M-H]$^+$: 221.0819; found: 221.0819.

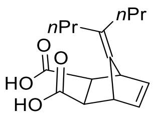

7b, (1R,2R,3S,4S)-7-(heptan-4-ylidene)bicyclo[2.2.1]hept-5-ene-2,3-dicarboxylic acid

Yield: 40%.

Physical state: yellow oil.

^{1}H NMR (500 MHz, Acetone-*d*$_6$) δ 6.39 (t, *J* = 2.0 Hz, 2H), 3.57 (t, *J* = 2.1 Hz, 2H), 2.64 (s, 2H), 2.05 – 1.99 (m, 2H), 1.96 – 1.89 (m, 2H), 1.45 – 1.36 (m, 2H), 1.35 – 1.26 (m, 2H), 0.84 (t, *J* = 7.4 Hz, 6H).

^{13}C NMR (126 MHz, Acetone-*d*$_6$) δ 174.1, 146.4, 139.0, 120.7, 47.7, 46.2, 34.3, 22.2, 14.5.

HRMS (APCI) calcd for $C_{16}H_{21}O_4^-$ [M-H]$^-$: 277.1445; found: 277.1443.

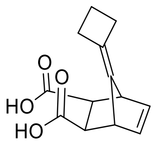

7c, (1R,3S,4S)-7-cyclobutylidenebicyclo[2.2.1]hept-5-ene-2,3-dicarboxylic acid

Yield: 26%.

Physical state: pale yellow solid.

^{1}H NMR (500 MHz, Acetone-*d*$_6$) δ 7.24 (t, *J* = 2.0 Hz, 2H), 4.21 (t, *J* = 2.0 Hz, 2H), 3.60 – 3.54 (m, 2H), 3.52 (s, 2H), 3.47 – 3.40 (m, 2H), 2.77 – 2.70 (m, 2H).

^{13}C NMR (126 MHz, Acetone-*d*$_6$) δ 174.3, 141.2, 138.5, 120.2, 48.0, 46.1, 29.5, 17.6.

HRMS (ESI) calcd for $C_{13}H_{13}O_4^-$ [M-H]$^-$: 233.0819; found: 233.0816.

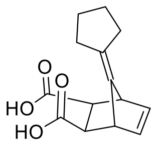

7d, (1R,2R,3S,4S)-7-cyclopentylidenebicyclo[2.2.1]hept-5-ene-2,3-dicarboxylic acid

Yield: 30%.

Physical state: white solid.

^{1}H NMR (500 MHz, Acetone-*d*$_6$) δ 6.37 (t, *J* = 2.0 Hz, 2H), 3.40 (t, *J* = 2.0 Hz, 2H), 2.63 (s, 2H), 2.40 – 2.29 (m, 2H), 2.08 – 2.07 (m, 2H), 1.58 – 1.51 (m, 4H).

^{13}C NMR (126 MHz, Acetone-*d*$_6$) δ 174.1, 141.1, 138.5, 123.6, 47.9, 47.3, 30.3, 27.1.

HRMS (APCI) calcd for $C_{14}H_{15}O_4^-$ [M-H]$^-$: 247.0976; found: 247.0977.

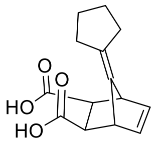

7e, (1R,2R,3S,4S)-7-cyclohexylidenebicyclo[2.2.1]hept-5-ene-2,3-dicarboxylic acid

Yield: 63%.

Physical state: white solid.

^{1}H NMR (500 MHz, Acetone-*d*$_6$) δ 6.36 (s, 2H), 3.57 (s, 2H), 2.64 (s, 2H), 2.30 – 2.21 (m, 2H), 1.93 – 1.81 (m, 2H), 1.56 – 1.35 (m, 6H).

^{13}C NMR (126 MHz, Acetone-*d*$_6$) δ 174.1, 142.1, 138.6, 120.5, 47.8, 45.7, 31.7, 27.9, 27.5.

HRMS (APCI) calcd for $C_{15}H_{17}O_4^-$ [M-H]$^-$: 261.1132; found: 261.1131.

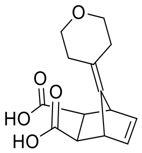

7f, (1R,3S,4S)-7-(tetrahydro-4H-pyran-4-ylidene)bicyclo[2.2.1]hept-5-ene-2,3-dicarboxylic acid

Yield: 22%.

Physical state: white solid.

^{1}H NMR (500 MHz, Acetone-*d$_6$*) δ 6.36 (t, *J* = 2.0 Hz, 2H), 3.63 (dt, *J* = 10.6, 4.3 Hz, 2H), 3.56 (t, *J* = 2.1 Hz, 2H), 3.47 (td, *J* = 10.2, 3.1 Hz, 2H), 2.66 (s, 2H), 2.26 (dt, *J* = 13.4, 3.5 Hz, 2H), 2.08 – 2.06 (m, 2H).

^{13}C NMR (126 MHz, Acetone-*d$_6$*) δ 174.4, 143.8, 138.4, 116.1, 68.9, 47.9, 45.7, 32.3.

HRMS (ESI) calcd for $C_{14}H_{15}O_5^-$ [M-H]$^-$: 263.0925; found: 263.0921.

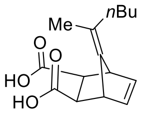

7g, (1R,3S,4S,Z)-7-(hexan-2-ylidene)bicyclo[2.2.1]hept-5-ene-2,3-dicarboxylic acid

Yield: 8%.

Physical state: pale yellow foam.

^{1}H NMR (500 MHz, Acetone-*d$_6$*) δ 6.37 (t, *J* = 2.0 Hz, 2H), 3.57 (q, *J* = 1.9 Hz, 1H), 3.53 (q, *J* = 1.8 Hz, 1H), 2.62 (s, 2H), 2.17 – 2.09 (m, 1H), 1.91 – 1.83 (m, 1H), 1.42 – 1.34 (m, 1H), 1.32 – 1.21 (m, 3H), 0.86 (t, *J* = 7.2 Hz, 3H).

^{13}C NMR (126 MHz, Acetone-*d$_6$*) δ 174.4, 174.3, 145.7, 138.8, 116.5, 48.1, 47.6, 46.4, 46.1, 34.5, 30.9, 23.2, 17.9, 14.4.

HRMS (ESI) calcd for $C_{15}H_{20}O_4Na^+$ [M+Na]$^+$: 287.1254; found: 287.1252.

4. Desymmetrizing asymmetric halolactonization of *meso*-diacids 3 and 7

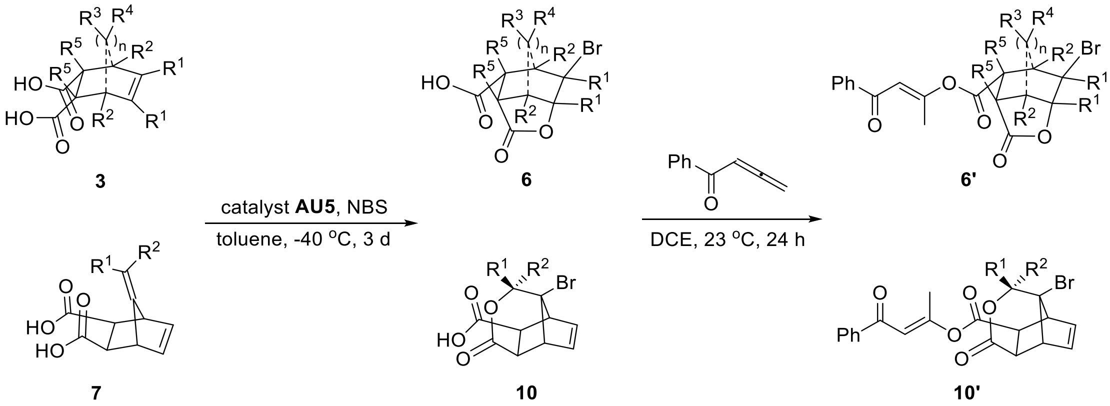

General procedure for the halocyclization of *meso*-diacid 3 and 7

To a solution of **3** or **7** (0.1 mmol, 1 equiv) in toluene (0.025 M) was added **AU5** (0.01 or 0.03 mmol) and 3Å MS (15 or 30 mg) in a resealable tube. The mixture was stirred at -40 °C for 30 min and then NBS (0.105 mmol, 1.05 equiv) was added in the absence of light. After completion of the reaction, 1,3,5-trimethoxybenzene (0.105 mmol, 1.05 equiv) was added to consume unreacted NBS. The reaction mixture was stirred at -40 °C for 1 h. The solution was filtered through a thin plug of celite and the filtrate was concentrated under reduced pressure to give the product **6** or **10**.

Compounds **6** and **10** have very weak UV absorption, making measurement of enantioselectivity with HPLC non-trivial. To facilitate the e.r. measurement, compounds **6** and **10** were converted into esters **6'** and **10'** that have strong UV absorption under mild and racemization-free conditions.[10] To a solution of **6** or **10** (1.0 equiv) in DCE (0.1 M) was added 1-phenylbuta-2,3-dien-1-one (1.5 equiv). The mixture was stirred at room temperature for 24 h. Upon consumption of the starting material (TLC analysis), the mixture was concentrated under reduced pressure. The residue was purified by flash column chromatography (Hexanes: EtOAc = 5:1) to yield the product **6'** or **10'**.

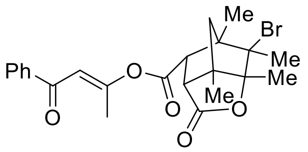

6a’, (E)-4-oxo-4-phenylbut-2-en-2-yl 6-bromo-3a,5,6,6a-tetramethyl-2-oxohexahydro-2H-3,5-methanocyclopenta[b]furan-7-carboxylate

Yield: 85%.

Physical state: orange oil.

$[\alpha]_D^{25}$ 11.4 (c 0.35, $CHCl_3$, 95:5 er).

HPLC (Daicel Chiralpak IC, *i*-PrOH/*n*-hexane = 40/60, 0.8 mL/min, 254 nm) t1= 17.0 min (major), t2 = 22.9 min (minor).

^{1}H NMR (500 MHz, Acetone-*d*$_6$) δ 7.94 (d, *J* = 7.4 Hz, 2H), 7.63 (t, *J* = 7.4 Hz, 1H), 7.54 (t, *J* = 7.7 Hz, 2H), 6.94 (s, 1H), 3.73 (d, *J* = 10.6 Hz, 1H), 2.86 (d, *J* = 10.6 Hz, 1H), 2.36 (s, 3H), 2.29 (d, *J* = 11.4 Hz, 1H), 1.84 (s, 3H), 1.81 (d, *J* = 11.4 Hz, 1H), 1.64 (s, 3H), 1.61 (s, 3H), 1.27 (s, 3H).

^{13}C NMR (126 MHz, Acetone-*d*$_6$) δ 190.2, 175.1, 168.6, 164.1, 139.4, 133.8, 129.6, 128.8, 114.6, 94.7, 84.9, 56.7, 56.3, 52.9, 50.5, 49.7, 25.2, 23.6, 19.2, 18.8, 14.7.

HRMS (APCI) calcd for $C_{23}H_{26}BrO_5^+$ [M+H]$^+$: 461.0958 (Br^{79}), 463.0941 (Br^{81}); found: 461.0957 (Br^{79}), 463.0941 (Br^{81}).

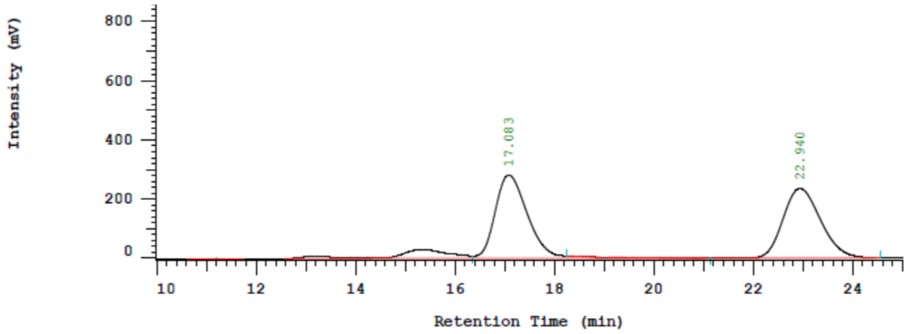

No.	RT	Area	Conc 1	BC
1	17.083	13094839	51.459	VV
2	22.940	12352092	48.541	BB
		25446931	100.000	

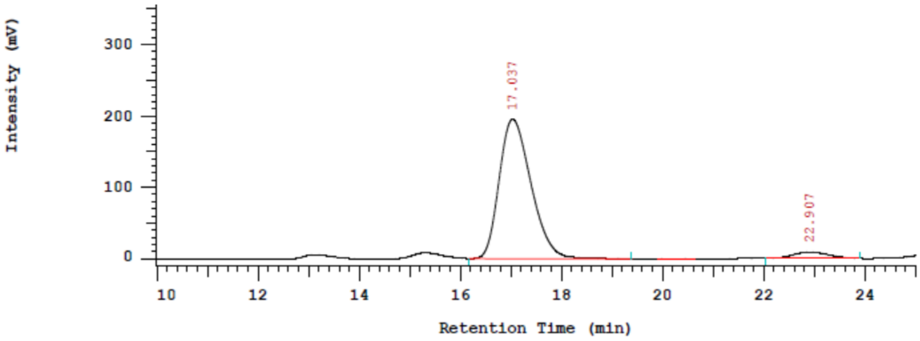

No.	RT	Area	Conc 1	BC
1	17.037	8537720	95.198	MC
2	22.907	430683	4.802	MC
		8968403	100.000	

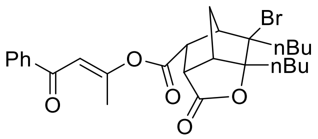

6b', (E)-4-oxo-4-phenylbut-2-en-2-yl 6-bromo-6,6a-dibutyl-2-oxohexahydro-2H-3,5-methanocyclopenta[b]furan-7-carboxylate

Yield: 90%.

Physical state: orange oil.

$[\alpha]_D^{25}$ 15.9 (c 0.27, $CHCl_3$, 95:5 er).

HPLC (Daicel Chiralpak IC, *i*-PrOH/*n*-hexane = 15/85, 0.6 mL/min, 254 nm) t1= 35.3 min (major), t2 = 40.9 min (minor).

^{1}H NMR (500 MHz, Acetone-*d*$_6$) δ 7.95 (d, *J* = 7.7 Hz, 2H), 7.64 (t, *J* = 7.3 Hz, 1H), 7.54 (t, *J* = 7.7 Hz, 2H), 6.96 (s, 1H), 3.68 (dd, *J* = 10.2, 3.0 Hz, 1H), 3.32 (d, *J* = 4.2 Hz, 1H), 3.28 (s, 1H), 3.02 (dd, *J* = 10.2, 5.0 Hz, 1H), 2.54 (d, *J* = 11.5 Hz, 1H), 2.38 (s, 3H), 2.02 – 1.84 (m, 4H), 1.69 – 1.62 (m, 2H), 1.54 – 1.45 (m, 1H), 1.42 – 1.27 (m, 6H), 0.93 (t, *J* = 7.0 Hz, 3H), 0.87 (t, *J* = 7.4 Hz, 3H).

^{13}C NMR (126 MHz, Acetone-*d*$_6$) δ 190.3, 175.0, 168.8, 164.2, 139.4, 133.9, 129.6, 128.8, 114.3, 93.9, 83.7, 54.9, 52.3, 49.6, 41.6, 39.2, 37.2, 30.1, 27.6, 23.5, 18.8, 14.3, 14.2.

HRMS (ESI) calcd for $C_{27}H_{33}BrO_5Na^+$ $[M+Na]^+$: 539.1404 (Br^{79}), 541.1383 (Br^{81}); found: 539.1410 (Br^{79}), 541.1379 (Br^{81}).

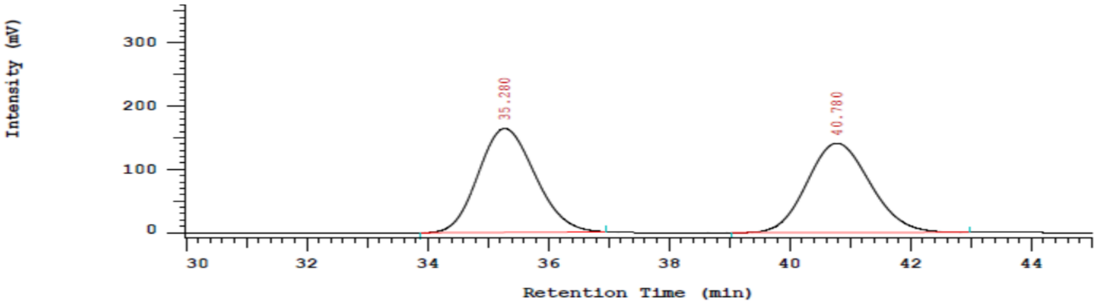

No.	RT	Area	Conc 1	BC
1	35.280	10716261	51.066	MC
2	40.780	10268812	48.934	MC
		20985073	100.000	

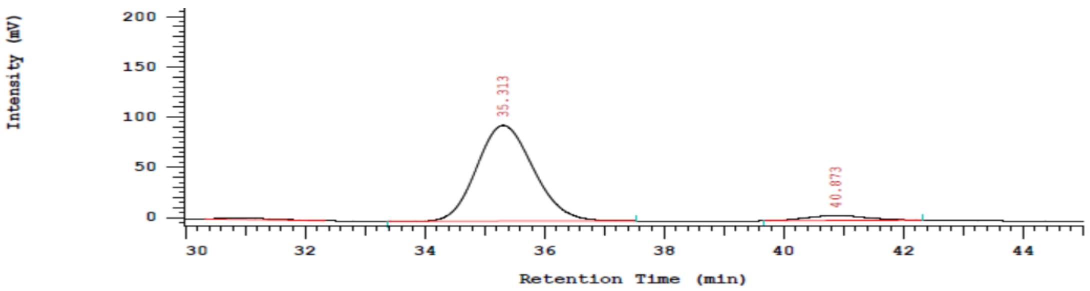

No.	RT	Area	Conc 1	BC
1	35.313	6255841	94.941	MC
2	40.873	333349	5.059	MC
		6589190	100.000	

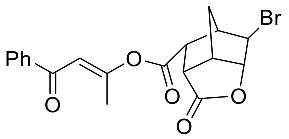

6c', (E)-4-oxo-4-phenylbut-2-en-2-yl 6-bromo-2-oxohexahydro-2H-3,5-methanocyclopenta[b]furan-7-carboxylate

Yield: 97%.

Physical state: orange oil.

$[\alpha]_D^{25}$ -19.6 (c 0.28, $CHCl_3$, 90:10 er).

HPLC (Daicel Chiralpak IA, *i*-PrOH/*n*-hexane = 10/90, 0.8 mL/min, 254 nm) t1= 51.3 min (minor), t2 = 54.9 min (major).

^{1}H NMR (500 MHz, Acetone-*d*$_6$) δ 7.95 (d, *J* = 7.9 Hz, 2H), 7.63 (t, *J* = 7.3 Hz, 1H), 7.54 (t, *J* = 7.4 Hz, 2H), 6.97 (s, 1H), 5.06 (d, *J* = 5.1 Hz, 1H), 4.68 (s, 1H), 3.63 (dd, *J* = 11.0, 2.9 Hz, 1H), 3.55 (t, *J* = 4.8 Hz, 1H), 3.10 (dd, *J* = 11.1, 4.8 Hz, 1H), 3.00 (s, 1H), 2.39 – 2.33 (m, 4H), 2.00 (d, *J* = 11.6 Hz, 1H).

^{13}C NMR (126 MHz, Acetone-*d*$_6$) δ 190.2, 176.8, 169.6, 164.0, 139.4, 133.9, 129.6, 128.8, 114.9, 88.3, 50.9, 48.8, 48.7, 48.6, 41.6, 35.9, 18.8.

HRMS (APCI) calcd for $C_{19}H_{18}BrO_5^+$ $[M+H]^+$: 405.0332 (Br^{79}), 407.0314 (Br^{81}); found: 405.0335 (Br^{79}), 407.0314 (Br^{81}).

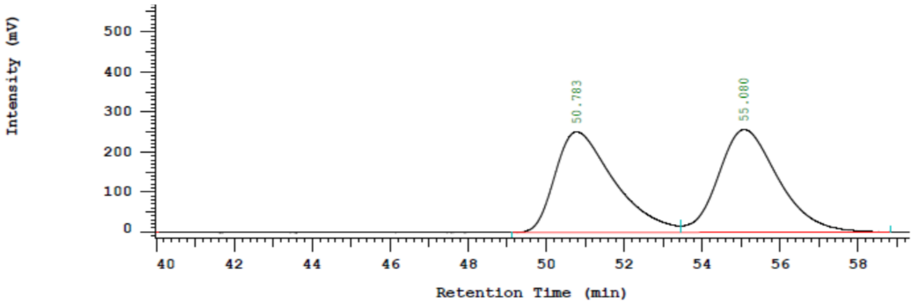

No.	RT	Area	Conc 1	BC
1	50.783	26413859	49.133	BV
2	55.080	27346588	50.867	VB
		53760447	100.000	

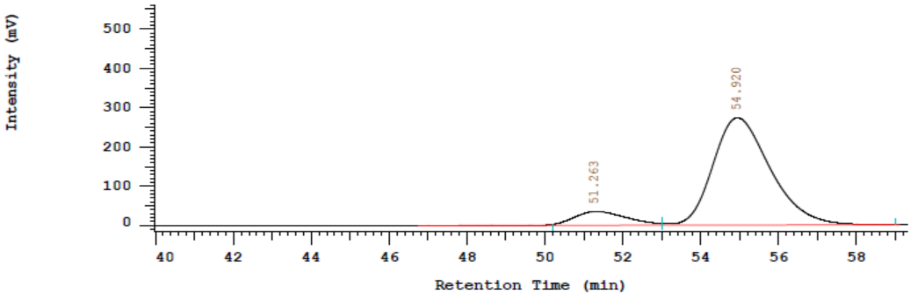

No.	RT	Area	Conc 1	BC
1	51.263	3277111	10.492	MC
2	54.920	27956080	89.508	MC
		31233191	100.000	

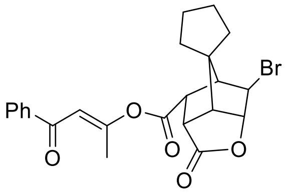

6d’, (E)-4-oxo-4-phenylbut-2-en-2-yl 6'-bromo-2'-oxohexahydrospiro[cyclopentane-1,4'-[3,5]methanocyclopenta[b]furan]-7'-carboxylate

Yield: 86%.

Physical state: orange oil.

$[\alpha]_D^{25}$ 17.5 (c 0.2, $CHCl_3$, 95:5 er).

HPLC (Daicel Chiralpak IA, *i*-PrOH/*n*-hexane = 15/85, 0.6 mL/min, 254 nm) t1= 38.3 min (major), t2 = 44.8 min (minor).

^{1}H NMR (500 MHz, Acetone-*d*$_6$) δ 7.96 (d, *J* = 8.0 Hz, 2H), 7.64 (t, *J* = 7.2 Hz, 1H), 7.54 (t, *J* = 7.4 Hz, 2H), 6.97 (s, 1H), 5.20 (d, *J* = 4.9 Hz, 1H), 4.71 (s, 1H), 3.83 (dd, *J* = 10.7, 2.9 Hz, 1H), 3.35 (t, *J* = 4.9 Hz, 1H), 3.18 (dd, *J* = 10.8, 4.7 Hz, 1H), 2.73 (d, *J* = 1.8 Hz, 1H), 2.36 (s, 3H), 2.05 – 2.01 (m, 2H), 1.85 – 1.76 (m, 3H), 1.74 – 1.63 (m, 3H).

^{13}C NMR (126 MHz, Acetone-*d*$_6$) δ 190.2, 176.6, 169.5, 164.0, 139.4, 133.9, 129.6, 128.8, 114.9, 89.6, 60.5, 57.1, 55.2, 49.8, 49.5, 41.8, 33.7, 32.5, 26.4, 24.3, 18.8.

HRMS (APCI) calcd for $C_{23}H_{24}BrO_5^+$ [M+H]$^+$: 459.0802 (Br^{79}), 461.0784 (Br^{81}); found: 459.0806 (Br^{79}), 461.0783 (Br^{81}).

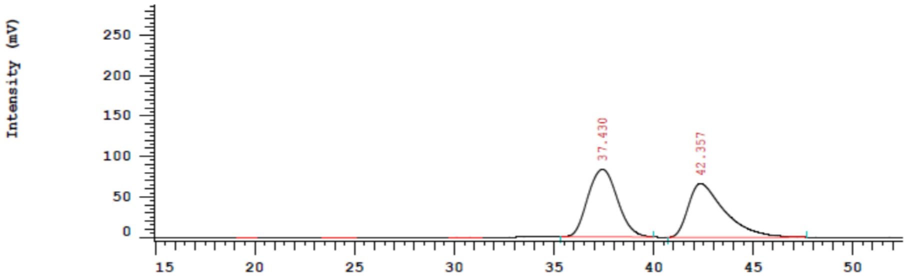

No.	RT	Area	Conc 1	BC
1	37.430	8564238	50.540	MC
2	42.357	8381363	49.460	MC
		16945601	100.000	

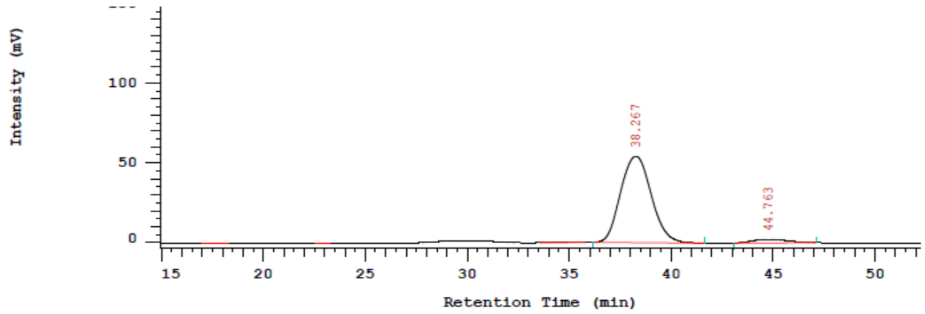

No.	RT	Area	Conc 1	BC
1	38.267	5630408	95.303	MC
2	44.763	277506	4.697	MC
		5907914	100.000	

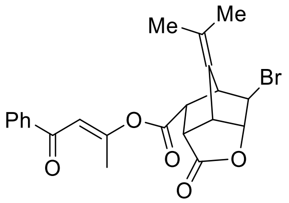

6e’, (E)-4-oxo-4-phenylbut-2-en-2-yl 6-bromo-2-oxo-4-(propan-2-ylidene)hexahydro-2H-3,5-methanocyclopenta[b]furan-7-carboxylate

Yield: 91%.

Physical state: orange oil.

$[\alpha]_D^{25}$ 26.7 (c 0.35, $CHCl_3$, 89:11 er).

HPLC (Daicel Chiralpak IC, *i*-PrOH/*n*-hexane = 40/60, 0.8 mL/min, 254 nm) t1= 19.0 min (minor), t2 = 53.5 min (major).

^{1}H NMR (400 MHz, Acetone-*d*$_6$) δ 7.96 (d, *J* = 7.4 Hz, 2H), 7.64 (t, *J* = 7.3 Hz, 1H), 7.54 (t, *J* = 7.7 Hz, 2H), 4.99 (d, *J* = 5.0 Hz, 1H), 4.70 (s, 1H), 4.11 (t, *J* = 5.0 Hz, 1H), 3.55 (d, *J* = 3.4 Hz, 1H), 3.47 (dd, *J* = 10.9, 3.4 Hz, 1H), 3.14 (dd, *J* = 10.9, 4.8 Hz, 1H), 2.36 (s, 3H), 1.83 (s, 3H), 1.80 (s, 3H).

^{13}C NMR (101 MHz, Acetone-*d*$_6$) δ 190.2, 176.7, 169.6, 164.1, 139.4, 134.0, 133.9, 129.6, 128.8, 126.7, 114.9, 87.0, 50.6, 48.9, 48.3, 48.0, 42.0, 20.9, 18.8.

HRMS (APCI) calcd for $C_{22}H_{22}BrO_5^+$ $[M+H]^+$: 445.0645 (Br^{79}), 447.0628 (Br^{81}); found: 445.0646 (Br^{79}), 447.0626 (Br^{81}).

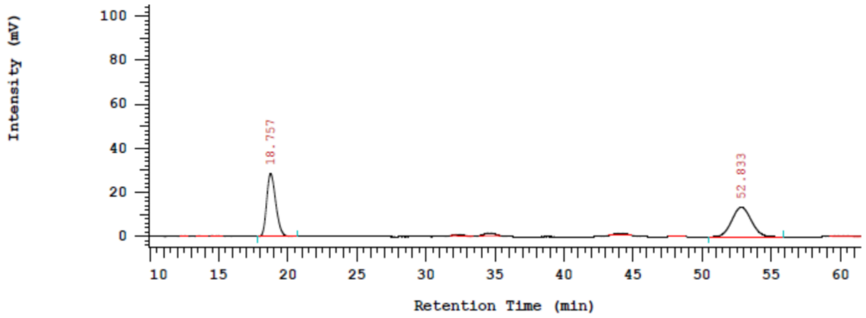

No.	RT	Area	Conc 1	BC
1	18.757	1287570	49.568	MC
2	52.833	1310033	50.432	MC
		2597603	100.000	

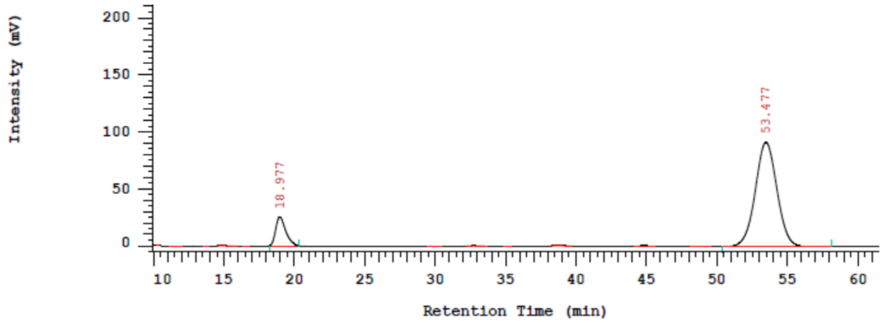

No.	RT	Area	Conc 1	BC
1	18.977	1230340	11.291	MC
2	53.477	9666574	88.709	MC
		10896914	100.000	

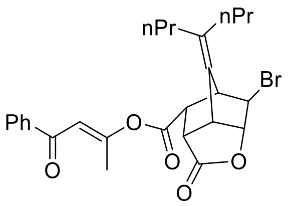

6f', (E)-4-oxo-4-phenylbut-2-en-2-yl 6-bromo-4-(heptan-4-ylidene)-2-oxohexahydro-2H-3,5-methanocyclopenta[b]furan-7-carboxylate

Yield: 86%.

Physical state: orange oil.

$[\alpha]_D^{25}$ 46.7 (c 0.24, $CHCl_3$, 92:8 er).

HPLC (Daicel Chiralpak IC, *i*-PrOH/*n*-hexane = 40/60, 0.8 mL/min, 254 nm) t1= 16.0 min (minor), t2 = 45.4 min (major).

^{1}H NMR (500 MHz, Acetone-d_6) δ 7.95 (d, *J* = 7.8 Hz, 2H), 7.63 (t, *J* = 7.4 Hz, 1H), 7.54 (t, *J* = 7.6 Hz, 2H), 6.96 (s, 1H), 4.98 (d, *J* = 5.2 Hz, 1H), 4.72 (s, 1H), 4.11 (t, *J* = 5.2 Hz, 1H), 3.56 (d, *J* = 3.3 Hz, 1H), 3.51 – 3.42 (m, 1H), 3.16 (dd, *J* = 11.0, 4.8 Hz, 1H), 2.35 (s, 3H), 2.25 – 2.11 (m, 4H), 1.58 – 1.40 (m, 4H), 0.93 (q, *J* = 7.2 Hz, 6H).

^{13}C NMR (126 MHz, Acetone-d_6) δ 190.2, 176.7, 169.5, 164.1, 139.4, 135.3, 135.0, 133.9, 129.6, 128.8, 114.9, 87.1, 50.4, 48.7, 48.3, 48.1, 42.2, 35.0, 34.5, 22.6, 22.4, 18.8, 14.6, 14.0.

HRMS (APCI) calcd for $C_{26}H_{30}BrO_5^+$ $[M+H]^+$: 501.1271 (Br^{79}), 503.1255 (Br^{81}); found: 501.1277 (Br^{79}), 503.1251 (Br^{81}).

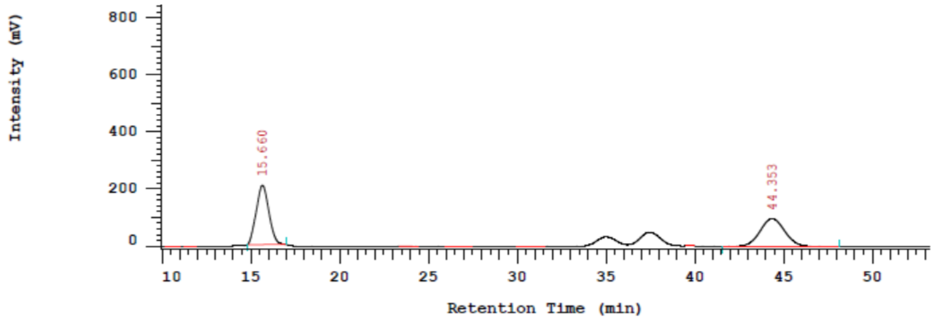

No.	RT	Area	Conc 1	BC
1	15.660	10195792	52.113	MC
2	44.353	9368863	47.887	MC
		19564655	100.000	

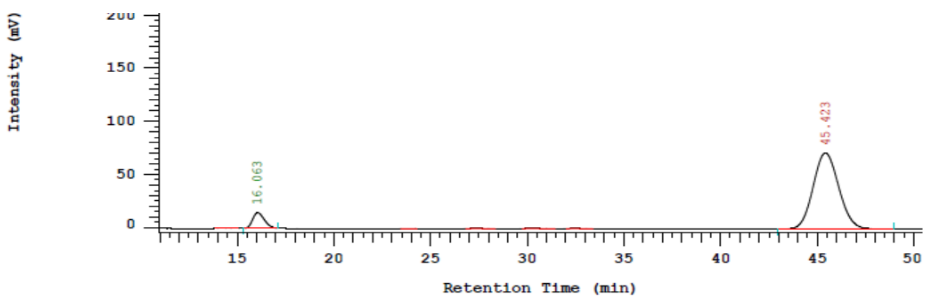

No.	RT	Area	Conc 1	BC
1	16.063	608213	8.496	BB
2	45.423	6550346	91.504	MC
		7158559	100.000	

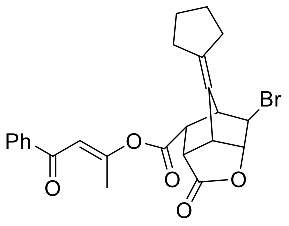

6g’, (E)-4-oxo-4-phenylbut-2-en-2-yl 6-bromo-4-cyclopentylidene-2-oxohexahydro-2H-3,5-methanocyclopenta[b]furan-7-carboxylate

Yield: 84%.

Physical state: orange oil.

$[\alpha]_D^{25}$ 48.0 (c 0.125, $CHCl_3$, 90:10 er).

HPLC (Daicel Chiralpak IC, *i*-PrOH/*n*-hexane = 60/40, 0.8 mL/min, 254 nm) t1= 13.6 min (minor), t2 = 38.1 min (major).

^{1}H NMR (500 MHz, Acetone-*d*$_6$) δ 7.96 (d, *J* = 7.7 Hz, 2H), 7.64 (t, *J* = 7.3 Hz, 1H), 7.54 (t, *J* = 7.7 Hz, 2H), 6.96 (s, 1H), 5.01 (d, *J* = 5.2 Hz, 1H), 4.70 (s, 1H), 3.95 (t, *J* = 5.0 Hz, 1H), 3.50 (dd, *J* = 11.0, 3.5 Hz, 1H), 3.39 (d, *J* = 3.3 Hz, 1H), 3.15 (dd, *J* = 10.9, 4.8 Hz, 1H), 2.45 – 2.32 (m, 7H), 1.74 – 1.66 (m, 4H).

^{13}C NMR (126 MHz, Acetone-*d*$_6$) δ 190.2, 176.7, 169.6, 164.1, 139.4, 137.5, 133.9, 130.1, 129.6, 128.8, 115.0, 87.1, 50.7, 50.1, 49.3, 48.2, 42.2, 31.2, 31.1, 27.2, 27.0, 18.8.

HRMS (ESI) calcd for $C_{24}H_{23}BrO_5Na^+$ $[M+Na]^+$: 493.0621 (Br^{79}), 495.0601 (Br^{81}); found: 493.0624 (Br^{79}), 495.0596 (Br^{81}).

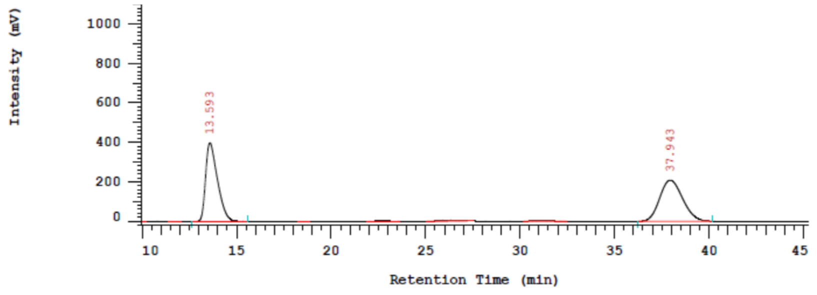

No.	RT	Area	Conc 1	BC
1	13.593	17100064	49.434	MC
2	37.943	17491850	50.566	MC
		34591914	100.000	

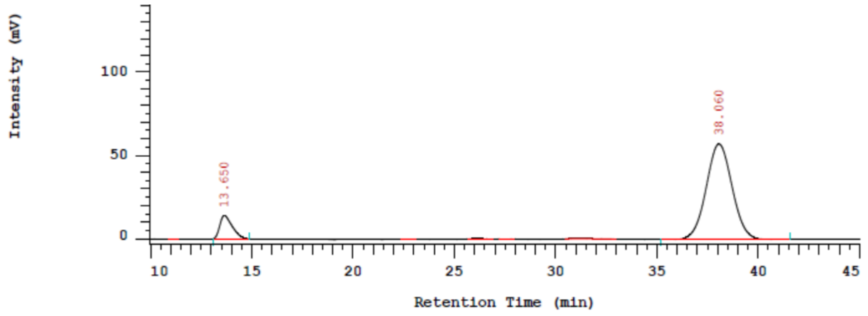

No.	RT	Area	Conc 1	BC
1	13.650	590167	10.439	MC
2	38.060	5063240	89.561	MC
		5653407	100.000	

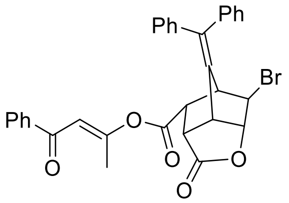

6h', (E)-4-oxo-4-phenylbut-2-en-2-yl 6-bromo-4-(diphenylmethylene)-2-oxohexahydro-2H-3,5-methanocyclopenta[b]furan-7-carboxylate

Yield: 79%.

Physical state: orange oil.

$[\alpha]_D^{25}$ 9.7 (c 0.1, $CHCl_3$, 87:13 er).

HPLC (Daicel Chiralpak IC, *i*-PrOH/*n*-hexane = 60/40, 0.8 mL/min, 254 nm) t1= 12.7 min (minor), t2 = 27.4 min (major).

^{1}H NMR (400 MHz, Acetone-*d*$_6$) δ 7.95 (d, *J* = 7.5 Hz, 2H), 7.63 (t, *J* = 7.4 Hz, 1H), 7.54 (t, *J* = 7.6 Hz, 2H), 7.44 – 7.27 (m, 8H), 6.98 (s, 1H), 5.27 (d, *J* = 5.2 Hz, 1H), 4.84 (s, 1H), 4.06 (t, *J* = 4.6 Hz, 1H), 3.90 (dd, *J* = 10.9, 3.5 Hz, 1H), 3.46 (d, *J* = 3.4 Hz, 1H), 3.40 (dd, *J* = 10.9, 4.8 Hz, 1H), 2.35 (s, 3H).

^{13}C NMR (126 MHz, Acetone-*d*$_6$) δ 190.2, 176.3, 169.3, 164.0, 141.6, 141.5, 139.4, 139.0, 137.8, 133.9, 130.2, 129.6, 129.2, 129.0, 128.8, 128.6, 128.4, 115.0, 86.7, 50.6, 50.4, 50.1, 47.2, 42.7, 18.8.

HRMS (ESI) calcd for $C_{32}H_{25}BrO_5Na^+$ $[M+Na]^+$: 591.0778 (Br^{79}), 593.0757 (Br^{81}); found: 591.0782 (Br^{79}), 593.0760 (Br^{81}).

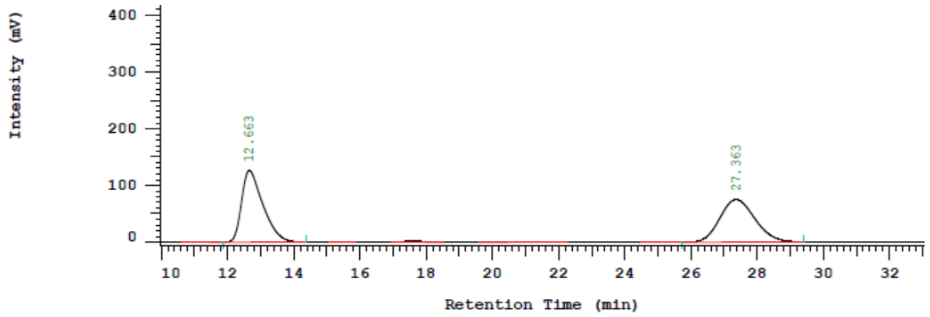

No.	RT	Area	Conc 1	BC
1	12.663	5609464	50.375	BB
2	27.363	5526052	49.625	BB
		11135516	100.000	

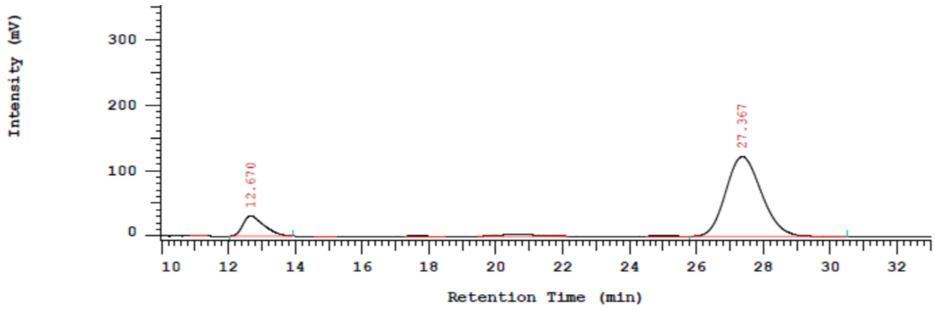

No.	RT	Area	Conc 1	BC
1	12.670	1354559	13.066	MC
2	27.367	9012372	86.934	MC
		10366931	100.000	

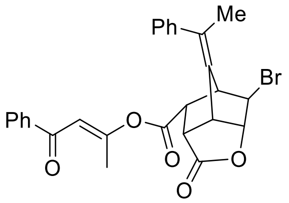

6i', (E)-4-oxo-4-phenylbut-2-en-2-yl (E)-6-bromo-2-oxo-4-(1-phenylethylidene)hexahydro-2H-3,5-methanocyclopenta[b]furan-7-carboxylate

Yield: 80%.

Physical state: orange oil.

$[\alpha]_D^{25}$ 33.0 (c 0.1, $CHCl_3$, 86:14 er). 1.3 : 1 *dr*.

HPLC (Daicel Chiralpak IC, *i*-PrOH/*n*-hexane = 30/70, 0.8 mL/min, 254 nm) t1= 27.1 min (minor), t2 = 73.2 min (major).

^{1}H NMR (500 MHz, Acetone-*d*$_6$) δ 7.96 (d, *J* = 7.7 Hz, 2H), 7.64 (t, *J* = 7.3 Hz, 1H), 7.55 (t, *J* = 7.6 Hz, 2H), 7.44 – 7.29 (m, 5H), 6.98 (s, 1H), 5.08 (d, *J* = 5.3 Hz, 1H), 4.81 (s, 1H), 3.88 (t, *J* = 5.1 Hz, 1H), 3.75 (d, *J* = 3.3 Hz, 1H), 3.69 (dd, *J* = 10.9, 3.5 Hz, 1H), 3.27 (dd, *J* = 10.9, 4.9 Hz, 1H), 2.38 (s, 3H), 2.18 (s, 3H).

^{13}C NMR (126 MHz, Acetone-*d*$_6$) δ 190.2, 176.5, 169.5, 164.0, 142.4, 139.4, 137.0, 133.9, 129.6, 129.6, 129.2, 128.8, 128.6, 128.2, 115.0, 86.8, 50.5, 49.5, 49.4, 47.7, 42.2, 20.8, 18.8.

HRMS (ESI) calcd for $C_{27}H_{23}BrO_5Na^+$ $[M+Na]^+$: 529.0621 (Br^{79}), 531.0601 (Br^{81}); found: 529.0630 (Br^{79}), 531.0600 (Br^{81}).

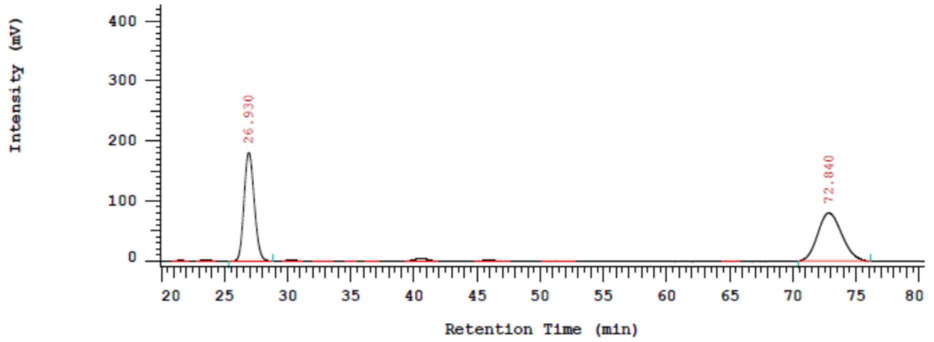

No.	RT	Area	Conc 1	BC
1	26.930	10003291	48.738	MC
2	72.840	10521386	51.262	MC
		20524677	100.000	

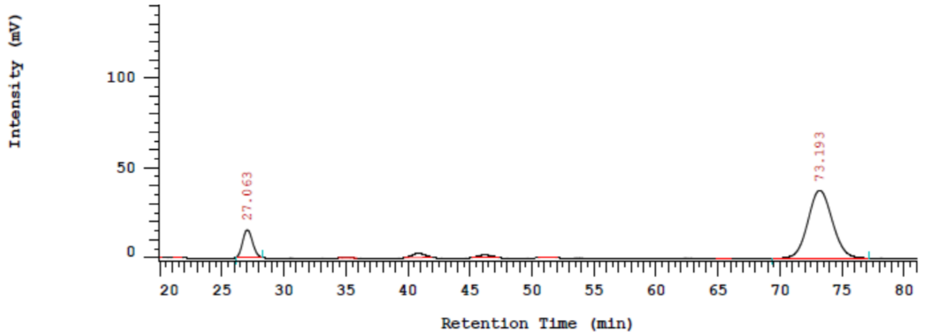

No.	RT	Area	Conc 1	BC
1	27.063	837173	14.159	MC
2	73.193	5075440	85.841	MC
		5912613	100.000	

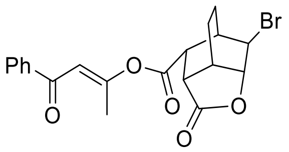

6j', (E)-4-oxo-4-phenylbut-2-en-2-yl 7-bromo-2-oxooctahydro-3,6-methanobenzofuran-8-carboxylate

Yield: 82%.

Physical state: orange oil.

$[\alpha]_D^{25}$ 1.0 (c 0.37, $CHCl_3$, 77:23 er).

HPLC (Daicel Chiralpak IC, *i*-PrOH/*n*-hexane = 30/70, 0.8 mL/min, 254 nm) t1= 33.6 min (minor), t2 = 44.0 min (major).

^{1}H NMR (500 MHz, Acetone-*d*$_6$) δ 7.96 (d, *J* = 7.2 Hz, 2H), 7.63 (t, *J* = 7.4 Hz, 1H), 7.54 (t, *J* = 7.6 Hz, 2H), 7.01 (s, 1H), 4.89 (d, *J* = 5.2 Hz, 1H), 4.78 (d, *J* = 3.8 Hz, 1H), 3.58 (d, *J* = 10.2 Hz, 1H), 3.13 (dd, *J* = 10.2, 4.8 Hz, 1H), 2.96 – 2.85 (m, 1H), 2.56 – 2.51 (m, 1H), 2.36 (s, 3H), 2.25 (ddt, *J* = 16.6, 12.7, 3.9 Hz, 1H), 2.04 – 2.02 (m, 1H), 1.92 – 1.84 (m, 1H), 1.74 – 1.65 (m, 1H).

^{13}C NMR (126 MHz, Acetone-*d*$_6$) δ 190.2, 176.8, 170.7, 164.1, 139.4, 133.9, 129.6, 128.8, 115.0, 84.7, 50.4, 43.7, 40.6, 37.8, 34.2, 21.9, 18.8, 15.1.

HRMS (ESI) calcd for $C_{20}H_{19}BrO_5Na^+$ $[M+Na]^+$: 441.0308 (Br^{79}), 443.0288 (Br^{81}); found: 441.0303 (Br^{79}), 443.0285 (Br^{81}).

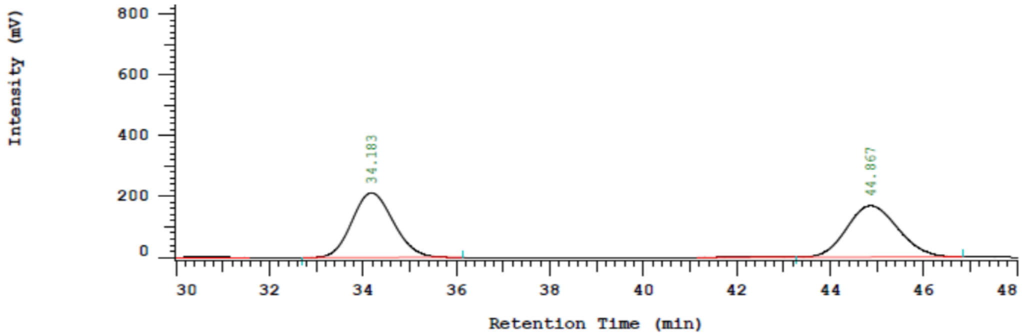

No.	RT	Area	Conc 1	BC
1	34.183	13088325	50.465	BB
2	44.867	12847118	49.535	VB
		25935443	100.000	

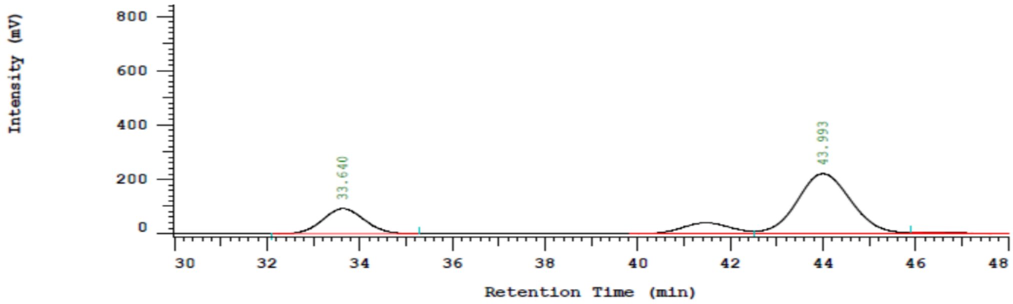

No.	RT	Area	Conc 1	BC
1	33.640	5848389	22.772	BB
2	43.993	19833771	77.228	VV
		25682160	100.000	

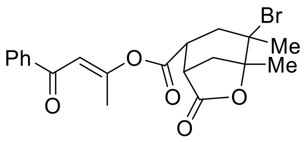

6k', (E)-4-oxo-4-phenylbut-2-en-2-yl 4-bromo-4,5-dimethyl-7-oxo-6-oxabicyclo[3.2.1]octane-2-carboxylate

Yield: 85%.

Physical state: orange oil.

$[\alpha]_D^{25}$ -28.0 (c 0.15, $CHCl_3$, 76:24 er).

HPLC (Daicel Chiralpak IC, *i*-PrOH/*n*-hexane = 30/70, 0.8 mL/min, 254 nm) t1= 26.0 min (minor), t2 = 34.7 min (major).

^{1}H NMR (500 MHz, Acetone-*d*$_6$) δ 7.96 (d, *J* = 7.6 Hz, 2H), 7.63 (t, *J* = 7.3 Hz, 1H), 7.54 (t, *J* = 7.6 Hz, 2H), 6.98 (s, 1H), 3.36 – 3.26 (m, 2H), 2.93 (d, *J* = 12.8 Hz, 1H), 2.62 (dd, *J* = 16.1, 5.1 Hz, 1H), 2.42 – 2.36 (m, 4H), 2.23 (dd, *J* = 16.1, 12.5 Hz, 1H), 1.88 (s, 3H), 1.62 (s, 3H).

^{13}C NMR (126 MHz, Acetone-*d*$_6$) δ 190.2, 175.0, 169.6, 164.5, 139.4, 133.9, 129.6, 128.8, 114.6, 88.5, 66.7, 43.7, 41.1, 41.0, 40.9, 20.4, 18.8.

HRMS (ESI) calcd for $C_{20}H_{21}BrO_5Na^+$ $[M+Na]^+$: 443.0465 (Br^{79}), 445.0444 (Br^{81}); found: 443.0465 (Br^{79}), 445.0445 (Br^{81}).

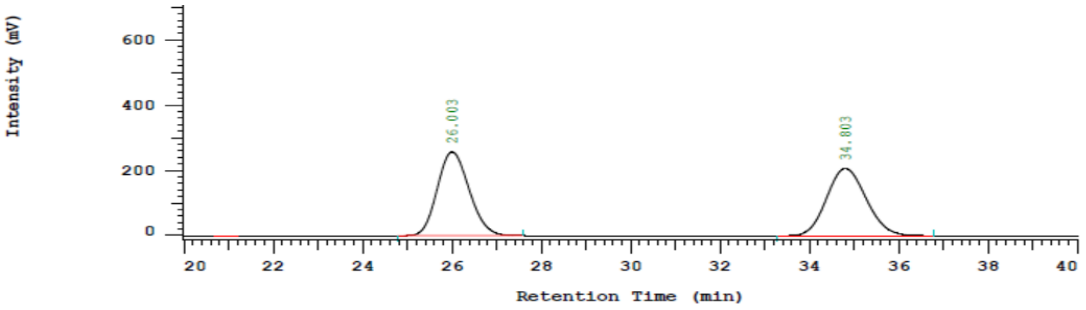

No.	RT	Area	Conc 1	BC
1	26.003	12693260	50.023	BB
2	34.803	12681579	49.977	BB
		25374839	100.000	

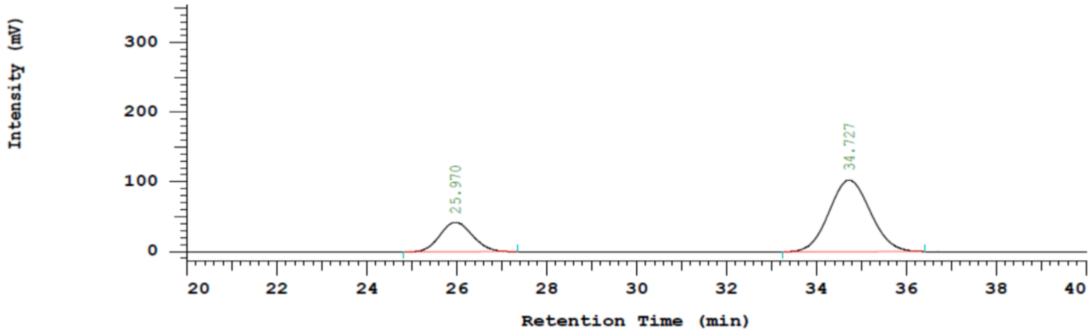

No.	RT	Area	Conc 1	BC
1	25.970	2115860	24.431	BB
2	34.727	6544586	75.569	BB
		8660446	100.000	

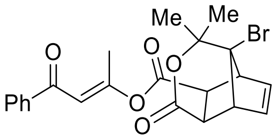

10a', (E)-4-oxo-4-phenylbut-2-en-2-yl 7a-bromo-1,1-dimethyl-3-oxo-1,3,4,4a,7,7a-hexahydro-4,7-methanocyclopenta[c]pyran-8-carboxylate

Yield: 96%.

Physical state: orange oil.

$[\alpha]_D^{25}$ 27.6 (c 0.34, $CHCl_3$, 93:7 er).

HPLC (Daicel Chiralpak IC, *i*-PrOH/*n*-hexane = 30/70, 0.8 mL/min, 254 nm) t1= 18.5 min (minor), t2 = 24.8 min (major).

^{1}H NMR (500 MHz, Chloroform-*d*) δ 7.91 (d, *J* = 7.7 Hz, 2H), 7.56 (t, *J* = 7.4 Hz, 1H), 7.47 (t, *J* = 7.6 Hz, 2H), 6.79 (dd, *J* = 5.9, 3.3 Hz, 1H), 6.75 (s, 1H), 6.21 (dd, *J* = 5.9, 3.0 Hz, 1H), 3.83 (s, 1H), 3.66 (s, 1H), 2.98 (d, *J* = 8.6 Hz, 1H), 2.80 (d, *J* = 8.6 Hz, 1H), 2.38 (s, 3H), 1.79 (s, 3H), 1.73 (s, 3H).

^{13}C NMR (126 MHz, Chloroform-*d*) δ 190.0, 170.9, 167.9, 163.2, 144.6, 138.4, 132.9, 132.7, 128.6, 128.1, 114.0, 87.1, 85.2, 48.7, 42.5, 30.5, 23.4, 18.4.

HRMS (APCI) calcd for $C_{22}H_{20}BrO_5^-$ [M-H]$^-$: 443.0500 (Br^{79}), 445.0482 (Br^{81}); found: 443.0505 (Br^{79}), 445.0483 (Br^{81}).

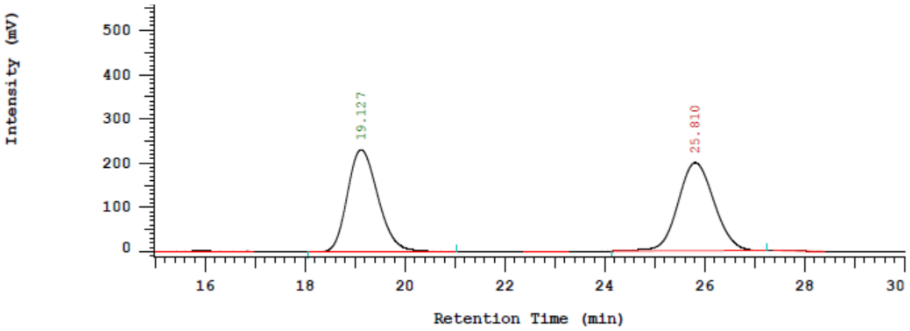

No.	RT	Area	Conc 1	BC
1	19.127	9806007	49.597	BB
2	25.810	9965361	50.403	MC
		19771368	100.000	

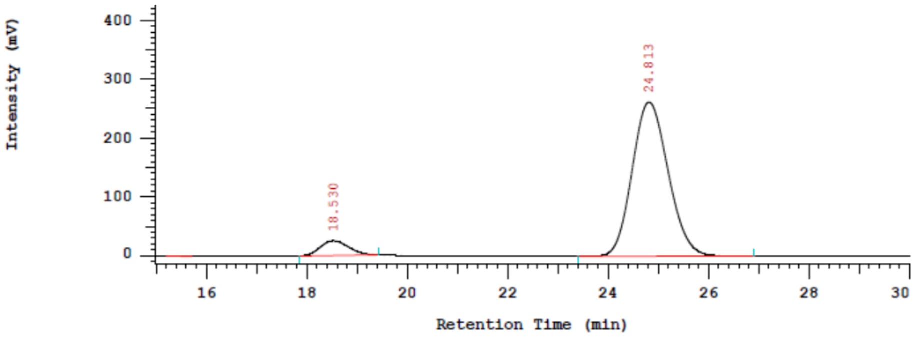

No.	RT	Area	Conc 1	BC
1	18.530	978922	7.003	MC
2	24.813	12999957	92.997	MC
		13978879	100.000	

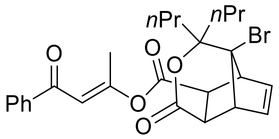

10b', (E)-4-oxo-4-phenylbut-2-en-2-yl 7a-bromo-3-oxo-1,1-dipropyl-1,3,4,4a,7,7a-hexahydro-4,7-methanocyclopenta[c]pyran-8-carboxylate

Yield: 94%.

Physical state: orange oil.

$[\alpha]_D^{25}$ 37.6 (c 0.42, $CHCl_3$, 96:4 er).

HPLC (Daicel Chiralpak IC, *i*-PrOH/*n*-hexane = 15/85, 0.6 mL/min, 254 nm) t1= 30.8 min (major), t2 = 34.7 min (minor).

^{1}H NMR (500 MHz, Acetone-*d*$_6$) δ 7.94 (d, *J* = 7.2 Hz, 2H), 7.64 (t, *J* = 7.4 Hz, 1H), 7.54 (t, *J* = 7.7 Hz, 2H), 6.87 (s, 1H), 6.84 (dd, *J* = 5.9, 3.3 Hz, 1H), 6.21 (dd, *J* = 5.8, 3.1 Hz, 1H), 3.86 (s, 1H), 3.78 (s, 1H), 3.05 (d, *J* = 8.6 Hz, 1H), 2.96 (dd, *J* = 8.6, 1.1 Hz, 1H), 2.33 (s, 3H), 2.22 – 2.15 (m, 1H), 2.13 – 2.09 (m, 2H), 2.04 – 1.99 (m, 1H), 1.72 – 1.62 (m, 1H), 1.60 – 1.51 (m, 1H), 1.49 – 1.37 (m, 2H), 0.91 (q, *J* = 7.2 Hz, 6H).

^{13}C NMR (126 MHz, Acetone-*d*$_6$) δ 190.2, 171.6, 168.9, 164.2, 145.0, 139.4, 133.9, 133.6, 129.6, 128.8, 114.6, 89.4, 87.8, 54.3, 53.8, 49.1, 44.8, 42.7, 38.7, 18.9, 18.5, 17.5, 14.7, 14.7.

HRMS (APCI) calcd for $C_{26}H_{28}BrO_5^-$ [M-H]$^-$: 499.1126 (Br^{79}), 501.1109 (Br^{81}); found: 499.1136 (Br^{79}), 501.1117 (Br^{81}).

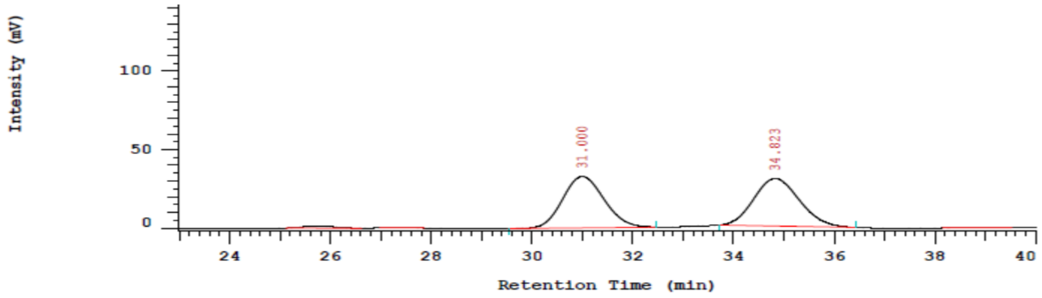

No.	RT	Area	Conc 1	BC
1	31.000	1856354	49.439	MC
2	34.823	1898474	50.561	MC
		3754828	100.000	

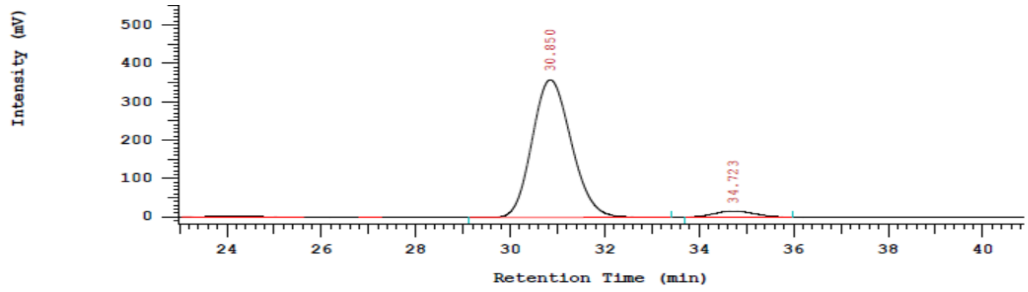

No.	RT	Area	Conc 1	BC
1	30.850	20632048	95.533	MC
2	34.723	964641	4.467	MC
		21596689	100.000	

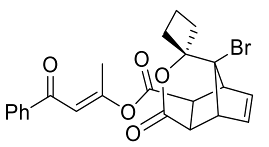

10c', (E)-4-oxo-4-phenylbut-2-en-2-yl 7a'-bromo-3'-oxo-4',4a',7',7a'-tetrahydro-3'H-spiro[cyclobutane-1,1'-[4,7]methanocyclopenta[c]pyran]-8'-carboxylate

Yield: 89%.

Physical state: orange oil.

$[\alpha]_D^{25}$ 50.0 (c 0.63, $CHCl_3$, 93:7 er).

HPLC (Daicel Chiralpak IC, *i*-PrOH/*n*-hexane = 20/80, 0.8 mL/min, 254 nm) t1= 27.7 min (minor), t2 = 32.3 min (major).

^{1}H NMR (500 MHz, Acetone-*d*$_6$) δ 7.97 – 7.93 (m, 2H), 7.67 – 7.61 (m, 1H), 7.57 – 7.53 (m, 2H), 6.89 (s, 1H), 6.77 – 6.73 (m, 1H), 6.29 – 6.24 (m, 1H), 3.74 – 3.69 (m, 1H), 3.66 – 3.61 (m, 1H), 3.14 – 3.07 (m, 1H), 2.99 – 2.94 (m, 2H), 2.80 – 2.73 (m, 1H), 2.54 – 2.46 (m, 1H), 2.39 – 2.30 (m, 4H), 1.97 – 1.87 (m, 2H).

^{13}C NMR (126 MHz, Acetone-*d*$_6$) δ 190.2, 170.2, 169.1, 164.3, 144.0, 139.4, 134.0, 133.9, 129.6, 128.8, 114.5, 89.1, 84.7, 54.2, 53.2, 48.1, 43.4, 38.8, 32.6, 18.5, 13.9.

HRMS (ESI) calcd for $C_{21}H_{23}BrO_5Na^+$ $[M+Na]^+$: 479.0465 (Br^{79}), 481.0444 (Br^{81}); found: 479.0459 (Br^{79}), 481.0436 (Br^{81}).

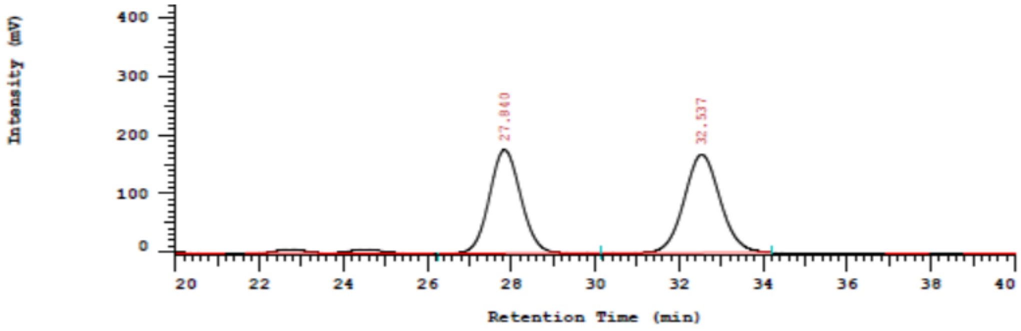

No.	RT	Area	Conc 1	BC
1	27.840	9112067	47.757	MC
2	32.537	9968104	52.243	MC
		19080171	100.000	

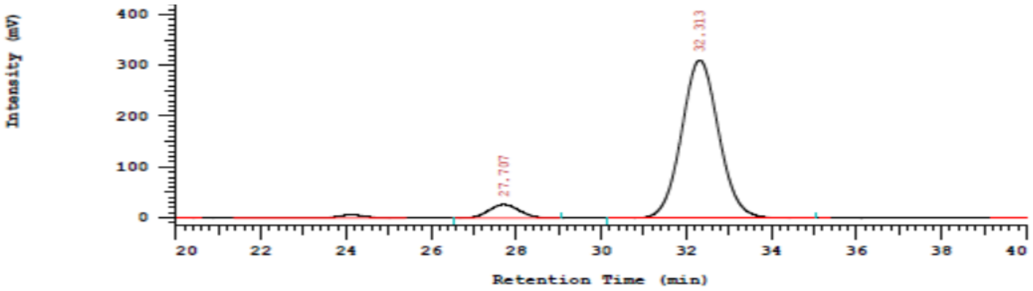

No.	RT	Area	Conc 1	BC
1	27.707	1353226	6.668	MC
2	32.313	18941102	93.332	MC
		20294328	100.000	

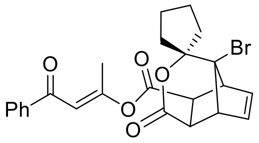

10d’, (E)-4-oxo-4-phenylbut-2-en-2-yl 7a'-bromo-3'-oxo-4',4a',7',7a'-tetrahydro-3'H-spiro[cyclopentane-1,1'-[4,7]methanocyclopenta[c]pyran]-8'-carboxylate

Yield: 90%.

Physical state: orange oil.

$[\alpha]_D^{25}$ 48.1 (c 0.54, $CHCl_3$, 95:5 er).

HPLC (Daicel Chiralpak IC, *i*-PrOH/*n*-hexane = 15/85, 0.8 mL/min, 254 nm) t1= 34.3 min (minor), t2 = 36.6 min (major).

^{1}H NMR (500 MHz, Acetone-*d*$_6$) δ 7.93 (d, *J* = 7.7 Hz, 2H), 7.64 (t, *J* = 7.4 Hz, 1H), 7.54 (t, *J* = 7.7 Hz, 2H), 6.86 (s, 1H), 6.81 (dd, *J* = 5.8, 3.2 Hz, 1H), 6.25 (dd, *J* = 5.8, 3.0 Hz, 1H), 3.77 – 3.72 (m, 1H), 3.74 – 3.69 (m, 1H), 3.01 (s, 2H), 2.75 – 2.66 (m, 1H), 2.32 (s, 3H), 2.28 – 2.19 (m, 1H), 2.16 – 2.09 (m, 1H), 2.02 – 1.94 (m, 1H), 1.87 – 1.67 (m, 4H).

^{13}C NMR (126 MHz, Acetone-*d*$_6$) δ 190.1, 170.9, 169.4, 164.3, 144.9, 139.4, 133.9, 133.7, 129.6, 128.7, 114.6, 97.4, 87.0, 55.0, 54.6, 49.0, 44.0, 42.0, 35.8, 26.2, 25.9, 18.5.

HRMS (ESI) calcd for $C_{24}H_{23}BrO_5Na^+$ $[M+Na]^+$: 493.0621 (Br^{79}), 495.0601 (Br^{81}); found: 493.0622 (Br^{79}), 495.0600 (Br^{81}).

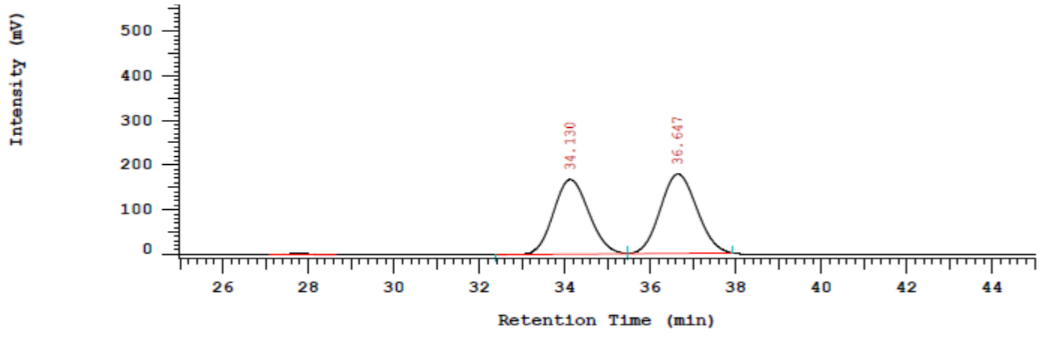

No.	RT	Area	Conc 1	BC
1	34.130	9603493	47.666	MC
2	36.647	10544064	52.334	MC
		20147557	100.000	

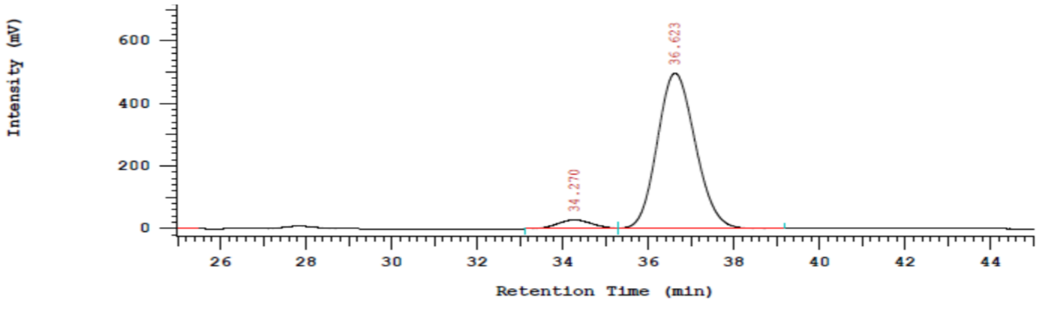

No.	RT	Area	Conc 1	BC
1	34.270	1624922	5.019	MC
2	36.623	30753405	94.981	MC
		32378327	100.000	

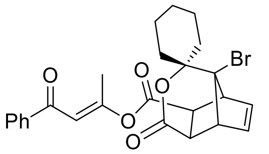

10e’, (E)-4-oxo-4-phenylbut-2-en-2-yl 7a'-bromo-3'-oxo-4',4a',7',7a'-tetrahydro-3'H-spiro[cyclohexane-1,1'-[4,7]methanocyclopenta[c]pyran]-8'-carboxylate

Yield: 93%.

Physical state: orange oil.

$[\alpha]_D^{25}$ 31.2 (c 0.6, $CHCl_3$, 90:10 er).

HPLC (Daicel Chiralpak IA, *i*-PrOH/*n*-hexane = 3/97, 0.6 mL/min, 254 nm) t1= 74.5 min (minor), t2 = 80.1 min (major).

^{1}H NMR (400 MHz, Acetone-*d*$_6$) δ 7.95 (d, *J* = 7.3 Hz, 2H), 7.64 (t, *J* = 7.4 Hz, 1H), 7.54 (t, *J* = 7.6 Hz, 2H), 6.88 (s, 1H), 6.84 (dd, *J* = 5.9, 3.3 Hz, 1H), 6.25 (dd, *J* = 5.8, 3.0 Hz, 1H), 3.83 (s, 1H), 3.76 (s, 1H), 3.03 – 2.95 (m, 2H), 2.44 (td, *J* = 13.3, 4.3 Hz, 1H), 2.38 – 2.30 (m, 4H), 2.03 – 1.98 (m, 1H), 1.94 – 1.85 (m, 1H), 1.82 – 1.72 (m, 1H), 1.71 – 1.62 (m, 1H), 1.62 – 1.51 (m, 3H), 1.24 – 1.15 (m, 1H).

^{13}C NMR (101 MHz, Acetone-*d*$_6$) δ 190.1, 171.1, 169.3, 164.3, 145.5, 139.4, 134.1, 133.9, 129.6, 128.8, 114.7, 88.2, 87.5, 53.5, 53.4, 49.5, 43.9, 36.4, 30.7, 25.6, 22.6, 21.5, 18.5.

HRMS (APCI) calcd for $C_{25}H_{26}BrO_5^+$ $[M+H]^+$: 485.0958 (Br^{79}), 487.0942 (Br^{81}); found: 485.0959 (Br^{79}), 487.0941 (Br^{81}).

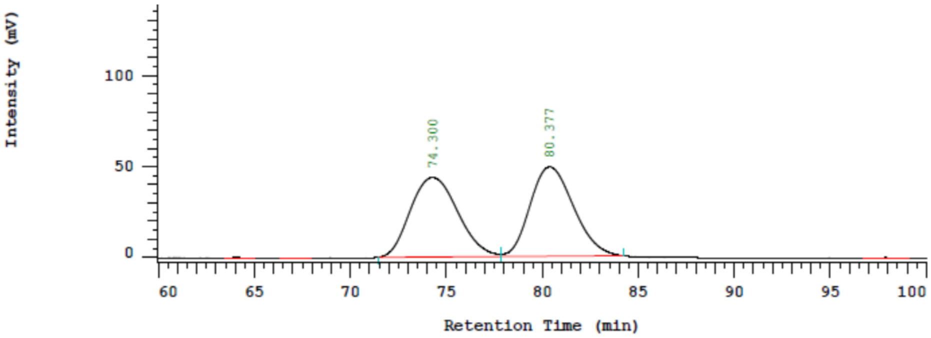

No.	RT	Area	Conc 1	BC
1	74.300	7481901	49.303	BV
2	80.377	7693367	50.697	VB
		15175268	100.000	

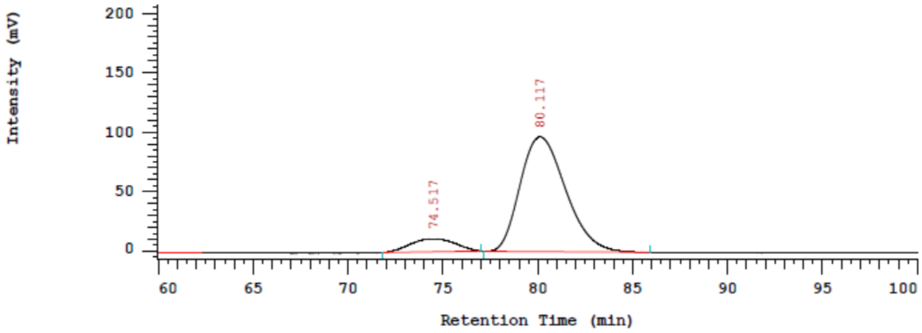

No.	RT	Area	Conc 1	BC
1	74.517	1815293	10.146	MC
2	80.117	16077089	89.854	MC
		17892382	100.000	

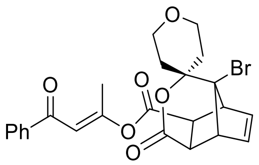

10f', (E)-4-oxo-4-phenylbut-2-en-2-yl 7a'-bromo-3'-oxo-2,3,4',4a',5,6,7',7a'-octahydro-3'H-spiro[pyran-4,1'-[4,7]methanocyclopenta[c]pyran]-8'-carboxylate

Yield: 88%.

Physical state: orange oil.

$[\alpha]_D^{25}$ 30.3 (c 0.33, $CHCl_3$, 90:10 er).

HPLC (Daicel Chiralpak ID, *i*-PrOH/*n*-hexane = 20/80, 0.8 mL/min, 254 nm) t1= 74.8 min (minor), t2 = 85.2 min (major).

^{1}H NMR (500 MHz, Acetone-d_6) δ 7.97 – 7.92 (m, 2H), 7.67 – 7.62 (m, 1H), 7.58 – 7.53 (m, 2H), 6.88 – 6.84 (m, 2H), 6.30 – 6.25 (m, 1H), 3.90 – 3.85 (m, 1H), 3.81 – 3.75 (m, 4H), 3.63 – 3.57 (m, 1H), 3.05 (d, J = 1.2 Hz, 2H), 2.82 – 2.75 (m, 1H), 2.39 – 2.30 (m, 4H), 2.20 – 2.15 (m, 1H), 1.80 – 1.74 (m, 1H).

^{13}C NMR (126 MHz, Acetone-d_6) δ 190.1, 170.7, 169.5, 164.2, 145.4, 139.4, 134.0, 133.9, 129.6, 128.8, 114.7, 87.2, 85.2, 64.1, 63.0, 53.1, 53.1, 49.4, 44.0, 36.6, 31.2, 18.4.

HRMS (ESI) calcd for $C_{24}H_{23}BrO_6Na^+$ $[M+Na]^+$: 509.0570 (Br^{79}), 511.0550 (Br^{81}); found: 509.0564 (Br^{79}), 511.0541 (Br^{81}).

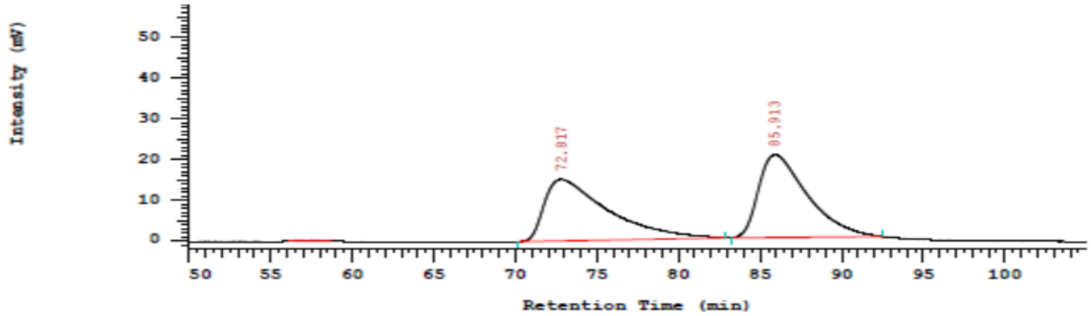

No.	RT	Area	Conc 1	BC
1	72.817	4054503	49.106	MC
2	85.913	4202105	50.894	MC
		8256608	100.000	

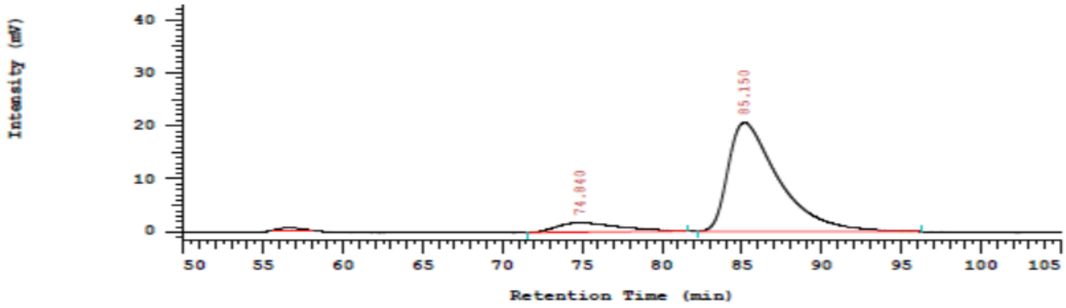

No.	RT	Area	Conc 1	BC
1	74.840	488083	9.816	MC
2	85.150	4484126	90.184	MC
		4972209	100.000	

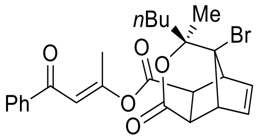

10g’, (E)-4-oxo-4-phenylbut-2-en-2-yl 7a-bromo-1-butyl-1-methyl-3-oxo-1,3,4,4a,7,7a-hexahydro-4,7-methanocyclopenta[c]pyran-8-carboxylate

Yield: 87%.

Physical state: orange oil.

$[\alpha]_D^{25}$ 31.8 (c 0.28, $CHCl_3$, 90:10 er). 2:1 *dr*.

HPLC (Daicel Chiralpak IC, *i*-PrOH/*n*-hexane = 15/85, 0.6 mL/min, 254 nm) t1= 34.4 min (minor), t2 = 36.6 min (major).

^{1}H NMR (500 MHz, Acetone-*d*$_6$) δ 7.94 (d, *J* = 7.3 Hz, 2H), 7.64 (t, *J* = 7.4 Hz, 1H), 7.54 (t, *J* = 7.7 Hz, 2H), 6.85 (s, 1H), 6.82 (dd, *J* = 5.8, 3.3 Hz, 1H), 6.24 (dd, *J* = 5.8, 3.0 Hz, 1H), 3.87 (s, 1H), 3.79 (s, 1H), 3.03 (d, *J* = 8.6 Hz, 1H), 2.97 (d, *J* = 8.7 Hz, 1H), 2.32 (s, 3H), 2.29 – 2.24 (m, 1H), 2.11 – 2.07 (m, 1H), 1.71 (s, 3H), 1.47 – 1.39 (m, 2H), 1.34 – 1.27 (m, 2H), 0.84 (t, *J* = 7.3 Hz, 3H).

^{13}C NMR (126 MHz, Acetone-*d*$_6$) δ 190.1, 171.0, 169.1, 164.3, 145.3, 139.4, 133.9, 133.8, 129.6, 128.7, 114.5, 88.6, 88.1, 53.8, 53.2, 49.2, 43.4, 35.2, 26.9, 25.6, 23.5, 18.5, 14.3.

HRMS (ESI) calcd for $C_{25}H_{27}BrO_5Na^+$ $[M+Na]^+$: 509.0934 (Br^{79}), 511.0914 (Br^{81}); found: 509.0932 (Br^{79}), 511.0910 (Br^{81}).

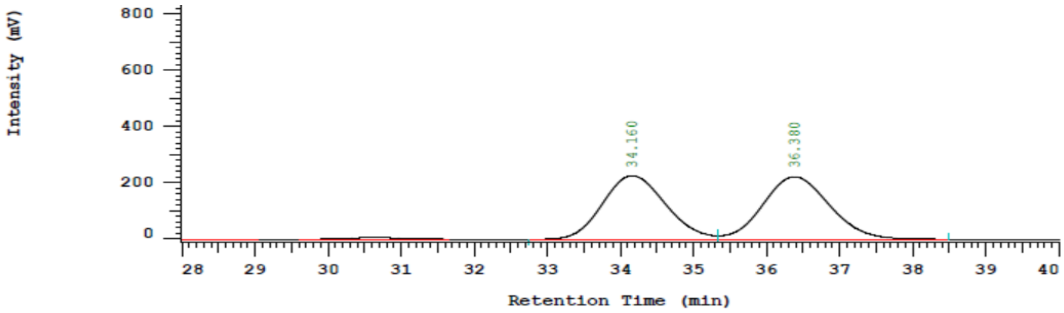

No.	RT	Area	Conc 1	BC
1	34.160	13469643	49.064	BV
2	36.380	13983321	50.936	VB
		27452964	100.000	

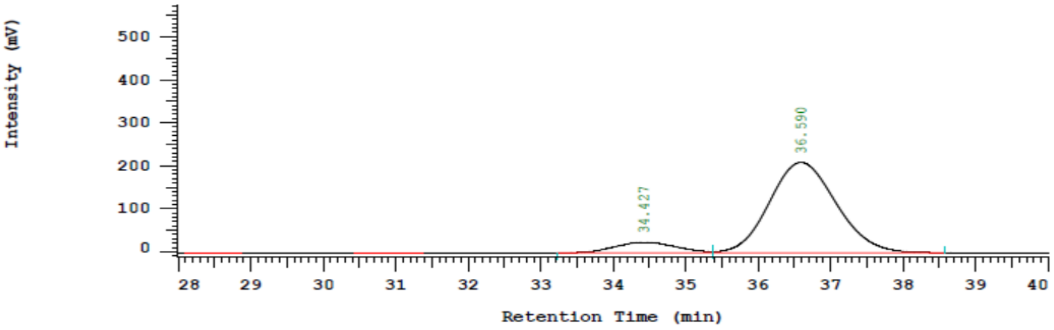

No.	RT	Area	Conc 1	BC
1	34.427	1459664	9.783	BV
2	36.590	13460940	90.217	VB
		14920604	100.000	

5. Synthesis of compound 13

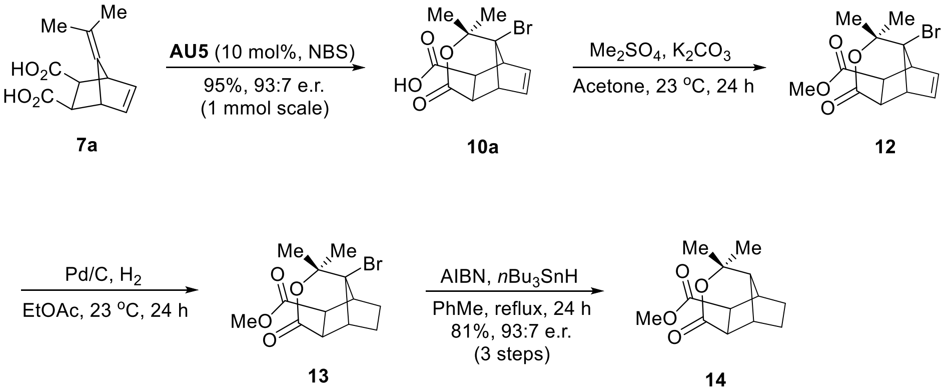

To a solution of **7a** (1.0 mmol, 1.0 equiv) in toluene (40.0 mL, 0.025 M) was added catalyst **AU5** (10 mol%) and 3Å MS (150.0 mg). The mixture was stirred at -40 °C for 30 min and NBS (187.0 mg, 1.05 equiv) was added in the absence of light. The resultant mixture was stirred at -40 °C for 3 d. The solution was filtered through a thin plug of celite. The filtrate was concentrated under reduced pressure and the residue was purified using flash column chromatography to give **10a** (95%).

To a solution of **10a** (1.0 equiv) in acetone (20.0 mL, 0.05 M) was added K_2CO_3 (276.0 mg, 2.0 equiv) and Me_2SO_4 (252.0 mg, 2.0 equiv). The resultant mixture was stirred at room temperature for 24 h. The solution was filtered through a thin plug of silica gel eluted with EtOAc. The filtrate was concentrated under reduced pressure to give crude product **12**.

Ester **12** (283.5 mg, 1.0 equiv) was dissolved in ethyl acetate (9.0 mL, 0.1 M) and palladium (5% on C) was added. The suspension was stirred at room temperature for 24 h under hydrogen atmosphere with a hydrogen balloon. The mixture was then filtered through a thin plug of celite and the filtrate was concentrated under reduced pressure to give ester **13**, which was used in the next step directly.

To a solution of **13** (282.4 mg, 1.0 equiv) in toluene (8.9 mL, 0.1 M) was added AIBN (15.0 mg, 10 mol%) and *n*Bu_3SnH (647.5 mg, 2.5 eq.) under nitrogen atmosphere. The mixture was heated at reflux for 24 h. The solution was cooled to room temperature and concentrated under reduced pressure. The residue was purified by flash column chromatography to give product **14** (194.9 mg, 81% in 3 steps from **10a**).

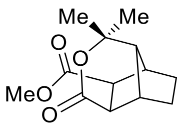

14, Methyl 1,1-dimethyl-3-oxooctahydro-4,7-methanocyclopenta[c]pyran-8-carboxylate [3]

Yield: 81% (3 steps from **10a**).

Physical state: white solid.

$[\alpha]_D^{25}$ 20.2 (c 0.25, $CHCl_3$, 93:7 er).

HPLC (Daicel Chiralpak IA, *i*-PrOH/*n*-hexane = 10/90, 0.8 mL/min, 214 nm) t1= 20.1 min (major), t2 = 24.5 min (minor).

^{1}H NMR (500 MHz, Chloroform-*d*) δ 3.65 (s, 3H), 2.96 – 2.90 (m, 2H), 2.61 (d, *J* = 5.1 Hz, 1H), 2.55 (d, *J* = 8.0 Hz, 1H), 1.88 – 1.80 (m, 2H), 1.70 – 1.62 (m, 1H), 1.47 – 1.35 (m, 2H), 1.43 (s, 3H), 1.37 (s, 3H).

^{13}C NMR (126 MHz, Chloroform-*d*) δ 171.9, 171.8, 82.6, 54.2, 52.1, 49.0, 48.5, 39.9, 38.8, 31.9, 31.1, 26.3, 25.2.

HRMS (ESI) calcd for $C_{13}H_{18}O_4Na^+$ $[M+Na]^+$: 261.1097; found: 261.1094.

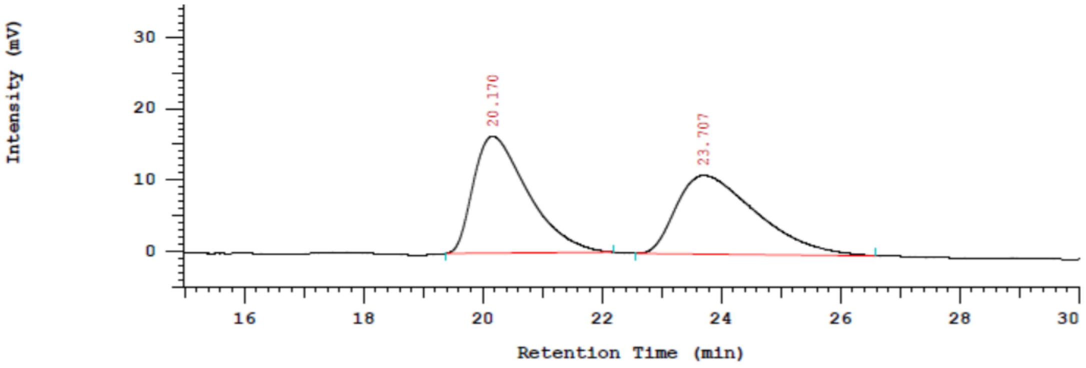

No.	RT	Area	Conc 1	BC
1	20.170	1042812	50.249	MC
2	23.707	1032460	49.751	MC
		2075272	100.000	

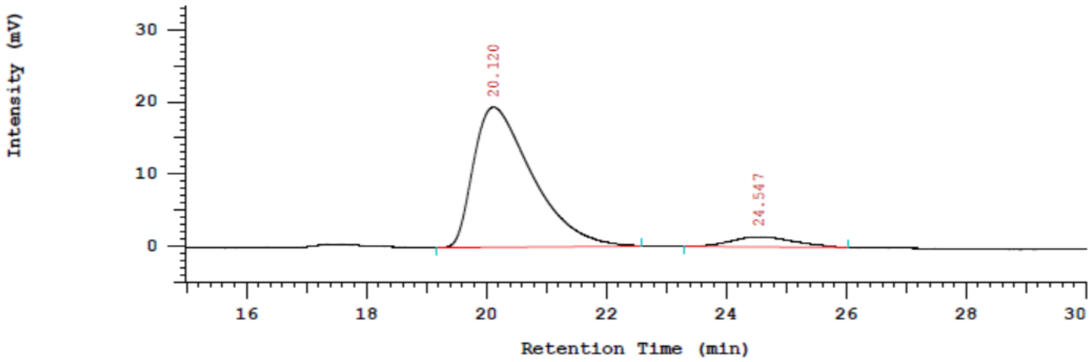

No.	RT	Area	Conc 1	BC
1	20.120	1322561	92.785	MC
2	24.547	102838	7.215	MC
		1425399	100.000	

6. Catalyst screening

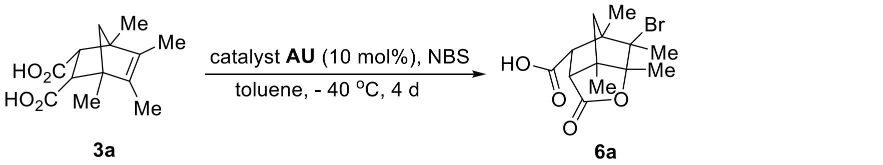

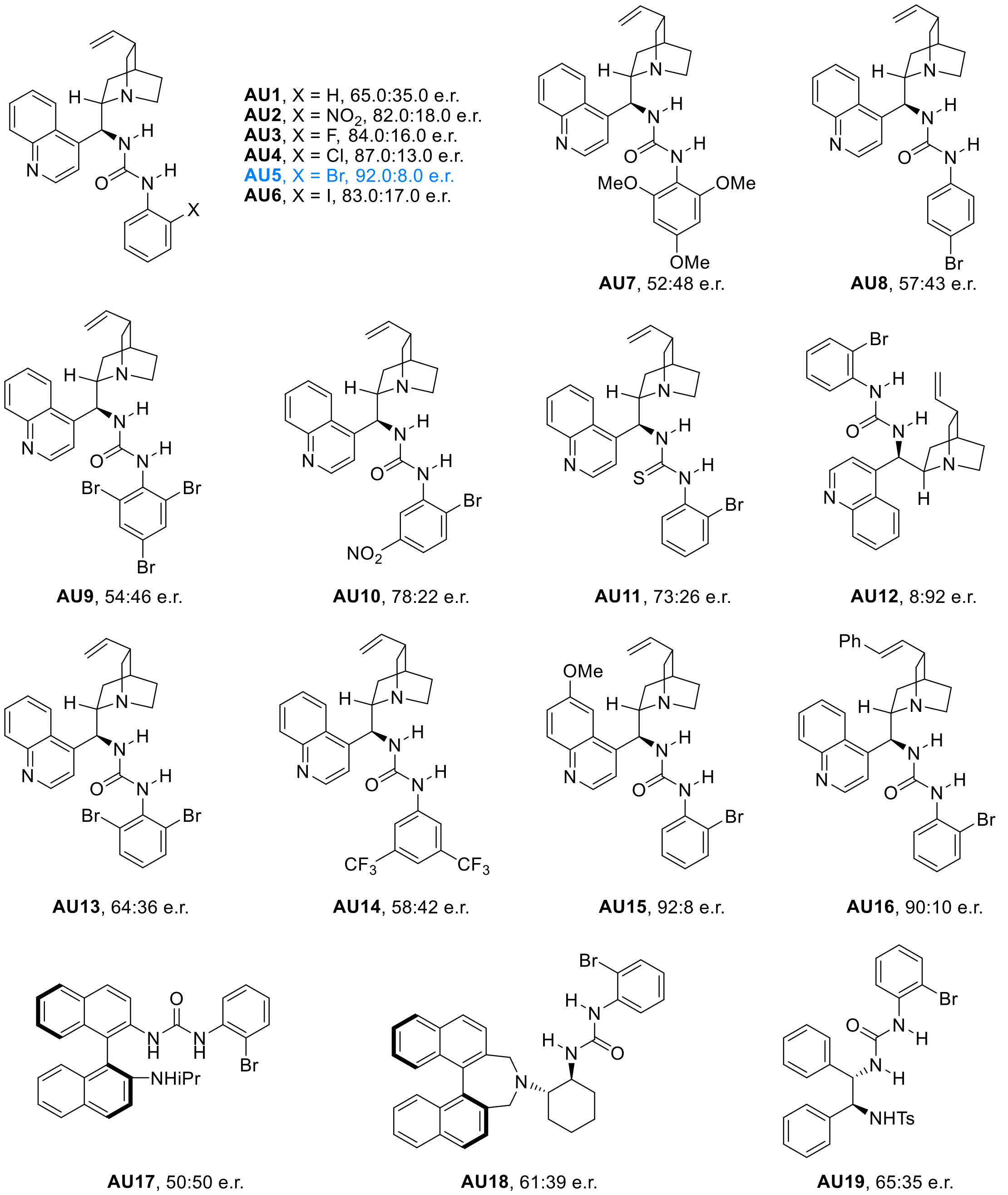

Figure S1. Catalyst study.

7. Kinetic studies

Reaction orders were studied using the initial rate method. Components including diacid substrate **3a**, NBS, and catalyst **AU5** were used in the study. The reactions were carried out at 0.05 mmol scale and the amount of one of the components was varied according to the values indicated in Figure S2-S4. The amount of product was measured after 2 h in each of the reactions.

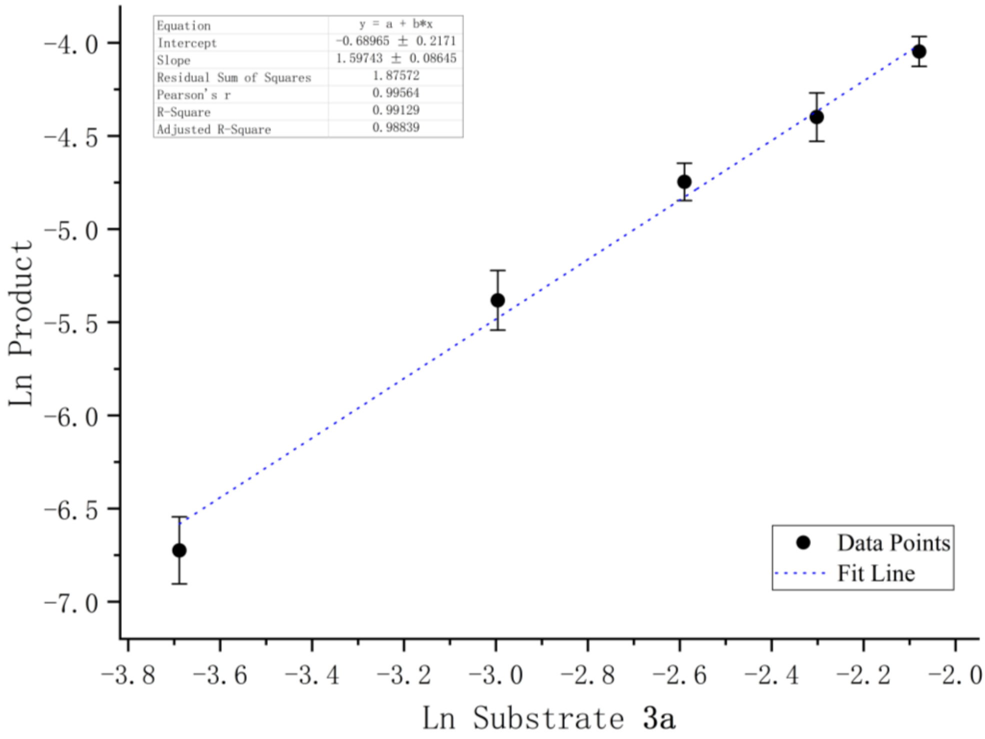

Acid/mmol	Ln Acid	Product/mmol	Ln Product
0.025	-3.689	0.0012	-6.725
0.050	-2.996	0.0046	-5.382
0.075	-2.59	0.0089	-4.746
0.100	-2.302	0.0123	-4.398
0.125	-2.079	0.0175	-4.046

Figure S2. Reaction order with respect to diacid substrate **3a**.

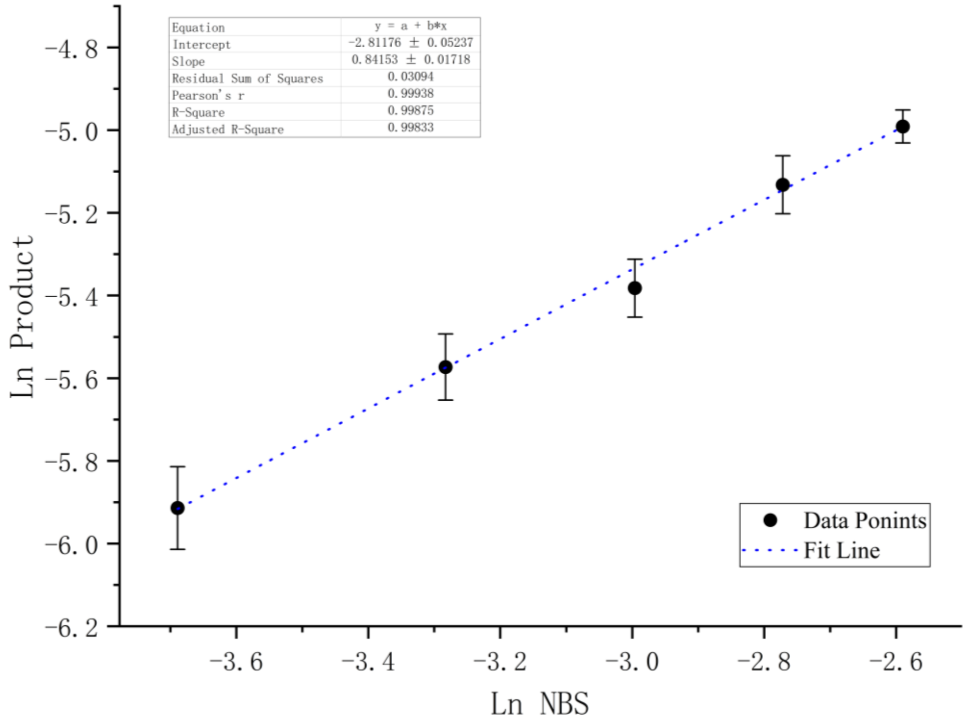

NBS/mmol	Ln NBS	Product/mmol	Ln Product
0.025	-3.689	0.0027	-5.914
0.0375	-3.283	0.0038	-5.573
0.050	-2.996	0.0046	-5.382
0.0625	-2.772	0.0059	-5.034
0.0750	-2.590	0.0068	-4.991

Figure S3. Reaction order with respect to NBS.

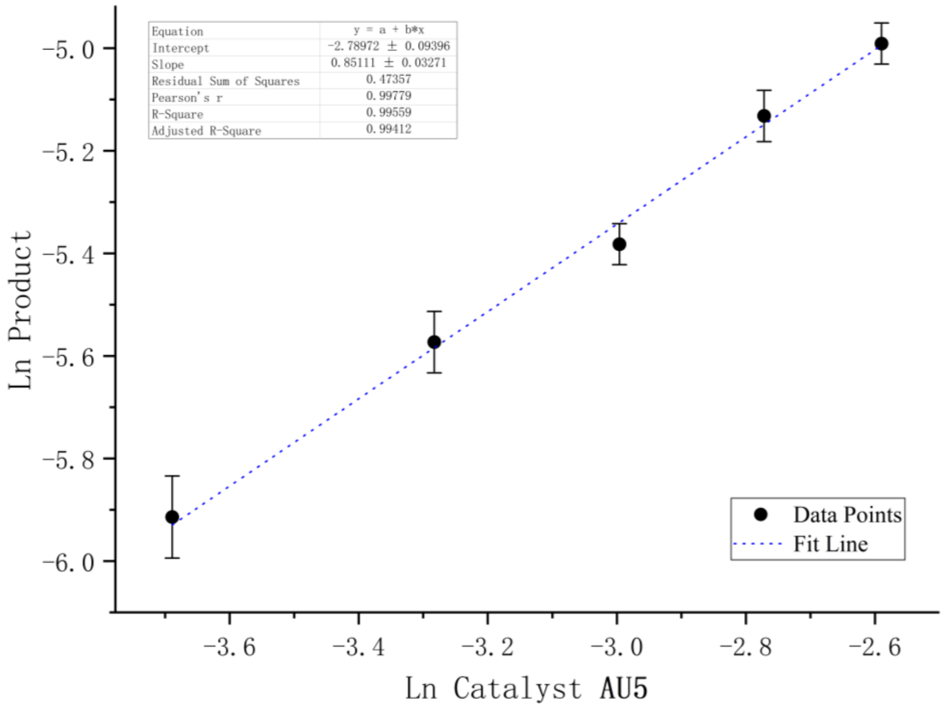

Cat./mmol	Ln Cat.	Product/mmol	Ln Product
0.00125	-6.685	0.0015	-6.502
0.0025	-5.991	0.003	-5.809
0.005	-5.298	0.0046	-5.382
0.0075	-4.893	0.0063	-5.067
0.0100	-4.605	0.0081	-4.815

Figure S4. Reaction order with respect to catalyst **AU5**.

8. NMR experiments

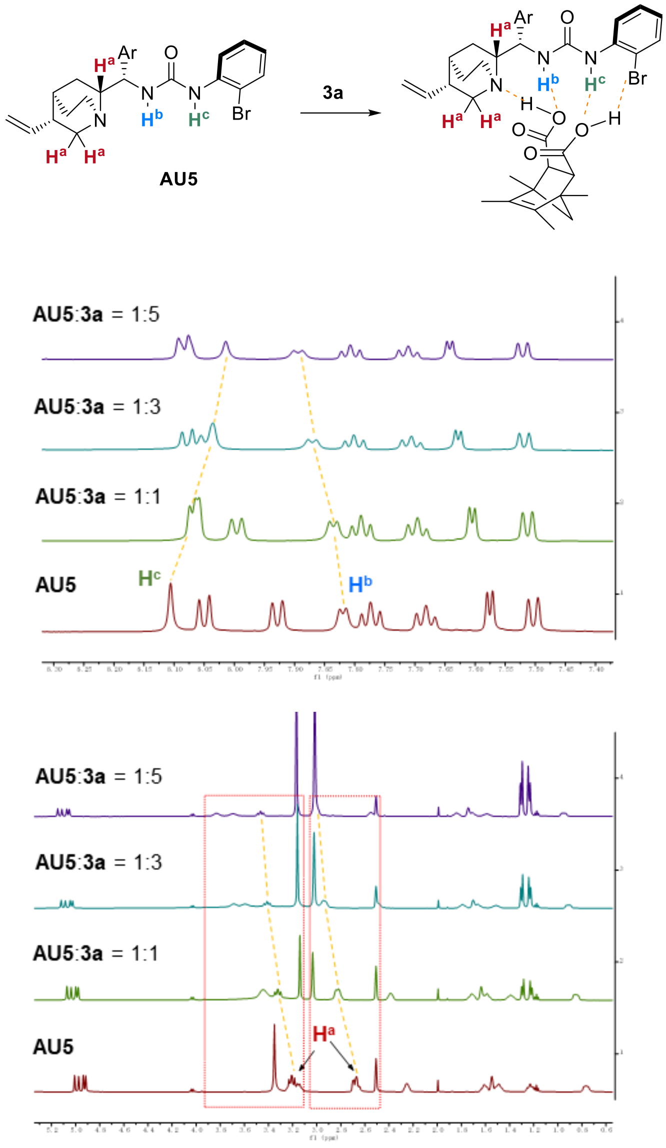

Figure S5. NMR experiments.

*Note: ^{1}H NMR titration experiments were carried out on a mixture of **AU5** (0.01 mmol) and **3a** in $CDCl_3$ (0.5 mL). Significant downfield shift of the protons (H^a) that are adjacent to the quinuclidine nitrogen was observed, suggesting that the nitrogen was protonated. In addition, the urea hydrogens (H^b and H^c) also exhibited significant chemical shifts, attributed to the hydrogen bond interactions between **AU5** and **3a**. The unusual upfield shift of H^c could be attributed to the interaction due to halide bond with the Br.*

9. Computational studies

The geometry optimization and frequency analysis were carried out using Gaussian 16 (version C.02)[11] and ORCA (version 6.1.0)[12] with a CPCM solvent model (toluene) at the level of M06-2X/def2-SVP with Grimme D3(0) dispersion correction.[13-17] The single-point energies were calculated at the calculation level of ωB97X-2 functional /def2-TZVPP basis set with Grimme D3BJ dispersion correction.[18-20] The auxiliary basis set def2-TZVPP/C[21] was utilized for Resolution of Identity (RI) approximation to accelerate the ORCA calculations. To investigate the interactions within multi-component complexes, constrained molecular simulations for conformational searching were conducted using xTB[22] with the GFN2-xTB model.[23] Several optimized configurations were obtained, and the one with the lowest potential energy was selected for further study. Thermal corrections to the Gibbs free energy at 233 K were computed with Shermo using Minenkov's quasi-RRHO approximation to account for low-frequency vibrational modes.[24-25] In addition, zero-point vibrational energy scale factors were established based on Martin's benchmark.[26] The solvation free energies were obtained using the SMD toluene solvation model[27] with its relative permittivity (ε_r) adjusted to 2.52 at 233 K obtained from a linear extrapolation (R^2 = 0.9999) using the reference value from 454 to 273 K (see Figure S17).[28] Natural Bond Orbital (NBO) analysis,[29] including second-order perturbation E(2), was performed at the level of M06-2X /def2-TZVP[5] using NBO 7.0.[30] Non-Covalent Interactions (NCI)[31] and Atom in Molecules (AIM) analysis[32] were carried out using Multiwfn 3.8(dev).[33-34] Snapshots of the molecular structures were produced with VMD 1.9.4[35] and CYLview20.[36]

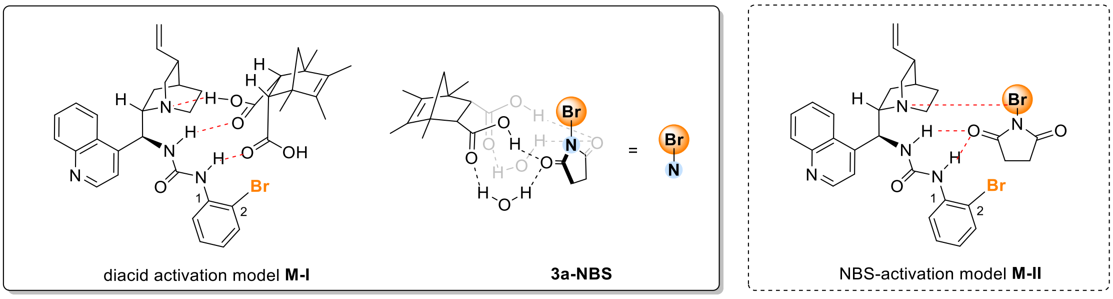

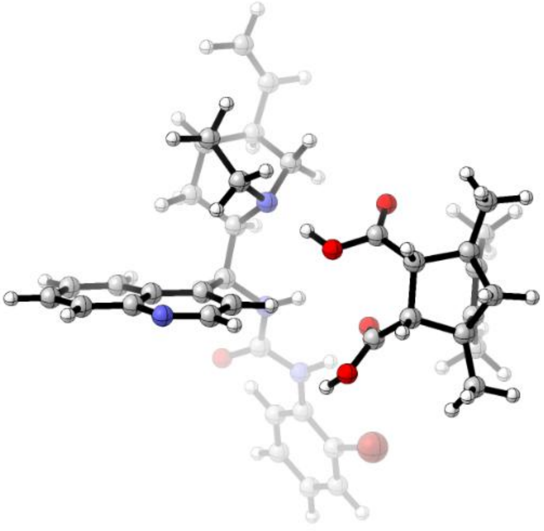

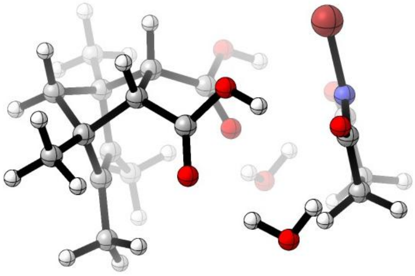

M-I **3a-NBS**

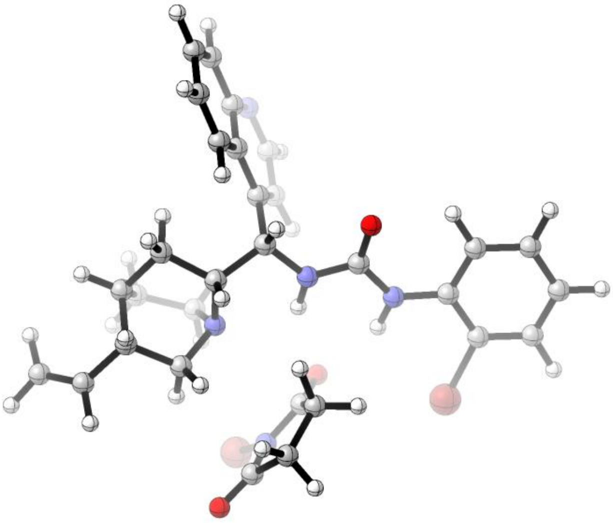

M-II

Figure S6. Computational study on the initial complex.

*Note: we considered two potential initial interaction models among the reaction components: the diacid activation model **M-I** and NBS activation model **M-II**. Computational results indicated that the formation of **M-I** is exergonic by 4.5 kcal/mol relative to its starting materials, whereas the formation of **M-II** is endergonic by 2.7 kcal/mol. On the other hand, the complexation between the diacid **3a** and NBS in the presence of two explicit water molecules to form complex **3a-NBS** increased the free energy slightly by 2.7 kcal/mol.*

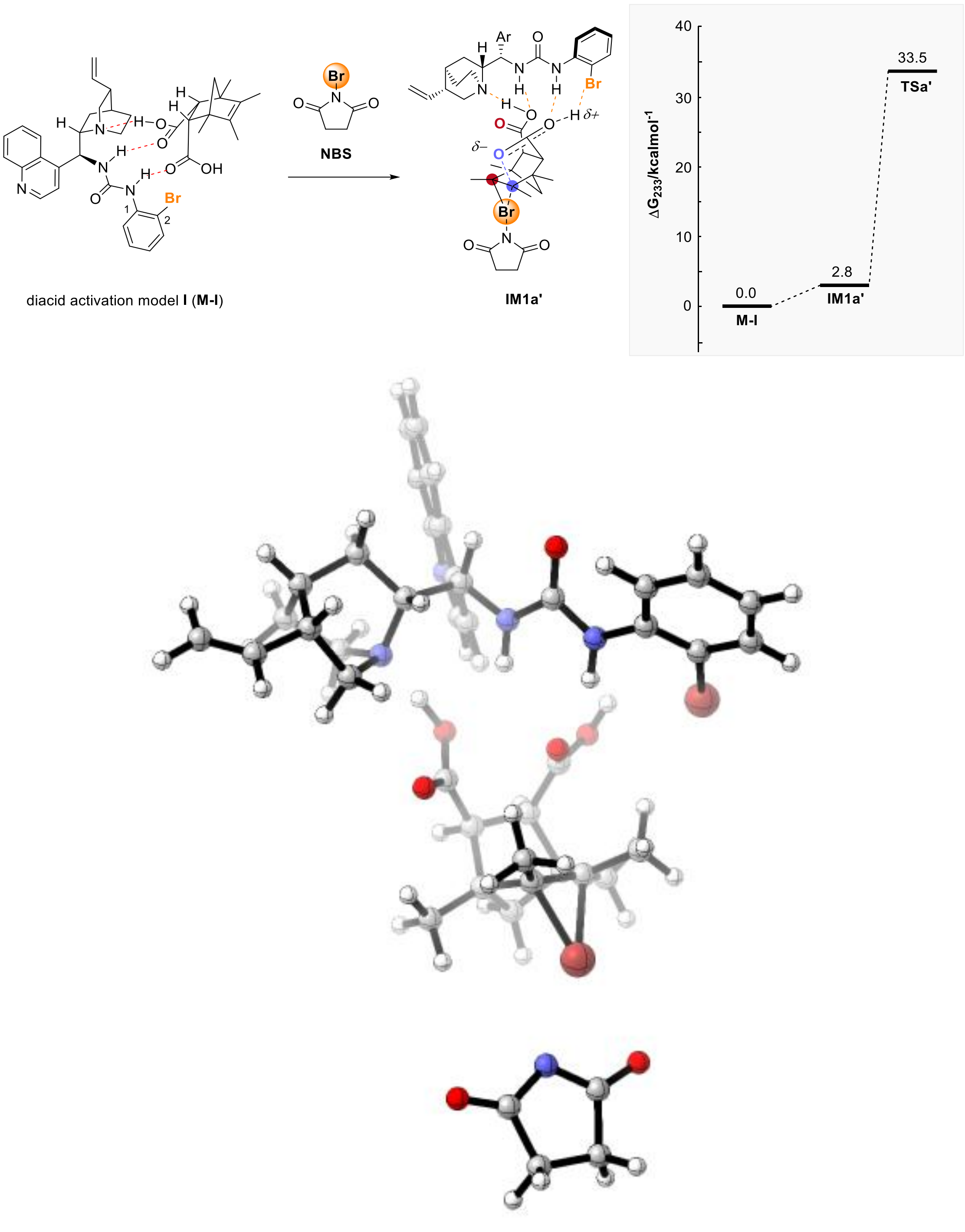

TSa'

Figure S7. Computational study on the **TS** with one diacid molecule.

*Note: the calculation of **IM1a'** and **TSa'** that contained one diacid molecule was carried out. It was calculated that the reaction of species **M-1** with a molecule of NBS gave **IM1a'** and subsequent **TSa'**. Their energies were found to be considerably higher than those of the cases with two diacid molecules.*

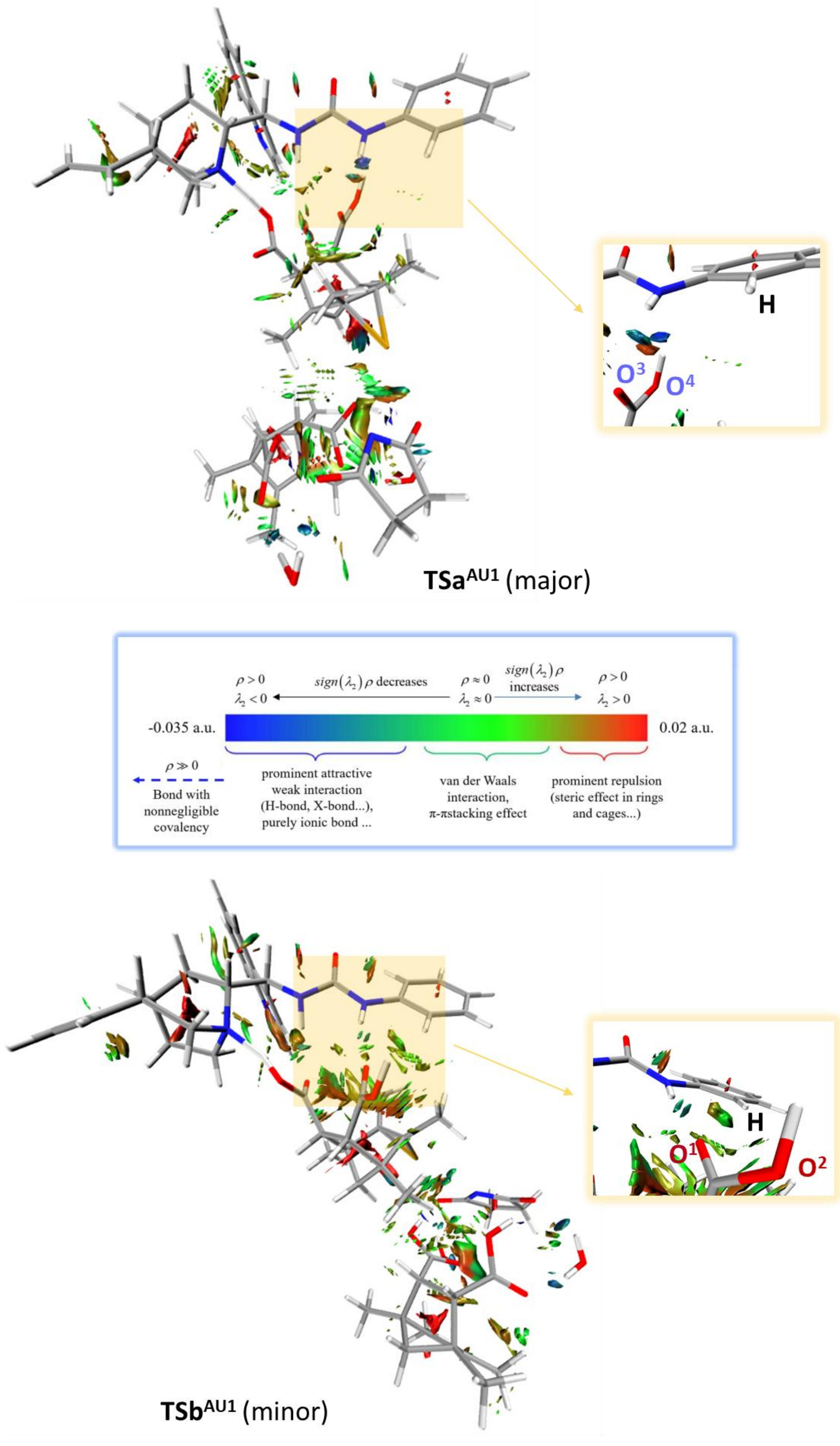

Figure S8. NCI plots of **TSa^{AU1}** and **TSb^{AU1}**.

Note: The isosurfaces correspond to a reduced density gradient of 0.50. The colors are explained in the respective color bar. No NCI between the hydrogens of phenyl and the OH of the diacid substrate were observed.

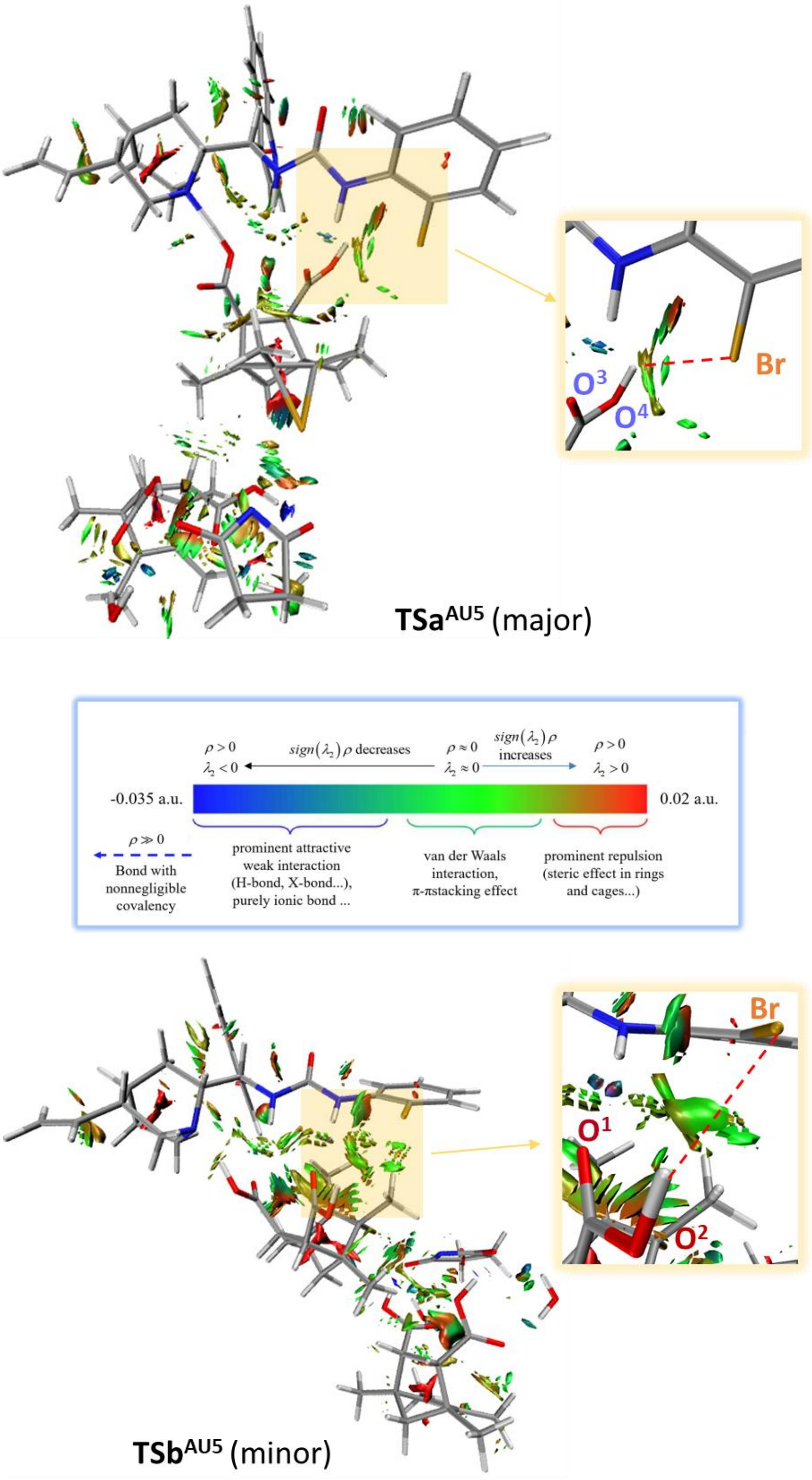

Figure S9. NCI plots of $\mathbf{TSa^{AU5}}$ and $\mathbf{TSb^{AU5}}$.

Note: The isosurfaces correspond to a reduced density gradient of 0.50. The colors are explained in the respective color bar. Significant NCI between the Br of phenyl and the OH of the diacid substrate were observed in $\boldsymbol{TSa^{AU5}}$ *(major). Such NCI were also found in* $\boldsymbol{TSb^{AU5}}$*(minor), although they were much weaker.*

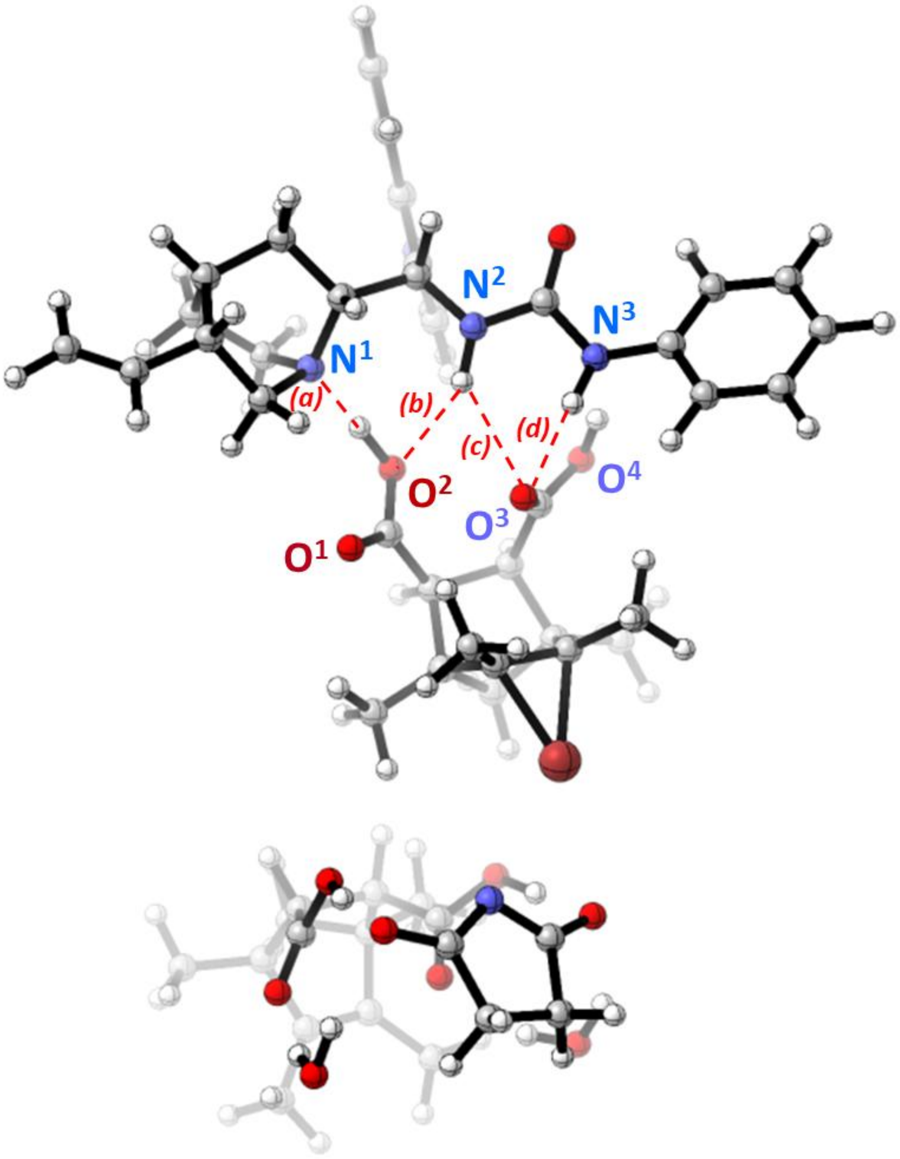

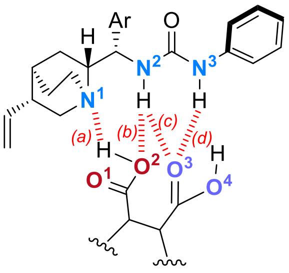

Interaction	Distance	ρ
(a) N^1(LP)---H-O^2(σ*)	1.48 Å	0.0955
(b) N^2-H(σ*)---O^2(LP)	2.43 Å	0.0097
(c) N^2-H(σ*)---O^3(LP)	2.37 Å	0.0110
(d) N^3-H(σ*)---O^3(LP)	2.06 Å	0.0227

Figure S10. Computational study on the hydrogen bonds in **TSa**$^{\mathbf{AU1}}$ with catalyst **AU1**.

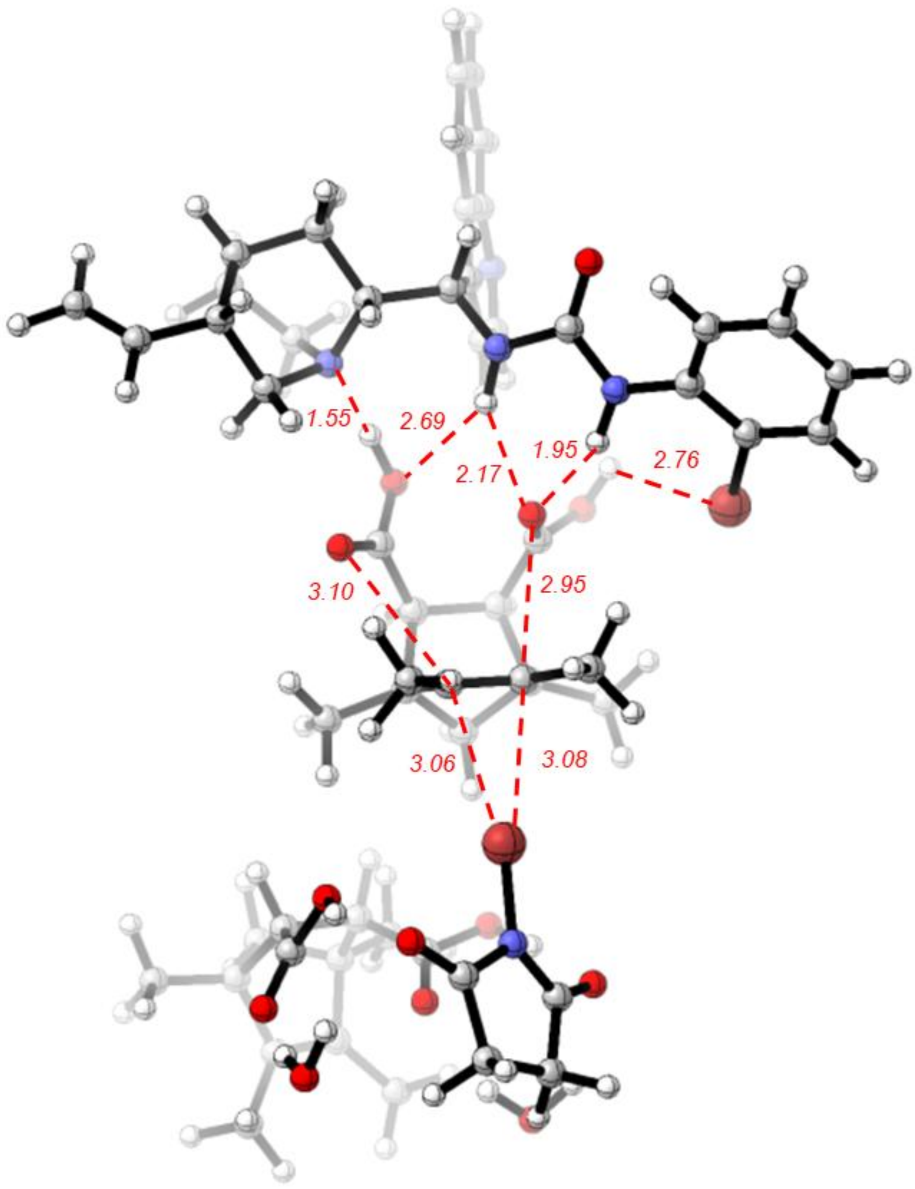

Figure S11. Snapshot of **IM1a**.

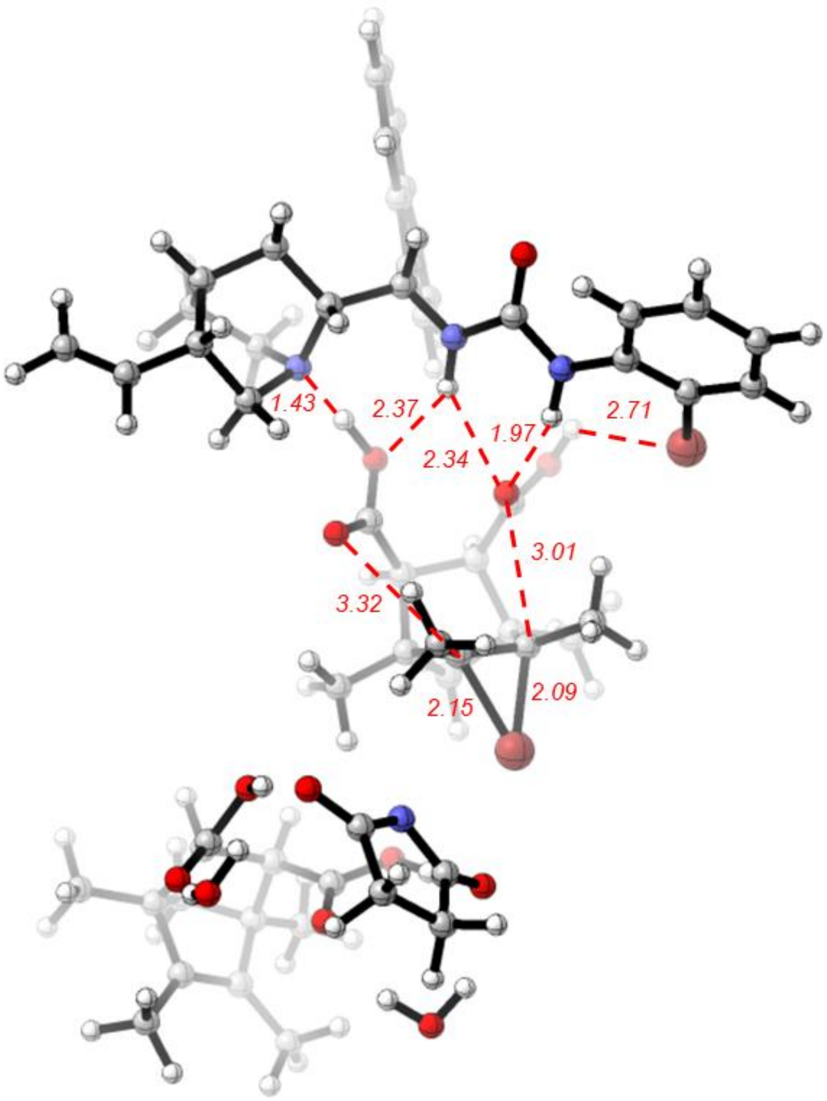

Figure S12. Snapshot of **IM2a**.

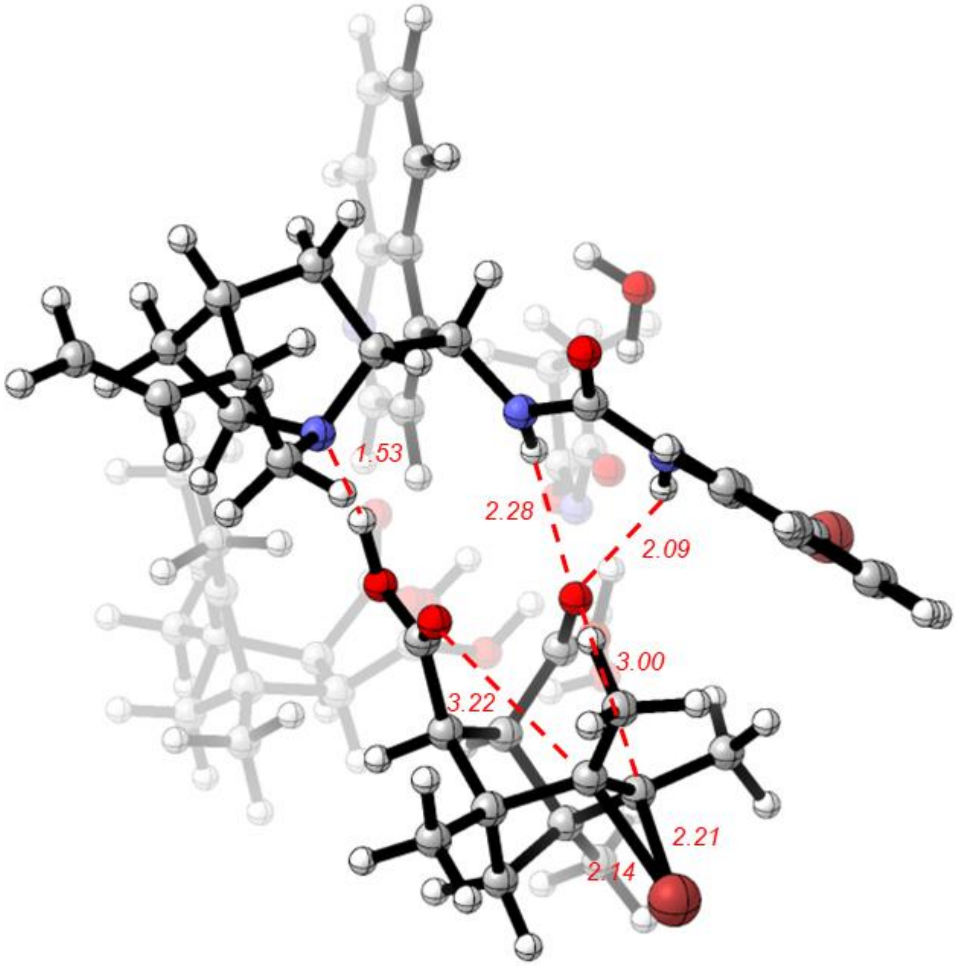

Figure S13. Snapshot of **IM3a**.

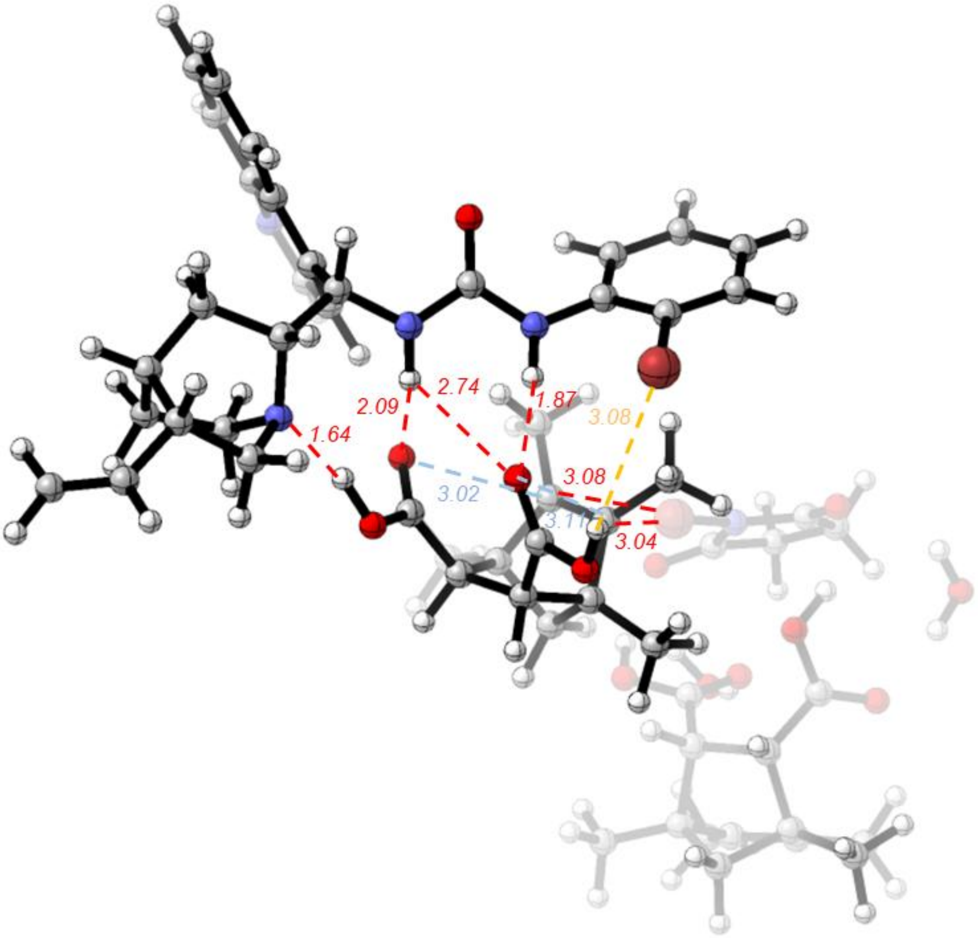

Figure S14. Snapshot of **IM1b**.

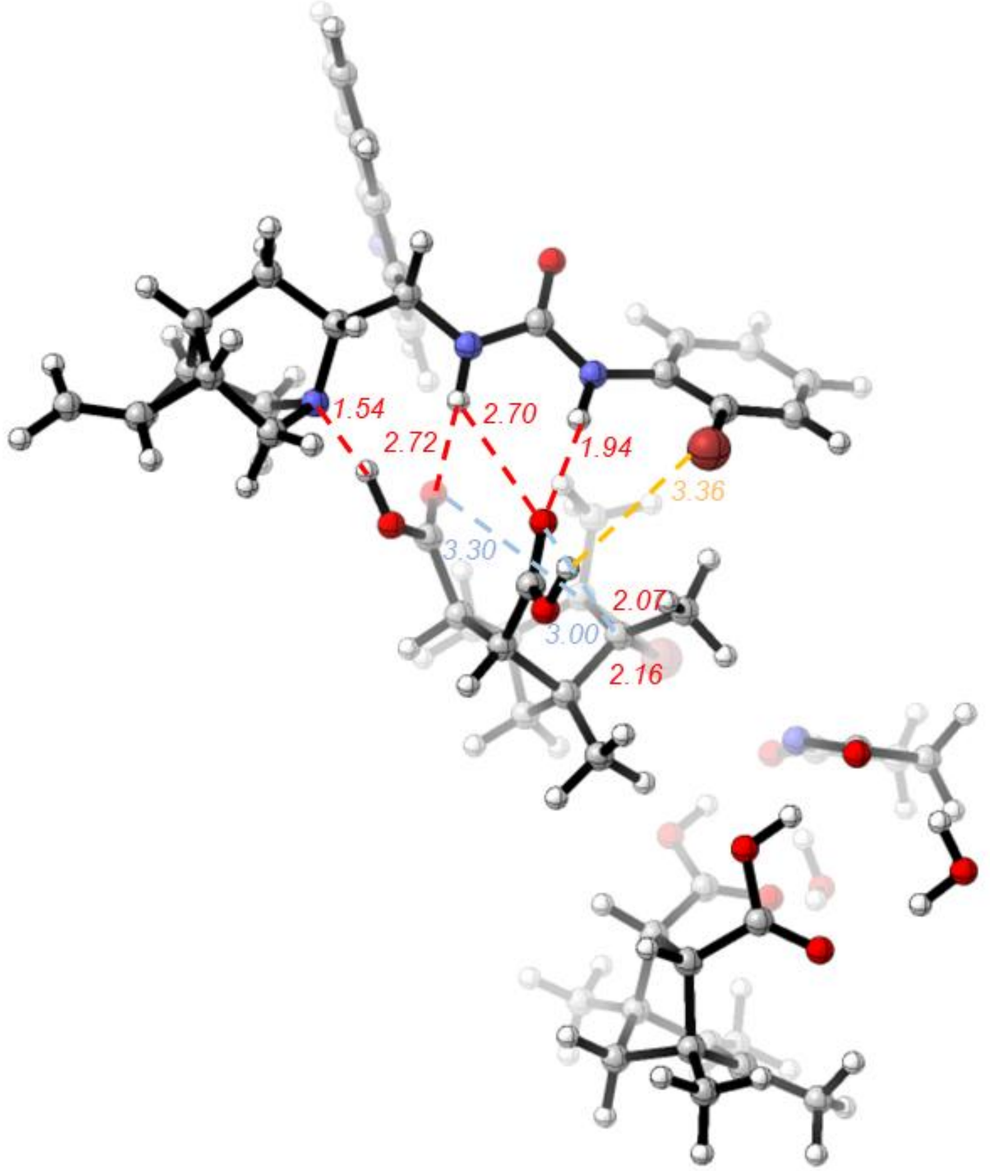

Figure S15. Snapshot of **IM2b**.

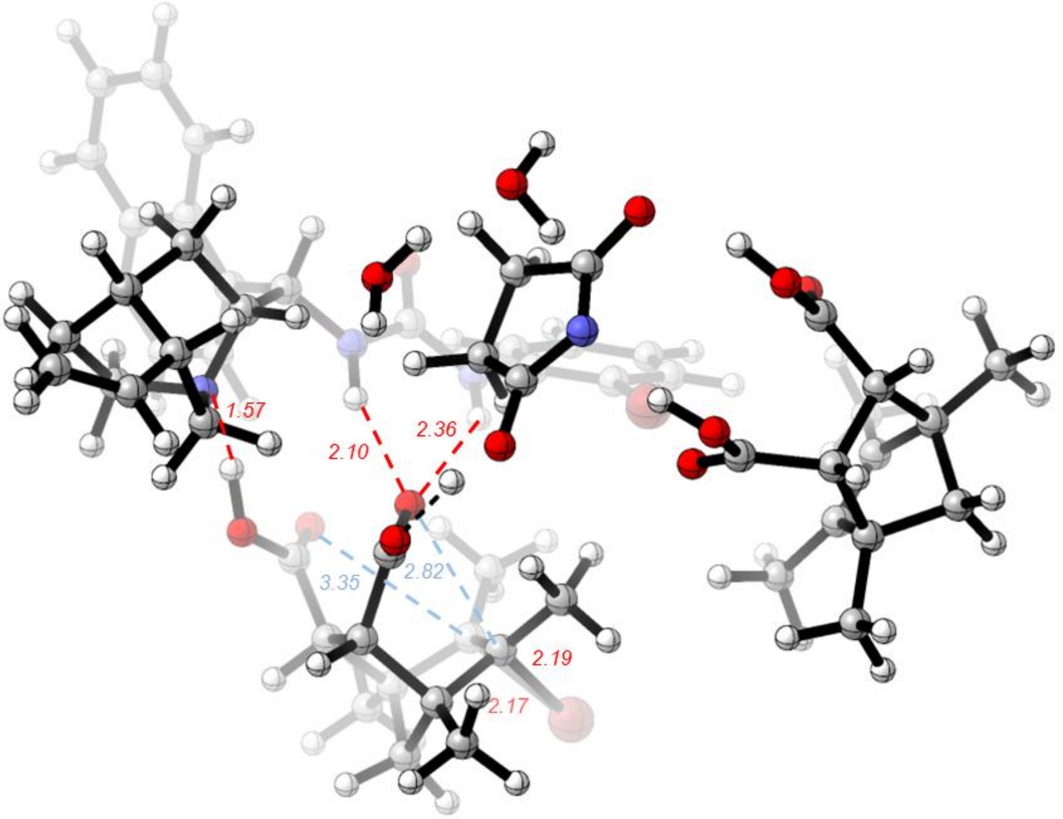

Figure S16. Snapshot of **IM3b**.

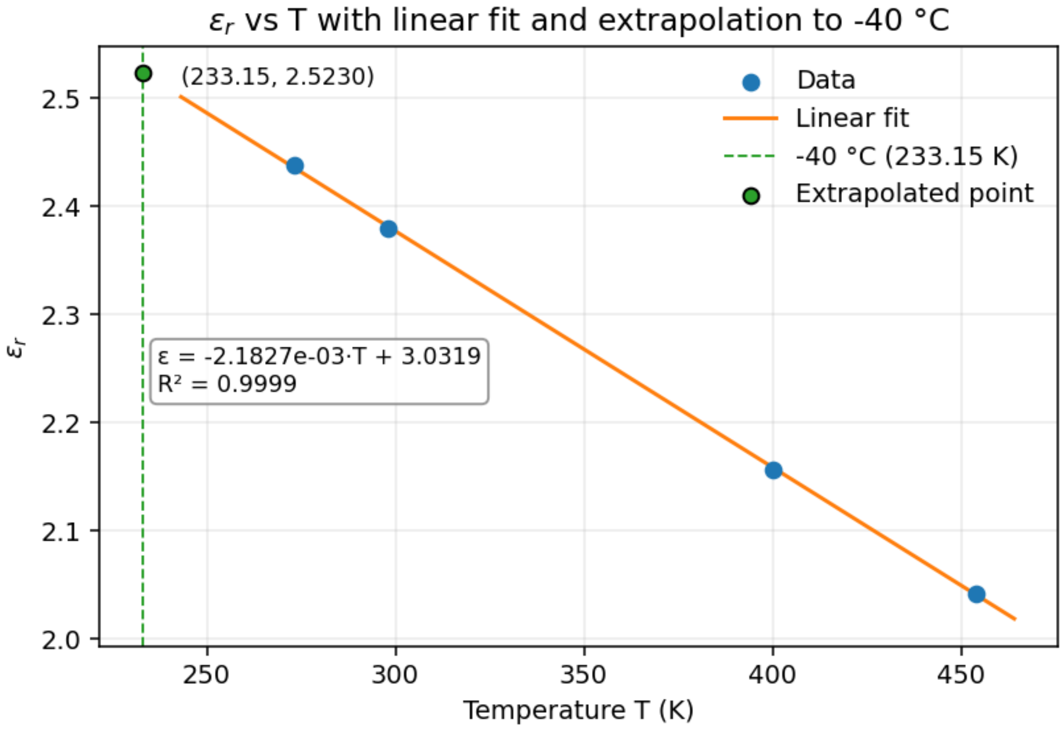

Figure S17. Linear extrapolation of the relative permittivity ε_r as a function of the temperature. Reference data was obtained from Table of Dielectric Constants of Pure Liquids.[28]

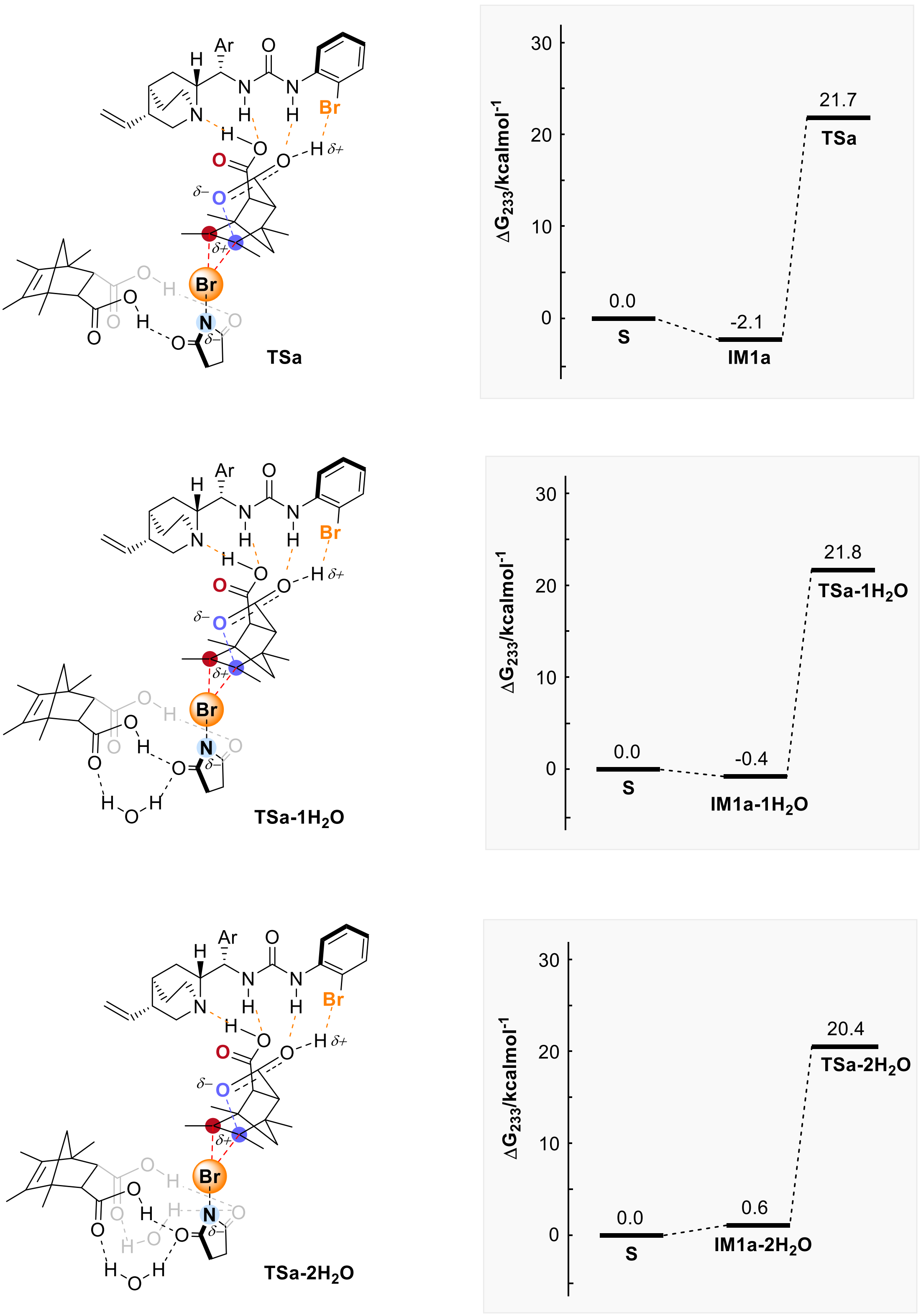

Figure S18. Free energy barrier measured from the lowest free energy to **TSa** with explicit water molecule.

Note: In searching for the transition states, it was found that explicit water molecules can reduce the free energy barriers. The free energy barrier is reduced from 23.8 kcal/mol to 22.2 kcal/mol with one explicit water molecule. With two explicit water molecules, the barrier is further reduced to 20.4 kcal/mol.

Cartesian Coordinates

3a

E (ωb97X-2 D3BJ/def2-TZVPP) = -805.653001007029

C	-1.57390100	1.49520200	-0.06177200
O	-1.31625600	2.76234900	-0.40803400
O	-2.37574700	1.20832200	0.78720800
C	-0.81484600	0.52760600	-0.93866800
C	0.73469200	0.62414300	-0.90340000
C	1.31650600	1.46521400	0.20230200
O	2.56132800	1.86694200	-0.08200700
O	0.78363700	1.71267900	1.25279000
C	1.16032800	-0.89324200	-0.72768000
C	0.04649100	-1.56348600	-1.54723300
C	-1.10748600	-0.98872800	-0.70624200
C	-0.61546100	-1.35156600	0.69870300
C	0.72875900	-1.29392100	0.68273000
H	-1.81581100	3.34075500	0.19099800
H	-1.13327000	0.78315600	-1.96286800
H	1.13565800	0.99263300	-1.85757500
H	2.90184800	2.35138200	0.68854700
H	0.02480800	-1.24052000	-2.60145000
H	0.10015100	-2.65991800	-1.48745700
C	-2.51748800	-1.41601500	-1.06576400
H	-2.75301900	-1.14407600	-2.10628000
H	-2.62779100	-2.50618100	-0.96543400
H	-3.25180900	-0.93234200	-0.40593100
C	2.59653400	-1.18763500	-1.11180100
H	2.80921900	-2.26109000	-0.99752400
H	2.78348000	-0.90868700	-2.15973600
H	3.30217100	-0.62694300	-0.48142700
C	1.70117900	-1.44164500	1.80820000
H	1.19478700	-1.70682400	2.74523500
H	2.45449500	-2.21502800	1.59042700
H	2.24782700	-0.49940800	1.97926600
C	-1.53184500	-1.64409700	1.84208500
H	-0.97087300	-1.91272500	2.74684900
H	-2.15924900	-0.76805300	2.05857300
H	-2.20709700	-2.47943400	1.59738800

AU5

E (ωb97X-2 D3BJ/def2-TZVPP) = -3870.792392660635

C	3.95805800	4.63080500	-0.35183000
C	3.32386200	4.48021100	0.85593300

C	2.42741900	3.39767800	1.07754000
C	2.19997400	2.45239600	0.03140200
C	2.86711200	2.64517400	-1.21234100
C	3.72120900	3.70537800	-1.39887300
N	1.81725500	3.31816800	2.29114500
C	0.97397400	2.33621600	2.48568300
C	0.67611600	1.34641800	1.51854300
C	1.29809800	1.37197500	0.29120300
C	1.00034800	0.30648300	-0.75886800
N	-0.31863300	-0.25854200	-0.59950300
C	1.97067800	-0.90022900	-0.83969700
N	1.72661500	-1.89815900	0.21718800
C	2.52088000	-1.61503200	1.42388000
C	3.48045300	-0.60412100	-0.93893800
C	4.03263200	-1.81217600	1.15322900
C	4.22044300	-1.83004200	-0.37078000
C	3.57228500	-3.10728800	-0.93499100
C	4.33709200	-4.38633300	-0.73419900
C	5.56779800	-4.52118900	-0.23806600
H	1.66100200	-1.39760200	-1.77192600
C	-1.42675600	0.49540900	-0.85641900
O	-1.36017900	1.59491000	-1.37451100
N	-2.60596000	-0.12224000	-0.47423100
C	-3.90399200	0.37021100	-0.58260800
C	-4.22314100	1.57822900	-1.22973300
C	-5.54111300	2.01804900	-1.30541400
C	-6.58215500	1.27772800	-0.74848000
C	-6.28965700	0.07762500	-0.10596000
C	-4.97232200	-0.36272700	-0.02640300
Br	-4.63732900	-2.00710000	0.87365600
C	2.14520500	-3.20239600	-0.30601100
H	4.64260600	5.46513900	-0.51280900
H	3.47564700	5.18023500	1.67847200
H	2.70245800	1.94409900	-2.03049900
H	4.21744000	3.83801600	-2.36133000
H	0.47976400	2.29059800	3.46206000
H	-0.04804600	0.56597200	1.75717000
H	1.02916300	0.80457300	-1.73843300
H	-0.35289500	-1.12521800	-0.06753800
H	2.16789900	-2.27717300	2.22795900
H	2.31364400	-0.58656500	1.74066600
H	3.76371800	0.28114500	-0.34917300
H	3.76361400	-0.40794500	-1.98423000
H	4.39566400	-2.75399700	1.59014300

H	4.61474900	-0.99371000	1.60179400
H	5.28457100	-1.78333400	-0.64081900
H	3.45475200	-2.97593600	-2.02608700
H	3.79421600	-5.29139800	-1.03561800
H	6.02688500	-5.50667700	-0.13647300
H	6.16283000	-3.66140800	0.08055400
H	-2.52003000	-1.03078500	-0.03271600
H	-3.41741100	2.15992000	-1.66719200
H	-5.75062000	2.96016300	-1.81409300
H	-7.61376200	1.62512400	-0.80885500
H	-7.08038800	-0.52494300	0.34191000
H	2.14504500	-3.91557800	0.53277600
H	1.41348400	-3.55879400	-1.04659600

Complex **M-I**

E (ωB97X-2 D3BJ/def2-TZVPP) = -4676.479149140301

C	-5.92623500	-3.54853300	2.17864200
C	-5.15025000	-2.88971100	3.09879300
C	-4.01563000	-2.13537300	2.68745100
C	-3.69833500	-2.04565900	1.29716400
C	-4.51705900	-2.75651800	0.37228700
C	-5.59846700	-3.48723400	0.80128500
N	-3.27135000	-1.53698400	3.65608800
C	-2.20299500	-0.87773400	3.28538900
C	-1.79596200	-0.72391000	1.93830200
C	-2.55290100	-1.26998500	0.92602900
C	-2.16218500	-1.05152000	-0.52960200
N	-0.72452700	-1.07489200	-0.71723800
C	-2.77797400	0.18117900	-1.22462600
N	-2.51721100	1.49528000	-0.58567800
C	-3.55922700	1.83121900	0.41685300
C	-4.28553000	0.07446400	-1.52789400
C	-4.90815100	2.11717200	-0.27397700
C	-4.83657300	1.50265000	-1.67907500
C	-3.84442100	2.32067200	-2.52221700
C	-4.34055200	3.64141500	-3.04355300
C	-5.57430100	4.13626900	-2.93947200
H	-2.24012900	0.21798300	-2.18322000
C	-0.12578700	-2.27130700	-1.01465500
O	-0.76466900	-3.29093800	-1.20577700
N	1.25513500	-2.22088600	-1.07861100
C	2.05507100	-3.28826400	-1.50096000
C	1.61619400	-4.21273100	-2.46409200
C	2.43599400	-5.25454000	-2.88305500

C	3.72469200	-5.39946700	-2.36883500
C	4.18531500	-4.49249800	-1.41943600
C	3.35293200	-3.46105500	-0.99057100
Br	4.01064500	-2.28464700	0.34857700
C	-2.56811800	2.51651400	-1.65589100
H	-6.79100000	-4.12992700	2.50239200
H	-5.36407500	-2.92564100	4.16768000
H	-4.28149600	-2.73471900	-0.69162000
H	-6.20549000	-4.03148400	0.07633600
H	-1.60417600	-0.41506700	4.07714900
H	-0.89664600	-0.14773200	1.72090600
H	-2.52962000	-1.90927100	-1.10589200
H	-0.15942900	-0.25036600	-0.52395000
H	-3.19999000	2.69856400	0.98977300
H	-3.64327600	0.99324700	1.11837500
H	-4.82108900	-0.42904400	-0.70888100
H	-4.43623300	-0.52283800	-2.43947500
H	-5.09501800	3.19837000	-0.34230000
H	-5.73181700	1.67107500	0.30183100
H	-5.82673000	1.47889700	-2.15439900
H	-3.56677100	1.71670700	-3.40471000
H	-3.57873500	4.23908400	-3.55960400
H	-5.82247600	5.11233800	-3.36131100
H	-6.37949900	3.59543100	-2.43559200
H	1.70938300	-1.34013200	-0.83090300
H	0.61427400	-4.10180200	-2.87137500
H	2.06077700	-5.95716900	-3.62828500
H	4.36958900	-6.21463800	-2.69830000
H	5.18524700	-4.58518400	-0.99490000
H	-2.56762800	3.50278500	-1.16876900
H	-1.64791300	2.45723500	-2.25192800
C	0.39478400	2.87918700	0.36733400
O	-0.39967100	1.94014600	0.84488200
O	0.18373200	3.47253100	-0.66671600
C	1.54794000	3.17425000	1.30464800
C	2.36795800	1.94061600	1.77625200
C	2.00593300	0.62544800	1.14721400
O	1.90730400	-0.35733100	2.03628000
O	1.85147800	0.44194600	-0.04254700
C	3.85091000	2.37125300	1.44733900
C	3.74069900	3.85990600	1.82017000
C	2.63414200	4.17220600	0.79385500
C	3.26481100	3.56186500	-0.46087000
C	3.97907200	2.48986600	-0.07235700

H	-1.20543700	1.74656600	0.20158700
H	1.06462700	3.60637800	2.19735500
H	2.28258500	1.81247800	2.86267000
H	1.75282800	-1.19889400	1.56978900
H	3.43015300	4.02680100	2.86539100
H	4.66996400	4.40749900	1.60847200
C	2.16469600	5.61041900	0.68975700
H	1.77879600	5.96359100	1.65845200
H	2.99586900	6.26805700	0.39374300
H	1.36311200	5.70585600	-0.05611800
C	4.89345600	1.52575500	2.15171900
H	5.90405300	1.91283000	1.95497400
H	4.72890600	1.52585500	3.23956400
H	4.86121600	0.48052200	1.80422600
C	4.73578400	1.50069000	-0.89808600
H	4.69516600	1.75310200	-1.96566000
H	5.79440100	1.45725900	-0.59593000
H	4.32073700	0.48867000	-0.77156700
C	3.03952000	4.09788200	-1.83751000
H	3.60181400	3.52899900	-2.58974700
H	1.96982800	4.06076900	-2.08826200
H	3.35355600	5.15199000	-1.90153200

Complex **Water**

E (ωB97X-2 D3BJ/def2-TZVPP) = -76.311845188546

O	0.00000000	0.00000000	0.12019200
H	0.00000000	0.75387800	-0.48076700
H	0.00000000	-0.75387800	-0.48076700

Complex **3a-NBS**

E (ωB97X-2 D3BJ/def2-TZVPP) = -3889.862583874422

Br	2.77735400	-0.69630000	-1.77864500
C	-0.76648100	1.67380200	-0.83416300
O	0.32168000	1.50535800	-1.58017800
O	-0.85163800	2.50516700	0.03819700
C	-1.87551200	0.76192900	-1.29731400
C	-1.59972700	-0.76267000	-1.18966200
C	-0.43005400	-1.14964800	-0.32650300
O	0.02393000	-2.36694100	-0.58899100
O	0.03326100	-0.45241900	0.55498100
C	-2.96107100	-1.30484200	-0.57418400
C	-3.94444400	-0.31458100	-1.21797600
C	-3.27465600	0.94362500	-0.63371900
C	-3.16678200	0.52706100	0.83682800
C	-2.99611700	-0.80727300	0.86981200
H	1.05660500	2.06625700	-1.26633300

H	-1.97915400	0.99457600	-2.36975900
H	-1.45161700	-1.21792700	-2.17844700
H	0.81551700	-2.54466100	-0.04033700
H	-3.93837400	-0.34319100	-2.32030400
H	-4.96680300	-0.45690900	-0.84006400
C	-3.92881100	2.28276800	-0.91289100
H	-3.99481400	2.46443100	-1.99661700
H	-4.94783500	2.30920500	-0.49956300
H	-3.35152100	3.10117000	-0.45945700
C	-3.19302100	-2.78582900	-0.79540800
H	-4.16628000	-3.08562700	-0.37966900
H	-3.18778200	-3.02468500	-1.86953100
H	-2.41187200	-3.38932700	-0.31036100
C	-2.76166200	-1.71540000	2.03294600
H	-2.71901900	-1.16024200	2.97949700
H	-3.55995600	-2.47058200	2.11248300
H	-1.81400500	-2.26919700	1.93561700
C	-3.20235800	1.50141000	1.96871200
H	-3.09451700	0.99736900	2.93804000
H	-2.39296100	2.23724000	1.85925100
H	-4.15182400	2.06062500	1.97680100
N	2.89712800	-0.15166300	-0.03449600
C	2.89729300	1.18998500	0.34209700
O	2.84510100	2.11113800	-0.43569300
C	2.92553400	1.23142000	1.84884200
C	2.93244200	-0.22849300	2.29814800
C	2.74392600	-1.03148900	1.03475200
O	2.49600300	-2.20698400	0.91704700
H	2.03907000	1.80473400	2.15729100
H	3.80385000	1.80975900	2.16618400
H	3.88833800	-0.54066200	2.74425300
H	2.13129300	-0.49999100	2.99682900
O	0.40677600	-2.07085400	3.00185800
H	0.13716000	-1.40670400	2.34966200
H	1.10107400	-2.55500700	2.53377200
O	1.31669600	3.88112600	1.33984300
H	0.53170200	3.50222600	0.91194200
H	2.01097600	3.73389300	0.68446400

Complex **IM1a**

E (ωB97X-2 D3BJ/def2-TZVPP) = -8566.360743481371

C	-10.29145800	-1.80970000	-2.21634800
C	-9.23318900	-1.75020400	-3.08735700
C	-7.92706400	-1.42681700	-2.62488100

C	-7.71464100	-1.18805700	-1.23170700
C	-8.84094000	-1.23886300	-0.35976200
C	-10.09313000	-1.53992100	-0.83921300
N	-6.93325000	-1.34653000	-3.55022300
C	-5.73862200	-1.00986000	-3.13474900
C	-5.41474000	-0.76211500	-1.77949000
C	-6.38255800	-0.87977500	-0.80687400
C	-6.02159800	-0.67276000	0.65882200
N	-5.04239400	0.38358100	0.82215600
C	-5.59608500	-1.93361600	1.43935600
N	-4.45922500	-2.70428100	0.87433000
C	-4.92354100	-3.73766100	-0.08465000
C	-6.73802600	-2.91646300	1.76426700
C	-5.68545200	-4.86057100	0.64796600
C	-6.11331100	-4.30070200	2.01159000
C	-4.85838600	-4.12251000	2.88156500
C	-4.27952400	-5.37426000	3.48291100
C	-4.80015200	-6.60110400	3.44332500
H	-5.21979400	-1.52329800	2.38785300
C	-5.49607300	1.67365900	0.94596800
O	-6.66736500	1.93769100	1.15080800
N	-4.51149500	2.63523400	0.82285900
C	-4.72849600	4.00101000	1.02427900
C	-5.62897600	4.47084700	1.99563900
C	-5.81442800	5.83307600	2.19988800
C	-5.09785300	6.77010200	1.45456100
C	-4.19893500	6.33007400	0.48819000
C	-4.03223500	4.96353600	0.27440800
Br	-2.84434100	4.42108800	-1.10757700
C	-3.80006700	-3.39199000	2.00656400
H	-11.29065200	-2.05249200	-2.58132700
H	-9.35256200	-1.93368000	-4.15578100
H	-8.71714200	-1.02586500	0.70200200
H	-10.94379400	-1.56383000	-0.15658400
H	-4.95198600	-0.92403700	-3.89208500
H	-4.38508400	-0.51019900	-1.52527000
H	-6.91480300	-0.30792900	1.18048400
H	-4.06758300	0.21578700	0.58224700
H	-4.03756800	-4.11805700	-0.61383600
H	-5.56130500	-3.25187700	-0.83235000
H	-7.44726200	-2.99221800	0.92629300
H	-7.29676800	-2.55578500	2.64086200
H	-5.04936800	-5.74672400	0.78478000
H	-6.56343200	-5.16766700	0.06156300

H	-6.84016800	-4.96195600	2.50258100
H	-5.11896600	-3.45841400	3.72518100
H	-3.32474300	-5.22835400	4.00317900
H	-4.29210200	-7.43920900	3.92437800
H	-5.74597500	-6.81712400	2.93991300
H	-3.59107400	2.32940200	0.50545500
H	-6.18705200	3.74252100	2.57975100
H	-6.52276900	6.16625200	2.95959200
H	-5.23782500	7.83894000	1.61841300
H	-3.63058500	7.03870200	-0.11480800
H	-3.07644300	-4.10459600	1.58290100
H	-3.22335500	-2.65528000	2.58039600
C	-1.39898200	-1.60853700	0.00803500
O	-2.57366000	-1.69108300	-0.58415600
O	-1.19999200	-1.93926500	1.15578000
C	-0.32010200	-1.12489100	-0.94022000
C	-0.63447000	0.18753100	-1.71462400
C	-1.88932900	0.90511600	-1.30622600
O	-2.58916400	1.35079900	-2.34336900
O	-2.23151700	1.10002400	-0.15869500
C	0.66187100	1.05483400	-1.45579100
C	1.70078600	-0.07739200	-1.51189600
C	1.09992000	-0.87435100	-0.33852500
C	0.96244500	0.23819200	0.70401200
C	0.70224800	1.38566600	0.03772600
H	-3.32080100	-2.02874300	0.07253900
H	-0.23611400	-1.93092200	-1.68852800
H	-0.70368800	-0.00256900	-2.79290200
H	-3.35103000	1.86470600	-2.01783100
H	1.68445600	-0.63273400	-2.46510400
H	2.71803800	0.28603700	-1.31538700
C	1.83552700	-2.12156600	0.11036200
H	1.97288100	-2.81908600	-0.73194500
H	2.82629700	-1.85292300	0.50862500
H	1.27669400	-2.63979300	0.90241400
C	0.82422400	2.22183500	-2.40874400
H	1.78550600	2.72779400	-2.23177500
H	0.80290200	1.87987700	-3.45397100
H	0.02221500	2.96571000	-2.27648600
C	0.38709100	2.74398700	0.57616000
H	0.47322900	2.77457700	1.67053000
H	1.06510600	3.50379100	0.15583300
H	-0.63878600	3.04001200	0.30765200
C	1.03362300	-0.00360500	2.17795200

H	0.97520800	0.93457600	2.74619000
H	0.21166500	-0.66468900	2.48619900
H	1.97836200	-0.50553600	2.44323100
Br	3.63054200	1.70756900	0.93928200
C	6.00265500	-1.87965900	1.14422100
O	4.98180600	-1.17767700	1.63008700
O	7.04542700	-2.03117500	1.73368900
C	5.62836400	-2.51759500	-0.17113600
C	5.29099400	-1.54344200	-1.33199200
C	5.72628400	-0.11772600	-1.12648200
O	5.10860000	0.73408600	-1.93506300
O	6.58507000	0.23332700	-0.34255500
C	6.06611900	-2.19840400	-2.55473200
C	5.95268400	-3.68049400	-2.16637100
C	6.64521500	-3.51757600	-0.79945300
C	7.89126000	-2.73283000	-1.22467000
C	7.55067600	-1.96721100	-2.27729400
H	5.22478600	-0.70156000	2.44840900
H	4.70723300	-3.07864700	0.05854200
H	4.21495300	-1.54076200	-1.55812600
H	5.37048000	1.65016400	-1.70232100
H	4.91129500	-4.03583600	-2.09119600
H	6.52515400	-4.32356200	-2.84972800
C	6.88030400	-4.77023200	0.02197200
H	5.92815100	-5.28163100	0.23119200
H	7.53199800	-5.47029800	-0.52153800
H	7.35967300	-4.52467800	0.98033200
C	5.54426900	-1.77422800	-3.91250600
H	6.08649800	-2.29914800	-4.71288900
H	4.47373000	-2.01205400	-4.00722800
H	5.66628600	-0.69221700	-4.06587800
C	8.35204000	-0.95425400	-3.02871100
H	9.37988500	-0.88460200	-2.64927100
H	8.39367600	-1.20283200	-4.10100600
H	7.90817900	0.05181000	-2.95375300
C	9.19901200	-2.82010500	-0.50723100
H	9.96643200	-2.20433100	-0.99437700
H	9.08291300	-2.48522900	0.53390100
H	9.56158400	-3.85968800	-0.47680200
N	5.33821900	2.24600800	1.40204300
C	5.99373200	1.79085900	2.54090800
O	5.51757300	1.01535600	3.33627800
C	7.37228100	2.40359400	2.54814800
C	7.47467000	3.18105700	1.23727800

C	6.17451600	2.91411200	0.51770900
O	5.88117400	3.18982900	-0.62315600
H	8.08285100	1.57082200	2.65212600
H	7.46629800	3.02674000	3.44840200
H	7.56542500	4.26810700	1.37533200
H	8.29204500	2.85929200	0.57907400
O	8.43945800	2.10848700	-1.63564300
H	8.03438500	1.36210700	-1.16838000
H	7.71418800	2.74703800	-1.67616200
O	8.05183400	-0.38198800	3.87606100
H	7.76820900	-1.01486100	3.19632300
H	7.21506600	-0.01386500	4.18872100

Complex **TSa^{AU5}**

E (ωB97X-2 D3BJ/def2-TZVPP) = -8566.31619083566

C	-10.12578000	-2.57316600	-1.94422100
C	-9.14875600	-2.39903200	-2.89119700
C	-7.84565000	-1.96400100	-2.52091400
C	-7.54985400	-1.72683100	-1.14278200
C	-8.59404500	-1.90311400	-0.18887600
C	-9.84559300	-2.31435500	-0.57906500
N	-6.93518200	-1.78219400	-3.51519500
C	-5.74643700	-1.34822800	-3.18221600
C	-5.34589500	-1.08387800	-1.85063900
C	-6.22518700	-1.29662000	-0.81202000
C	-5.79267000	-1.05618200	0.62977800
N	-4.92087300	0.09522700	0.74852300
C	-5.20268700	-2.26695100	1.38281400
N	-3.97138400	-2.86025300	0.79439200
C	-4.29038000	-3.92735700	-0.19117800
C	-6.19092600	-3.40761900	1.68752700
C	-4.88607200	-5.15846800	0.51990400
C	-5.37536600	-4.69424300	1.89885200
C	-4.15158000	-4.36199800	2.76878000
C	-3.40565800	-5.53569200	3.34459600
C	-3.75285300	-6.82085200	3.27157500
H	-4.87556300	-1.83119800	2.33780700
C	-5.45176400	1.29355300	1.15817900
O	-6.58818100	1.40096800	1.58090100
N	-4.58347900	2.36071100	1.04549100
C	-4.91219600	3.66325600	1.43702300
C	-5.62070200	3.91668300	2.62243400
C	-5.92240000	5.21656600	3.01131200
C	-5.51160400	6.30276000	2.23747100

C	-4.80718000	6.07805600	1.05857000
C	-4.52781500	4.77113500	0.66618200
Br	-3.63182800	4.51846000	-0.99374300
C	-3.20772800	-3.47822900	1.90714100
H	-11.12356600	-2.90137200	-2.23916100
H	-9.33310500	-2.57645300	-3.95135200
H	-8.40671000	-1.70402100	0.86633300
H	-10.63101900	-2.43822800	0.16786000
H	-5.02646300	-1.19217200	-3.99290200
H	-4.32713100	-0.74154400	-1.66798800
H	-6.68368200	-0.78104900	1.20719800
H	-3.96780800	0.05420400	0.39529600
H	-3.36024400	-4.16586600	-0.72632200
H	-4.99167700	-3.51505100	-0.92475100
H	-6.89193700	-3.56348500	0.85322400
H	-6.78615100	-3.14886600	2.57574700
H	-4.13405500	-5.95217300	0.63231000
H	-5.71880200	-5.56720000	-0.06981600
H	-5.99663200	-5.46203700	2.37891300
H	-4.49589600	-3.75643000	3.62609900
H	-2.48591500	-5.27118900	3.88053900
H	-3.13742000	-7.59344800	3.73667600
H	-4.65628400	-7.15222500	2.75311900
H	-3.69575800	2.20910400	0.56826000
H	-5.93258400	3.06841500	3.22868200
H	-6.47436700	5.38210300	3.93728400
H	-5.74081300	7.32414700	2.54294200
H	-4.48617000	6.91025000	0.43158700
H	-2.40418800	-4.07975900	1.45632300
H	-2.72936000	-2.68123200	2.48997400
C	-1.23819900	-1.20783900	-0.01751800
O	-2.40622400	-1.42651600	-0.56492400
O	-0.94994200	-1.48547900	1.12698600
C	-0.23350600	-0.67166000	-1.02420200
C	-0.70072300	0.47769400	-1.96216800
C	-1.93906100	1.16792700	-1.44979200
O	-2.76792300	1.54602000	-2.40550100
O	-2.14538100	1.36745400	-0.27052800
C	0.59476800	1.39845700	-2.07879500
C	1.65764300	0.29495500	-1.93940900
C	1.18749100	-0.22862600	-0.56235700
C	0.97468400	1.05965300	0.24601200
C	0.68054500	2.11272700	-0.72851900
H	-3.09289800	-1.99359700	0.09937600

H	-0.05372200	-1.54981000	-1.66460200
H	-0.91326000	0.11418200	-2.97495800
H	-3.53648500	2.00266400	-2.01315000
H	1.53599400	-0.47484000	-2.71338900
H	2.69382900	0.64923700	-1.96422500
C	2.03994500	-1.28848100	0.10219900
H	2.16448900	-2.13998400	-0.58415500
H	3.03604700	-0.88870100	0.34641800
H	1.56955900	-1.65585200	1.02336800
C	0.62851200	2.29377500	-3.29768800
H	1.54592500	2.90085000	-3.29693100
H	0.61548900	1.68534100	-4.21253400
H	-0.23985600	2.96882500	-3.32136400
C	0.01142100	3.42796100	-0.47342100
H	0.02999500	3.69346300	0.59056400
H	0.51771200	4.21334900	-1.04989300
H	-1.03368800	3.39528000	-0.80309400
C	0.58778500	1.08473900	1.68496000
H	0.57758600	2.10751800	2.07831100
H	-0.41808800	0.64972500	1.76387200
H	1.27682800	0.46690000	2.27466200
Br	2.66170600	2.29156000	-0.03483500
C	6.08875700	-1.94965000	1.23450900
O	5.00799100	-1.29895700	1.62446300
O	7.08662400	-2.07873800	1.90669800
C	5.87919500	-2.55800600	-0.13524900
C	5.60209100	-1.52963300	-1.26935500
C	5.89903500	-0.09487300	-0.90647100
O	5.06181300	0.77067000	-1.45806500
O	6.83443800	0.24019000	-0.21174000
C	6.54715800	-2.04807700	-2.42912100
C	6.47375700	-3.56053600	-2.15988700
C	7.00651400	-3.46417200	-0.71661200
C	8.25026100	-2.60235600	-0.95623400
C	7.98045800	-1.77200900	-1.97973400
H	5.16766300	-0.72211400	2.43458400
H	4.97261000	-3.17674900	-0.02756400
H	4.56006900	-1.58473300	-1.61354900
H	5.24132000	1.68629800	-1.10203400
H	5.44964100	-3.96636000	-2.22170700
H	7.14657300	-4.12790000	-2.81868800
C	7.21829100	-4.75568300	0.04975800
H	6.28642100	-5.34060100	0.09592000
H	7.98763400	-5.37497900	-0.43523100

H	7.54325600	-4.54186700	1.07828000
C	6.15438500	-1.54548600	-3.80460100
H	6.82137300	-1.96402900	-4.57293400
H	5.12101500	-1.83730700	-4.04702400
H	6.21917600	-0.44802500	-3.86099400
C	8.80383000	-0.66507700	-2.55342800
H	9.78656600	-0.60290500	-2.06817000
H	8.96278000	-0.80638500	-3.63443400
H	8.30921100	0.31206400	-2.42784400
C	9.47750000	-2.67875100	-0.10685800
H	10.25752500	-1.99481100	-0.46595300
H	9.23126300	-2.41902900	0.93254500
H	9.89177000	-3.69942200	-0.10045200
N	5.06645200	1.77167000	1.61103700
C	5.77232800	1.44931400	2.71635200
O	5.51655800	0.48314800	3.44970900
C	6.98027500	2.35142200	2.92973000
C	6.94572800	3.25072300	1.70235500
C	5.73283500	2.72593900	0.94829600
O	5.41841400	3.11794000	-0.19623600
H	7.86209500	1.69902100	3.00552500
H	6.87969600	2.87919400	3.88883700
H	6.79395900	4.31732900	1.92299600
H	7.82538700	3.17130400	1.04911800
O	7.99497300	2.58769900	-1.38216700
H	7.17792900	3.08365100	-1.22219300
H	7.84065200	1.78080900	-0.86649800
O	8.10435800	-0.47452200	4.10429800
H	7.92738600	-1.07510400	3.36355200
H	7.21871700	-0.10835900	4.25039700

Complex **IM2a**

E (ωB97X-2 D3BJ/def2-TZVPP) = -8566.317400369979

C	-9.79660400	-3.37207400	-1.73126000
C	-8.90845500	-3.07230900	-2.73339000
C	-7.65399500	-2.47005500	-2.43717500
C	-7.30727800	-2.20065300	-1.07723600
C	-8.26118900	-2.50962500	-0.06472700
C	-9.47135700	-3.07698100	-0.38364000
N	-6.83861200	-2.16483200	-3.48241100
C	-5.70129900	-1.57413300	-3.21789500
C	-5.25611100	-1.26615500	-1.90989600
C	-6.03021900	-1.60506800	-0.82273600
C	-5.53665500	-1.33315100	0.59225800

N	-4.79939900	-0.08785200	0.68360400
C	-4.75892600	-2.47518700	1.27999800
N	-3.51132600	-2.91501200	0.59736400
C	-3.77520300	-4.00084300	-0.38406200
C	-5.58364300	-3.72704100	1.63250200
C	-4.16868600	-5.30150700	0.34356600
C	-4.61247200	-4.91305900	1.76084600
C	-3.37544900	-4.45269400	2.54929900
C	-2.45412000	-5.53827900	3.03741800
C	-2.66284000	-6.85364700	2.97911100
H	-4.41849600	-2.01482200	2.21863400
C	-5.43255100	1.02343300	1.17953300
O	-6.56862300	0.99229700	1.61627900
N	-4.67479900	2.17718400	1.13493600
C	-5.11607900	3.40350300	1.64619400
C	-5.84226600	3.48052400	2.84561800
C	-6.25580500	4.70587300	3.35451200
C	-5.94296000	5.89359900	2.69205800
C	-5.22316200	5.84455900	1.50225500
C	-4.83186800	4.61005900	0.98842800
Br	-3.91797100	4.59648900	-0.68057500
C	-2.60690900	-3.45178600	1.64359500
H	-10.75931300	-3.82810600	-1.96708300
H	-9.13065400	-3.27179300	-3.78236100
H	-8.04041800	-2.28598400	0.97908700
H	-10.19157500	-3.29420800	0.40644400
H	-5.06551900	-1.31598000	-4.07158800
H	-4.28460200	-0.78892300	-1.78152000
H	-6.41498300	-1.17445000	1.22934800
H	-3.84659800	-0.02697300	0.33330300
H	-2.86461100	-4.12263900	-0.98788400
H	-4.57122600	-3.66583600	-1.05861600
H	-6.32172600	-3.95430800	0.84854000
H	-6.13697000	-3.54928000	2.56642500
H	-3.32067800	-5.99933800	0.39098100
H	-4.98448400	-5.80406200	-0.19513900
H	-5.10908300	-5.75210800	2.26590100
H	-3.72256300	-3.90384900	3.44295800
H	-1.52043100	-5.17451200	3.48403700
H	-1.92506400	-7.55539600	3.37271900
H	-3.57090800	-7.28093200	2.54586500
H	-3.76559700	2.14167200	0.67481400
H	-6.08189100	2.55566100	3.36621700
H	-6.82329700	4.73131900	4.28577700

H	-6.26293300	6.85611400	3.09237100
H	-4.97418000	6.75588600	0.95813800
H	-1.76829900	-3.94352500	1.12889400
H	-2.18849100	-2.61222900	2.21228200
C	-1.02068800	-0.99899400	-0.39508400
O	-2.21908800	-1.28355500	-0.83881600
O	-0.58858100	-1.33974600	0.68443500
C	-0.18896800	-0.29246000	-1.45422200
C	-0.85760900	0.88649500	-2.21647900
C	-2.08583700	1.41353000	-1.51984900
O	-3.03212500	1.79631000	-2.35732600
O	-2.18734100	1.48871600	-0.31315100
C	0.33257200	1.92549600	-2.38856800
C	1.48444600	0.90959200	-2.50289900
C	1.25071100	0.22442300	-1.13712900
C	1.05985800	1.40268100	-0.18350900
C	0.55471000	2.51998400	-0.99167800
H	-2.78083600	-1.93898800	-0.14987100
H	-0.02927300	-1.09497900	-2.19193000
H	-1.15417500	0.59173400	-3.23035100
H	-3.79164300	2.15730500	-1.86134900
H	1.31874200	0.20571400	-3.32902800
H	2.47690600	1.35551500	-2.62224200
C	2.25255400	-0.81058800	-0.67890600
H	2.39267000	-1.57426800	-1.45900600
H	3.21809500	-0.32903000	-0.47409100
H	1.91595800	-1.30702500	0.23971900
C	0.13926000	2.93462800	-3.49947400
H	1.00966500	3.60498400	-3.55528700
H	0.03294900	2.42095600	-4.46474100
H	-0.76218200	3.54277200	-3.33302900
C	-0.19049700	3.72333100	-0.49640700
H	0.01894400	3.91353600	0.56314200
H	0.10544500	4.60293900	-1.08303100
H	-1.27087500	3.58637100	-0.61393100
C	0.97696100	1.25332900	1.28921800
H	0.78887100	2.21033000	1.78894900
H	0.16258700	0.54300300	1.49550500
H	1.91643800	0.81539300	1.66100100
Br	2.57278900	2.87448400	-0.59839100
C	5.75649600	-2.14976300	1.01799800
O	4.55779700	-1.62492800	1.19855800
O	6.55964300	-2.34448200	1.90178200
C	5.94332900	-2.52482000	-0.43551900

C	5.87929000	-1.32765700	-1.42783200
C	5.96626600	0.03378700	-0.77940700
O	5.23192800	0.95629400	-1.37950300
O	6.66869000	0.26744400	0.18200000
C	7.11793400	-1.61237200	-2.37039500
C	7.09170900	-3.15073600	-2.36903300
C	7.24219900	-3.28464900	-0.84113900
C	8.43972500	-2.35689500	-0.61574900
C	8.37111800	-1.37275900	-1.53063200
H	4.43158500	-1.18256600	2.08598000
H	5.08432500	-3.17832800	-0.66104900
H	4.96317000	-1.35834700	-2.03283300
H	5.27032900	1.80696200	-0.85197400
H	6.14725600	-3.57222800	-2.75357100
H	7.94667500	-3.57497000	-2.91462600
C	7.34754700	-4.68045800	-0.25774800
H	6.46576400	-5.28256700	-0.52639200
H	8.24306700	-5.19514600	-0.63709700
H	7.41291900	-4.63501400	0.83900600
C	7.04383800	-0.89504900	-3.70358200
H	7.90915700	-1.15527000	-4.33135400
H	6.12770700	-1.17234300	-4.24687800
H	7.04022200	0.19714900	-3.56653900
C	9.22935100	-0.16031300	-1.69222800
H	10.07838700	-0.17339400	-0.99607500
H	9.62799700	-0.08773900	-2.71661600
H	8.66099700	0.76488000	-1.50189500
C	9.42372800	-2.54074100	0.49410900
H	10.23491200	-1.80333300	0.43579300
H	8.91956900	-2.43672100	1.46593700
H	9.87124300	-3.54682600	0.46201600
N	4.35746900	1.47217800	1.60785400
C	4.67957400	0.98935400	2.82870100
O	4.23124200	-0.06710200	3.29722200
C	5.71370600	1.84199600	3.55079200
C	6.03436800	2.91660700	2.52262000
C	5.15791500	2.51368200	1.34656100
O	5.22670800	3.07983100	0.23178100
H	6.55322400	1.18324100	3.81566500
H	5.28544300	2.21823500	4.49083400
H	5.77190200	3.93851800	2.83250900
H	7.07902600	2.93244700	2.18310000
O	8.06882800	2.74913400	-0.07437500
H	7.20806700	3.19053700	-0.13667900

H	7.80378300	1.84902100	0.17149800
O	6.54193200	-1.14303100	4.55122700
H	6.66826500	-1.59869100	3.70374700
H	5.63444200	-0.81611300	4.45579000

Complex **IM3a**

E (ωB97X-2 D3BJ/def2-TZVPP) = -8566.34042527173

C	4.86703000	4.44105600	3.14364800
C	5.03720900	3.19675700	2.58998700
C	3.98517400	2.57150300	1.86494100
C	2.74013900	3.25475900	1.70296200
C	2.59759900	4.54322200	2.29646300
C	3.63149900	5.11770700	2.99752300
N	4.21673000	1.32732700	1.36351900
C	3.24103100	0.72804300	0.72459600
C	1.96419000	1.31293900	0.51412000
C	1.70175000	2.58306700	0.97610400
C	0.35775200	3.23840300	0.72531800
N	-0.70489600	2.25978200	0.86500000
C	0.20832700	3.99041300	-0.61345400
N	0.40820800	3.17500300	-1.83655900
C	1.82005600	3.18690200	-2.28952400
C	1.02160300	5.29037000	-0.73762600
C	2.22804100	4.59204100	-2.78281200
C	1.19414500	5.58573400	-2.23747300
C	-0.14930200	5.33269200	-2.94329300
C	-0.27103600	5.85734600	-4.34793100
C	0.61043900	6.60999400	-5.00698600
H	-0.85716000	4.25733500	-0.61322000
C	-1.92281700	2.68291800	1.31739100
O	-2.29937700	3.84066200	1.23351500
N	-2.67032300	1.66933100	1.88727400
C	-4.05179100	1.60156000	1.97585700
C	-4.91362000	2.52146000	1.35087800
C	-6.29288800	2.35035000	1.40185200
C	-6.85897600	1.26404300	2.07104900
C	-6.02464000	0.35582700	2.71923900
C	-4.64372500	0.53316600	2.67897000
Br	-3.54103500	-0.68625600	3.63386900
C	-0.40739000	3.79875600	-2.90267100
H	5.67943900	4.91267100	3.69857400
H	5.97257100	2.64375100	2.68459100
H	1.65799800	5.08731100	2.19914400
H	3.49747900	6.10291400	3.44650000
H	3.43957200	-0.27866900	0.34359800

H	1.22463900	0.72497600	-0.03611700
H	0.18595200	4.00874800	1.49238700
H	-0.44782100	1.29178700	1.04142900
H	1.91676200	2.43212300	-3.08239900
H	2.45273700	2.85969700	-1.45714500
H	2.01619700	5.18909300	-0.27302700
H	0.49461100	6.10517000	-0.21921700
H	2.25331600	4.63403400	-3.88131300
H	3.23437700	4.84316500	-2.41781100
H	1.52244300	6.62332400	-2.38799800
H	-0.94327400	5.82280800	-2.35218600
H	-1.19835900	5.57138000	-4.86007100
H	0.41166100	6.93756200	-6.02953000
H	1.55446000	6.92952300	-4.55810300
H	-2.19237500	0.77481300	1.96472600
H	-4.47447900	3.36748700	0.82702600
H	-6.93438700	3.07918300	0.90382800
H	-7.94058100	1.13144100	2.10820300
H	-6.43869700	-0.48787800	3.27293700
H	-0.11578000	3.33000600	-3.85581000
H	-1.46264400	3.55958200	-2.71949000
C	-1.47630200	0.44843100	-2.20312200
O	-0.22149800	0.66916400	-1.93080400
O	-2.31117100	1.31492100	-2.37145300
C	-1.76821000	-1.02955100	-2.44460700
C	-1.42687500	-2.06116300	-1.33453100
C	-1.31214500	-1.42943900	0.03560200
O	-0.75164900	-2.14903800	0.95936400
O	-1.81701500	-0.34064300	0.24348500
C	-2.61664100	-3.12748900	-1.44096800
C	-2.94401400	-2.96509100	-2.93295400
C	-3.20757900	-1.44161900	-2.89941100
C	-4.09642000	-1.27143200	-1.65876900
C	-3.75359600	-2.39169800	-0.76795900
H	-0.02480600	1.71099900	-1.80461300
H	-1.13315500	-1.26741200	-3.31189600
H	-0.50555800	-2.61649000	-1.54837400
H	-0.47593800	-1.49139400	1.72197700
H	-2.07588400	-3.20602300	-3.55979300
H	-3.79290500	-3.57362800	-3.26238700
C	-3.71623800	-0.79505600	-4.17066800
H	-3.01249900	-0.99633200	-4.99109400
H	-4.69655600	-1.21212900	-4.44528100
H	-3.80200200	0.29157700	-4.04885400

C	-2.27899400	-4.49619600	-0.89308400
H	-3.15837300	-5.15583800	-0.93188300
H	-1.47561000	-4.94689900	-1.49171700
H	-1.92697600	-4.42612500	0.14615000
C	-4.05080000	-2.51159800	0.68161700
H	-4.79360800	-1.78055800	1.02258700
H	-4.39324700	-3.53249600	0.90012600
H	-3.12031100	-2.37202100	1.25188400
C	-4.66746100	0.01747200	-1.17958900
H	-5.36463900	-0.12958600	-0.34368400
H	-3.81731200	0.62790000	-0.83881000
H	-5.16237200	0.55177400	-1.99971600
Br	-5.68999300	-2.69505500	-1.78285000
C	4.51042100	-3.34888200	0.43367400
O	4.63065200	-4.39464200	1.23951800
O	4.52343500	-2.20839200	0.83601100
C	4.29884200	-3.73507100	-1.01489300
C	2.89618500	-3.30296700	-1.55856600
C	2.02703200	-2.63716300	-0.51371300
O	1.80784000	-3.44108900	0.50722100
O	1.57696400	-1.51585300	-0.60650200
C	3.23093200	-2.46694600	-2.83210200
C	4.44662300	-3.27534200	-3.32609600
C	5.27153300	-3.06037400	-2.04025200
C	5.14499500	-1.54090800	-1.90093100
C	3.94425900	-1.18532500	-2.39103000
H	4.53655100	-4.06765600	2.16196300
H	4.41002600	-4.82479800	-1.08482100
H	2.35241200	-4.21486600	-1.85647900
H	1.53394600	-2.92242200	1.35070700
H	4.21945500	-4.33677700	-3.52457900
H	4.92539500	-2.81919200	-4.20451300
C	6.67978300	-3.62236400	-1.99926400
H	6.67145400	-4.70875000	-2.17390700
H	7.30937600	-3.15355300	-2.77005400
H	7.15181100	-3.44051500	-1.02104600
C	2.04643600	-2.28254700	-3.76183900
H	2.33739100	-1.73465400	-4.67060600
H	1.62869900	-3.25533800	-4.06624700
H	1.25939200	-1.70438100	-3.25152700
C	3.34320600	0.17555300	-2.52085000
H	4.01242100	0.94878600	-2.11748200
H	3.15114900	0.41425800	-3.58108000
H	2.37948200	0.23094300	-1.99295600

C	6.20348800	-0.68916000	-1.27847800
H	5.87780800	0.35612600	-1.19014100
H	6.43728200	-1.04858600	-0.26540900
H	7.13561900	-0.71362700	-1.86609200
N	1.73509100	-2.02400000	2.70153000
C	1.25344600	-0.80004400	2.93259700
O	0.14541700	-0.39003600	2.51423300
C	2.16848600	0.04026500	3.80757700
C	3.39683400	-0.84750700	3.94669400
C	2.93190800	-2.16865800	3.35792300
O	3.54514500	-3.22261000	3.46147100
H	1.65384700	0.24729300	4.75746300
H	2.35898600	1.00605300	3.31971300
H	4.23155000	-0.49664200	3.31858600
H	3.76581300	-0.98907700	4.96983300
O	2.00579300	-5.46687800	2.50980300
H	2.46700100	-4.76584100	2.99626100
H	2.17746600	-5.20976600	1.59556400
O	-0.00035400	2.33140900	3.77248100
H	0.89383400	2.65139200	3.60439600
H	-0.02981400	1.46596200	3.33372100

Complex **+(-)6a +AU5**

E (ωB97X-2 D3BJ/def2-TZVPP) = -8566.41920676844

C	4.73165400	4.06739300	3.53677700
C	4.88963000	2.84556700	2.93173500
C	3.84383600	2.29219100	2.14309900
C	2.61523800	3.00394400	1.99304400
C	2.48883500	4.26930300	2.63468800
C	3.52055100	4.78665600	3.38118200
N	4.05708600	1.07842600	1.56719400
C	3.10228800	0.54744600	0.84137900
C	1.84233700	1.16248300	0.64603500
C	1.57947200	2.38527500	1.22424500
C	0.19889100	3.01174900	1.09779300
N	-0.80474700	1.98442400	1.23778100
C	-0.07846700	3.88857300	-0.14285100
N	-0.15854500	3.14052000	-1.43750700
C	1.16973600	2.99373100	-2.10424600
C	0.84877500	5.09238800	-0.36350400
C	1.64225700	4.36821400	-2.60638600
C	0.82879900	5.43826000	-1.86407400
C	-0.62661200	5.38367300	-2.36397800
C	-0.88567300	5.99478000	-3.71559100

C	-0.02623900	6.69289200	-4.45724700
H	-1.10203100	4.24963800	0.03475600
C	-2.02201700	2.25443100	1.78992000
O	-2.37692400	3.37159300	2.12613900
N	-2.78454200	1.11478500	1.94432400
C	-4.13143500	1.05418500	2.28965700
C	-5.03565400	2.07367800	1.95206700
C	-6.38825600	1.94903900	2.24813500
C	-6.87716700	0.80217600	2.87573200
C	-5.99402200	-0.21346200	3.23265200
C	-4.63545700	-0.07683700	2.95468400
Br	-3.45023700	-1.43653700	3.55776500
C	-1.06285400	3.89558400	-2.34978700
H	5.53735600	4.48932000	4.13924400
H	5.80805500	2.26425700	3.03050700
H	1.56691300	4.84235000	2.53472800
H	3.40364900	5.75837700	3.86295600
H	3.31956500	-0.42869300	0.39685300
H	1.10802400	0.63870200	0.02449400
H	0.04524900	3.70128400	1.93912500
H	-0.69346700	1.11833300	0.71518900
H	1.02415100	2.25747100	-2.90618700
H	1.86297500	2.57304300	-1.37000500
H	1.88107500	4.85649600	-0.05741700
H	0.50354000	5.93654700	0.25046200
H	1.49367300	4.46259000	-3.69099200
H	2.71589500	4.48804400	-2.40516200
H	1.25300400	6.43819600	-2.02283200
H	-1.25593600	5.93109600	-1.64037000
H	-1.90001100	5.83568600	-4.10089700
H	-0.32953200	7.09882100	-5.42423700
H	1.00074600	6.88748000	-4.13797900
H	-2.32980500	0.22007700	1.79278000
H	-4.65179100	2.96255800	1.45441300
H	-7.06944900	2.75539200	1.97299700
H	-7.93977600	0.69873200	3.09728700
H	-6.34825200	-1.11010500	3.74227400
H	-0.97813800	3.41803100	-3.33498700
H	-2.09270500	3.76402600	-1.99032200
C	-1.12073900	0.26059000	-2.44025100
O	-1.02162600	0.75181400	-1.26304000
O	-0.90480700	0.88065000	-3.47970800
C	-1.58166000	-1.19400500	-2.55470600
C	-1.35313100	-2.17138600	-1.36732700

C	-1.45265800	-1.50286800	-0.03012700
O	-0.56551900	-1.15000300	0.72912000
O	-2.72249700	-1.37238100	0.30133200
C	-2.63199900	-3.05074500	-1.39472500
C	-3.09343800	-2.96468700	-2.85120300
C	-3.08244400	-1.42872100	-2.95172800
C	-4.02826300	-1.05848300	-1.76340200
C	-3.59321400	-2.07349000	-0.64219000
H	-0.57859700	2.09805500	-1.32008500
H	-1.01293100	-1.59237300	-3.40576400
H	-0.42148300	-2.73857000	-1.44993800
H	-0.20462300	-0.67057200	2.30354800
H	-2.36922500	-3.42743100	-3.53744300
H	-4.08238500	-3.41159600	-3.01889600
C	-3.41280100	-0.80520500	-4.29544200
H	-2.65720200	-1.10746200	-5.03462200
H	-4.40048100	-1.12291000	-4.65193100
H	-3.38538600	0.29182700	-4.23594800
C	-2.45810300	-4.42352600	-0.77737600
H	-3.41201400	-4.96794100	-0.73886800
H	-1.75682700	-5.01035600	-1.38869800
H	-2.03510600	-4.35439600	0.23600700
C	-4.67069000	-2.70139000	0.21251000
H	-5.32877200	-1.92523400	0.63149700
H	-5.27890500	-3.39845600	-0.37470400
H	-4.18964400	-3.24337800	1.03808600
C	-4.10519300	0.38766200	-1.30765300
H	-4.86186100	0.49019900	-0.51600600
H	-3.14102500	0.72473300	-0.91271000
H	-4.39604300	1.03082300	-2.14987500
Br	-5.90974900	-1.48388400	-2.33573900
C	4.21364700	-3.21214500	0.05366600
O	3.81014700	-4.30922700	0.65751800
O	4.54203100	-2.20783700	0.65243400
C	4.23715100	-3.32911400	-1.45362800
C	3.03889600	-2.66963000	-2.21473500
C	2.02481000	-1.90117500	-1.39859700
O	1.71976900	-2.49919200	-0.25357800
O	1.47768000	-0.88580100	-1.76373800
C	3.74172700	-1.81421200	-3.32238500
C	4.96519800	-2.70702600	-3.59510600
C	5.48008300	-2.67449800	-2.14425000
C	5.45514800	-1.16071100	-1.89435600
C	4.43030000	-0.64963600	-2.60215900

H	3.68267700	-4.10493900	1.61984600
H	4.24383000	-4.40320900	-1.68175600
H	2.44772700	-3.47201100	-2.69043200
H	1.04241900	-1.97777900	0.23375900
H	4.70037900	-3.72053300	-3.94150500
H	5.66809500	-2.23882800	-4.29888300
C	6.78592500	-3.38127800	-1.83729500
H	6.72374100	-4.44860600	-2.09789000
H	7.61328100	-2.93462600	-2.40847800
H	7.03532500	-3.30246600	-0.76752400
C	2.86536300	-1.46268100	-4.51066700
H	3.45386900	-0.93666100	-5.27744600
H	2.45327700	-2.37591400	-4.96791000
H	2.02691900	-0.81786800	-4.21329000
C	3.96002600	0.76601500	-2.70040200
H	4.57250100	1.44029500	-2.08580600
H	3.99234900	1.12198100	-3.74329600
H	2.91416500	0.83673100	-2.37032600
C	6.44727300	-0.44929300	-1.03229500
H	6.27091500	0.63509300	-1.03512900
H	6.40103300	-0.79346000	0.01011300
H	7.47298900	-0.62532000	-1.39465000
N	1.71771600	-1.98338000	2.79065400
C	1.35974500	-0.86001900	3.33540700
O	0.24995800	-0.24192800	3.06983500
C	2.29847800	-0.26830400	4.34616300
C	3.43672900	-1.28022200	4.32593000
C	2.93470600	-2.35609100	3.37544700
O	3.50505400	-3.40185700	3.15694400
H	1.78140700	-0.18281000	5.31284200
H	2.57838700	0.74897400	4.03281900
H	4.37629500	-0.88829700	3.90454000
H	3.66754800	-1.72967900	5.30040800
O	6.12570400	-0.70081900	2.45685300
H	5.50982700	-0.03529400	2.08944200
H	5.75907600	-1.51430900	2.08026000
O	-0.59785000	-3.71016100	1.96593500
H	0.24855000	-3.25829400	2.13646000
H	-1.25123000	-3.08463800	2.30346100

Complex **IM1b**

E (ωB97X-2 D3BJ/def2-TZVPP) = -8566.35956381131

C	4.73165400	4.06739300	3.53677700
C	4.88963000	2.84556700	2.93173500

C	3.84383600	2.29219100	2.14309900
C	2.61523800	3.00394400	1.99304400
C	2.48883500	4.26930300	2.63468800
C	3.52055100	4.78665600	3.38118200
N	4.05708600	1.07842600	1.56719400
C	3.10228800	0.54744600	0.84137900
C	1.84233700	1.16248300	0.64603500
C	1.57947200	2.38527500	1.22424500
C	0.19889100	3.01174900	1.09779300
N	-0.80474700	1.98442400	1.23778100
C	-0.07846700	3.88857300	-0.14285100
N	-0.15854500	3.14052000	-1.43750700
C	1.16973600	2.99373100	-2.10424600
C	0.84877500	5.09238800	-0.36350400
C	1.64225700	4.36821400	-2.60638600
C	0.82879900	5.43826000	-1.86407400
C	-0.62661200	5.38367300	-2.36397800
C	-0.88567300	5.99478000	-3.71559100
C	-0.02623900	6.69289200	-4.45724700
H	-1.10203100	4.24963800	0.03475600
C	-2.02201700	2.25443100	1.78992000
O	-2.37692400	3.37159300	2.12613900
N	-2.78454200	1.11478500	1.94432400
C	-4.13143500	1.05418500	2.28965700
C	-5.03565400	2.07367800	1.95206700
C	-6.38825600	1.94903900	2.24813500
C	-6.87716700	0.80217600	2.87573200
C	-5.99402200	-0.21346200	3.23265200
C	-4.63545700	-0.07683700	2.95468400
Br	-3.45023700	-1.43653700	3.55776500
C	-1.06285400	3.89558400	-2.34978700
H	5.53735600	4.48932000	4.13924400
H	5.80805500	2.26425700	3.03050700
H	1.56691300	4.84235000	2.53472800
H	3.40364900	5.75837700	3.86295600
H	3.31956500	-0.42869300	0.39685300
H	1.10802400	0.63870200	0.02449400
H	0.04524900	3.70128400	1.93912500
H	-0.69346700	1.11833300	0.71518900
H	1.02415100	2.25747100	-2.90618700
H	1.86297500	2.57304300	-1.37000500
H	1.88107500	4.85649600	-0.05741700
H	0.50354000	5.93654700	0.25046200
H	1.49367300	4.46259000	-3.69099200

H	2.71589500	4.48804400	-2.40516200
H	1.25300400	6.43819600	-2.02283200
H	-1.25593600	5.93109600	-1.64037000
H	-1.90001100	5.83568600	-4.10089700
H	-0.32953200	7.09882100	-5.42423700
H	1.00074600	6.88748000	-4.13797900
H	-2.32980500	0.22007700	1.79278000
H	-4.65179100	2.96255800	1.45441300
H	-7.06944900	2.75539200	1.97299700
H	-7.93977600	0.69873200	3.09728700
H	-6.34825200	-1.11010500	3.74227400
H	-0.97813800	3.41803100	-3.33498700
H	-2.09270500	3.76402600	-1.99032200
C	-1.12073900	0.26059000	-2.44025100
O	-1.02162600	0.75181400	-1.26304000
O	-0.90480700	0.88065000	-3.47970800
C	-1.58166000	-1.19400500	-2.55470600
C	-1.35313100	-2.17138600	-1.36732700
C	-1.45265800	-1.50286800	-0.03012700
O	-0.56551900	-1.15000300	0.72912000
O	-2.72249700	-1.37238100	0.30133200
C	-2.63199900	-3.05074500	-1.39472500
C	-3.09343800	-2.96468700	-2.85120300
C	-3.08244400	-1.42872100	-2.95172800
C	-4.02826300	-1.05848300	-1.76340200
C	-3.59321400	-2.07349000	-0.64219000
H	-0.57859700	2.09805500	-1.32008500
H	-1.01293100	-1.59237300	-3.40576400
H	-0.42148300	-2.73857000	-1.44993800
H	-0.20462300	-0.67057200	2.30354800
H	-2.36922500	-3.42743100	-3.53744300
H	-4.08238500	-3.41159600	-3.01889600
C	-3.41280100	-0.80520500	-4.29544200
H	-2.65720200	-1.10746200	-5.03462200
H	-4.40048100	-1.12291000	-4.65193100
H	-3.38538600	0.29182700	-4.23594800
C	-2.45810300	-4.42352600	-0.77737600
H	-3.41201400	-4.96794100	-0.73886800
H	-1.75682700	-5.01035600	-1.38869800
H	-2.03510600	-4.35439600	0.23600700
C	-4.67069000	-2.70139000	0.21251000
H	-5.32877200	-1.92523400	0.63149700
H	-5.27890500	-3.39845600	-0.37470400
H	-4.18964400	-3.24337800	1.03808600

C	-4.10519300	0.38766200	-1.30765300
H	-4.86186100	0.49019900	-0.51600600
H	-3.14102500	0.72473300	-0.91271000
H	-4.39604300	1.03082300	-2.14987500
Br	-5.90974900	-1.48388400	-2.33573900
C	4.21364700	-3.21214500	0.05366600
O	3.81014700	-4.30922700	0.65751800
O	4.54203100	-2.20783700	0.65243400
C	4.23715100	-3.32911400	-1.45362800
C	3.03889600	-2.66963000	-2.21473500
C	2.02481000	-1.90117500	-1.39859700
O	1.71976900	-2.49919200	-0.25357800
O	1.47768000	-0.88580100	-1.76373800
C	3.74172700	-1.81421200	-3.32238500
C	4.96519800	-2.70702600	-3.59510600
C	5.48008300	-2.67449800	-2.14425000
C	5.45514800	-1.16071100	-1.89435600
C	4.43030000	-0.64963600	-2.60215900
H	3.68267700	-4.10493900	1.61984600
H	4.24383000	-4.40320900	-1.68175600
H	2.44772700	-3.47201100	-2.69043200
H	1.04241900	-1.97777900	0.23375900
H	4.70037900	-3.72053300	-3.94150500
H	5.66809500	-2.23882800	-4.29888300
C	6.78592500	-3.38127800	-1.83729500
H	6.72374100	-4.44860600	-2.09789000
H	7.61328100	-2.93462600	-2.40847800
H	7.03532500	-3.30246600	-0.76752400
C	2.86536300	-1.46268100	-4.51066700
H	3.45386900	-0.93666100	-5.27744600
H	2.45327700	-2.37591400	-4.96791000
H	2.02691900	-0.81786800	-4.21329000
C	3.96002600	0.76601500	-2.70040200
H	4.57250100	1.44029500	-2.08580600
H	3.99234900	1.12198100	-3.74329600
H	2.91416500	0.83673100	-2.37032600
C	6.44727300	-0.44929300	-1.03229500
H	6.27091500	0.63509300	-1.03512900
H	6.40103300	-0.79346000	0.01011300
H	7.47298900	-0.62532000	-1.39465000
N	1.71771600	-1.98338000	2.79065400
C	1.35974500	-0.86001900	3.33540700
O	0.24995800	-0.24192800	3.06983500
C	2.29847800	-0.26830400	4.34616300

C	3.43672900	-1.28022200	4.32593000
C	2.93470600	-2.35609100	3.37544700
O	3.50505400	-3.40185700	3.15694400
H	1.78140700	-0.18281000	5.31284200
H	2.57838700	0.74897400	4.03281900
H	4.37629500	-0.88829700	3.90454000
H	3.66754800	-1.72967900	5.30040800
O	6.12570400	-0.70081900	2.45685300
H	5.50982700	-0.03529400	2.08944200
H	5.75907600	-1.51430900	2.08026000
O	-0.59785000	-3.71016100	1.96593500
H	0.24855000	-3.25829400	2.13646000
H	-1.25123000	-3.08463800	2.30346100

Complex **TSb**$^{\mathbf{AU5}}$

E (ωB97X-2 D3BJ/def2-TZVPP) = -8566.31466339584

C	-9.55055800	2.50288200	-2.91103400
C	-8.49236700	2.13725200	-3.70398000
C	-7.34295000	1.51353600	-3.14397600
C	-7.29640700	1.25368600	-1.73980300
C	-8.41089800	1.65353900	-0.94665100
C	-9.50468100	2.26086700	-1.51571100
N	-6.32640500	1.19634900	-3.99087200
C	-5.25703400	0.64509300	-3.47701500
C	-5.10246700	0.34696000	-2.10152200
C	-6.12051600	0.62173800	-1.21992200
C	-5.96201500	0.32513900	0.27102900
N	-4.62002400	0.59771600	0.74528600
C	-6.44305000	-1.04465200	0.79966300
N	-5.58163800	-2.22599800	0.52226000
C	-5.87126800	-2.80985800	-0.80876300
C	-7.89463800	-1.38451200	0.39820700
C	-7.27827500	-3.44580200	-0.82926000
C	-8.05064500	-2.91192500	0.38541900
C	-7.42298800	-3.47047700	1.67094700
C	-7.73214000	-4.90557300	2.00179100
C	-8.58571300	-5.70969700	1.36787200
H	-6.39745500	-0.92655200	1.89346000
C	-4.21645500	1.90501200	0.82972800
O	-4.89252800	2.82087000	0.40113500
N	-2.99839400	2.07380900	1.45719100
C	-2.23303000	3.23657000	1.44883000
C	-2.28136800	4.15828000	0.38948900
C	-1.44428400	5.26855500	0.37499500

C	-0.53032400	5.48806800	1.40682000
C	-0.47289400	4.59394300	2.47275600
C	-1.32669300	3.49399700	2.49204100
C	-5.89100000	-3.24653700	1.55086100
H	-10.42617600	2.98352600	-3.34985300
H	-8.48767800	2.31330500	-4.78036000
H	-8.40825400	1.47815800	0.12878100
H	-10.34509700	2.56025200	-0.88763300
H	-4.44147300	0.40391000	-4.16769800
H	-4.18084700	-0.11714400	-1.75352200
H	-6.61453600	1.04446600	0.78676500
H	-3.99553500	-0.15830600	1.01030700
H	-5.08587900	-3.54998100	-1.02128200
H	-5.76819200	-2.01335600	-1.55334800
H	-8.12219600	-0.99947400	-0.60654100
H	-8.59725600	-0.91283400	1.10108900
H	-7.21392600	-4.54232100	-0.78422000
H	-7.80190400	-3.18192300	-1.75902500
H	-9.11448800	-3.17907900	0.32600300
H	-7.78932800	-2.86346400	2.51828900
H	-7.18279300	-5.30219800	2.86475900
H	-8.73734200	-6.73790500	1.70235100
H	-9.16498000	-5.38022200	0.50145700
H	-2.59802400	1.26762500	1.92945600
H	-2.99355400	3.98603200	-0.41583200
H	-1.50203900	5.96562300	-0.46207400
H	0.13119600	6.35454400	1.39057500
H	0.22639400	4.74859900	3.29498000
H	-5.38392800	-4.17455900	1.24501700
H	-5.45336600	-2.92625100	2.50812000
C	-0.85971600	-2.39269200	-0.17974500
C	-0.12861600	-2.08081300	1.15578100
C	1.24798800	-1.41908900	0.67830000
C	0.82928400	-0.02024500	0.23932500
C	-0.00240300	-0.20789900	-0.95742100
C	0.04406500	-1.72152500	-1.25944500
C	1.39960800	-2.15829000	-0.65827300
C	-1.13923400	0.69814400	-1.32055200
C	0.84960600	1.14792100	1.17286200
C	-0.21065800	-2.11337800	-2.70040100
C	2.37581600	-1.48129400	1.68215700
H	2.29019100	-1.85806400	-1.22321100
H	1.43147900	-3.24652500	-0.51186900
H	-0.88135300	1.74893400	-1.13503600

H	-1.42085600	0.56484200	-2.37245700
H	-2.00164100	0.43633900	-0.69096600
H	1.89484800	1.42950800	1.36760900
H	0.31033400	2.01328700	0.76519500
H	0.41016200	0.86959800	2.14085900
H	0.55803900	-1.66922700	-3.35004900
H	-0.15214000	-3.20686800	-2.80100300
H	-1.20266700	-1.78905100	-3.03563300
H	2.63567400	-2.52893000	1.89271800
H	3.26108300	-0.96225200	1.28580200
H	2.09408900	-1.00066300	2.63058500
C	-2.35771300	-2.16656100	-0.27240800
O	-2.87863200	-1.70549600	-1.26506200
O	-3.03104400	-2.59981900	0.76304100
C	-0.91086400	-1.19953800	2.09186700
O	-0.54494600	-1.32053000	3.35522600
H	-0.76443700	-3.47680800	-0.35255000
H	0.15038300	-2.99364300	1.69721200
H	-4.04468500	-2.33861300	0.64117400
O	-1.74352800	-0.40583200	1.71656200
H	-1.04014200	-0.67516900	3.89182100
Br	-1.26137700	2.33146800	4.00010600
Br	1.81028300	0.63288900	-1.51447700
C	6.27321200	-0.02930800	1.44082000
O	5.06533000	0.50540100	1.37977400
O	7.28663500	0.59987000	1.64067000
C	6.19875600	-1.53325100	1.28894300
C	5.61968500	-2.02319900	-0.06979800
C	5.52556800	-0.95608900	-1.13477100
O	4.46622300	-1.08089200	-1.91615700
O	6.36189800	-0.08964000	-1.28075800
C	6.63322900	-3.17037200	-0.47160300
C	6.98407200	-3.70375800	0.92722800
C	7.51320400	-2.35529900	1.45477700
C	8.46671200	-1.98592100	0.31369900
C	7.95019400	-2.48144300	-0.82522300
H	5.08081000	1.50972200	1.28319800
H	5.49868700	-1.85611000	2.07823200
H	4.62251800	-2.46679500	0.05903600
H	4.39668200	-0.28352100	-2.52575400
H	6.10956300	-4.07728400	1.48729200
H	7.76669300	-4.47450100	0.88509500
C	8.08988000	-2.32239600	2.85714100
H	7.34928100	-2.67738400	3.59036300

H	8.97858600	-2.96729900	2.92679700
H	8.38330700	-1.29842100	3.12962300
C	6.07573700	-4.14286400	-1.49199200
H	6.80266300	-4.94192400	-1.70032200
H	5.14862000	-4.60622200	-1.12076300
H	5.84447100	-3.63606400	-2.44122800
C	8.41847100	-2.32279600	-2.23539500
H	9.35526700	-1.75250000	-2.28512800
H	8.58332700	-3.30142900	-2.71358200
H	7.67431500	-1.78721100	-2.84752600
C	9.69183900	-1.15167600	0.50398900
H	10.26636600	-1.06519000	-0.42774200
H	9.41172000	-0.14350400	0.84145200
H	10.35105700	-1.58415700	1.27325400
N	4.30542200	2.02644500	-1.15348400
C	5.06618400	2.91390600	-0.48338800
O	5.07757900	3.02585800	0.75309100
C	5.98006800	3.71618500	-1.39867600
C	5.71617400	3.09254100	-2.76291100
C	4.68775300	2.01878300	-2.43909600
O	4.27947700	1.18338800	-3.27092600
H	7.00499800	3.59773200	-1.01917600
H	5.72467600	4.78316700	-1.32773400
H	5.29487900	3.78437600	-3.50629200
H	6.58823300	2.60621200	-3.22100200
O	6.90851900	0.29050800	-4.06116300
H	5.98270300	0.54335300	-4.19636300
H	6.96058000	0.25835400	-3.09307600
O	7.83263700	3.43778700	1.28189200
H	6.88430400	3.63698000	1.26993600
H	7.81111000	2.47674100	1.41126400

Complex **IM2b**

E (ωB97X-2 D3BJ/def2-TZVPP) = -8566.31805648888

C	10.58582100	1.71769800	1.91057700
C	9.62282000	1.53746400	2.87081700
C	8.31454100	1.10278000	2.51912500
C	8.00565900	0.83965200	1.14922300
C	9.02859300	1.04534600	0.17854100
C	10.28127700	1.47284500	0.54851200
N	7.40752300	0.96204700	3.52338100
C	6.19696000	0.58448700	3.20034600
C	5.78107900	0.29913400	1.87721500
C	6.67876700	0.39891600	0.84069400

C	6.24859100	0.09938400	-0.59400400
N	4.88714000	0.50916300	-0.86941800
C	6.44444500	-1.34111900	-1.11473500
N	5.48022700	-2.35663800	-0.61057500
C	5.92857300	-2.94398100	0.67478900
C	7.87501500	-1.89724800	-0.95567300
C	7.18677700	-3.81273200	0.46354600
C	7.79173000	-3.43044000	-0.89533500
C	6.85178800	-3.90634100	-2.01333800
C	6.87426400	-5.37810300	-2.32681200
C	7.70880600	-6.29026200	-1.82769100
H	6.21680900	-1.25651400	-2.18828200
C	4.57074400	1.84098600	-0.82009500
O	5.38184700	2.69606100	-0.52312700
N	3.25845200	2.10703300	-1.16430800
C	2.57861400	3.29024100	-0.88537700
C	2.89852500	4.08484800	0.22837500
C	2.14541200	5.21119200	0.53768900
C	1.04554700	5.57019100	-0.24394800
C	0.72212100	4.80769800	-1.36276200
C	1.49414800	3.69219100	-1.68338000
C	5.42313400	-3.44408800	-1.61640500
H	11.58542800	2.05331400	2.19080100
H	9.81832300	1.72364700	3.92756200
H	8.82585200	0.86054500	-0.87613500
H	11.04713800	1.62681200	-0.21323300
H	5.47299800	0.48306900	4.01620400
H	4.75362400	-0.01411000	1.69221700
H	6.88937200	0.71600200	-1.24073100
H	4.14532300	-0.18323500	-0.88155500
H	5.09052900	-3.52869700	1.08251000
H	6.12070600	-2.12310900	1.37354000
H	8.33889100	-1.53157900	-0.02765100
H	8.50148800	-1.56079600	-1.79493400
H	6.93281900	-4.88246100	0.47564700
H	7.91209200	-3.63430600	1.27013200
H	8.79320400	-3.86419600	-1.01856700
H	7.13441700	-3.37839700	-2.94160400
H	6.11547700	-5.70358900	-3.04915400
H	7.64258100	-7.33630400	-2.13342300
H	8.48700300	-6.03450700	-1.10410500
H	2.71980300	1.36550700	-1.60562800
H	3.75032600	3.79858500	0.84350800
H	2.41552200	5.80833800	1.40975900

H	0.44655200	6.44598100	0.00689700
H	-0.12054500	5.07885400	-1.99974000
H	4.85653700	-4.27199100	-1.16349800
H	4.85464200	-3.08953800	-2.48894100
C	0.85803200	-1.99802200	0.65690300
C	0.01209200	-1.84070100	-0.63509500
C	-1.36657100	-1.23458900	-0.09485200
C	-1.00626600	0.21626800	0.17028900
C	-0.07879900	0.20583400	1.31016500
C	0.01424300	-1.27311900	1.74820200
C	-1.35390600	-1.84933700	1.31187300
C	1.01424900	1.21270600	1.49046300
C	-1.21256100	1.27775700	-0.84979900
C	0.43974600	-1.50468000	3.18297200
C	-2.57257100	-1.46223400	-0.97305500
H	-2.21840700	-1.54895100	1.91364200
H	-1.32598800	-2.94598700	1.26669200
H	0.67311500	2.21828900	1.21154700
H	1.36464200	1.21869900	2.53020700
H	1.85333200	0.92957300	0.83958700
H	-2.29744000	1.46382700	-0.93000000
H	-0.68877400	2.20900100	-0.59911300
H	-0.87255500	0.92174900	-1.83262800
H	-0.28021900	-1.03779800	3.87118700
H	0.46447300	-2.58359100	3.39372400
H	1.43954700	-1.09341900	3.36860100
H	-2.78299300	-2.53850300	-1.05495200
H	-3.44685600	-0.95886600	-0.53946100
H	-2.42365100	-1.06148700	-1.98582800
C	2.34630500	-1.72522400	0.56697800
O	2.95584400	-1.08641100	1.39648700
O	2.90803300	-2.34108600	-0.44406800
C	0.67941500	-0.98587600	-1.68340200
O	0.22434900	-1.20703800	-2.90443300
H	0.82730900	-3.06462400	0.93079500
H	-0.24107000	-2.80723300	-1.08931600
H	3.95171500	-2.19895900	-0.45583100
O	1.50567400	-0.14233600	-1.42389900
H	0.64797500	-0.58187600	-3.52011500
Br	1.08137100	2.73621300	-3.27488600
Br	-1.87396200	0.98366100	1.99335500
C	-6.55030800	-0.72480500	-1.71650700
O	-5.31475300	-0.27066000	-1.84140100
O	-7.53511400	-0.14162100	-2.10710100

C	-6.53361600	-2.09702100	-1.07917900
C	-5.93460000	-2.13903300	0.35608600
C	-5.77404800	-0.78510600	1.00587000
O	-4.70116300	-0.68205100	1.77278200
O	-6.57405400	0.11564400	0.86029900
C	-6.97985600	-3.04471200	1.12741400
C	-7.38629200	-3.99268000	-0.01367300
C	-7.88076400	-2.87090900	-0.94818800
C	-8.78939400	-2.10721000	0.01991000
C	-8.26220000	-2.22384900	1.25192300
H	-5.25165300	0.69791600	-2.08006500
H	-5.86596200	-2.68878100	-1.72759400
H	-4.95961700	-2.64438400	0.36942800
H	-4.62483900	0.26156400	2.11081300
H	-6.54071000	-4.56620500	-0.42984200
H	-8.19494800	-4.67367900	0.28791100
C	-8.49359300	-3.27238800	-2.27593400
H	-7.78418100	-3.86998700	-2.86922400
H	-9.40082100	-3.87483200	-2.11948500
H	-8.76599300	-2.38127200	-2.85977800
C	-6.42675300	-3.64910800	2.40252700
H	-7.17061400	-4.30734200	2.87559100
H	-5.52590700	-4.24458300	2.18996600
H	-6.15436100	-2.86625200	3.12685600
C	-8.68882200	-1.59351600	2.53748000
H	-9.61822200	-1.02164900	2.41607500
H	-8.85284500	-2.35317600	3.31848900
H	-7.92277100	-0.89861800	2.91878400
C	-9.98306800	-1.32550700	-0.42275300
H	-10.48938400	-0.84879800	0.42666600
H	-9.67398300	-0.54498400	-1.13303400
H	-10.71095300	-1.97112700	-0.93992300
N	-4.35305000	1.95778900	0.14004000
C	-5.01809000	2.62408700	-0.83079600
O	-4.96823000	2.32997600	-2.03406500
C	-5.90179600	3.73810800	-0.28851200
C	-5.73780500	3.58966900	1.21621100
C	-4.79018800	2.40543200	1.32575500
O	-4.50120400	1.87646400	2.42220200
H	-6.91766200	3.56471700	-0.67187800
H	-5.55843600	4.70107200	-0.69316100
H	-5.28526400	4.45873400	1.71509100
H	-6.65937100	3.33916600	1.75915700
O	-7.20321700	1.48265100	3.29764700

H	-6.26022900	1.69295800	3.37681400
H	-7.23666100	1.10019800	2.40708100
O	-7.68341700	2.68608700	-2.80954700
H	-6.71926700	2.78543900	-2.80634800
H	-7.78032600	1.74411700	-2.59818900

Complex **IM3b**

E (ωB97X-2 D3BJ/def2-TZVPP) = -8566.32854250518

C	8.08960500	-3.10105100	-3.05946200
C	8.06410500	-1.75202600	-3.30983100
C	6.99880900	-0.94106400	-2.82777500
C	5.95818300	-1.53854200	-2.05229600
C	6.00797400	-2.94494200	-1.82757200
C	7.04314900	-3.70414400	-2.31814900
N	7.02105800	0.38320200	-3.13977600
C	6.02077000	1.12180100	-2.72832000
C	4.94251700	0.63811300	-1.94931800
C	4.91551100	-0.68486900	-1.57161200
C	3.81227200	-1.19273300	-0.65413700
N	2.56181300	-0.49833200	-0.88113800
C	4.12608600	-1.12922000	0.84968300
N	4.43714500	0.23267400	1.35289200
C	5.89331900	0.52004500	1.30606600
C	5.18829200	-2.10988600	1.37681400
C	6.65887800	-0.35296000	2.32149900
C	5.74242900	-1.52673000	2.68937100
C	4.56454100	-0.98917700	3.52052900
C	4.85845800	-0.65914700	4.95790800
C	5.98701800	-0.90092000	5.62610400
H	3.17624100	-1.40799500	1.33196500
C	1.87902500	-0.75533800	-2.03093900
O	2.31535600	-1.48502800	-2.90210300
N	0.64072600	-0.13648300	-2.10675000
C	-0.15715700	-0.16868500	-3.25901200
C	0.42114800	0.00112600	-4.52741300
C	-0.35752500	-0.02354500	-5.67786400
C	-1.73932800	-0.19989400	-5.59148600
C	-2.33298000	-0.36121700	-4.34417400
C	-1.54705600	-0.35600300	-3.19110700
C	4.01368200	0.25903500	2.77217000
H	8.90914500	-3.71493100	-3.43638000
H	8.84616400	-1.25543700	-3.88560000
H	5.21278500	-3.43532400	-1.26595700
H	7.05648300	-4.78034700	-2.13968800

H	6.04042400	2.18160000	-3.00500600
H	4.14952800	1.32552600	-1.64780800
H	3.63729000	-2.25356300	-0.88667300
H	2.19653900	0.17077200	-0.20358000
H	6.02258600	1.59389400	1.50619000
H	6.24572300	0.33579400	0.28576800
H	6.01497200	-2.23640900	0.66122700
H	4.72483400	-3.09513800	1.53430300
H	6.92159800	0.22086900	3.22199000
H	7.59587300	-0.71730900	1.87579700
H	6.28858200	-2.30203100	3.24363000
H	3.77374200	-1.75873300	3.51878700
H	4.03315800	-0.16748700	5.48846900
H	6.08646400	-0.62395700	6.67758400
H	6.84678900	-1.38530400	5.15643500
H	0.26829400	0.28263600	-1.25802300
H	1.49938500	0.14036500	-4.59080700
H	0.11975300	0.10478300	-6.65034800
H	-2.35616700	-0.21610900	-6.49080600
H	-3.40850800	-0.51321100	-4.25120900
H	4.40067100	1.19005700	3.21393300
H	2.91422200	0.30072000	2.81670800
C	1.61300000	4.00601700	0.81517100
C	0.86631900	3.38475300	2.01973600
C	-0.55653200	4.11013800	1.96740400
C	-1.24093000	3.34988800	0.85000200
C	-0.53202500	3.74035500	-0.38058700
C	0.50262800	4.78725100	0.03673600
C	-0.14214100	5.42701000	1.28921500
C	-0.42916100	2.93901000	-1.62491800
C	-2.06014500	2.12941600	1.09672900
C	1.00014800	5.69254000	-1.06980800
C	-1.29333100	4.16294200	3.28709300
H	-0.98103500	6.10019600	1.08297300
H	0.60278300	5.96709100	1.88719800
H	-1.23661500	2.19893900	-1.70640000
H	-0.43230600	3.59605500	-2.50486100
H	0.55261800	2.44181000	-1.59048300
H	-3.01164500	2.41911300	1.56389200
H	-2.25159600	1.55752700	0.18253000
H	-1.55064800	1.48342100	1.82429500
H	0.16278900	6.23189900	-1.53696400
H	1.70250900	6.43040200	-0.65715000
H	1.52543700	5.10832100	-1.83729800

H	-0.72122400	4.76640300	4.00491500
H	-2.28624500	4.61897200	3.15877000
H	-1.41386700	3.15542500	3.70917400
C	2.56432500	3.04567200	0.12260900
O	2.62234000	2.85920100	-1.07250800
O	3.36051800	2.51987700	1.02302000
C	0.74968700	1.86001600	1.91616700
O	0.54099500	1.24264800	2.99516100
H	2.28004000	4.79286800	1.19989400
H	1.31666100	3.64868300	2.98474500
H	3.78075000	1.57414800	0.85835600
O	0.77528000	1.37272100	0.77239100
H	-0.14251600	0.02595000	3.02435300
Br	-2.49753400	4.65182400	-0.38336200
Br	-2.41328000	-0.62376500	-1.52507100
C	-4.96885200	-3.38089000	0.07587500
O	-4.39048800	-4.04528100	1.06998400
O	-4.55978500	-3.38558100	-1.05772500
C	-6.19307600	-2.64136700	0.57701200
C	-5.88610300	-1.46130900	1.57338300
C	-4.40655900	-1.29976600	1.85395800
O	-4.03443900	-1.92266100	2.95823600
O	-3.62444500	-0.72086500	1.12753100
C	-6.63818400	-0.26032800	0.91752900
C	-7.88547700	-1.01630300	0.41643500
C	-7.09458500	-1.97068800	-0.50134600
C	-6.25861000	-0.94271100	-1.27353400
C	-5.99359900	0.07157600	-0.43149500
H	-3.49284800	-4.37131300	0.81165000
H	-6.78226700	-3.39495500	1.12270500
H	-6.35919000	-1.68627200	2.53876600
H	-3.07290600	-2.16509300	2.87062600
H	-8.43528100	-1.53579700	1.21992600
H	-8.56652200	-0.36461600	-0.14977800
C	-7.88354000	-2.95934400	-1.33893100
H	-8.49783400	-3.61051000	-0.69851600
H	-8.55452300	-2.43200800	-2.03388000
H	-7.20749300	-3.59455600	-1.93035800
C	-6.84446600	0.90628900	1.86505800
H	-7.43031100	1.70479900	1.38579400
H	-7.37950000	0.58442300	2.77128700
H	-5.87772800	1.33490300	2.17558100
C	-5.24454600	1.33920800	-0.67905500
H	-4.87690700	1.39820100	-1.71229100

H	-5.87959500	2.21899300	-0.48392900
H	-4.37729200	1.39858300	-0.00787100
C	-5.86137000	-1.10126000	-2.70607400
H	-5.42270500	-0.17291700	-3.09813100
H	-5.12680700	-1.91346700	-2.81162400
H	-6.73269000	-1.35473100	-3.33038200
N	-1.51800700	-2.70955600	2.14647600
C	-0.74667500	-1.64712000	2.17528500
O	-0.78528900	-0.81588200	3.14704400
C	0.11667000	-1.46568800	0.96782100
C	-0.00625000	-2.81485900	0.28874600
C	-1.20421700	-3.42435300	0.99832500
O	-1.78702700	-4.43081800	0.63619400
H	1.12345500	-1.15590000	1.25410100
H	-0.34446000	-0.65750300	0.38612500
H	-0.17267500	-2.78254100	-0.79617900
H	0.86644500	-3.45175800	0.50145300
O	-0.04433900	-5.03209400	3.17081800
H	-0.25814500	-5.58031200	2.40372900
H	-0.84393400	-4.49606800	3.28885500
O	1.75551600	-2.91170300	2.96036600
H	1.23245700	-3.73478000	3.04432100
H	1.51776900	-2.40931000	3.74978300

Complex **(-)-6a +AU5**

E (ωB97X-2 D3BJ/def2-TZVPP) = -8566.40096115151

C	-7.14035900	-2.41486600	4.79705800
C	-6.43366400	-1.23977000	4.84045600
C	-5.60216800	-0.85091500	3.75361500
C	-5.52114000	-1.68753400	2.59740800
C	-6.25607400	-2.90827200	2.59435300
C	-7.04314900	-3.26107300	3.66417000
N	-4.90757000	0.31203300	3.87589200
C	-4.10559000	0.65002700	2.89883400
C	-3.94663700	-0.10364900	1.71145800
C	-4.67726400	-1.25654800	1.52569700
C	-4.58076600	-2.00985100	0.20545900
N	-3.26410300	-1.90043200	-0.37722200
C	-5.62269500	-1.60361700	-0.85539900
N	-5.66091000	-0.14894400	-1.16213700
C	-6.64434800	0.56480200	-0.30427100
C	-7.06571000	-2.09537600	-0.64247800
C	-8.08480900	0.15882800	-0.67626400
C	-7.99684200	-1.16719800	-1.44325800
C	-7.36299600	-0.89562400	-2.81911600

C	-8.25665900	-0.26236800	-3.85103700
C	-9.56150100	-0.01334900	-3.73900400
H	-5.24105000	-2.07932900	-1.77005400
C	-2.30771100	-2.82772000	-0.07192000
O	-2.54994200	-3.85308700	0.53871900
N	-1.05176100	-2.48597800	-0.53650600
C	0.07655000	-3.30717500	-0.45920100
C	-0.01041500	-4.70821000	-0.51390900
C	1.13756700	-5.49427700	-0.48372800
C	2.40420400	-4.91291200	-0.41346900
C	2.50972200	-3.52606200	-0.35040400
C	1.35921200	-2.74280100	-0.36351500
C	-6.10472500	-0.01600000	-2.56870300
H	-7.76878000	-2.70764300	5.63956300
H	-6.47062500	-0.57407700	5.70357500
H	-6.18830300	-3.58069900	1.73878400
H	-7.59319500	-4.20305400	3.64517300
H	-3.53852000	1.57957800	3.02445400
H	-3.24726400	0.24361100	0.94958200
H	-4.73462400	-3.08008500	0.39792500
H	-2.99898600	-1.05988500	-0.89184500
H	-6.47413000	1.64327400	-0.43558200
H	-6.42265800	0.32585400	0.74203100
H	-7.35490900	-2.05517200	0.41826800
H	-7.14766300	-3.14225300	-0.96993600
H	-8.56437700	0.92810300	-1.29825600
H	-8.69025000	0.04175200	0.23378700
H	-8.98707600	-1.62840100	-1.55708700
H	-7.02536400	-1.86030000	-3.23802800
H	-7.74857400	0.01460400	-4.78309100
H	-10.11329600	0.45320100	-4.55737400
H	-10.12731600	-0.26346900	-2.83806500
H	-0.91269300	-1.53096900	-0.86087200
H	-0.99432400	-5.16639400	-0.58060300
H	1.03593700	-6.57961600	-0.53167000
H	3.31839200	-5.50723300	-0.41696400
H	3.48192700	-3.03515100	-0.28782200
H	-6.32763600	1.04699100	-2.74581800
H	-5.27310800	-0.30111100	-3.22988800
C	-2.00224000	2.94701900	-1.58754900
C	-0.68614700	3.05592300	-2.39351300
C	0.05879900	4.19797100	-1.64710500
C	0.66441600	3.34518300	-0.48596100
C	-0.54819000	3.16417200	0.50548600

C	-1.74136800	3.78328700	-0.28628900
C	-1.05247500	4.98803800	-0.95485100
C	-0.70777200	1.75304000	1.04125500
C	1.97188900	3.84759700	0.08125600
C	-3.01373100	4.05226800	0.49744200
C	1.06997000	4.95567700	-2.48462900
H	-0.66395900	5.71746800	-0.23100400
H	-1.71303400	5.50322600	-1.66745500
H	0.19988900	1.45168800	1.58301200
H	-1.55680000	1.70793000	1.73637700
H	-0.86397000	1.02974200	0.23270000
H	1.89194300	4.90688400	0.35382300
H	2.24656700	3.27535300	0.97653500
H	2.74773200	3.71145700	-0.68198100
H	-2.85073800	4.82748700	1.25582000
H	-3.80719900	4.38783700	-0.18540300
H	-3.37594300	3.14175000	0.99752600
H	0.57659700	5.39465400	-3.36360300
H	1.51684400	5.77324000	-1.90137100
H	1.89101600	4.30280200	-2.82139300
C	-2.66653400	1.59104800	-1.44256200
O	-2.09092000	0.52262400	-1.58059900
O	-3.93169200	1.70668600	-1.16168600
C	0.27898500	1.91100900	-2.23562200
O	0.51809700	1.02452000	-3.02515100
H	-2.74936600	3.54397100	-2.12893800
H	-0.86933400	3.23480100	-3.45921600
H	-4.48555000	0.79649900	-1.11311100
O	0.95934500	2.05973600	-1.10487700
H	2.00638700	0.49658400	-3.00823500
Br	-0.26190400	4.28363500	2.14276700
Br	1.56505900	-0.86160300	-0.27761000
C	6.51295300	-1.36681700	0.31733400
O	7.57975500	-1.63325700	-0.41147200
O	5.41845400	-1.84047100	0.07730800
C	6.78104600	-0.39366000	1.44024200
C	6.17384500	1.03241800	1.19288000
C	5.41288800	1.13717400	-0.11145900
O	6.19842600	0.86356500	-1.13959000
O	4.24385100	1.43361300	-0.22320000
C	5.38665800	1.32764200	2.50648700
C	6.31170600	0.61048100	3.50944600
C	6.18473600	-0.77098100	2.83450800
C	4.65841800	-0.86546900	2.72147900

C	4.18252700	0.38198700	2.56036900
H	7.28805900	-2.05637200	-1.25384600
H	7.87172500	-0.31238800	1.53373400
H	7.01132800	1.74267600	1.09774500
H	5.67488700	0.44028000	-1.89498000
H	7.34409200	0.99960700	3.51197400
H	5.90232400	0.61376000	4.52952900
C	6.87752100	-1.94907100	3.49227600
H	7.95804900	-1.76404300	3.58839400
H	6.46693100	-2.12908300	4.49681900
H	6.74079400	-2.86937500	2.90307600
C	5.09737700	2.79966500	2.72543500
H	4.60778100	2.96365000	3.69693700
H	6.02658800	3.38934100	2.70659800
H	4.43157100	3.18113600	1.93648600
C	2.76817200	0.85182400	2.45761900
H	2.05730200	0.03278500	2.63277600
H	2.56508800	1.64704800	3.19451000
H	2.57989700	1.26789800	1.45651000
C	3.91529000	-2.16092400	2.79059400
H	2.84645000	-2.02447400	2.57297900
H	4.31808100	-2.87737100	2.05920900
H	4.01016900	-2.62323400	3.78675600
N	4.87583100	-0.74433800	-2.77169700
C	3.60423600	-0.63638100	-3.04100800
O	3.01108300	0.49775400	-2.92965300
C	2.93228200	-1.89983100	-3.51259600
C	4.07587900	-2.90481900	-3.43309400
C	5.24916800	-2.06920200	-2.94596600
O	6.35958900	-2.50721700	-2.71375400
H	2.54253300	-1.73851300	-4.52851800
H	2.07695700	-2.13596300	-2.86284400
H	3.92135500	-3.70666900	-2.69580800
H	4.33291200	-3.37208500	-4.39322300
O	4.04399500	3.13313900	-2.55946700
H	4.69187800	3.56273800	-1.98865200
H	3.85812000	2.30460500	-2.08785200
O	5.52763000	-4.56894400	-0.91603000
H	6.03718100	-4.38701300	-1.71712400
H	5.47908700	-3.68978400	-0.50703400

6a

E (ωB97X-2 D3BJ/def2-TZVPP) = -3377.194675256962

C	2.29251900	-1.59072100	0.04229200

O	3.56312300	-1.18715400	0.01744500
O	1.91842800	-2.61348700	-0.46700200
C	1.44971100	-0.64516100	0.86831200
C	1.66596100	0.88212200	0.67350500
C	1.86063300	1.28403600	-0.77775300
O	2.87636300	1.40882300	-1.39378200
O	0.65164700	1.49990400	-1.32951700
C	0.26518500	1.44466800	1.01877900
C	-0.39710600	0.35899000	1.86905100
C	-0.09260000	-0.82129300	0.92455100
C	-0.73159300	-0.30190100	-0.40166400
C	-0.39996500	1.24739800	-0.38131100
H	4.07969500	-1.82438600	-0.50357400
H	1.79512200	-0.86045300	1.89414100
H	2.49855800	1.26293600	1.27596400
H	0.07979400	0.24046000	2.85335900
H	-1.47375800	0.52835400	2.01051800
C	-0.51255200	-2.20329800	1.39796300
H	0.10179800	-2.50013700	2.26175300
H	-1.56435300	-2.20701000	1.70915600
H	-0.36927200	-2.95478200	0.61001900
C	0.26831400	2.86368900	1.55059500
H	-0.75240800	3.21034700	1.76496500
H	0.84974800	2.90997100	2.48265800
H	0.72444700	3.55935700	0.82940300
C	-1.51862800	2.20667900	-0.72548300
H	-1.98652100	1.92037800	-1.67734600
H	-2.28940800	2.21146400	0.05401700
H	-1.09480800	3.21457200	-0.82573000
C	-0.33376200	-0.98758000	-1.69814700
H	-0.87307600	-0.53043000	-2.53892500
H	0.73963900	-0.88389500	-1.89628200
H	-0.57021100	-2.05781400	-1.65271600
Br	-2.71052000	-0.58655400	-0.26950500

Complex **TSa**$^{\mathbf{AU6}}$

E (ωB97X-2 D3BJ/def2-TZVPP) = -6291.2035933017

C	6.05544100	-1.90377900	1.39316300
O	4.99885500	-1.18049400	1.71408100
O	7.04826000	-2.00248000	2.07788900
C	5.82270500	-2.62984300	0.08550400
C	5.61021800	-1.70199700	-1.14523800
C	5.95060600	-0.24946400	-0.91385300
O	5.13495100	0.58702000	-1.53563700

O	6.89888700	0.11760300	-0.25281800
C	6.55069500	-2.36460900	-2.22967200
C	6.39068400	-3.84179500	-1.83208800
C	6.90474600	-3.64709800	-0.39198600
C	8.19890400	-2.87908600	-0.68008700
C	7.98817000	-2.12370100	-1.77340700
H	5.17391200	-0.53694200	2.46858600
H	4.88225500	-3.18399200	0.24266800
H	4.57398000	-1.75354400	-1.50568100
H	5.33962500	1.52880900	-1.26128800
H	5.34673200	-4.19589700	-1.87852600
H	7.04192500	-4.49876300	-2.42608000
C	7.02602200	-4.87230000	0.49324200
H	6.05459800	-5.38210000	0.58726600
H	7.75063700	-5.58817100	0.07756400
H	7.36144300	-4.58505600	1.50036000
C	6.21180300	-1.96237000	-3.65174000
H	6.86565300	-2.48278900	-4.36750300
H	5.16740200	-2.21507300	-3.89113300
H	6.34076000	-0.87908200	-3.79908700
C	8.87625000	-1.11309300	-2.42349200
H	9.84048400	-1.03232200	-1.90505100
H	9.07310600	-1.37325100	-3.47596600
H	8.41148800	-0.11347700	-2.42351800
C	9.41152000	-2.96075600	0.18935300
H	10.22037900	-2.32057500	-0.18668100
H	9.16169100	-2.64920100	1.21320100
H	9.78907100	-3.99453800	0.24256600
N	5.15843100	1.86369700	1.40332600
C	5.84806100	1.62871300	2.54125000
O	5.55704500	0.74949900	3.36476500
C	7.08609400	2.50526700	2.67338000
C	7.08781800	3.27985400	1.36315900
C	5.86024100	2.72498600	0.65657000
O	5.56699500	3.01215700	-0.52516300
H	7.94283600	1.83169800	2.81946300
H	7.00235800	3.12853400	3.57506900
H	6.97654500	4.36805800	1.47529200
H	7.96504300	3.10193100	0.72623400
O	8.13046200	2.29812100	-1.64035300
H	7.32180600	2.81726700	-1.51315300
H	7.96977700	1.53914600	-1.05841200
C	-9.57555100	-3.78755200	-2.23189000
C	-8.52899100	-3.67023800	-3.11155800

C	-7.33829700	-2.98627200	-2.73906700
C	-7.22396400	-2.44054000	-1.42347300
C	-8.33860300	-2.56388300	-0.54430700
C	-9.48149600	-3.21760000	-0.93798800
N	-6.35658100	-2.87343800	-3.67417900
C	-5.27785500	-2.20959300	-3.34826700
C	-5.05465900	-1.63763300	-2.07167000
C	-6.00190100	-1.77498600	-1.08218100
C	-5.75999800	-1.23459900	0.31745600
N	-4.96755100	-0.01945400	0.30319500
C	-5.18370600	-2.23521600	1.34850300
N	-3.83633500	-2.77220600	1.01181500
C	-3.92939200	-4.03569400	0.23200900
C	-6.08114300	-3.41250600	1.76602600
C	-4.46830500	-5.17589000	1.11718100
C	-5.16831500	-4.52347400	2.31691400
C	-4.09607700	-3.88654800	3.21803400
C	-3.30188500	-4.83142700	4.07935500
C	-3.50743600	-6.13931400	4.23545300
H	-5.02657100	-1.60445700	2.23597600
C	-5.38953700	1.05781700	1.04689400
O	-6.44412500	1.07021300	1.65546800
N	-4.52270800	2.12914200	1.01226300
C	-4.75739400	3.33954200	1.67639200
C	-5.28354700	3.36218800	2.97818900
C	-5.49449500	4.56375200	3.64394900
C	-5.16883000	5.77658500	3.03614800
C	-4.64494100	5.77932600	1.74635900
C	-4.45881600	4.57172700	1.07389900
C	-3.15116400	-3.07015100	2.29238600
H	-10.48560000	-4.31157700	-2.52784100
H	-8.57373300	-4.08147100	-4.12057300
H	-8.29506200	-2.13048000	0.45492900
H	-10.32498000	-3.29375800	-0.25035600
H	-4.50583100	-2.10479100	-4.11816700
H	-4.11411900	-1.12011000	-1.88823200
H	-6.72485700	-0.92663400	0.73912800
H	-3.99051000	-0.06529800	0.02206800
H	-2.92523000	-4.25267300	-0.15971600
H	-4.58399200	-3.85585100	-0.62888800
H	-6.64543000	-3.81208700	0.91056000
H	-6.81022000	-3.07041200	2.51514900
H	-3.65308100	-5.82807700	1.46127100
H	-5.17229600	-5.79635300	0.54470600

H	-5.76355700	-5.25666600	2.87720900
H	-4.59876000	-3.17930700	3.90157200
H	-2.47341000	-4.36091900	4.62313500
H	-2.86884200	-6.73262400	4.89278800
H	-4.31464200	-6.66722800	3.72114600
H	-3.68816200	2.05433800	0.43209300
H	-5.52694800	2.41365400	3.45321200
H	-5.90528600	4.54950500	4.65424500
H	-5.32430900	6.72202900	3.55675600
H	-4.39806900	6.72042900	1.25403500
H	-2.23995700	-3.63640800	2.05108800
H	-2.83130900	-2.12483100	2.74972500
C	-1.14425600	-1.18061400	0.00844400
O	-2.31179900	-1.46246700	-0.51108200
O	-0.84801400	-1.36237100	1.16974000
C	-0.15393100	-0.71495600	-1.04687900
C	-0.62986200	0.36796600	-2.05630000
C	-1.85051900	1.10984200	-1.57459500
O	-2.65405200	1.49443600	-2.54988200
O	-2.06362900	1.34744000	-0.40433000
C	0.67023300	1.26773700	-2.26125900
C	1.73130200	0.17403000	-2.04976600
C	1.27260400	-0.24334200	-0.63312100
C	1.07017900	1.10259400	0.07782200
C	0.77044300	2.08127900	-0.97049300
H	-2.98534300	-1.96841700	0.21680600
H	0.01736100	-1.63525000	-1.62707700
H	-0.86335100	-0.06273900	-3.03799800
H	-3.40820900	1.98940000	-2.17630500
H	1.60228800	-0.64946400	-2.76501200
H	2.76730700	0.52463600	-2.11039300
C	2.12456500	-1.25005600	0.10947000
H	2.25019900	-2.15287700	-0.50748600
H	3.11822300	-0.83011400	0.32578900
H	1.65140700	-1.54337200	1.05528300
C	0.69782500	2.07102300	-3.54291800
H	1.61964500	2.66950800	-3.59062700
H	0.67348200	1.40063400	-4.41297000
H	-0.16511600	2.74996300	-3.60807800
C	0.09713400	3.41025600	-0.81181400
H	0.09386600	3.74450500	0.23268000
H	0.61334600	4.15713200	-1.42916800
H	-0.94083100	3.35657600	-1.16329200
C	0.69595900	1.23355500	1.51399600

H	0.69239000	2.28141900	1.83469900
H	-0.31045800	0.80756400	1.62993200
H	1.38818300	0.65559200	2.13949700
Br	2.75647700	2.30628000	-0.30575500
I	-3.77314200	4.66573300	-0.92308500
O	8.11263400	-0.22947800	4.11809500
H	7.90048000	-0.88742100	3.43762400
H	7.24549300	0.18631500	4.23830200

Complex **TSb**$^{\mathbf{AU6}}$

E (ωB97X-2 D3BJ/def2-TZVPP) = -6291.20333062048

C	9.63933200	1.82560300	3.31400800
C	8.63173800	1.30216200	4.08440000
C	7.45023300	0.78287500	3.48496400
C	7.31837100	0.79582300	2.06260200
C	8.38146300	1.35422200	1.29554300
C	9.50766100	1.85501800	1.90360700
N	6.48622700	0.29905200	4.31371500
C	5.38709500	-0.15651900	3.76876500
C	5.14765400	-0.18737900	2.37310900
C	6.11250400	0.26221100	1.50268200
C	5.86921300	0.25112400	-0.00456900
N	4.49713600	0.57609400	-0.34138200
C	6.32081300	-0.98851600	-0.80796000
N	5.50242100	-2.22330700	-0.66849300
C	5.93030700	-3.02904300	0.49895000
C	7.80682200	-1.35579800	-0.60908600
C	7.33258900	-3.62856300	0.25846500
C	7.97864800	-2.85411800	-0.89977400
C	7.23248000	-3.18020900	-2.20206700
C	7.52511200	-4.51941600	-2.82383400
C	8.44538200	-5.39914400	-2.42776500
H	6.17339000	-0.68027600	-1.85480800
C	4.08765200	1.87950700	-0.21847400
O	4.74982500	2.71682100	0.36375300
N	2.87793300	2.14019000	-0.83043800
C	2.13526700	3.31302900	-0.70394500
C	2.09652600	4.03116900	0.50342000
C	1.28199900	5.14963400	0.64002100
C	0.47504800	5.57537800	-0.41566600
C	0.50897100	4.88717100	-1.62607000
C	1.34462800	3.78052400	-1.76823900
C	5.71785300	-3.03103900	-1.89278700
H	10.54027500	2.22569800	3.78171100

H	8.69334400	1.26910300	5.17280500
H	8.31194500	1.39153600	0.20878700
H	10.30544100	2.28374900	1.29519500
H	4.61766500	-0.53607200	4.44976100
H	4.20578600	-0.58639700	1.99660800
H	6.48644400	1.06496200	-0.41298500
H	3.90643600	-0.10906800	-0.80345400
H	5.17190700	-3.80951600	0.65977500
H	5.91211700	-2.38039800	1.38135200
H	8.12502100	-1.16062500	0.42562600
H	8.43468400	-0.73937800	-1.26919200
H	7.26571600	-4.69684900	0.00749400
H	7.94472600	-3.53990000	1.16721100
H	9.04568300	-3.09792200	-0.99147500
H	7.50332900	-2.41373200	-2.95003100
H	6.90220200	-4.76889200	-3.69182300
H	8.57964400	-6.34585500	-2.95463600
H	9.09780200	-5.21124200	-1.57126300
H	2.49991700	1.41671200	-1.43535100
H	2.71921700	3.69124900	1.33013900
H	1.26956900	5.68815400	1.58867600
H	-0.17480800	6.44412600	-0.30684600
H	-0.10469100	5.21652700	-2.46518800
H	5.25637600	-4.01466400	-1.71692300
H	5.17992400	-2.55524000	-2.72614700
C	0.76995900	-2.50688100	0.09354500
C	0.07924600	-2.01345100	-1.20869800
C	-1.29789800	-1.38966100	-0.68468000
C	-0.86931800	-0.06947400	-0.05373700
C	-0.07511900	-0.42939500	1.12936000
C	-0.15485700	-1.96843700	1.22789000
C	-1.49957300	-2.29522400	0.53672700
C	1.06089200	0.40080300	1.64161900
C	-0.84742600	1.21206200	-0.82542100
C	0.05349100	-2.55256800	2.60958400
C	-2.39619300	-1.29830900	-1.71870400
H	-2.40120300	-2.05658800	1.11357400
H	-1.54211900	-3.35347800	0.24515900
H	0.81888400	1.47055200	1.59455500
H	1.31071200	0.12256100	2.67299400
H	1.93894700	0.21173300	1.00809600
H	-1.88277900	1.54009000	-0.99917400
H	-0.30051800	2.00566800	-0.29980000
H	-0.39596500	1.05524400	-1.81548400

H	-0.70817900	-2.16197100	3.30034600
H	-0.05103300	-3.64628700	2.56653800
H	1.05189800	-2.31463300	2.99419000
H	-2.69071800	-2.30712900	-2.04252800
H	-3.27265300	-0.78737900	-1.29414000
H	-2.06863700	-0.73479300	-2.60454500
C	2.26827300	-2.31754900	0.24921600
O	2.77304800	-2.00240200	1.30490400
O	2.95367500	-2.61622700	-0.82428700
C	0.89304400	-1.03841000	-2.01465100
O	0.55221800	-1.00354600	-3.29035900
H	0.65709000	-3.60262900	0.12087200
H	-0.20306600	-2.84693900	-1.86433300
H	3.96385700	-2.35592900	-0.68104700
O	1.72525300	-0.30335500	-1.53310600
H	1.05517300	-0.29451300	-3.73213900
Br	-1.89143900	0.36298200	1.74909100
C	-6.27030000	0.09479100	-1.42333500
O	-5.07636300	0.63171600	-1.23877100
O	-7.28407900	0.73235500	-1.59203100
C	-6.17529300	-1.41627900	-1.45324900
C	-5.65259300	-2.06110700	-0.13730700
C	-5.59931700	-1.12831800	1.04932900
O	-4.56208700	-1.34069100	1.84015500
O	-6.44889700	-0.29169500	1.27281400
C	-6.68144500	-3.24086900	0.08701500
C	-6.95285300	-3.62089400	-1.37821200
C	-7.46748600	-2.22565100	-1.78355600
C	-8.48585500	-1.98961700	-0.66353700
C	-8.02027200	-2.59643500	0.44301500
H	-5.11235100	1.61606600	-1.02130700
H	-5.43286200	-1.62926800	-2.24130000
H	-4.65194500	-2.49442100	-0.27544800
H	-4.51063600	-0.62242000	2.54381000
H	-6.04547400	-3.92769600	-1.92677500
H	-7.72744600	-4.39602800	-1.46728100
C	-7.96516800	-2.03592800	-3.20308800
H	-7.17832500	-2.29232800	-3.92970500
H	-8.83664600	-2.67721500	-3.40209700
H	-8.25970800	-0.98967900	-3.37028100
C	-6.17048700	-4.31451200	1.02732200
H	-6.90575900	-5.12662300	1.12839800
H	-5.22885900	-4.74422300	0.65187500
H	-5.97882000	-3.90408400	2.03079800

C	-8.56543400	-2.59481700	1.83435900
H	-9.49182300	-2.00933200	1.90000100
H	-8.77892500	-3.61889600	2.18046600
H	-7.84569100	-2.15821400	2.54565500
C	-9.71929200	-1.16407000	-0.83571300
H	-10.32898000	-1.15675600	0.07758400
H	-9.44989900	-0.12894500	-1.08945300
H	-10.34111800	-1.55207600	-1.65835900
N	-4.39114100	1.82727300	1.47491000
C	-5.15225500	2.78694600	0.91202600
O	-5.14940200	3.05401400	-0.30028000
C	-6.08773400	3.45856100	1.90786500
C	-5.81717100	2.68690400	3.19261900
C	-4.78745300	1.66060000	2.74492800
O	-4.39229300	0.72513300	3.47087200
H	-7.10782100	3.35825200	1.50973600
H	-5.85964900	4.53269600	1.95916300
H	-5.39123100	3.29151200	4.00626700
H	-6.68533000	2.14916300	3.59764000
O	-7.04065800	-0.26960300	4.06929400
H	-6.12106000	-0.03657200	4.26594500
H	-7.06691100	-0.16000600	3.10576800
I	1.42986500	2.84822800	-3.66484600
O	-7.90669000	3.46575100	-0.80196500
H	-6.96289100	3.68129700	-0.75711900
H	-7.86436800	2.53357000	-1.06656100

Complex **IM1a’**

E (ωB97X-2 D3BJ/def2-TZVPP) = -7608.02751556874

C	-8.63690300	1.10938200	-1.33901900
C	-7.74077100	0.96816400	-2.36792400
C	-6.36790800	0.70090300	-2.10570600
C	-5.92627700	0.55344000	-0.75416600
C	-6.88232100	0.72431400	0.28946300
C	-8.19855300	0.99543500	0.00407800
N	-5.52988400	0.60883800	-3.17293100
C	-4.25938400	0.40167500	-2.93569300
C	-3.71564800	0.23551300	-1.63973000
C	-4.53973700	0.27328600	-0.53660100
C	-3.96454500	0.02893100	0.85330800
N	-2.65570000	0.63359600	0.99873200
C	-3.94792500	-1.43939900	1.32819300
N	-3.27308800	-2.41194200	0.43217900
C	-4.21016100	-2.97495100	-0.57128100

C	-5.32693200	-2.00772500	1.71987800
C	-5.25728800	-3.88391100	0.10521100
C	-5.26038100	-3.53966700	1.60084100
C	-3.93647500	-4.02009700	2.21671300
C	-3.81583500	-5.49885300	2.46675700
C	-4.77009800	-6.41935000	2.32418500
H	-3.32353700	-1.40432700	2.23279400
C	-2.58877600	1.94033400	1.41740400
O	-3.54086500	2.52075700	1.90765600
N	-1.35211600	2.52829100	1.23681000
C	-1.03022100	3.81482200	1.67439500
C	-1.53085700	4.33174200	2.88127800
C	-1.17673100	5.60347000	3.31672100
C	-0.30201200	6.39417300	2.57044500
C	0.20692500	5.90485700	1.37156900
C	-0.16648200	4.63693600	0.93168600
Br	0.50445400	4.04735200	-0.74728500
C	-2.79543600	-3.52481300	1.28327800
H	-9.68643600	1.32057700	-1.54994200
H	-8.03852000	1.06282100	-3.41285800
H	-6.56992000	0.65316200	1.33130600
H	-8.91273600	1.13148600	0.81752200
H	-3.59245500	0.34956000	-3.80305200
H	-2.64665800	0.04435900	-1.54171200
H	-4.59298900	0.55484900	1.58206700
H	-1.84905800	0.21669900	0.53908700
H	-3.60860000	-3.52184700	-1.31198600
H	-4.69304800	-2.14447700	-1.09781500
H	-6.11546500	-1.63357800	1.04901600
H	-5.58091800	-1.68723000	2.74123000
H	-5.01131100	-4.94557200	-0.03955400
H	-6.25040700	-3.71002900	-0.33358700
H	-6.11736000	-3.99815000	2.11270900
H	-3.81347800	-3.52365400	3.19610600
H	-2.82283300	-5.82338500	2.80165400
H	-4.56969100	-7.47082700	2.53939900
H	-5.78033800	-6.16432700	1.99403400
H	-0.66923500	2.02163400	0.67239000
H	-2.20683100	3.71301700	3.46756200
H	-1.58182400	5.97546400	4.25893000
H	-0.01667500	7.38903100	2.91355600
H	0.88266400	6.50599300	0.76263900
H	-2.45989100	-4.33082800	0.61258900
H	-1.91479900	-3.18816900	1.84460900

C	-0.08705700	-2.22133600	-0.65798300
O	-1.26498300	-1.84014500	-1.11307600
O	0.07819900	-2.75965900	0.41391300
C	1.01479700	-1.97588600	-1.66887600
C	1.10203300	-0.53281600	-2.24103000
C	0.21736200	0.48272600	-1.57845400
O	-0.35643200	1.31540600	-2.43929800
O	0.05286500	0.57518600	-0.37982300
C	2.63676300	-0.18642500	-2.06338400
C	3.21631200	-1.57667100	-2.37345300
C	2.47804300	-2.29249400	-1.22505800
C	2.81810300	-1.35760100	-0.06086600
C	2.91355200	-0.10616100	-0.56138000
H	-2.03238700	-2.02044700	-0.42197700
H	0.76002000	-2.64322300	-2.50935100
H	0.87082700	-0.51997400	-3.31360000
H	-0.85887400	1.98604500	-1.94142400
H	2.93199100	-1.95131800	-3.37058400
H	4.30902900	-1.60882600	-2.26091300
C	2.77394900	-3.76485700	-1.01567700
H	2.53713500	-4.34130600	-1.92293100
H	3.83954500	-3.91365800	-0.78380500
H	2.17612500	-4.16579100	-0.18522700
C	3.09428200	0.99284000	-2.89774800
H	4.17538800	1.15470000	-2.77411700
H	2.88805100	0.81967100	-3.96432100
H	2.57694300	1.91791100	-2.59629200
C	3.13561000	1.18733700	0.15434000
H	3.33469600	1.02778300	1.22234400
H	3.98990300	1.73480100	-0.27465000
H	2.25247300	1.83916000	0.06700600
C	2.91770900	-1.81106900	1.36037700
H	3.22355100	-0.99138900	2.02424900
H	1.94673600	-2.20172200	1.69562600
H	3.65327200	-2.62503300	1.46124600
Br	5.89265300	-0.79654200	0.01239300
N	7.69877300	-0.73526500	0.37903700
C	8.43890600	-1.87019800	0.71849500
O	7.99792700	-2.98167200	0.78785600
C	9.86153800	-1.40339400	0.97460400
C	9.86012000	0.10245500	0.70399200
C	8.42572600	0.45683500	0.35281000
O	7.96877300	1.53331600	0.09296300
H	10.53315600	-1.97347300	0.31924600

H	10.11719800	-1.66012700	2.01176500
H	10.16120200	0.70859100	1.56866800
H	10.49576500	0.39550700	-0.14267200

Complex **TSa’**

E (ωB97X-2 D3BJ/def2-TZVPP) = -7607.97019122961

C	-8.56823800	-0.24856200	-2.23259900
C	-7.57682900	-0.44155400	-3.16109700
C	-6.20648400	-0.42090300	-2.77794200
C	-5.86327800	-0.21949400	-1.40577400
C	-6.91916000	-0.00776300	-0.47231700
C	-8.23257500	-0.02130000	-0.87458200
N	-5.27540300	-0.58564700	-3.75638600
C	-4.01389800	-0.52624100	-3.41450600
C	-3.56188300	-0.32972900	-2.08692500
C	-4.47264300	-0.20962400	-1.06156900
C	-4.01068300	-0.04872400	0.37909400
N	-2.79943400	0.74165800	0.48127300
C	-3.88178700	-1.34471000	1.21144100
N	-2.88175600	-2.32834900	0.71568800
C	-3.48897000	-3.30306600	-0.22732600
C	-5.19020500	-2.09576000	1.51557700
C	-4.46486900	-4.23788700	0.51496500
C	-4.82457900	-3.55451600	1.84153700
C	-3.58494900	-3.56312900	2.75294700
C	-3.26637300	-4.86940500	3.42917900
C	-3.99607500	-5.98509300	3.40149500
H	-3.47343100	-0.97868000	2.16491000
C	-2.82484800	1.92639000	1.17352600
O	-3.83474700	2.36843800	1.69151200
N	-1.61015900	2.57944700	1.21155600
C	-1.40409200	3.79660100	1.86944500
C	-2.01894400	4.08624300	3.09798700
C	-1.78091000	5.29089600	3.74992200
C	-0.91101600	6.23538300	3.20391000
C	-0.28923400	5.97166100	1.98702400
C	-0.55024200	4.77137800	1.32963500
Br	0.26475400	4.49523300	-0.36802100
C	-2.38377700	-3.07713000	1.89370000
H	-9.61609900	-0.26009300	-2.53620300
H	-7.79598900	-0.60821300	-4.21638300
H	-6.69142300	0.17618900	0.57757600
H	-9.02305000	0.14879400	-0.14204500
H	-3.27483800	-0.64571500	-4.21407000

H	-2.48970700	-0.30154800	-1.89414000
H	-4.76532100	0.54382500	0.91083200
H	-1.91153500	0.35440900	0.16987000
H	-2.66542100	-3.85623700	-0.70094300
H	-3.99831100	-2.73941200	-1.01778000
H	-5.86686700	-2.08759600	0.64763300
H	-5.71211600	-1.60410500	2.34976300
H	-4.00615700	-5.21912700	0.70278800
H	-5.36619700	-4.40166000	-0.09253300
H	-5.66888500	-4.05673900	2.33222300
H	-3.74757300	-2.82260700	3.55636600
H	-2.33072000	-4.86949600	4.00194100
H	-3.67410400	-6.87833900	3.94043100
H	-4.93687100	-6.05133800	2.84913700
H	-0.82617300	2.17247100	0.70198200
H	-2.69440000	3.34793100	3.52448400
H	-2.27348200	5.48635300	4.70330300
H	-0.71649600	7.17731400	3.71743100
H	0.38959800	6.69625700	1.53666200
H	-1.79139300	-3.92480700	1.51965100
H	-1.70002100	-2.43114500	2.45989000
C	0.23935900	-1.79681100	-0.22839900
O	-0.94991100	-1.51717500	-0.70589800
O	0.44289500	-2.25494300	0.87444900
C	1.32221200	-1.59786800	-1.27459600
C	1.28972400	-0.27752500	-2.09807000
C	0.46911300	0.80301600	-1.44177900
O	-0.10361700	1.62433200	-2.30532200
O	0.35970500	0.92505600	-0.23970600
C	2.83473500	0.07581000	-2.27525200
C	3.38800500	-1.35738400	-2.27783400
C	2.82318700	-1.76502900	-0.89672300
C	3.16870800	-0.55616500	-0.01205200
C	3.26519000	0.59202700	-0.89978400
H	-1.75222300	-1.81648900	-0.02412900
H	1.11255500	-2.41497900	-1.98329700
H	0.88653500	-0.43609800	-3.10620600
H	-0.58699300	2.32127200	-1.82233200
H	2.94322800	-1.96002800	-3.08113200
H	4.47700900	-1.41166200	-2.36647700
C	3.24330400	-3.11296300	-0.34633400
H	3.05930900	-3.89546700	-1.09693100
H	4.31848000	-3.10601900	-0.10728900
H	2.67699800	-3.36156100	0.56000800

C	3.15591100	0.97550100	-3.44794400
H	4.24097200	1.14797700	-3.50162900
H	2.82996400	0.50729700	-4.38713000
H	2.65236800	1.94922700	-3.35834100
C	3.16931200	2.04203200	-0.53405500
H	3.28819400	2.19873800	0.54489000
H	3.94633100	2.60698900	-1.06576000
H	2.19907900	2.45287400	-0.84000800
C	2.85366600	-0.48586300	1.44622500
H	3.30878300	0.39825700	1.90793700
H	1.76117600	-0.42700000	1.54525300
H	3.20306300	-1.39007800	1.95998400
Br	5.19740700	-0.08848000	-0.31934300
N	7.39537200	-1.47164300	0.42988400
C	8.08327300	-2.53786700	0.92624100
O	7.59612800	-3.56633400	1.36147200
C	9.60152900	-2.27464300	0.87640200
C	9.70833400	-0.93405800	0.16100700
C	8.24112000	-0.52502900	-0.01708100
O	7.88602200	0.53982400	-0.52448700
H	10.09695500	-3.10911800	0.36153400
H	9.98196700	-2.25531500	1.90798600
H	10.23952400	-0.15140600	0.72018300
H	10.17507800	-0.99916300	-0.83271200

10. References

[1] L. Zhou, C. K. Tan, X. Jiang, F. Chen, Y. Y. Yeung, *J. Am. Chem. Soc.* **2010,** *132*, 15474-6.

[2] S. E. Border, R. Z. Pavlovic, L. Zhiquan, J. D. Badjic, *J. Am. Chem. Soc.* **2017,** *139*, 18496-18499.

[3] T.-L. Ho, W.-L. Yeh, J. Yule, H.-J. Liu, *Can. J. Chem.* **1992**, *70*, 1375-1384.

[4] C. Reynaud, Y. Fall, M. Feuerstein, H. Doucet, M. Santelli, *Tetrahedron* **2009,** *65*, 7440-7448.

[5] K. Chajara, H. Ottosson, *Tetrahedron Lett.* **2004,** *45*, 6741-6744.

[6] H. Kurata, T. Ekinaka, T. Kawase, M. Kawase, *Tetrahedron Lett.* **1993,** *34*, 3445-3448.

[7] M.-M. Xu, L. Yang, K. Tan, X. Chen, Q.-T. Lu, K. N. Houk, Q. Cai, *Nat. Catal.* **2021,** *4*, 892-900.

[8] P. Sieverding, J. Osterbrink, C. Besson, P. Kogerler, *J. Org. Chem.* **2019,** *84*, 486-494.

[9] K. J. Stone, R. D. Little, *J. Org. Chem.* **2002,** *49*, 1849-1853.

[10] Z. Wang, X. Wang, P. Wang, J. Zhao, *J. Am. Chem. Soc.* **2021,** *143*, 10374-10381.

[11] M. J. Frisch, G. W. Trucks, H. B. Schlegel, G. E. Scuseria, M. A. Robb, J. R. Cheeseman, G. Scalmani, V. Barone, G. A. Petersson, H. Nakatsuji, X. Li, M. Caricato, A. V. Marenich, J. Bloino, B. G. Janesko, R. Gomperts, B. Mennucci, H. P. Hratchian, J. V. Ortiz, A. F. Izmaylov, J. L. W. Sonnenberg, F. Ding, F. Lipparini, F. Egidi, J. Goings, B. Peng, A. Petrone, T. Henderson, D. Ranasinghe, V. G. Zakrzewski, J. Gao, N. Rega, G. Zheng, W. Liang, M. Hada, M. Ehara, K. Toyota, R. Fukuda, J. Hasegawa, M. Ishida, T. Nakajima, Y. Honda, O. Kitao, H. Nakai, T. Vreven, K. Throssell, J. A. Montgomery Jr., J. E. Peralta, F. Ogliaro, M. J. Bearpark, J. J. Heyd, E. N. Brothers, K. N. Kudin, V. N. Staroverov, T. A. Keith, R. Kobayashi, J. Normand, K. Raghavachari, A. P. Rendell, J. C. Burant, S. S. Iyengar, J. Tomasi, M. Cossi, J. M. Millam, M. Klene, C. Adamo, R. Cammi, J. W. Ochterski, R. L. Martin, K. Morokuma, O. Farkas, J. B. Foresman, D. J. Fox, *Gaussian 16*, C.02.; Gaussian, Inc.: Wallingford, CT, 2016.

[12] F. Neese, *Wiley Interdiscip. Rev.: Comput. Mol. Sci.* **2012**, *2*, 73–7.

[13] V. Barone, M. Cossi, *J. Phys. Chem. A* **1998**, *102*, 1995–2001.

[14] P. J. Stephens, F. J. Devlin, C. F. Chabalowski, M. J. Frisch, *J. Phys. Chem.* **1994**, *98*, 11623–11627.

[15] F. Weigend, R. Ahlrichs, *Phys. Chem. Chem. Phys.* **2005**, *7*, 3297–3305.

[16] S. Grimme, J. Antony, S. Ehrlich, H. Krieg, *J. Chem. Phys.* **2010**, *132*, 154104.

[17] S. Grimme, S. Ehrlich, L. Goerigk, *J. Comput. Chem.* **2011**, *32*, 1456–1465.

[18] J.-D. Chai, M. Head-Gordon, *J. Chem. Phys*. **2009**, 131, 174105.

[19] F. Weigend, F. Furche, R. Ahlrichs, *J. Chem. Phys.* **2003**, *119*, 12753–12762.

[20] S. Grimme, S. Ehrlich, L. Goerigk, *J. Comput. Chem.* **2011**, *32*, 1456–1465.

[21] F. Weigend, *Phys. Chem. Chem. Phys.* **2006**, *8*, 1057–1065.

[22] C. Bannwarth, E. Caldeweyher, S. Ehlert, A. Hansen, P. Pracht, J. Seibert, S. Spicher, S. Grimme, *WIREs Comput. Mol. Sci.* **2021**, *11*, e1493.

[23] C. Bannwarth, S. Ehlert, S. Grimme, *J. Chem. Theory Comput.,* **2019**, *15*, 1652–1671.

[24] T. Lu, Q. Chen, *Comput. Theor. Chem.* **2021**, *1200*, 113249.

[25] A. A. Otlyotov, Y. Minenkov, *J. Comput. Chem.* **2023**, *44*, 1807–1816.

[26] M. K. Kesharwani, B. Brauer, J. M. L. Martin, *J. Phys. Chem. A* **2015**, *119*, 1701–1714

[27] A. V. Marenich, C. J. Cramer, D. G. Truhlar, *J. Phys. Chem. B* **2009**, *113*, 6378-6396.

[28] A. A. Maryott, E. R. Smith, *Table of Dielectric Constants of Pure Liquids*, U. S. Govt. Print. Off, **1951.**

[29] E. D. Glendening, C. R. Landis, F. Weinhold, *Wiley Interdiscip. Rev.: Comput. Mol. Sci.* **2012**, *2*, 1-42.

[30] E. D. Glendening, C. R. Landis, F. Weinhold, *J. Comput. Chem.* **2019**, *40*, 2234-2241.

[31] E. R. Johnson, S. Keinan, P. Mori-Sánchez, J. Contreras-García, A. J. Cohen, W. Yang, *J. Am. Chem. Soc.* **2010**, *132*, 6498-6506.

[32] R. F. W. Bader, *Atoms in Molecules: A Quantum Theory*, Clarendon Press: Oxford, **1990**.

[33] T. Lu, F. Chen, *J. Comput. Chem.* **2012**, *33*, 580-592.

[34] T. Lu, *J. Chem. Phys.* **2024**, *161*, 082503.

[35] W. Humphrey, A. Dalke, K. Schulten, *J. Mol. Graph.* **1996**, *14*, 33-38.

[36] C. Y. Legault, *CYLview20*, Université de Sherbrooke, **2020**.

11. NMR Spectra

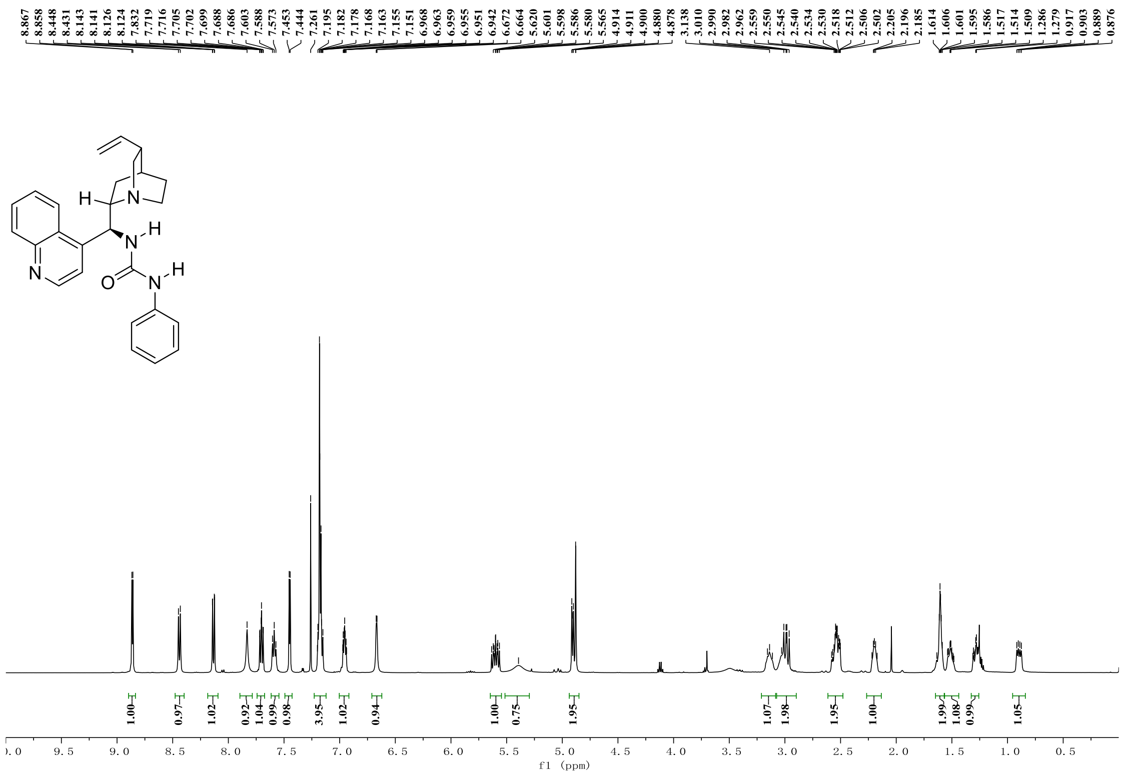

^{1}H NMR was recorded on Bruker 500 MHz; Solvent: $CDCl_3$

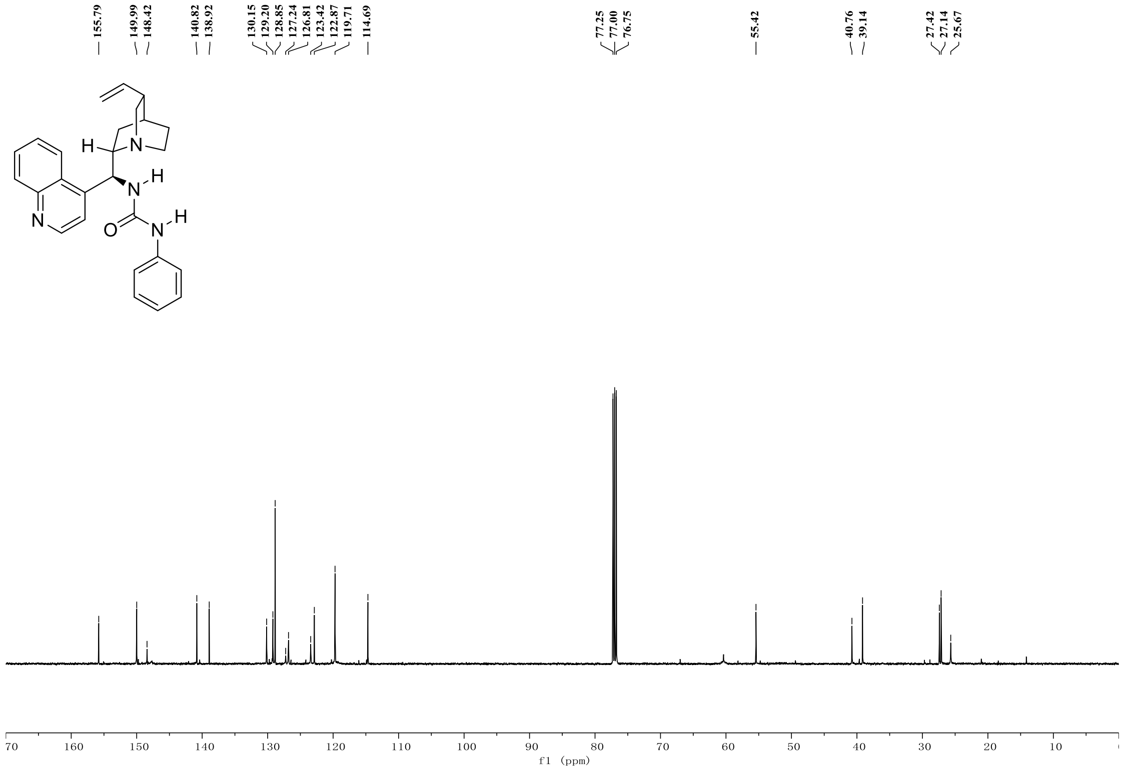

^{13}C NMR was recorded on Bruker 126 MHz; Solvent: $CDCl_3$

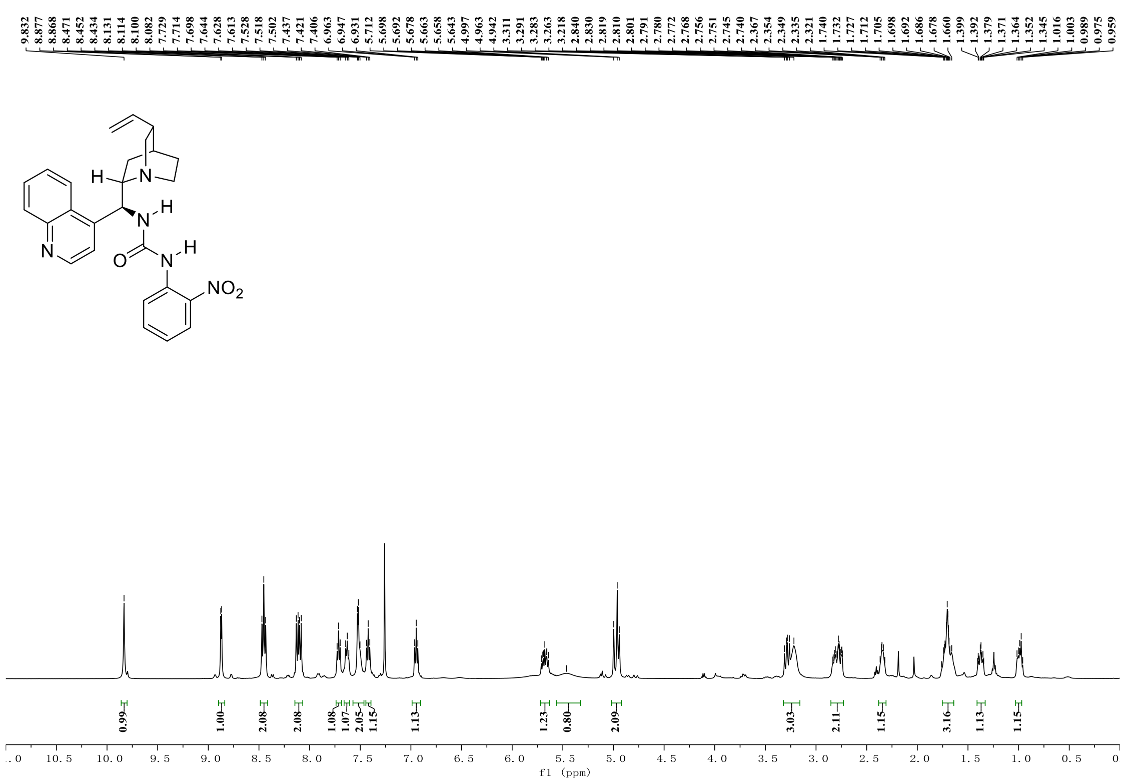

^{1}H NMR was recorded on Bruker 500 MHz; Solvent: $CDCl_3$

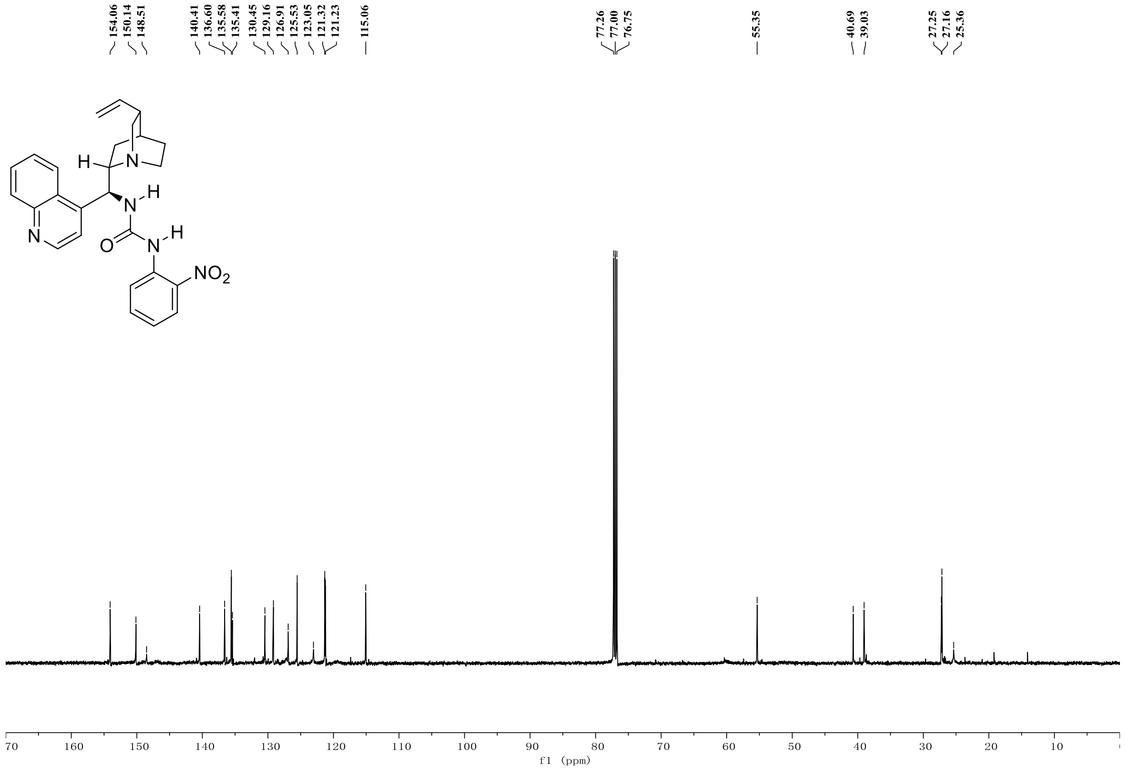

^{13}C NMR was recorded on Bruker 126 MHz; Solvent: $CDCl_3$

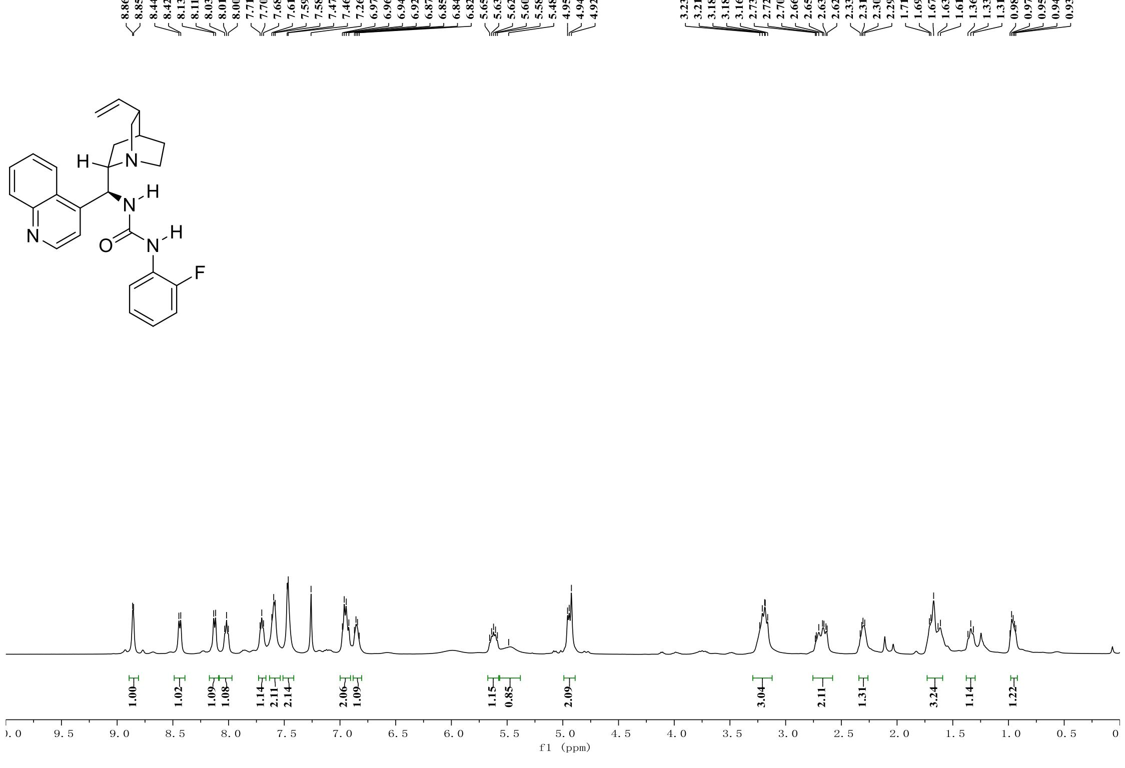

^{1}H NMR was recorded on Bruker 500 MHz; Solvent: $CDCl_3$

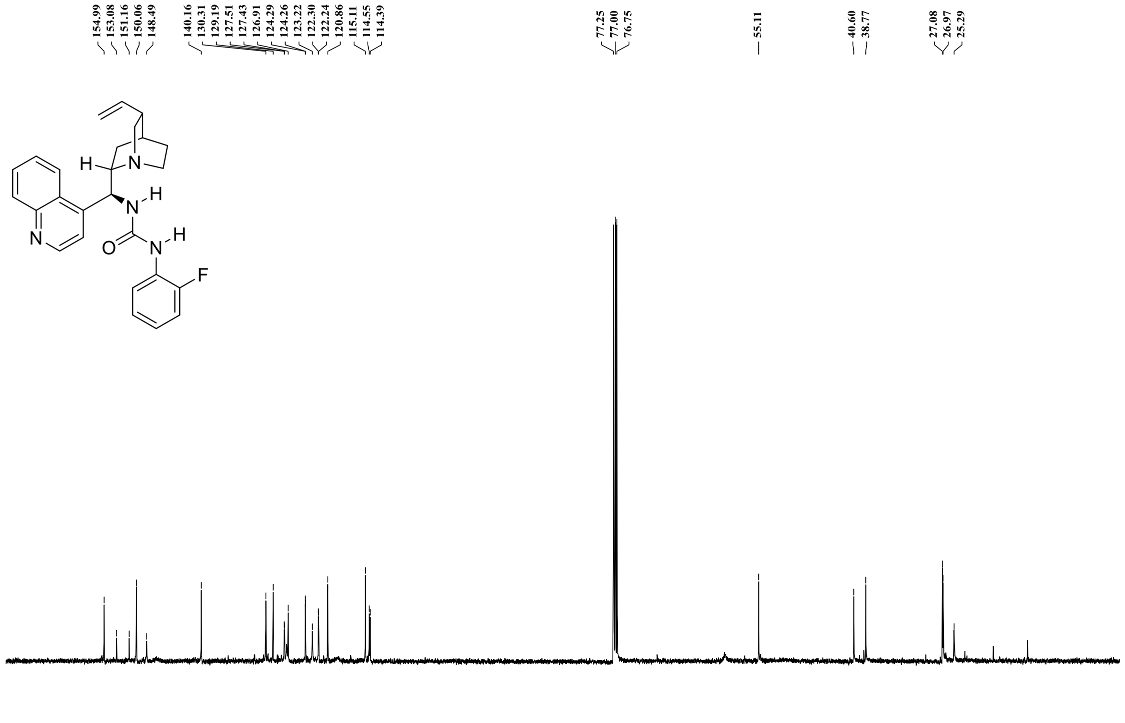

^{13}C NMR was recorded on Bruker 126 MHz; Solvent: $CDCl_3$

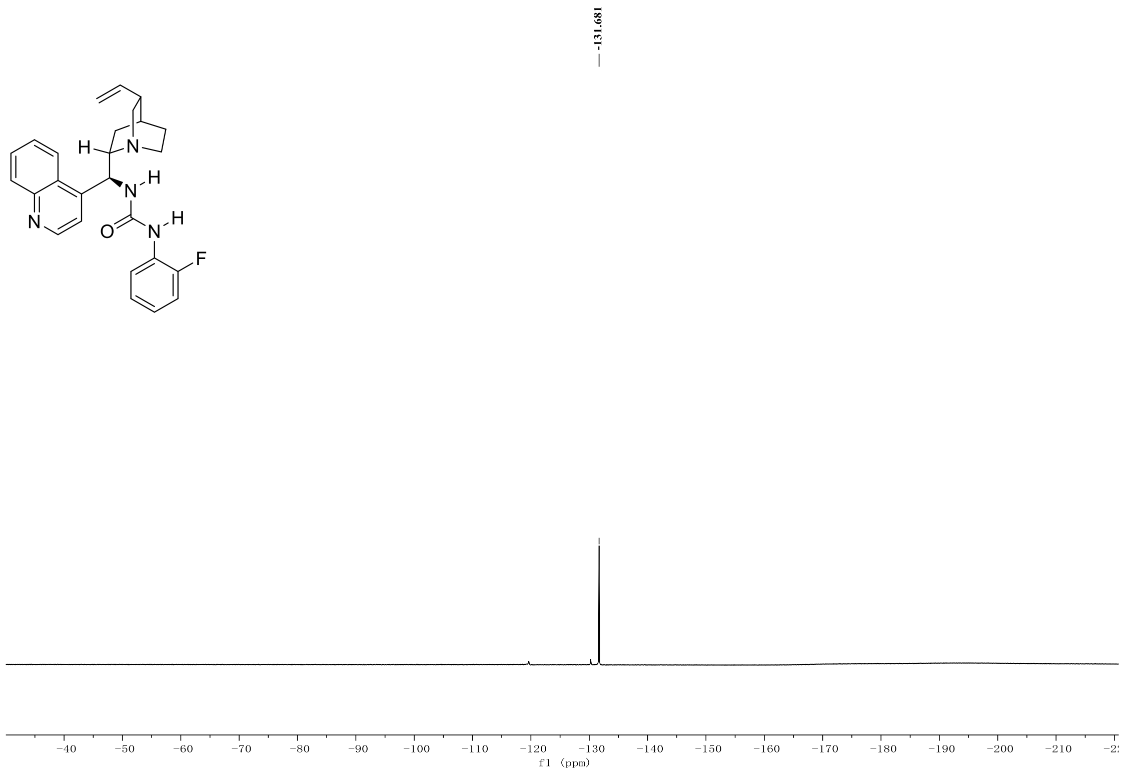

^{19}F NMR was recorded on Bruker 471 MHz; Solvent: $CDCl_3$

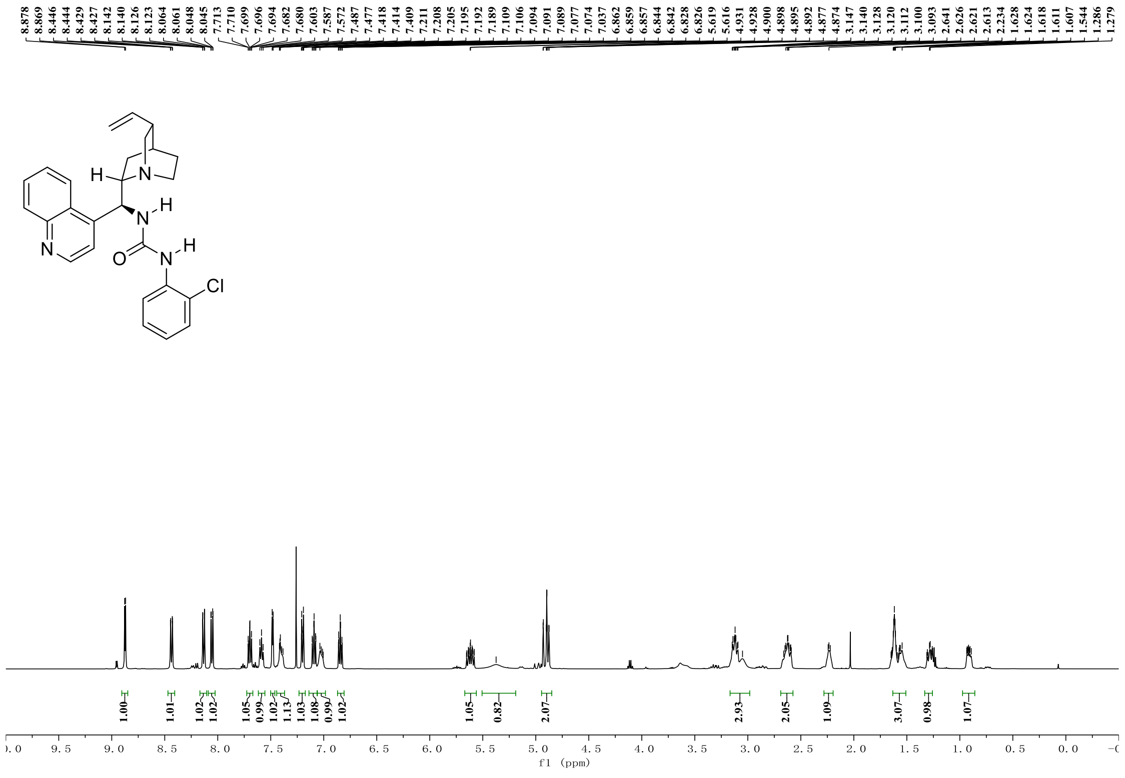

^{1}H NMR was recorded on Bruker 500 MHz; Solvent: $CDCl_3$

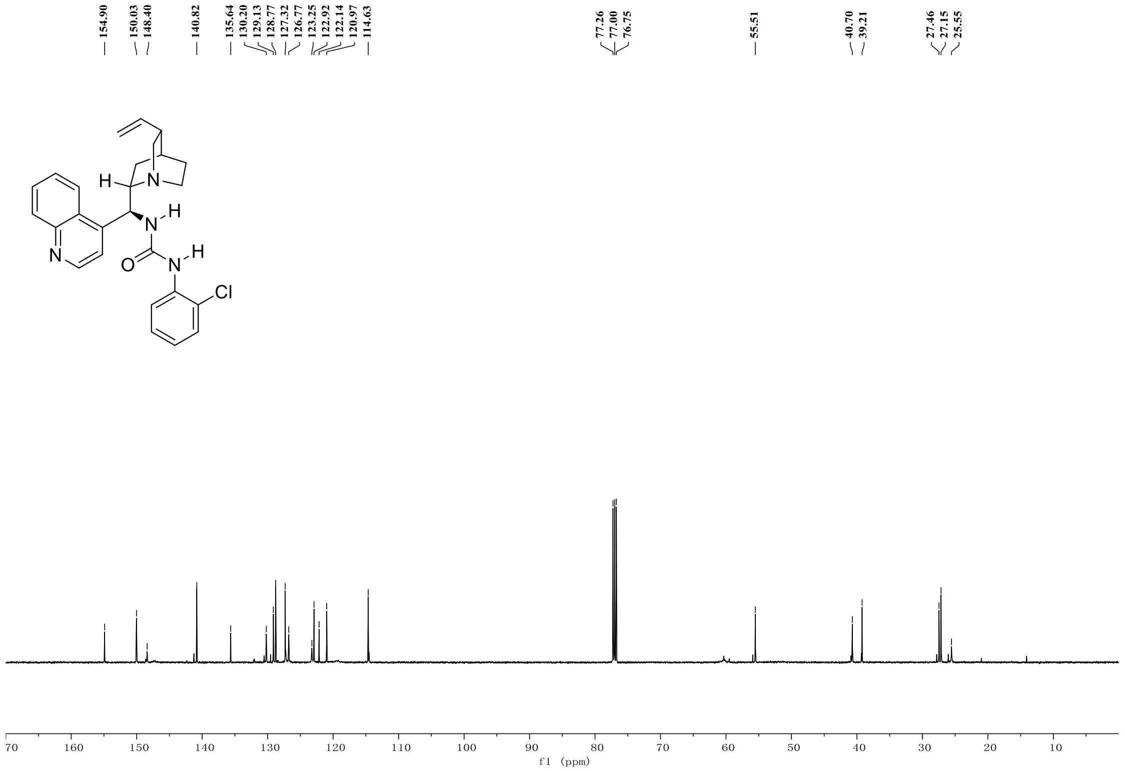

^{13}C NMR was recorded on Bruker 126 MHz; Solvent: $CDCl_3$

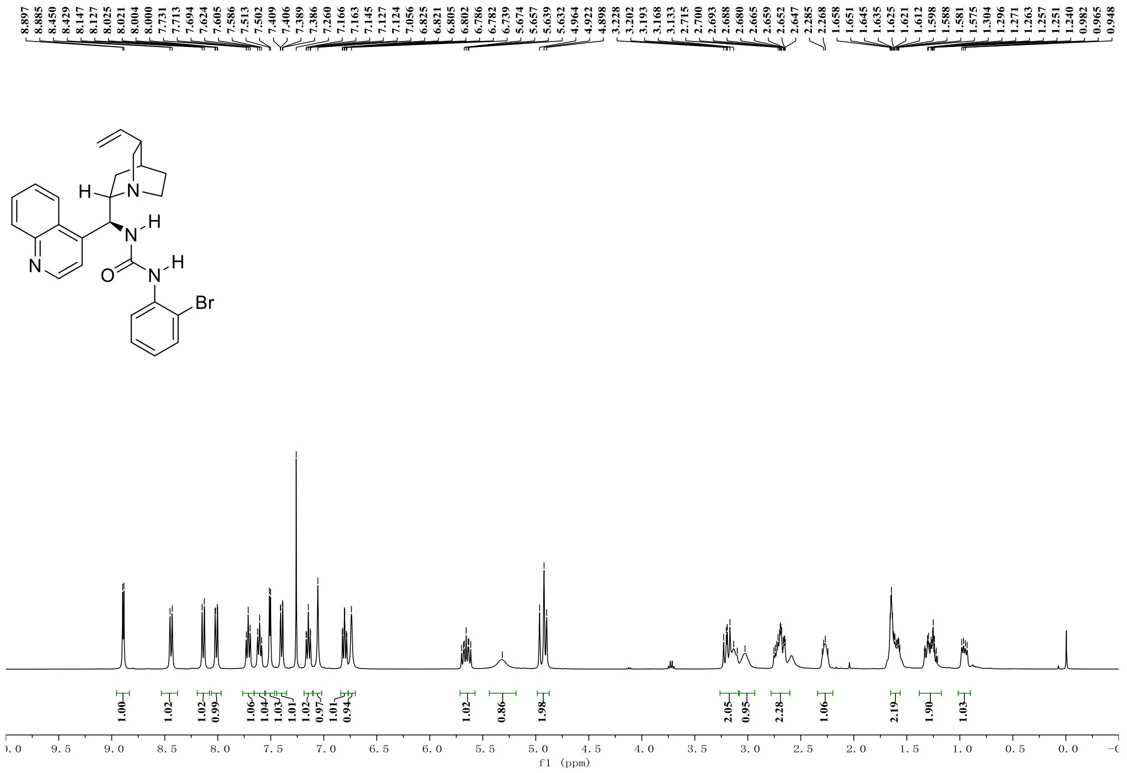

^{1}H NMR was recorded on Bruker 500 MHz; Solvent: $CDCl_3$

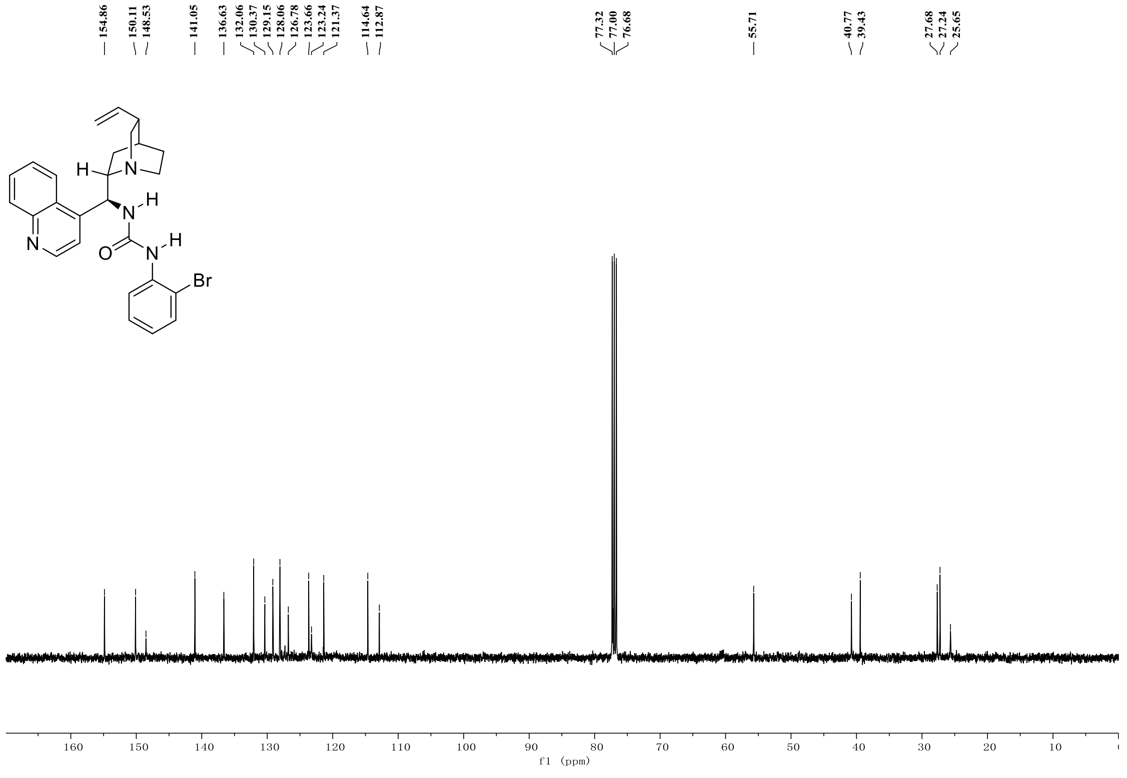

^{13}C NMR was recorded on Bruker 126 MHz; Solvent: $CDCl_3$

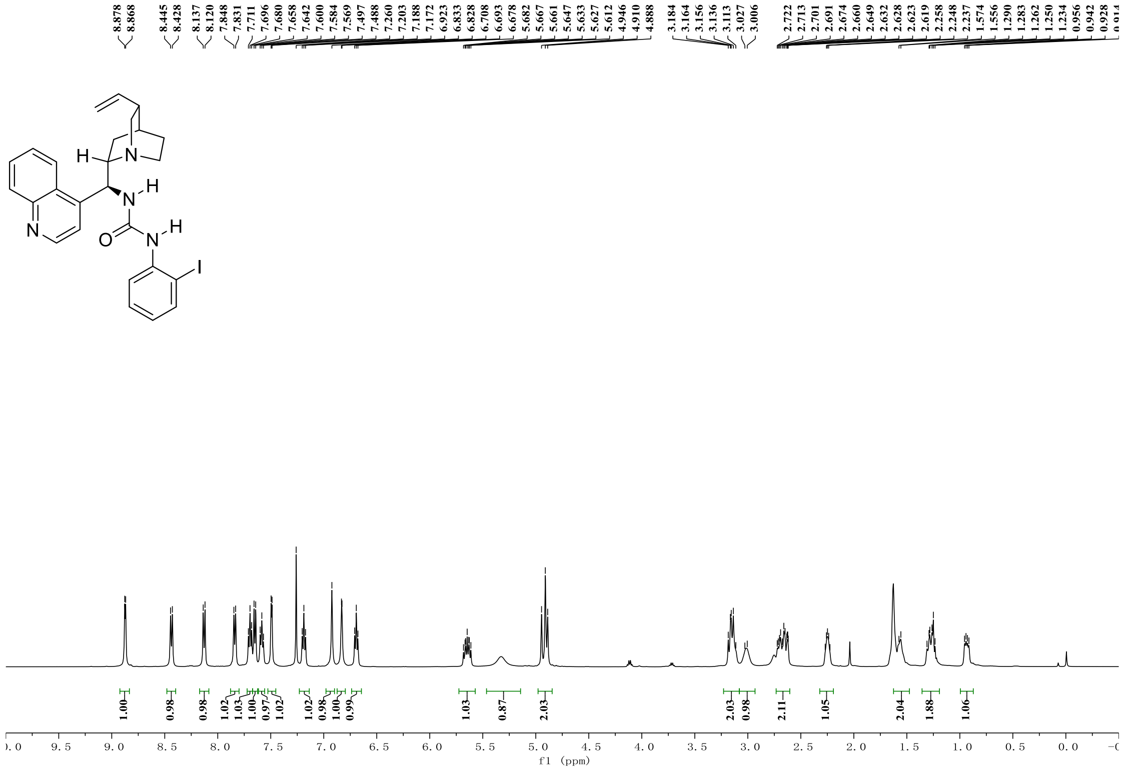

^{1}H NMR was recorded on Bruker 500 MHz; Solvent: $CDCl_3$

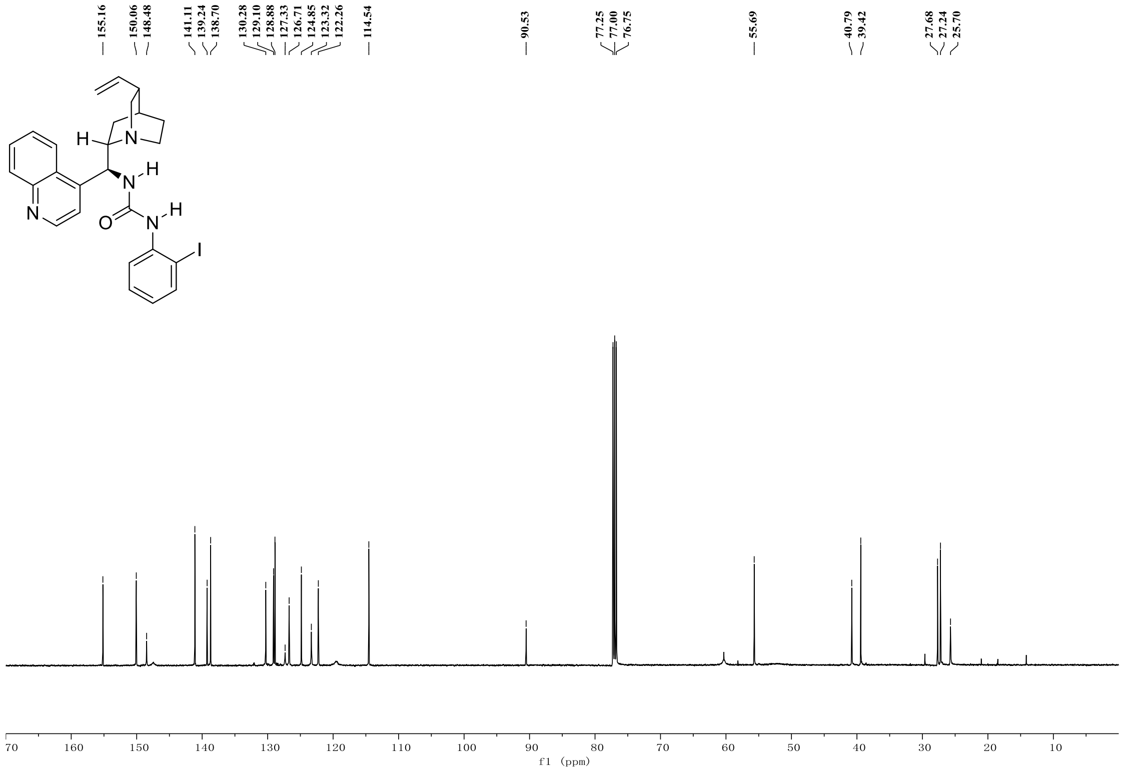

^{13}C NMR was recorded on Bruker 126 MHz; Solvent: $CDCl_3$

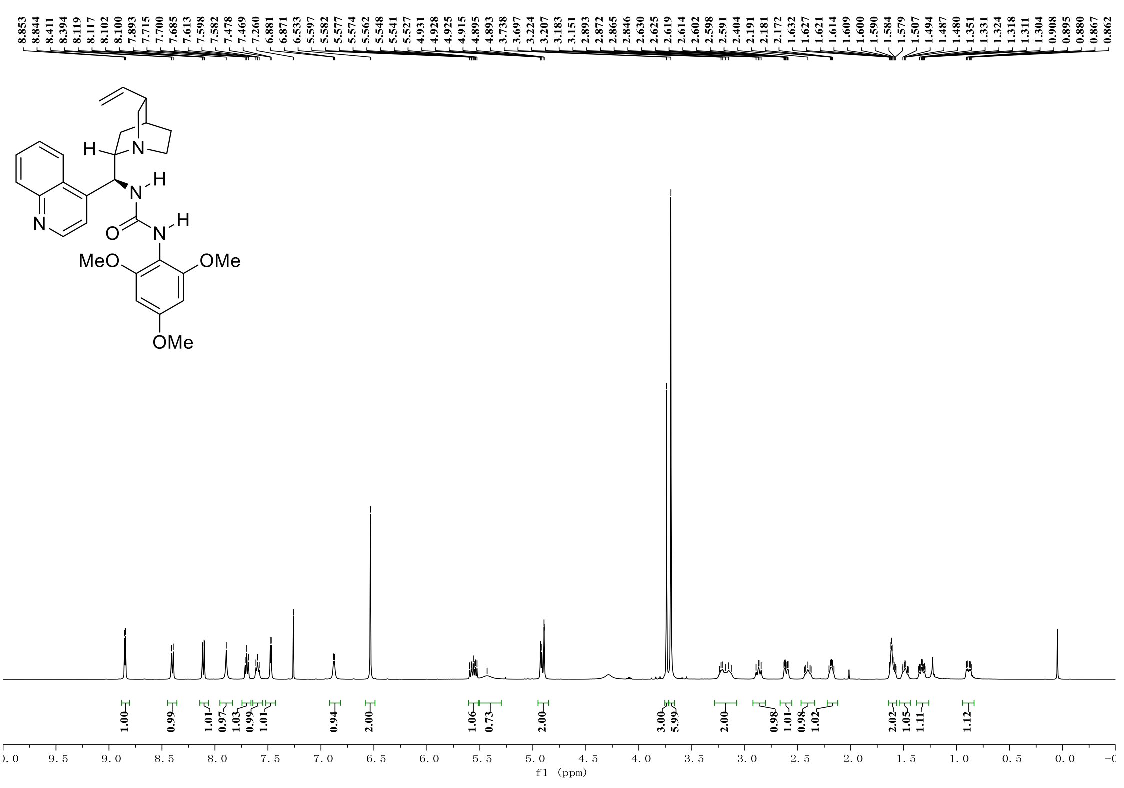

^{1}H NMR was recorded on Bruker 400 MHz; Solvent: $CDCl_3$

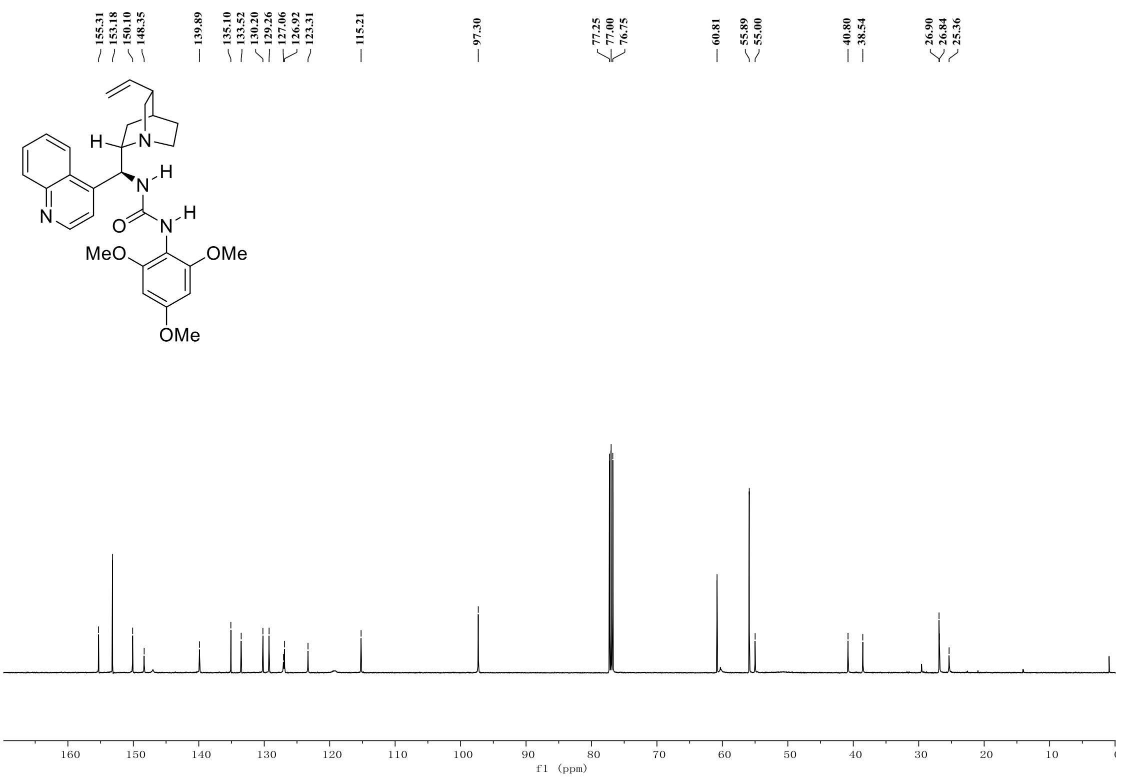

^{13}C NMR was recorded on Bruker 101 MHz; Solvent: $CDCl_3$

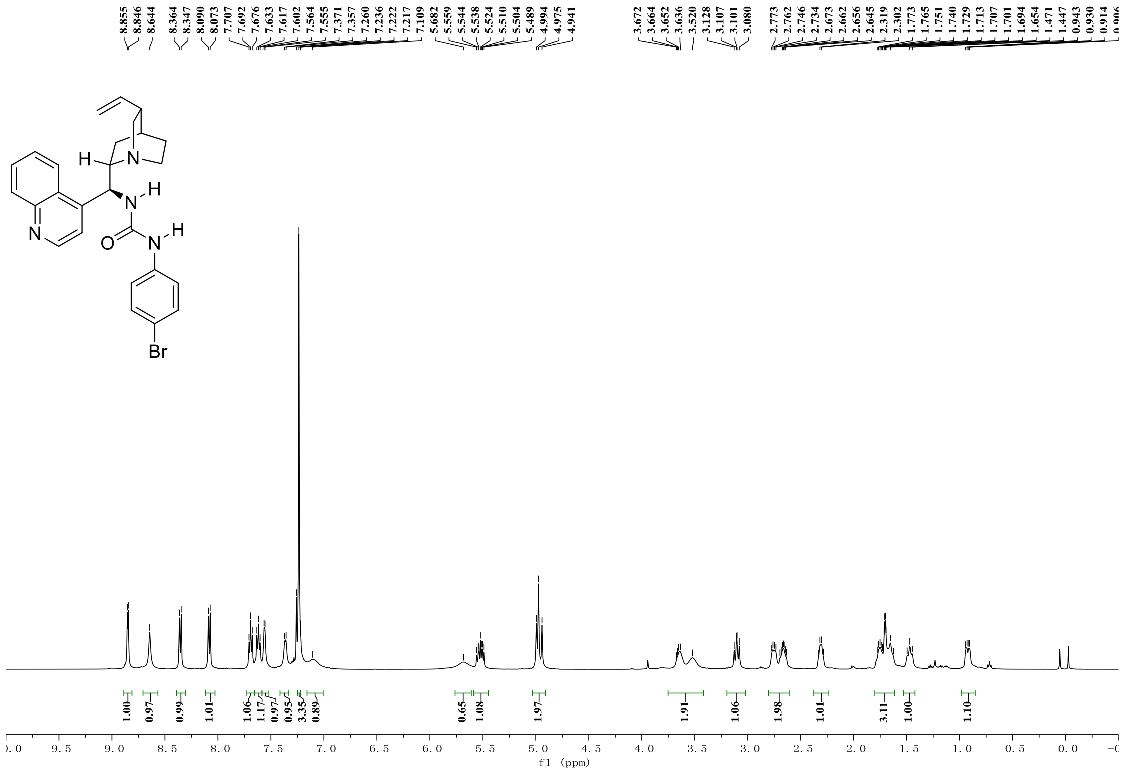

^{1}H NMR was recorded on Bruker 500 MHz; Solvent: $CDCl_3$

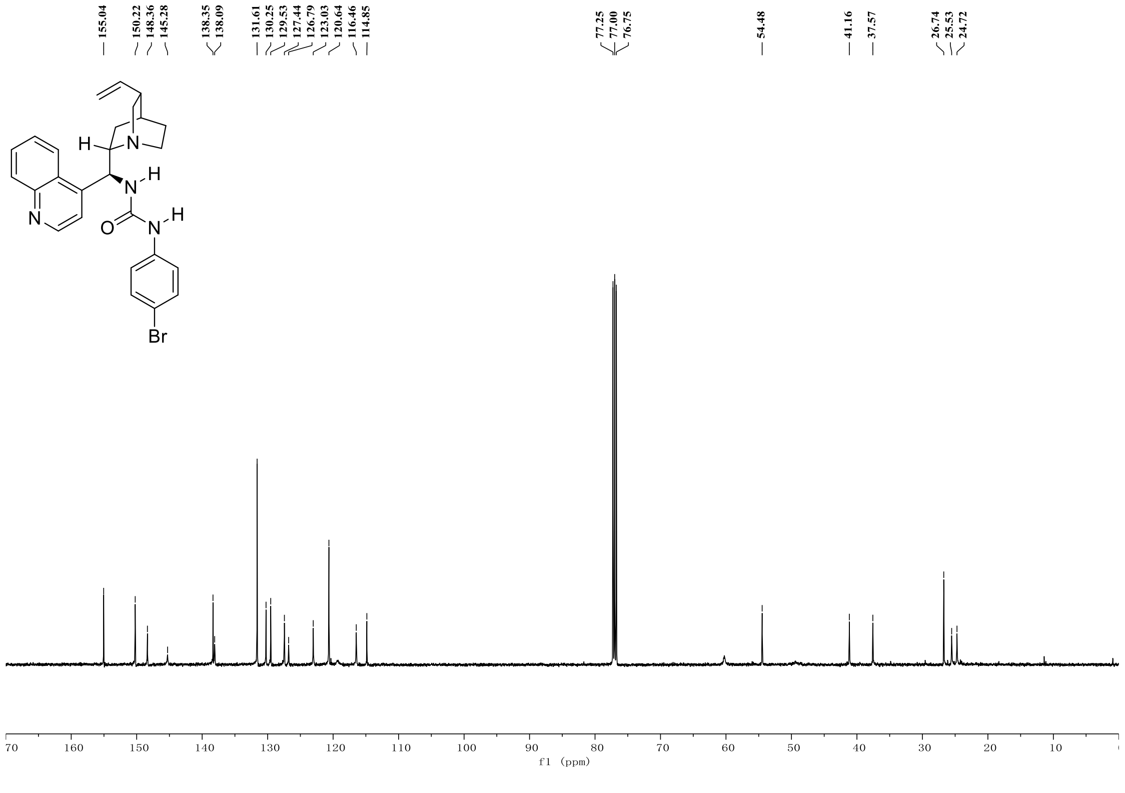

^{13}C NMR was recorded on Bruker 126 MHz; Solvent: $CDCl_3$

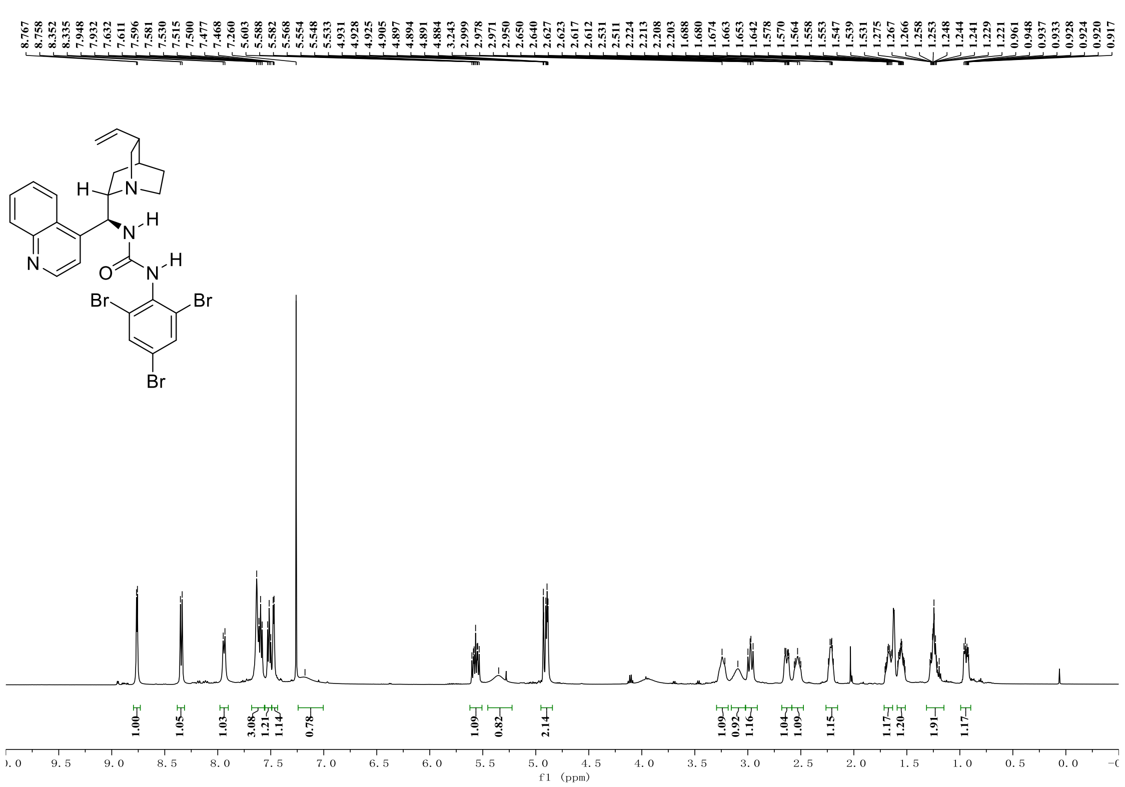

^{1}H NMR was recorded on Bruker 500 MHz; Solvent: $CDCl_3$

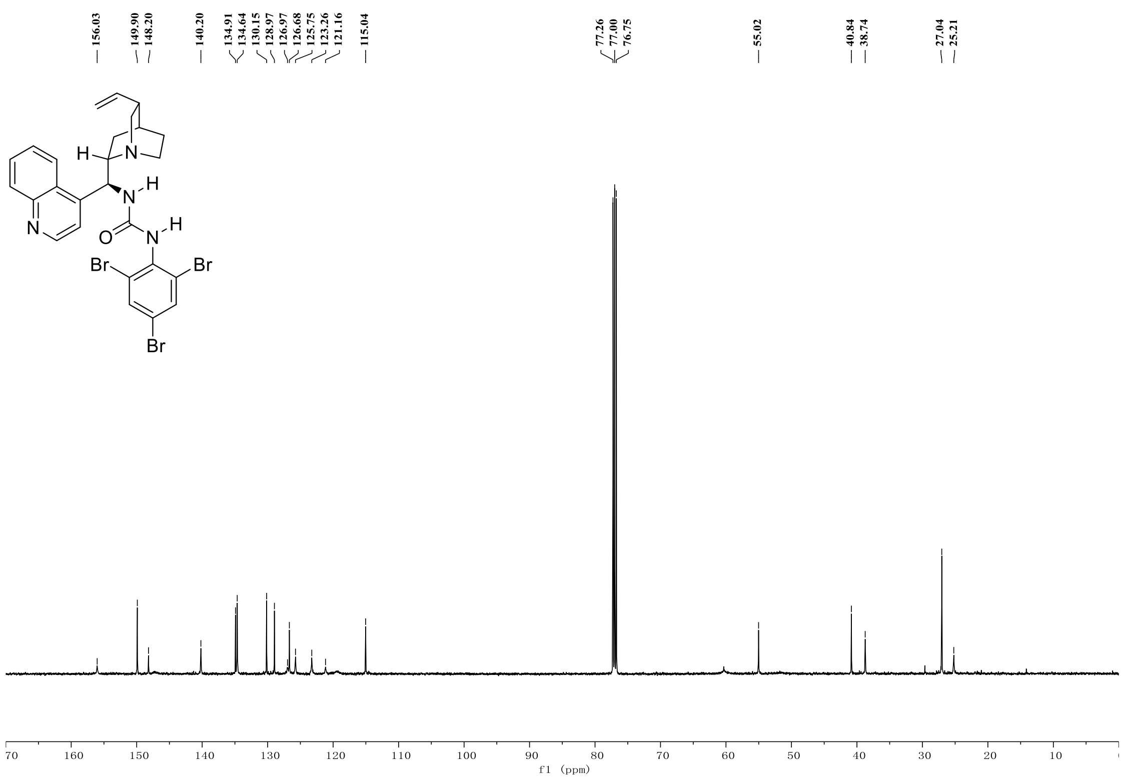

^{13}C NMR was recorded on Bruker 126 MHz; Solvent: $CDCl_3$

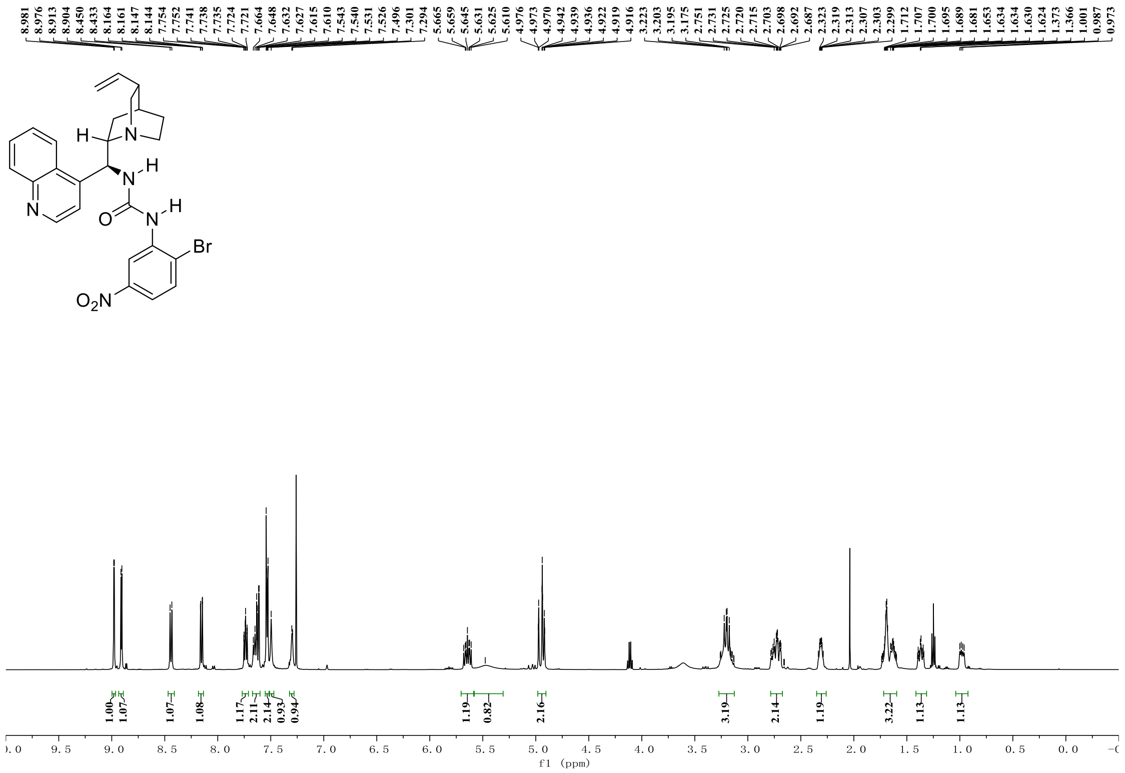

^{1}H NMR was recorded on Bruker 500 MHz; Solvent: $CDCl_3$

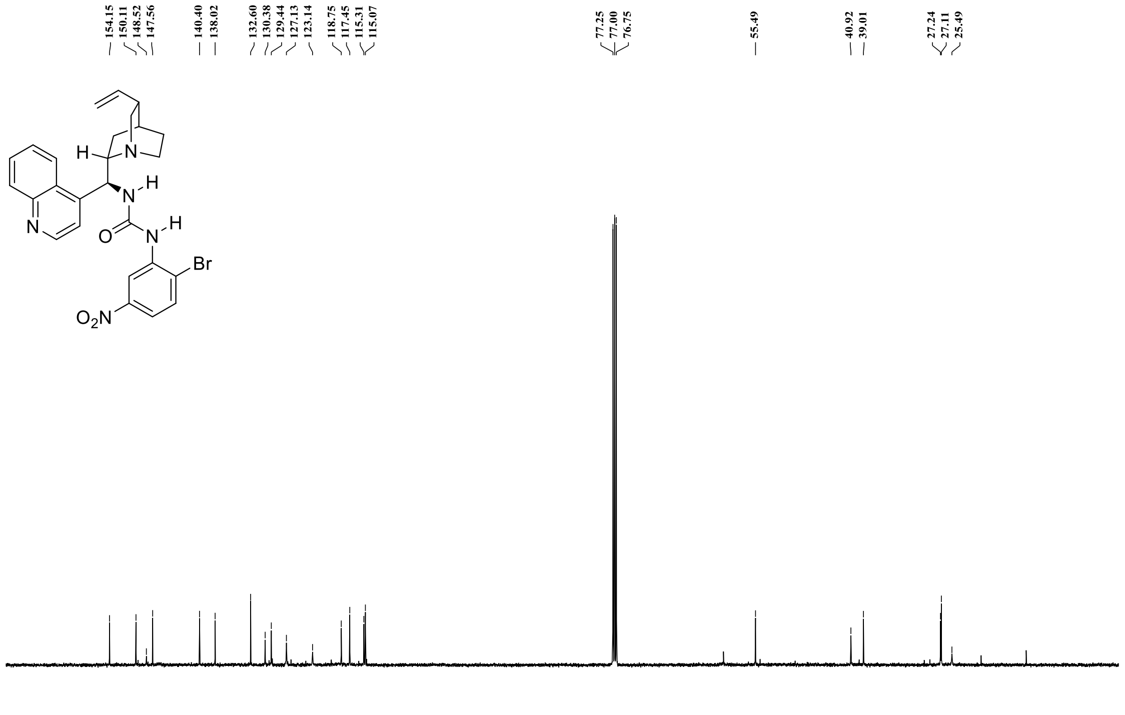

^{13}C NMR was recorded on Bruker 126 MHz; Solvent: $CDCl_3$

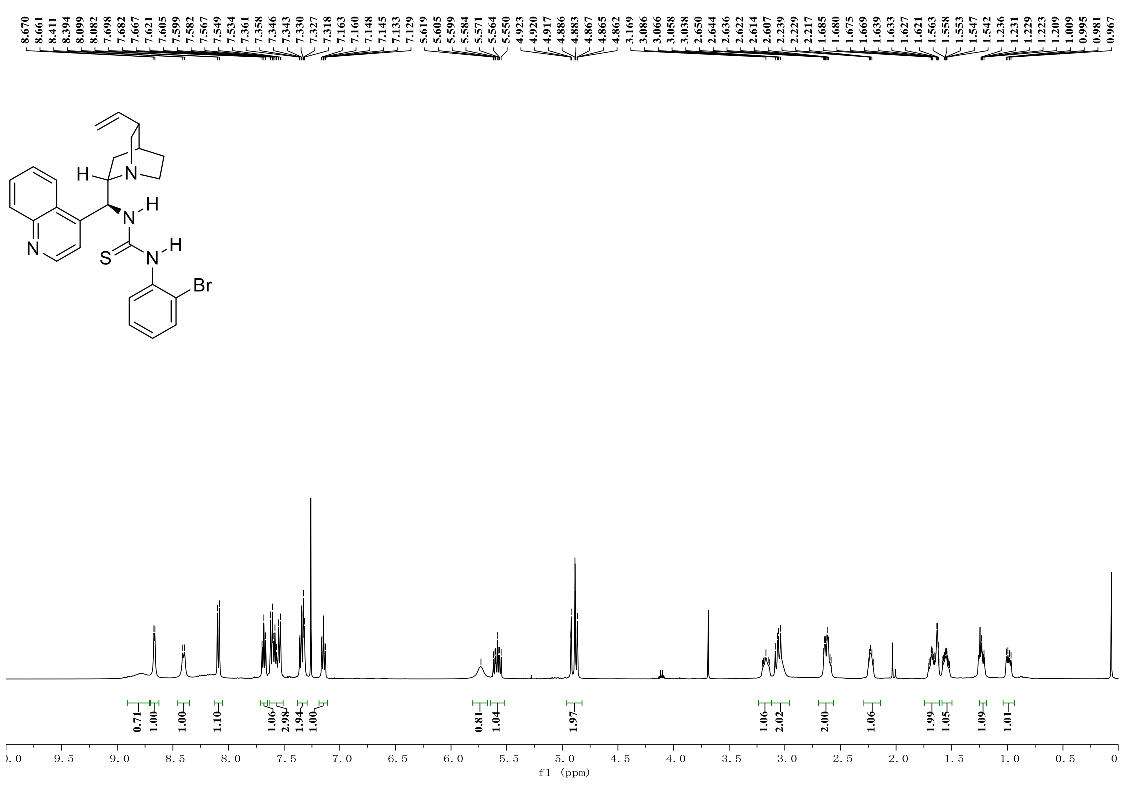

^{1}H NMR was recorded on Bruker 500 MHz; Solvent: $CDCl_3$

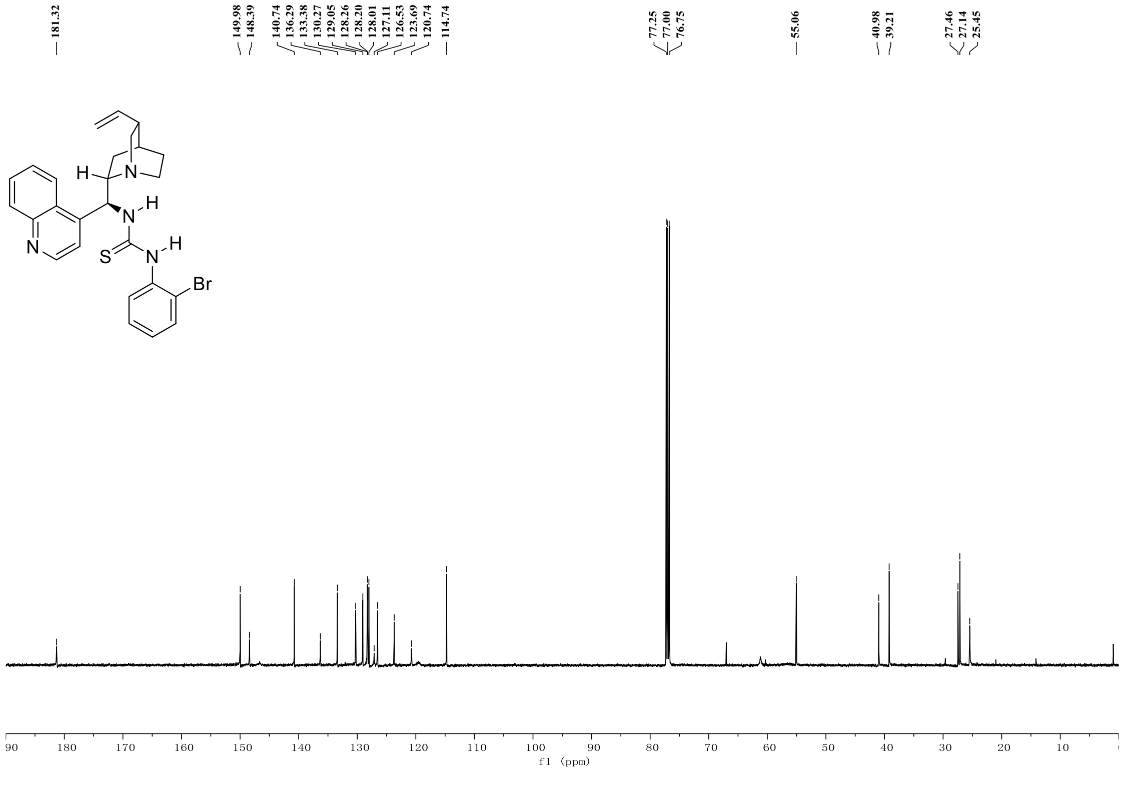

^{13}C NMR was recorded on Bruker 126 MHz; Solvent: $CDCl_3$

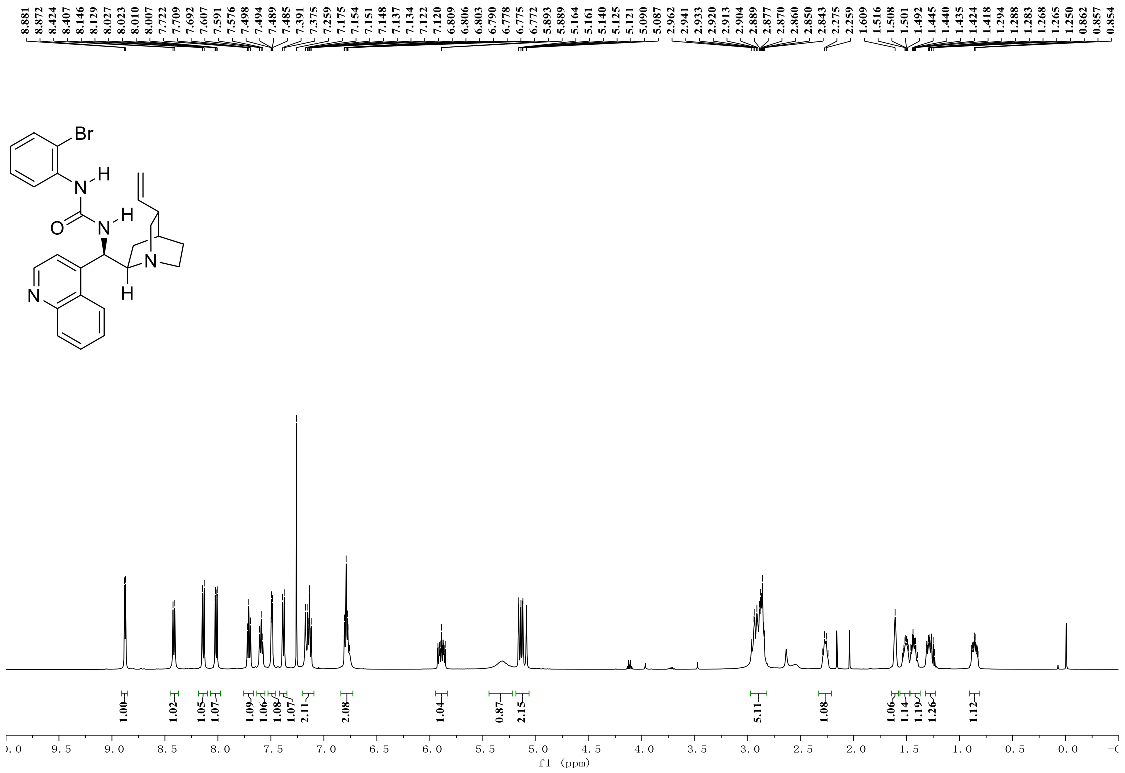

^{1}H NMR was recorded on Bruker 500 MHz; Solvent: $CDCl_3$

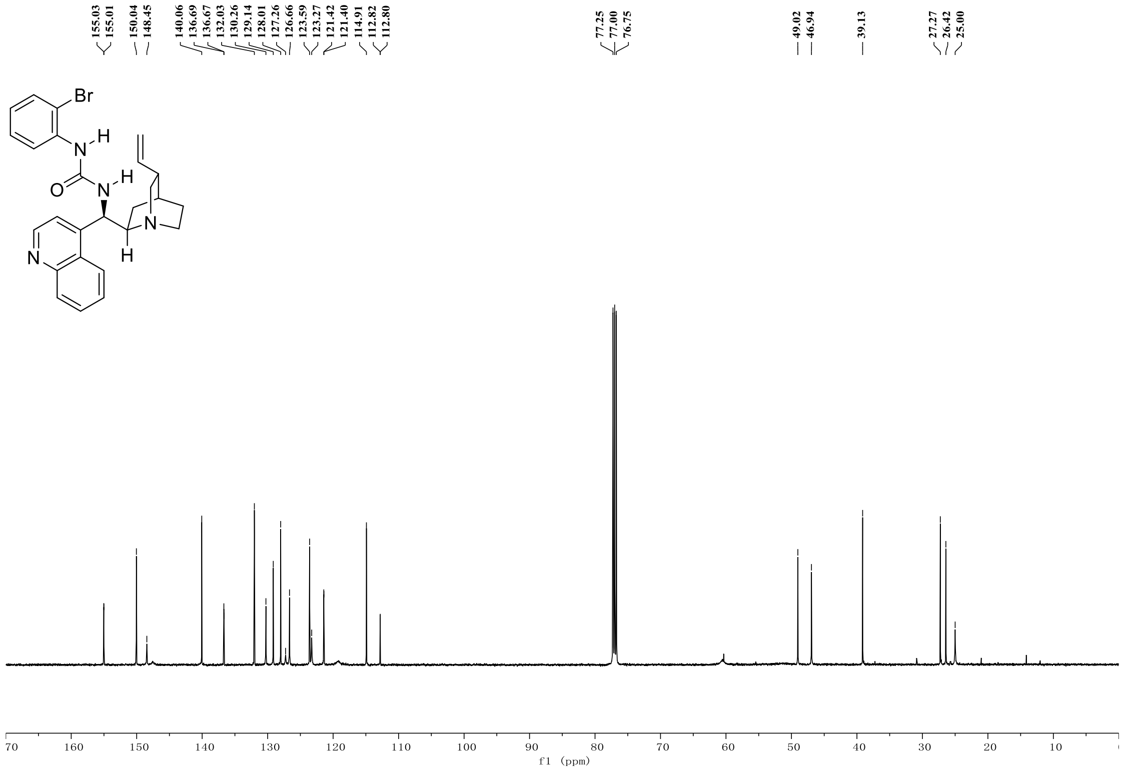

^{13}C NMR was recorded on Bruker 126 MHz; Solvent: $CDCl_3$

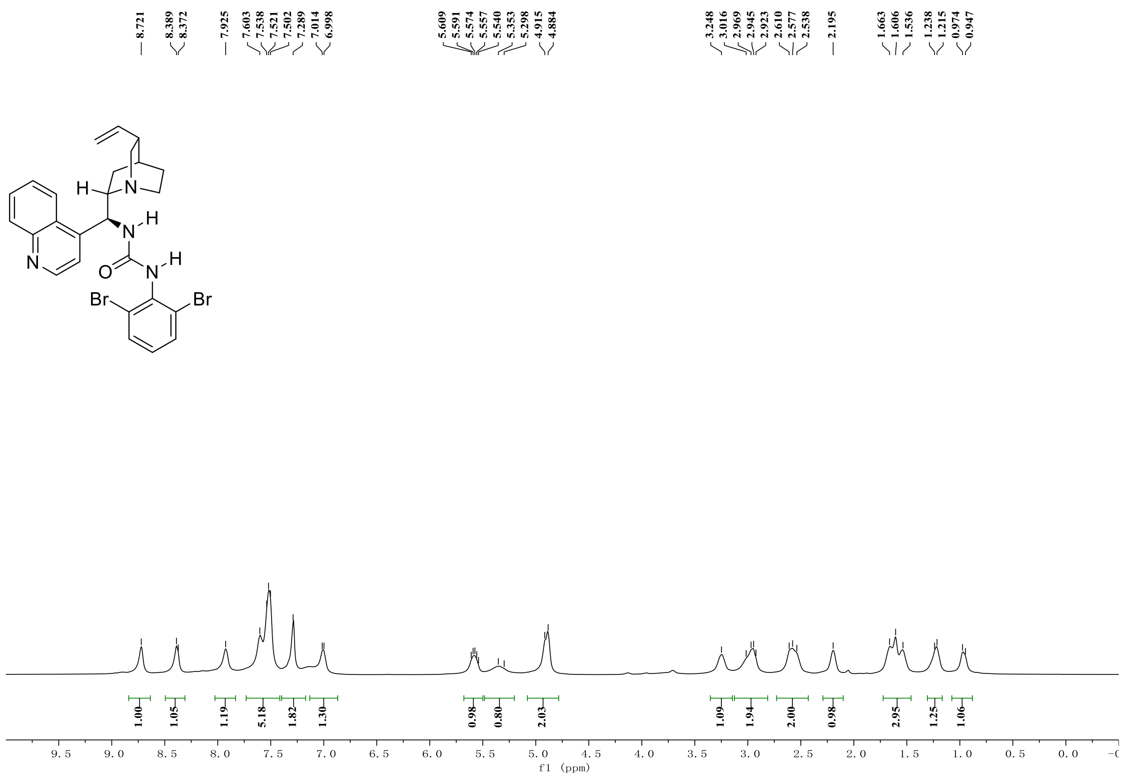

^{1}H NMR was recorded on Bruker 500 MHz; Solvent: $CDCl_3$

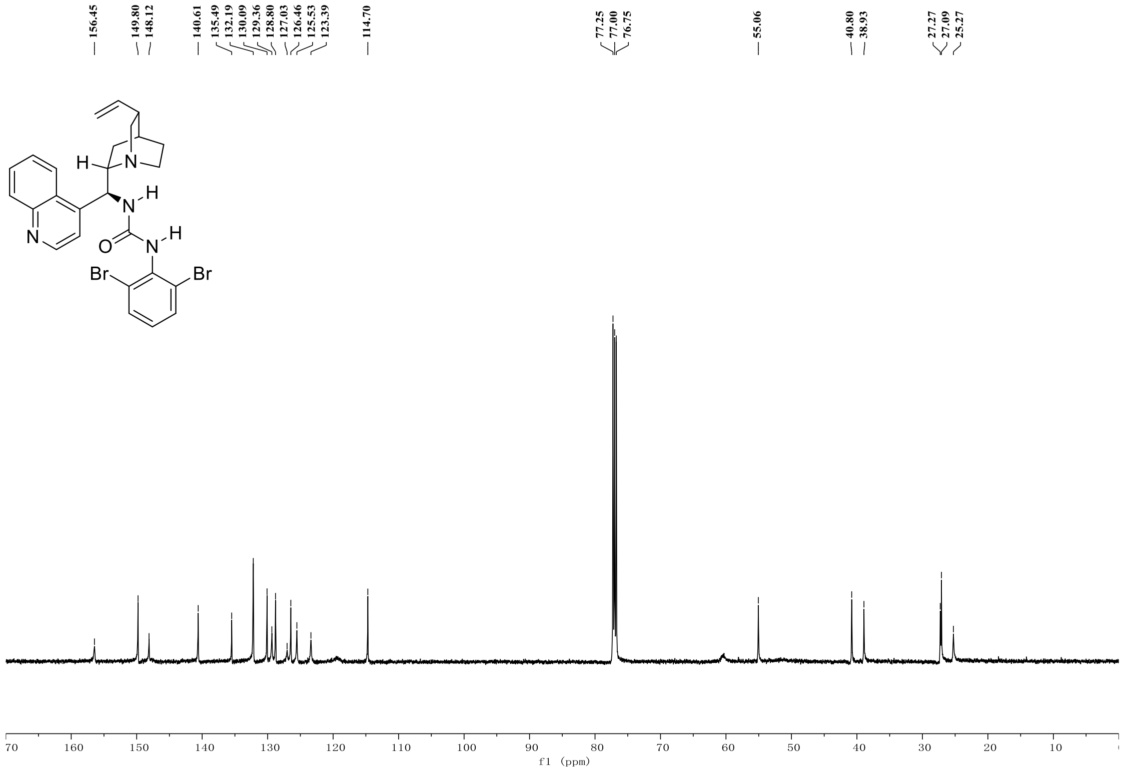

^{13}C NMR was recorded on Bruker 126 MHz; Solvent: $CDCl_3$

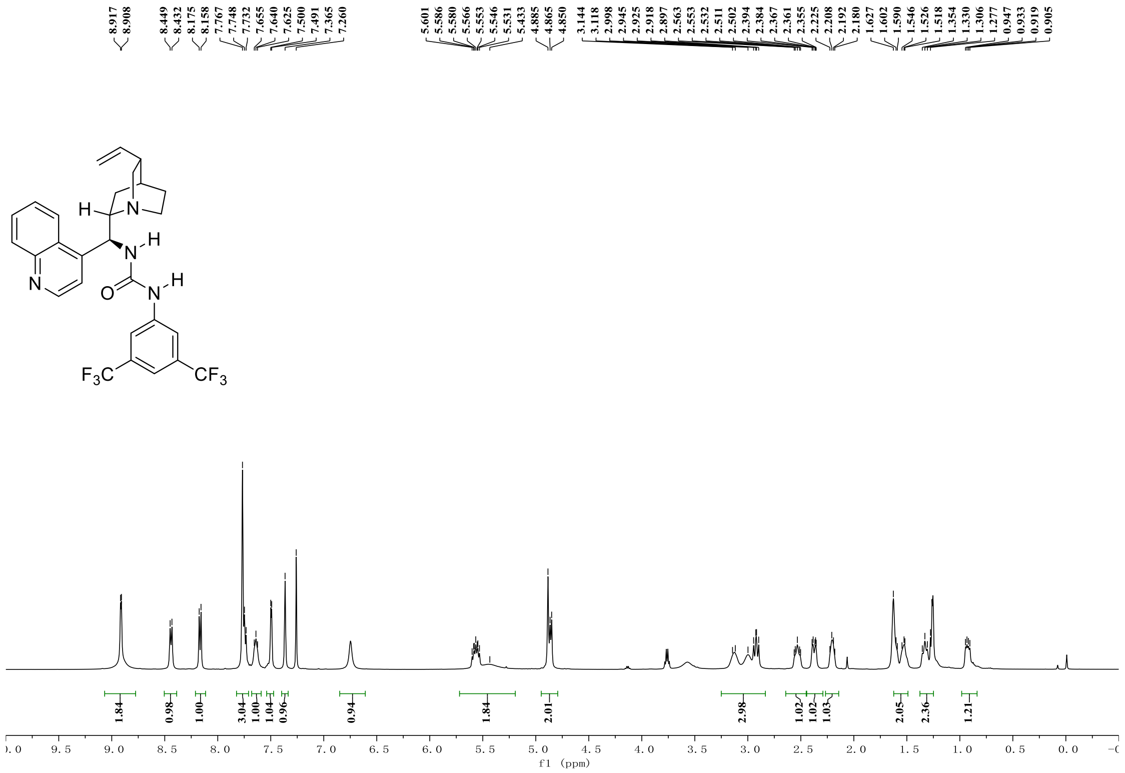

^{1}H NMR was recorded on Bruker 500 MHz; Solvent: $CDCl_3$

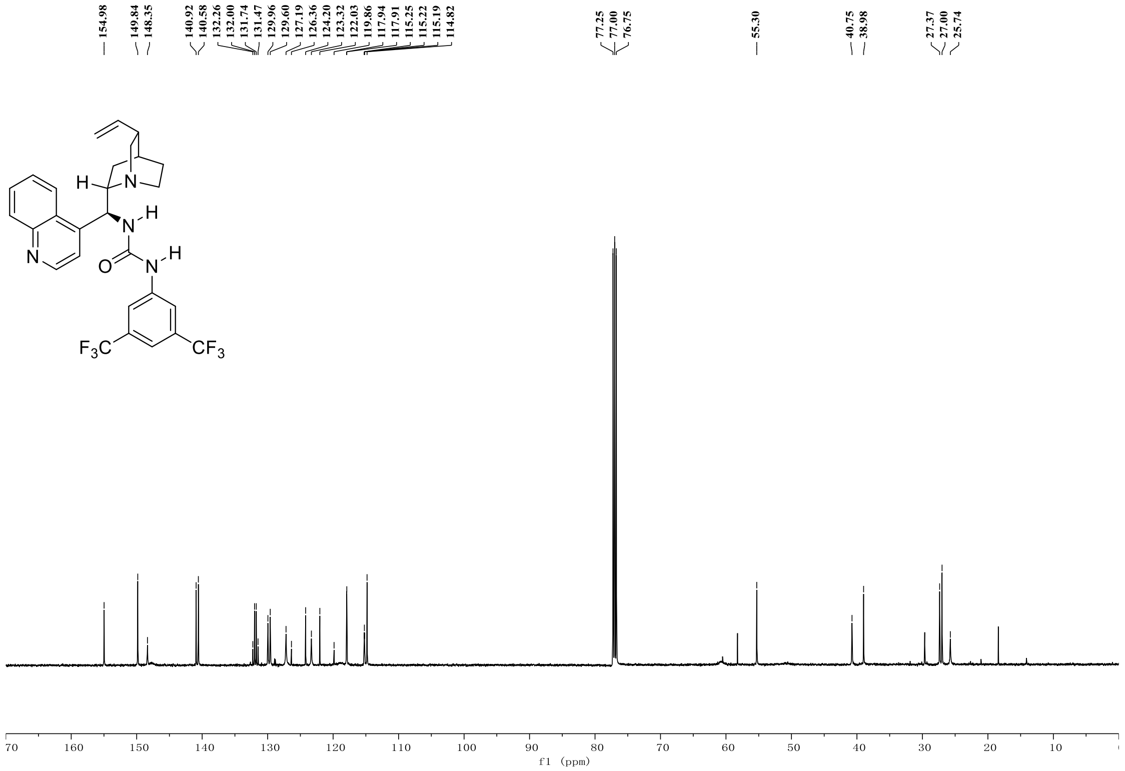

^{13}C NMR was recorded on Bruker 126 MHz; Solvent: $CDCl_3$

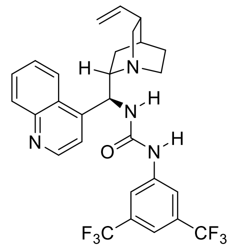

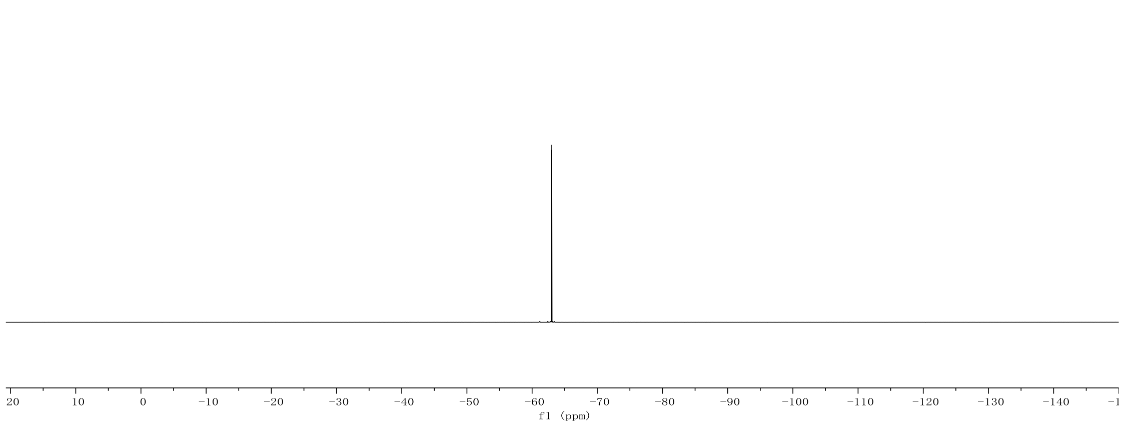

^{19}F NMR was recorded on Bruker 471 MHz; Solvent: $CDCl_3$

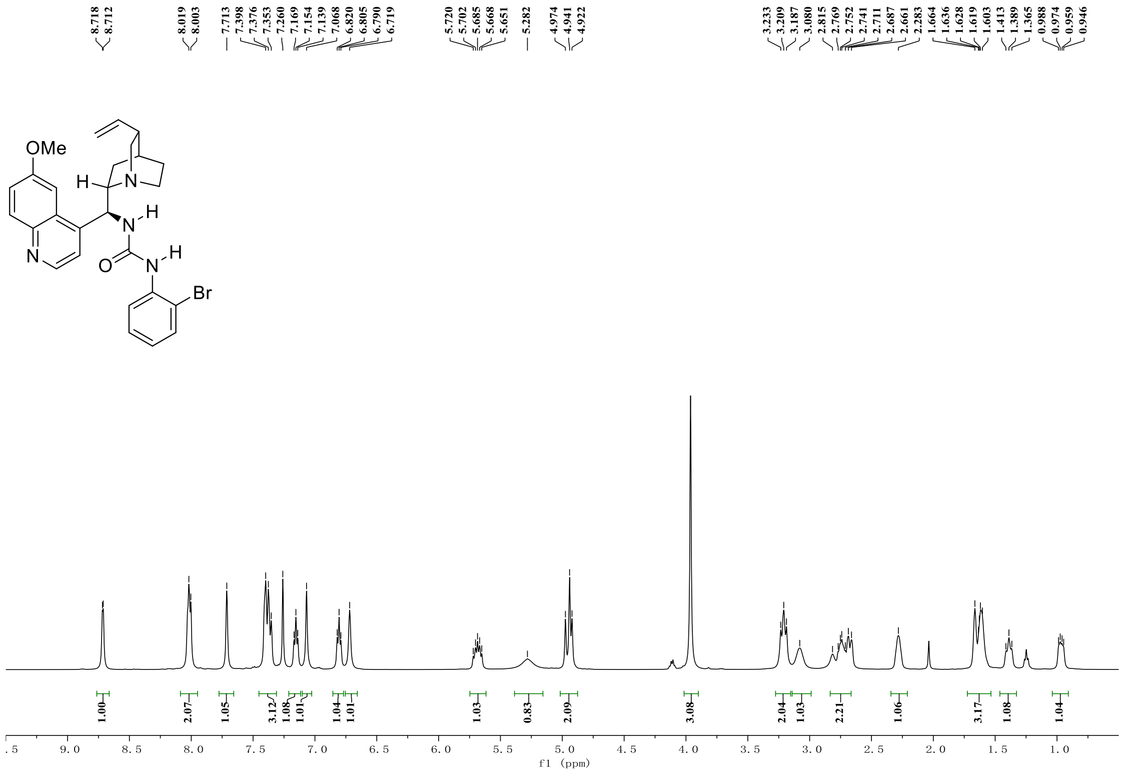

^{1}H NMR was recorded on Bruker 400 MHz; Solvent: $CDCl_3$

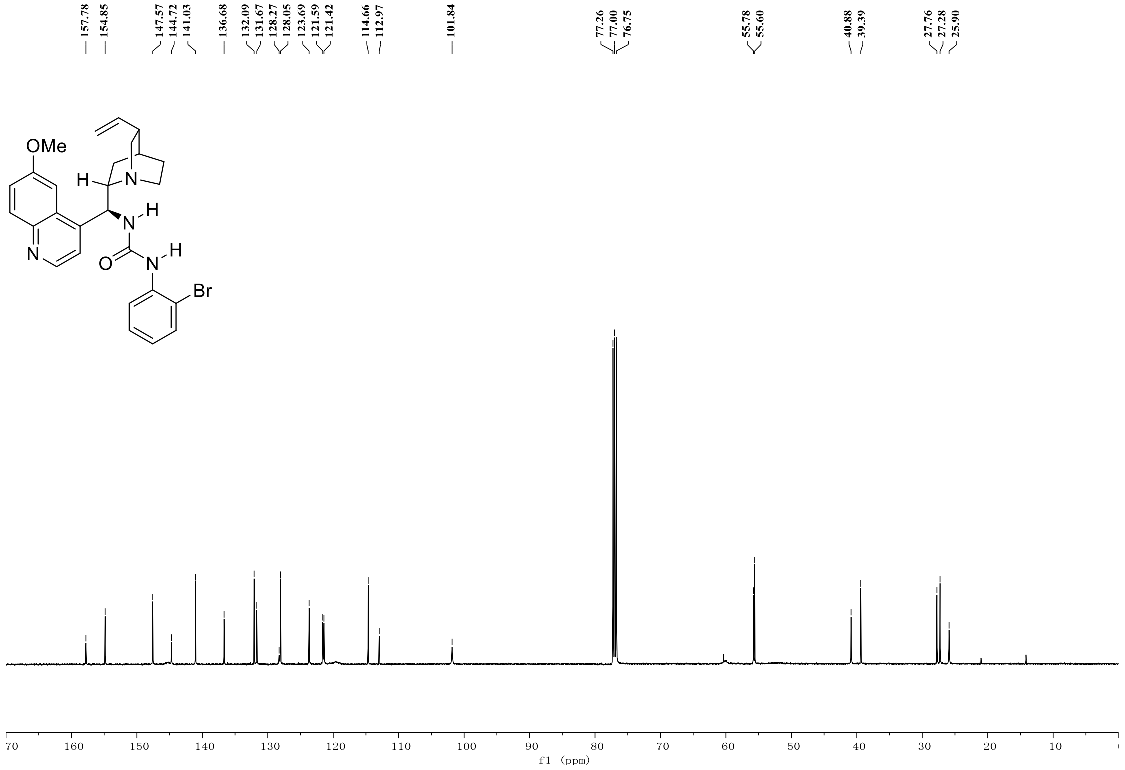

^{13}C NMR was recorded on Bruker 101 MHz; Solvent: $CDCl_3$

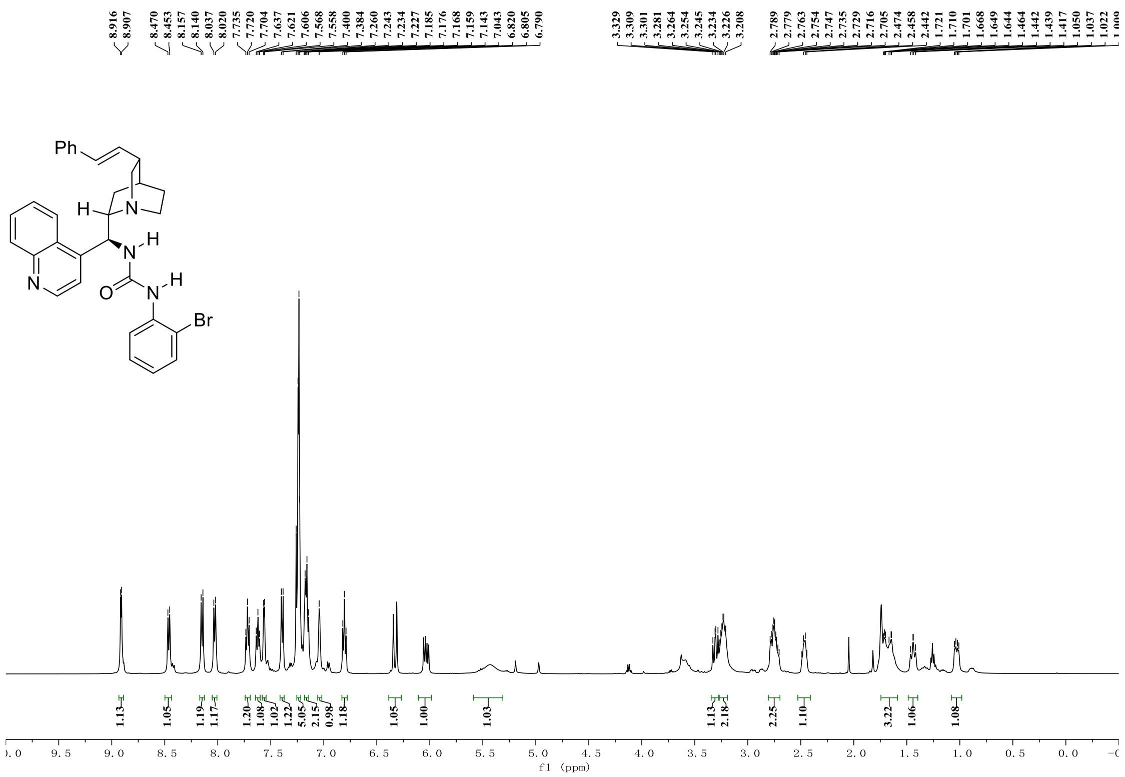

^{1}H NMR was recorded on Bruker 400 MHz; Solvent: $CDCl_3$

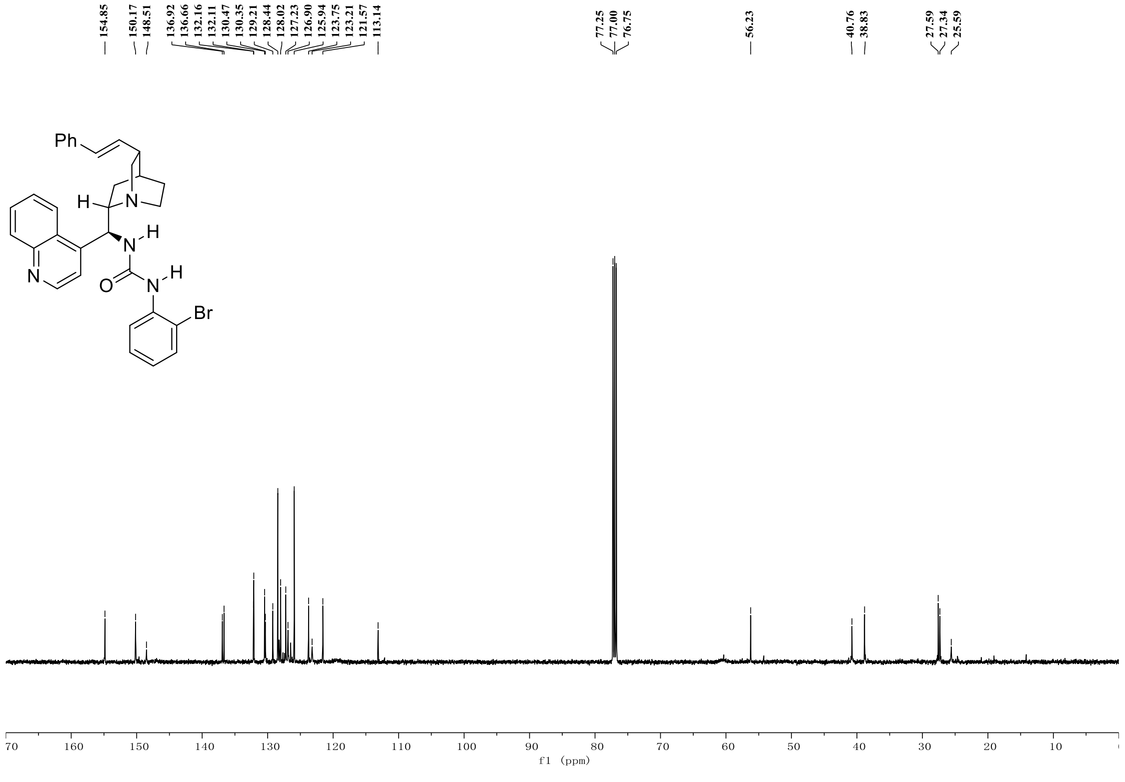

^{13}C NMR was recorded on Bruker 101 MHz; Solvent: $CDCl_3$

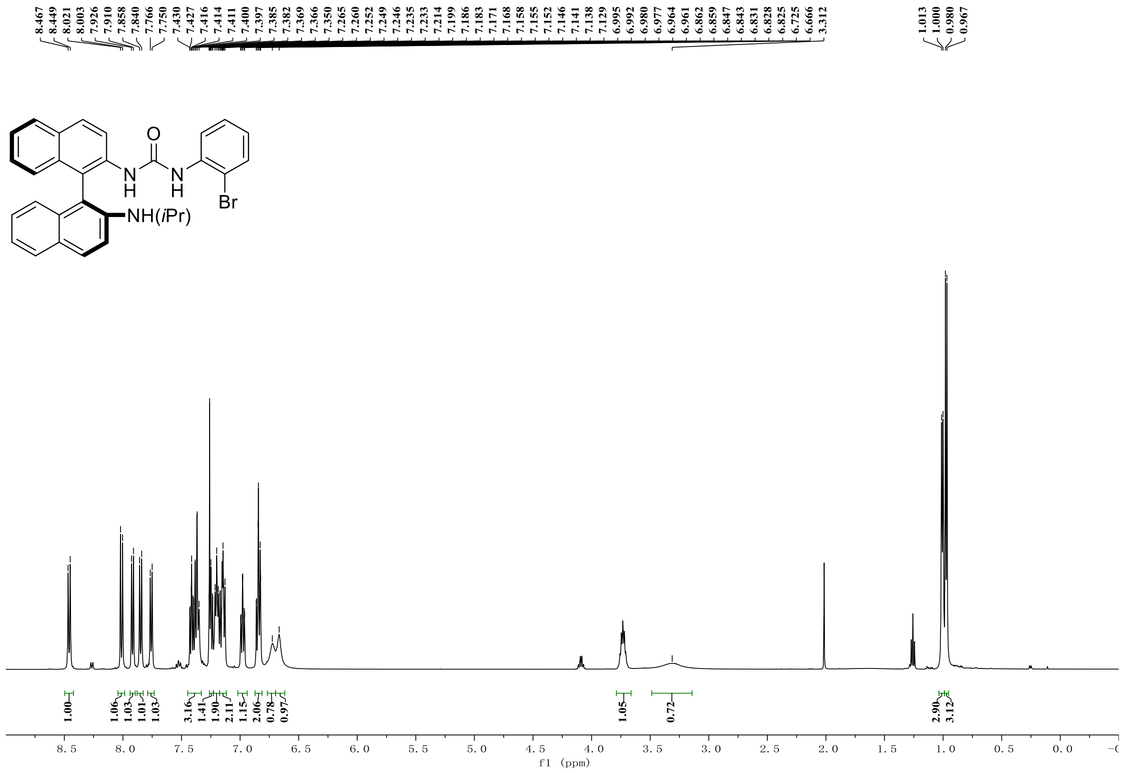

^{1}H NMR was recorded on Bruker 500 MHz; Solvent: $CDCl_3$

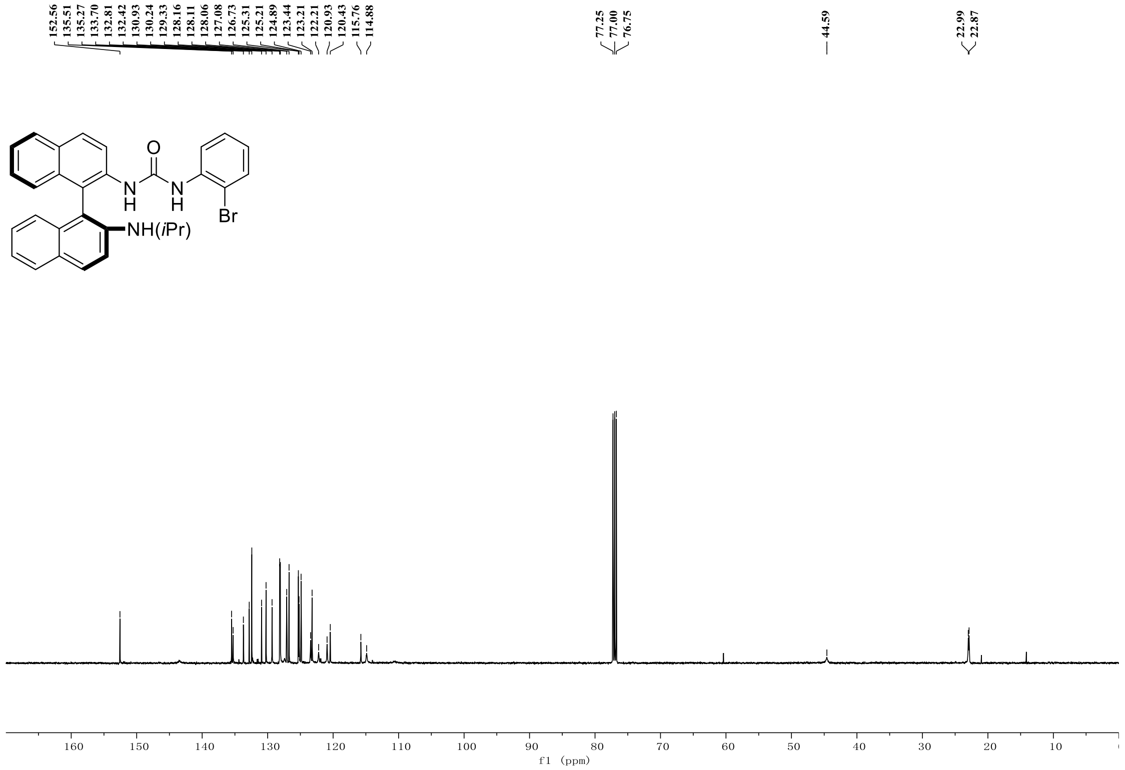

^{13}C NMR was recorded on Bruker 126 MHz; Solvent: $CDCl_3$

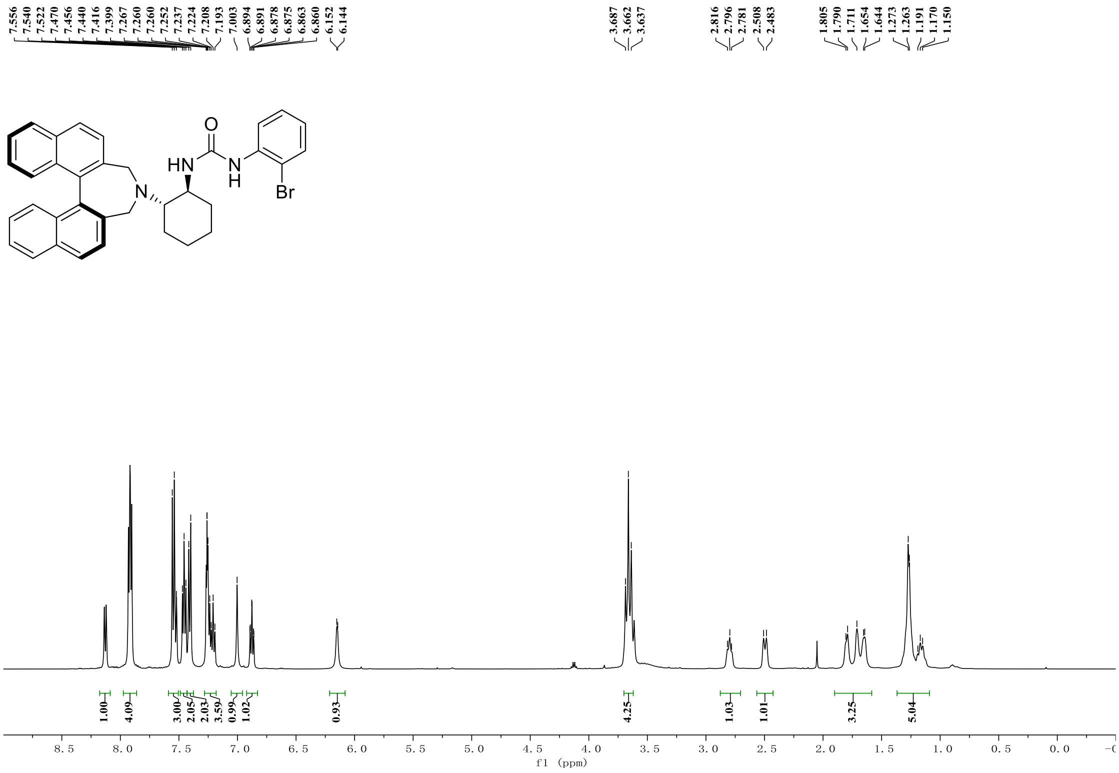

^{1}H NMR was recorded on Bruker 500 MHz; Solvent: $CDCl_3$

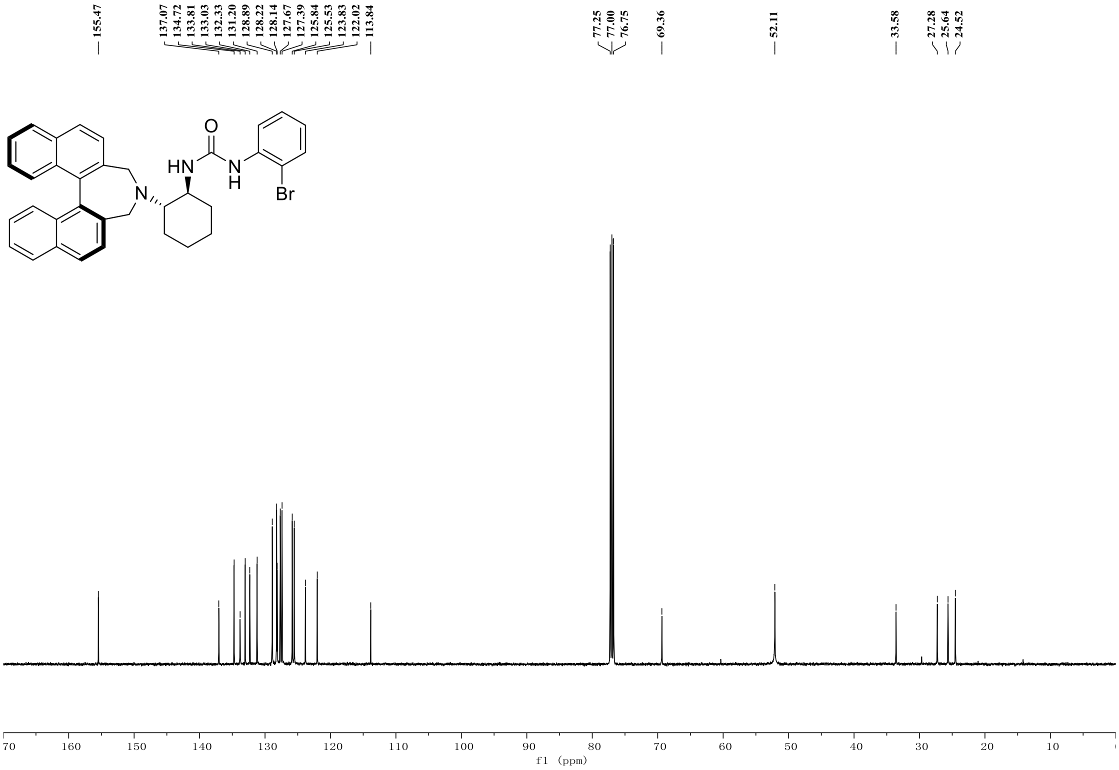

^{13}C NMR was recorded on Bruker 126 MHz; Solvent: $CDCl_3$

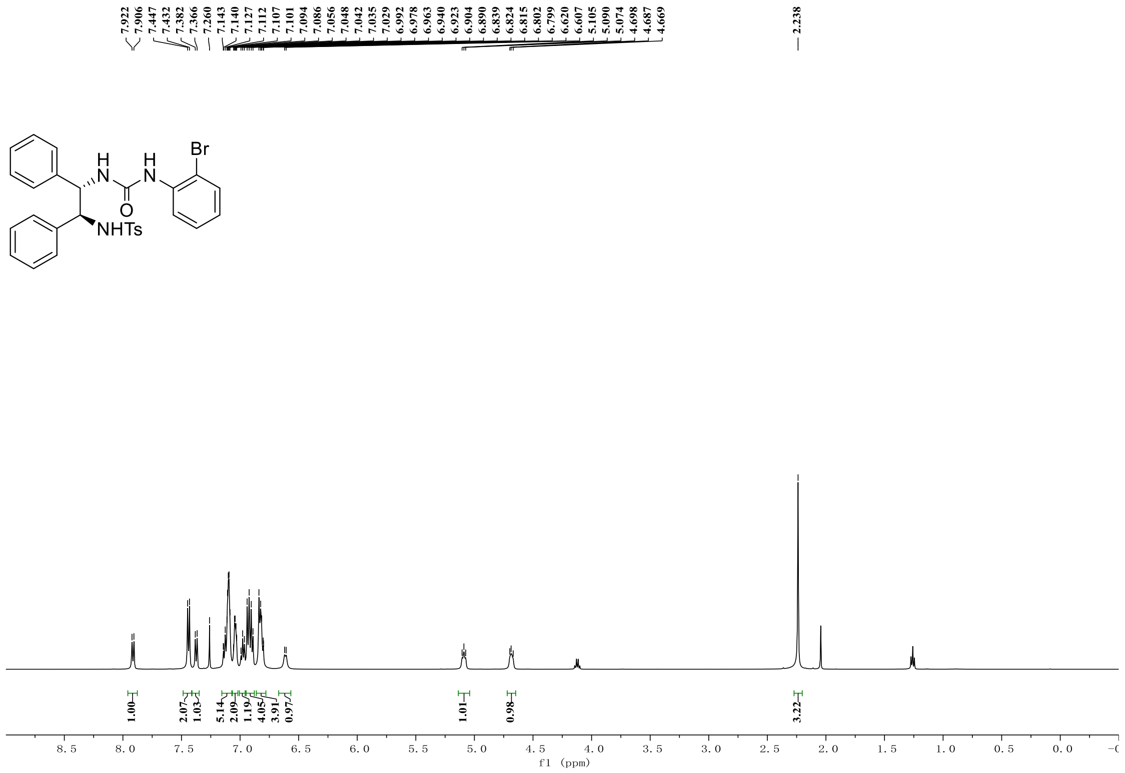

^{1}H NMR was recorded on Bruker 500 MHz; Solvent: $CDCl_3$

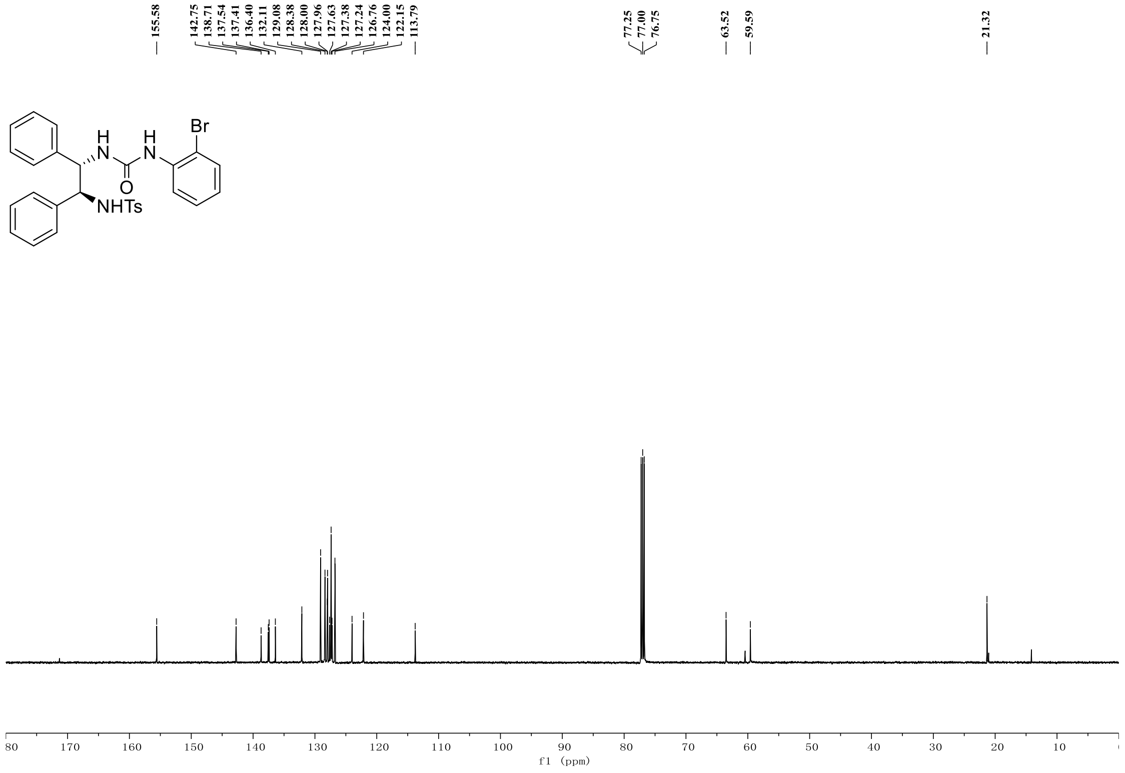

^{13}C NMR was recorded on Bruker 126 MHz; Solvent: $CDCl_3$

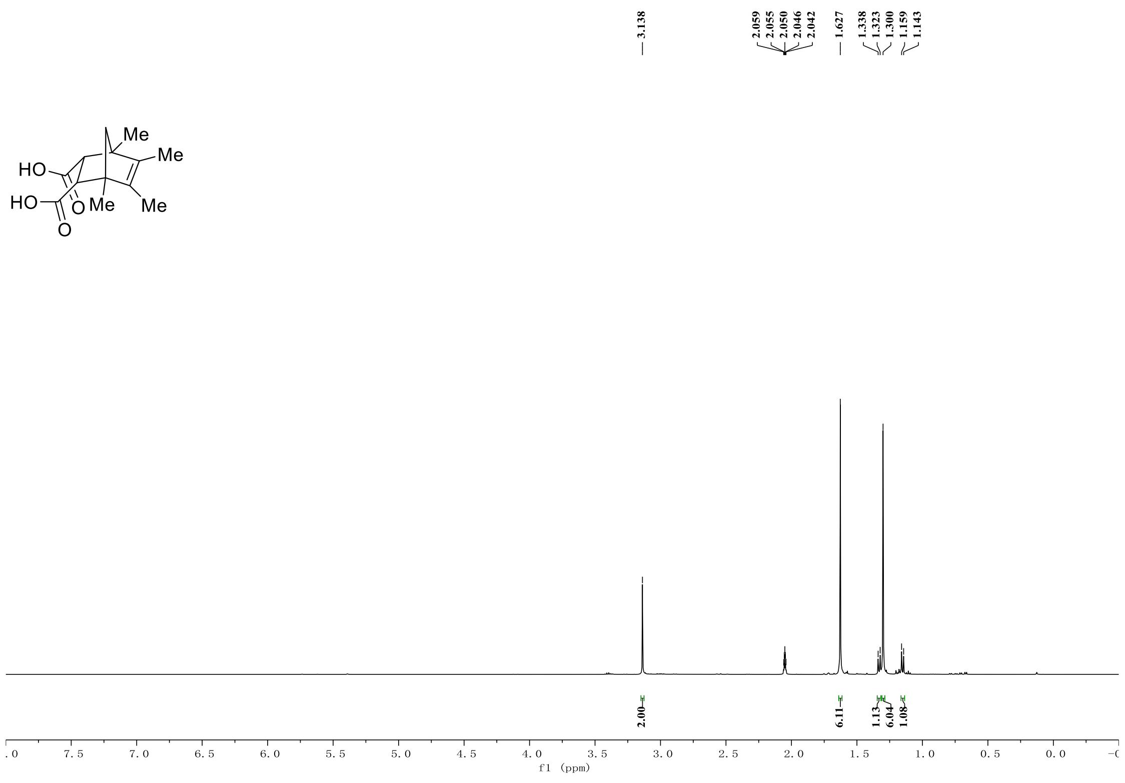

^{1}H NMR was recorded on Bruker 500 MHz; Solvent: Acetone-d_6

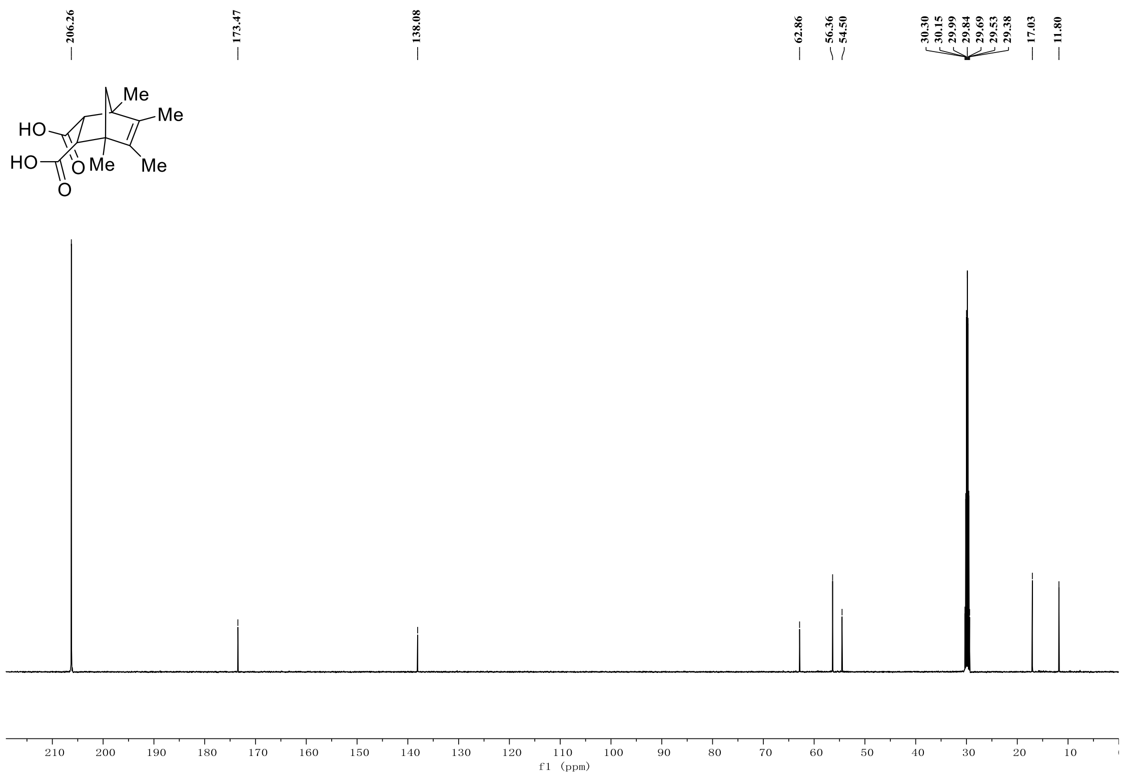

^{13}C NMR was recorded on Bruker 126 MHz; Solvent: Acetone-d_6

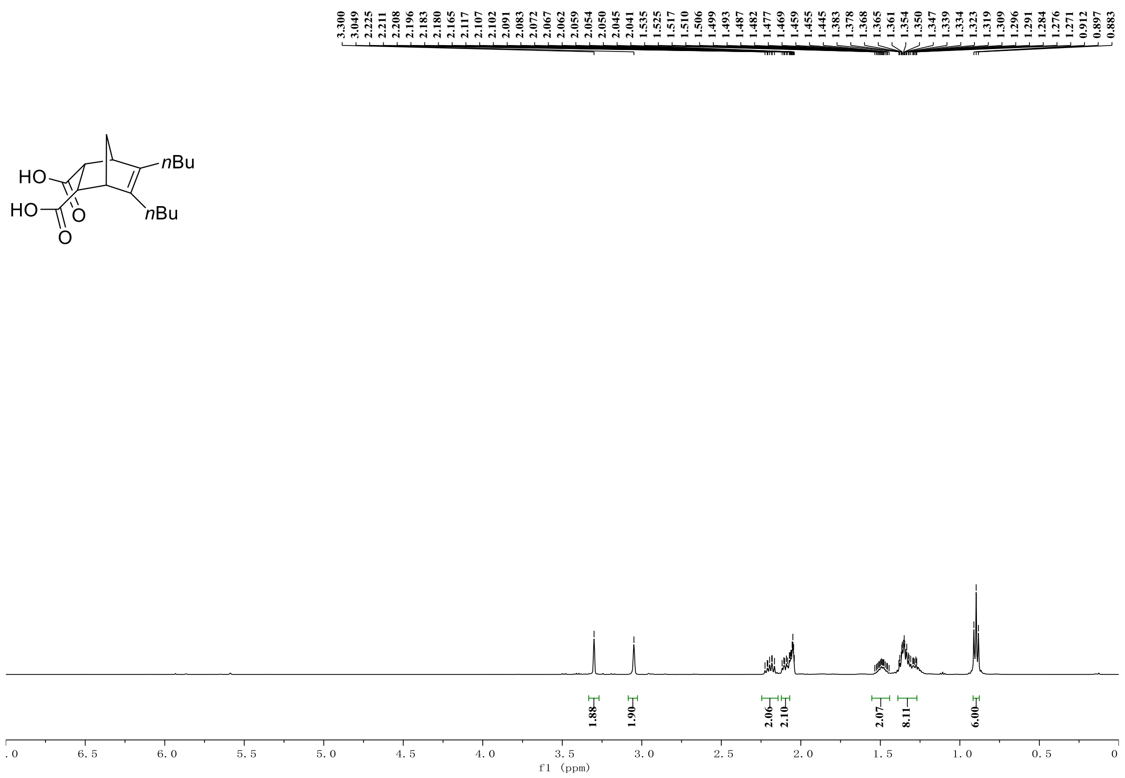

^{1}H NMR was recorded on Bruker 500 MHz; Solvent: Acetone-d_6

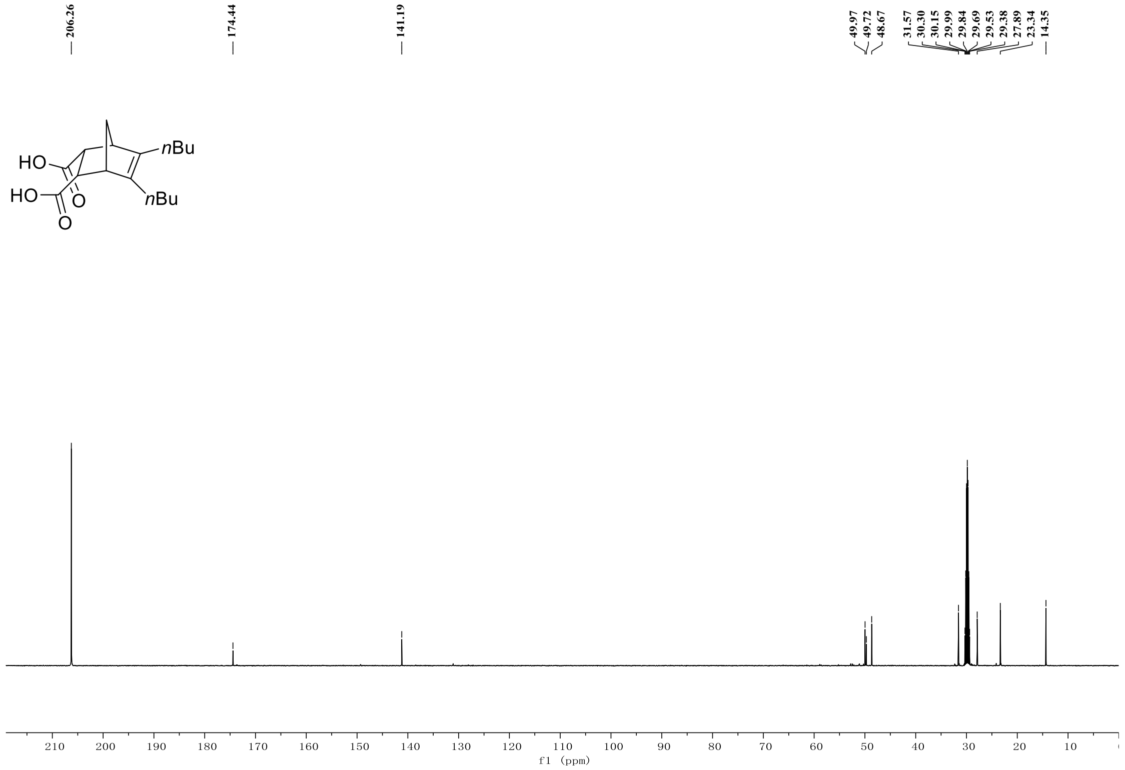

^{13}C NMR was recorded on Bruker 126 MHz; Solvent: Acetone-d_6

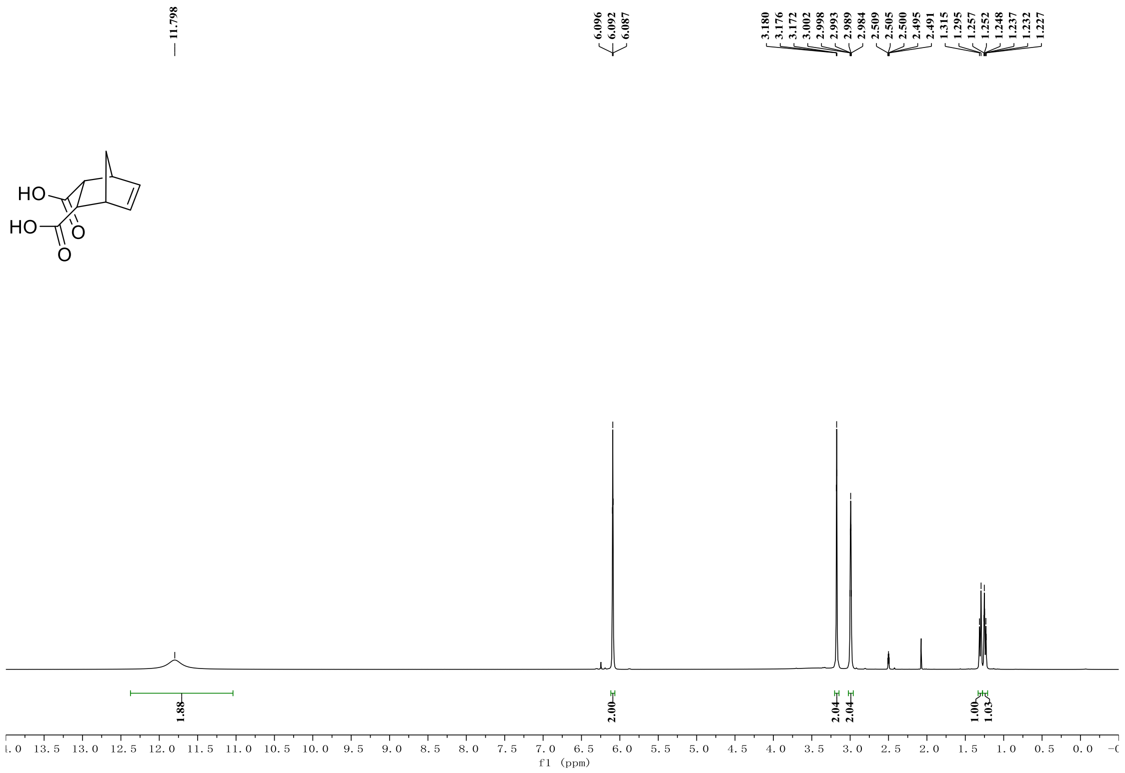

^{1}H NMR was recorded on Bruker 400 MHz; Solvent: DMSO-d_6

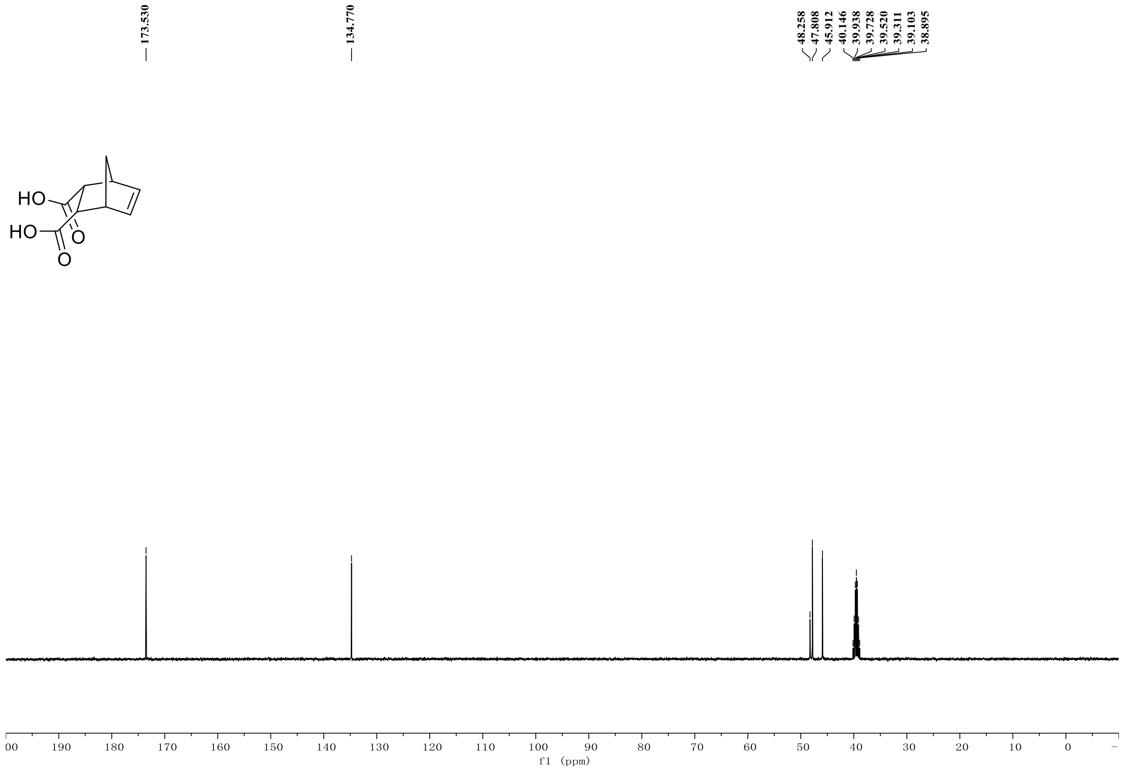

^{13}C NMR was recorded on Bruker 101 MHz; Solvent: DMSO-d_6

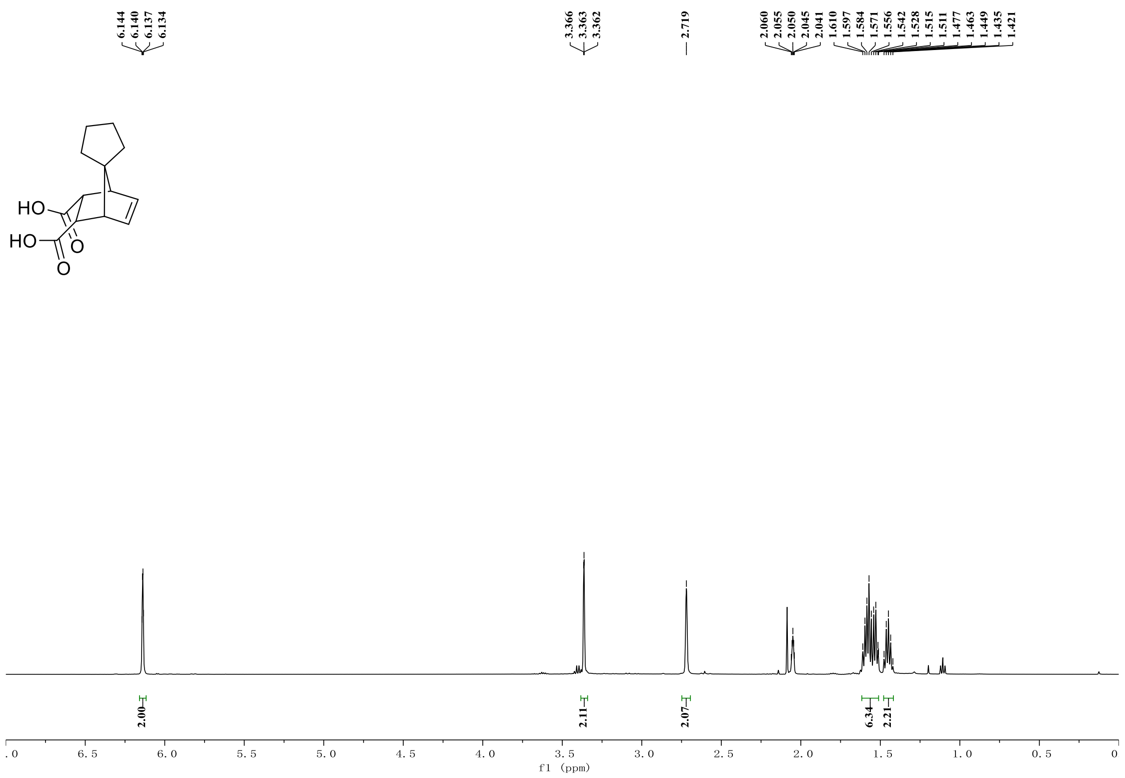

^{1}H NMR was recorded on Bruker 500 MHz; Solvent: Acetone-d_6

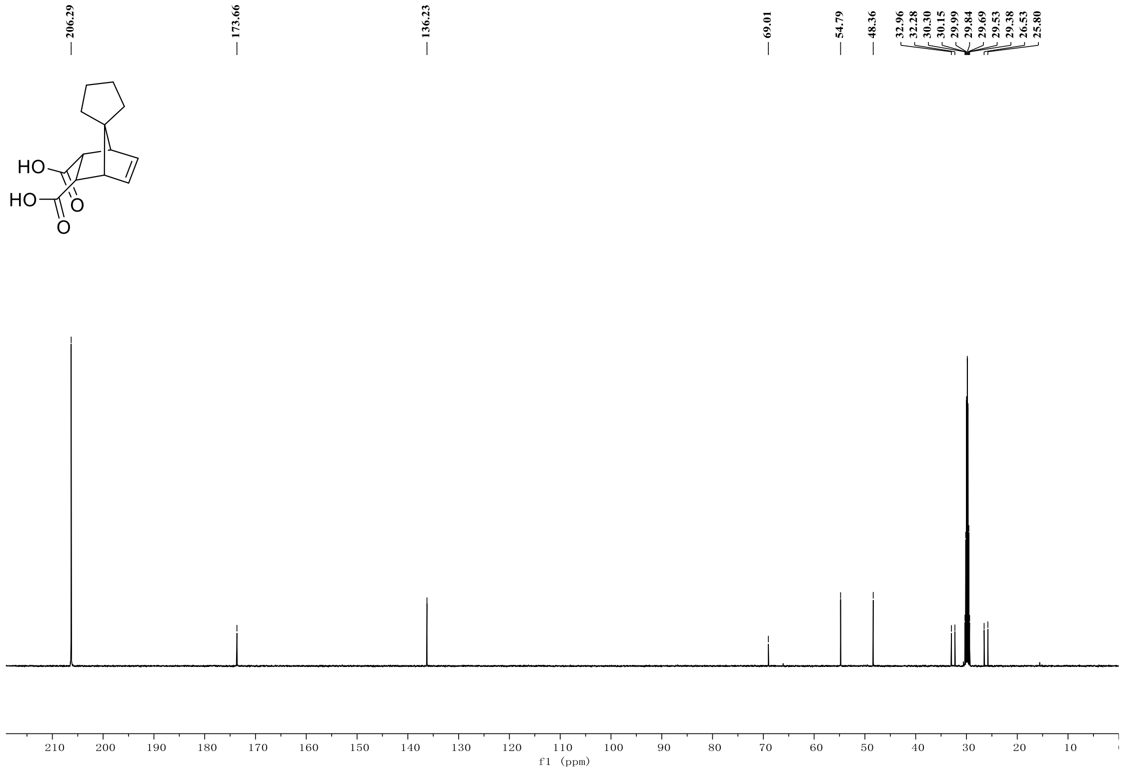

^{13}C NMR was recorded on Bruker 126 MHz; Solvent: Acetone-d_6

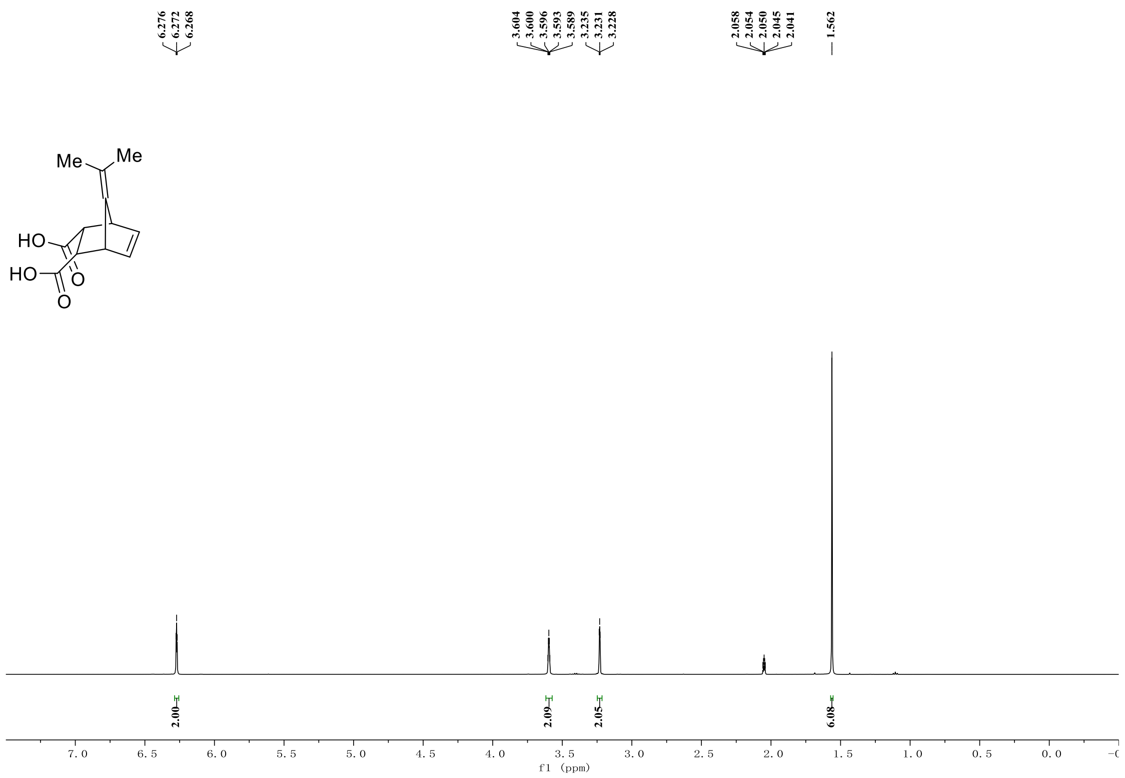

^{1}H NMR was recorded on Bruker 500 MHz; Solvent: Acetone-d_6

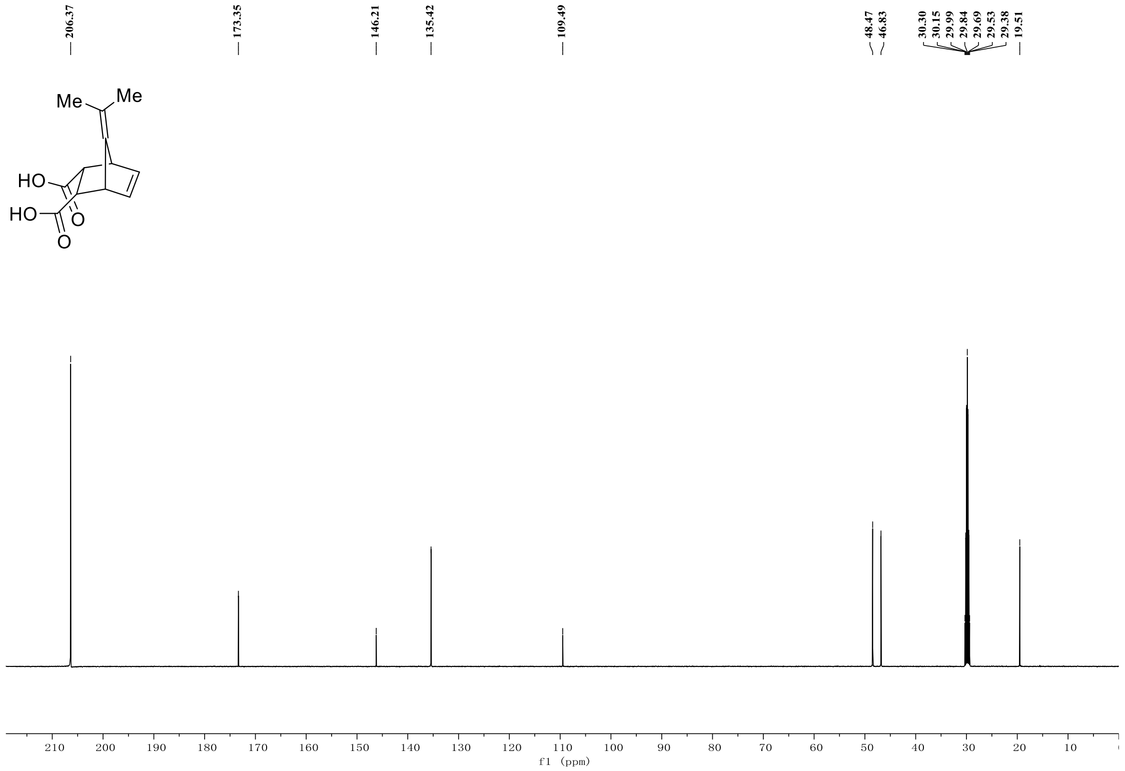

^{13}C NMR was recorded on Bruker 126 MHz; Solvent: Acetone-d_6

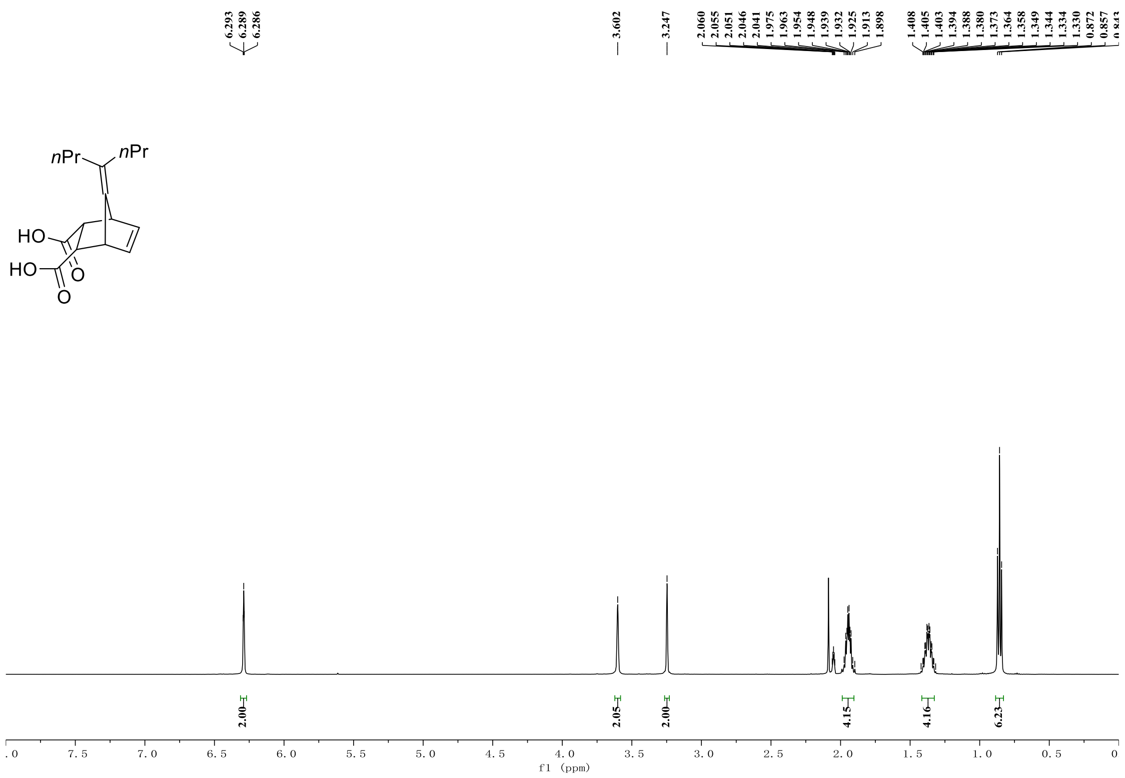

^{1}H NMR was recorded on Bruker 500 MHz; Solvent: Acetone-d_6

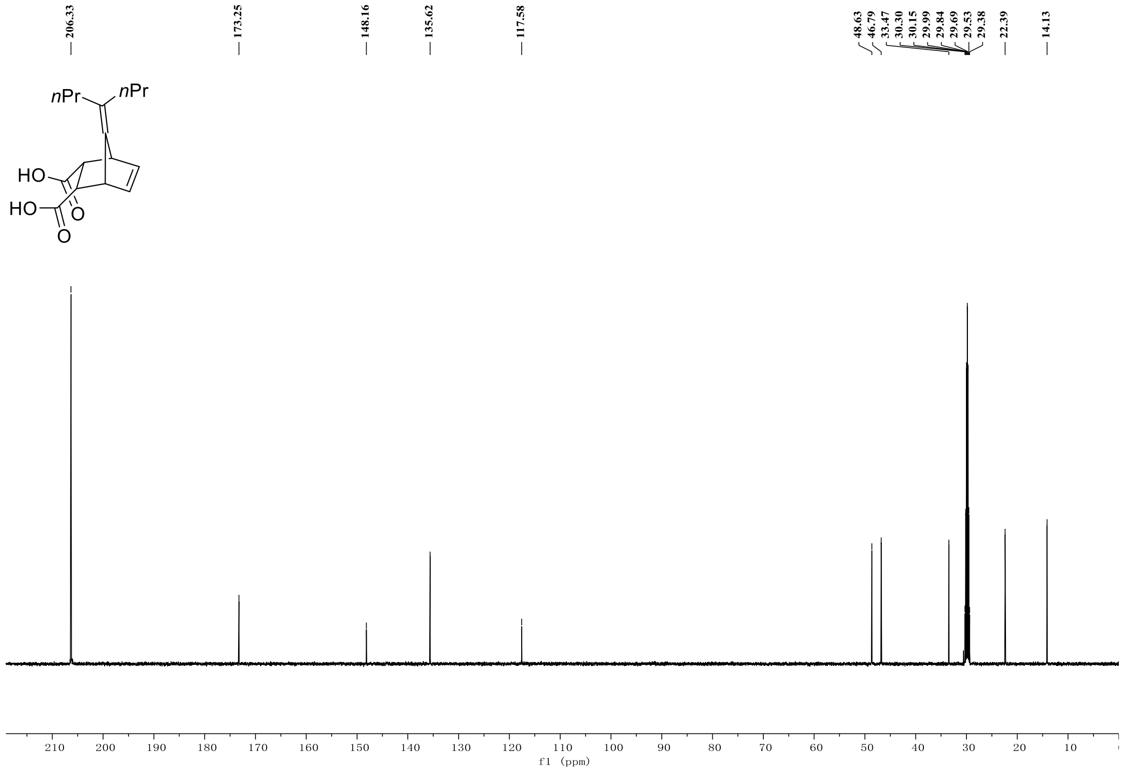

^{13}C NMR was recorded on Bruker 126 MHz; Solvent: Acetone-d_6

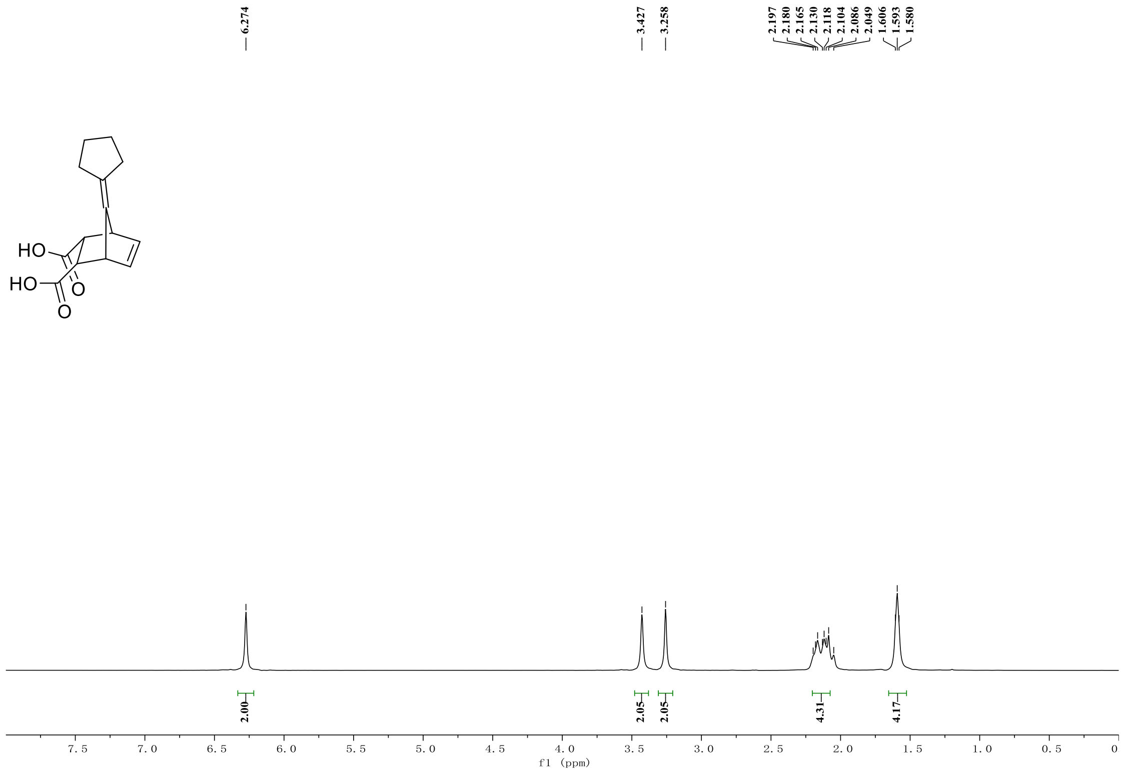

^{1}H NMR was recorded on Bruker 500 MHz; Solvent: Acetone-d_6

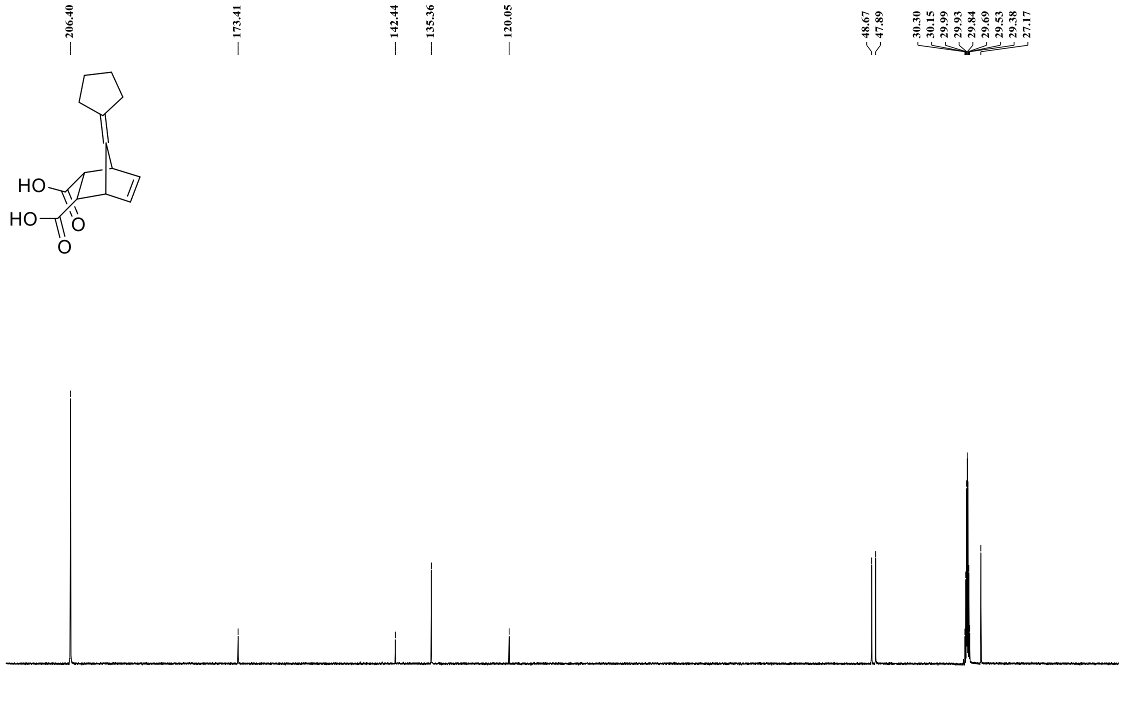

^{13}C NMR was recorded on Bruker 126 MHz; Solvent: Acetone-d_6

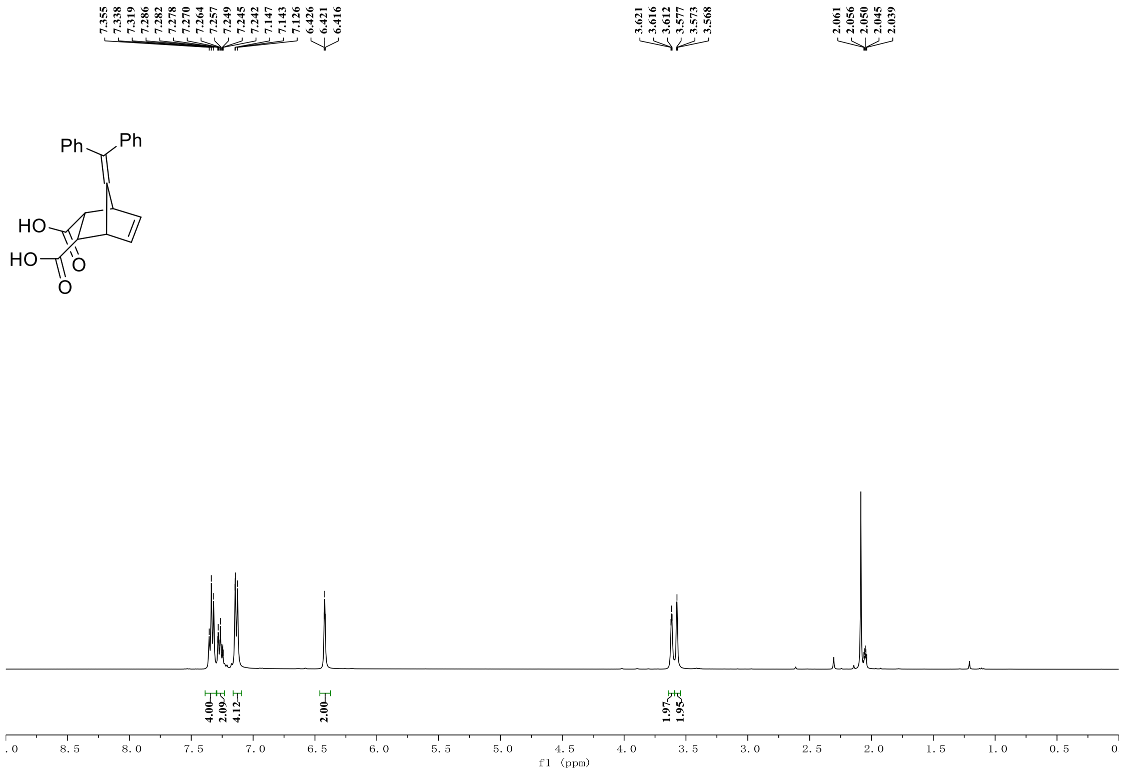

^{1}H NMR was recorded on Bruker 400 MHz; Solvent: Acetone-d_6

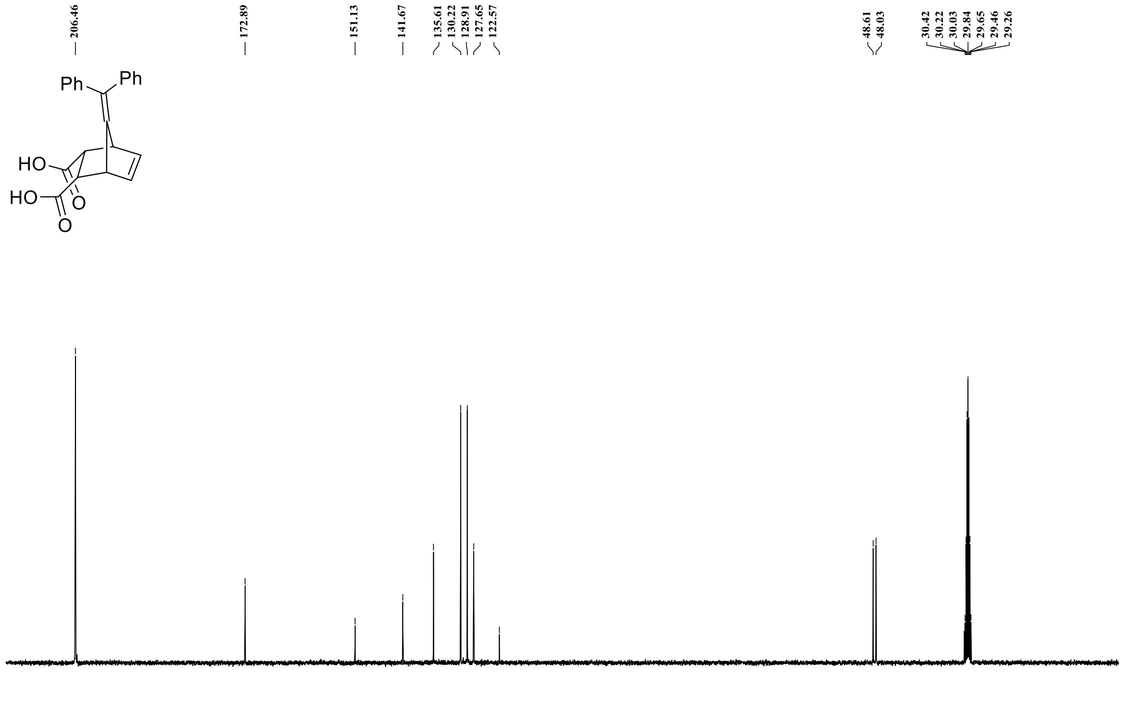

^{13}C NMR was recorded on Bruker 101 MHz; Solvent: Acetone-d_6

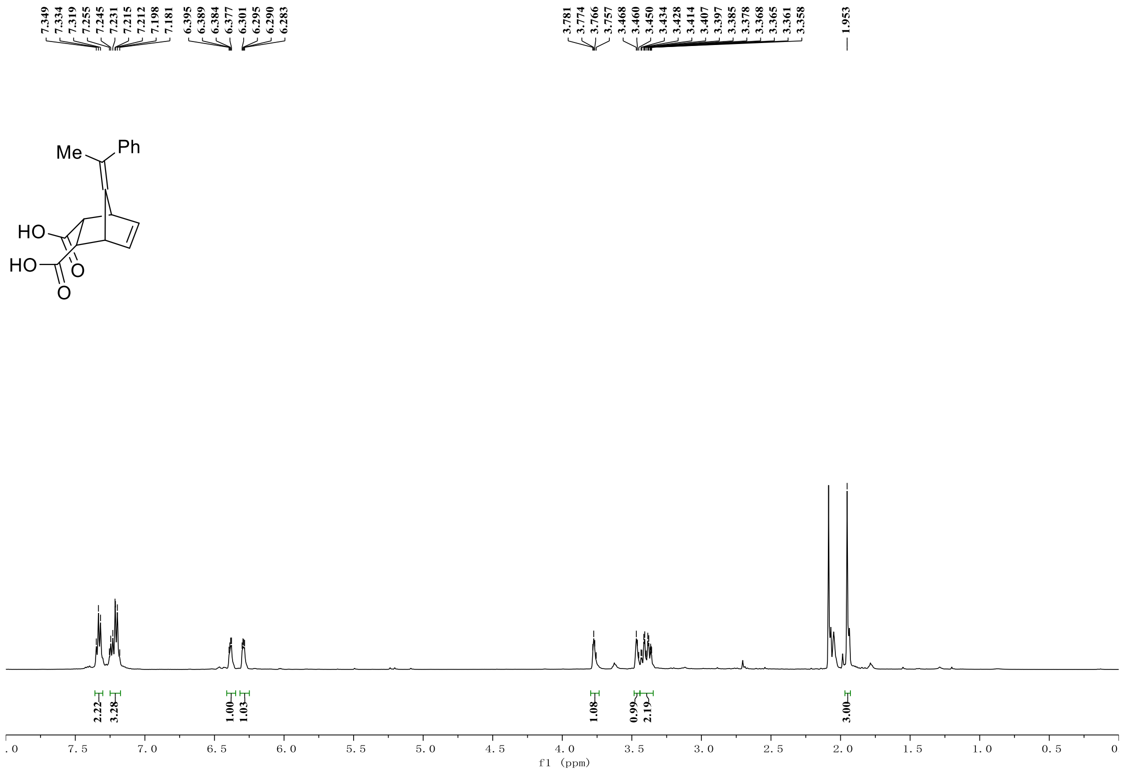

^{1}H NMR was recorded on Bruker 500 MHz; Solvent: Acetone-d_6

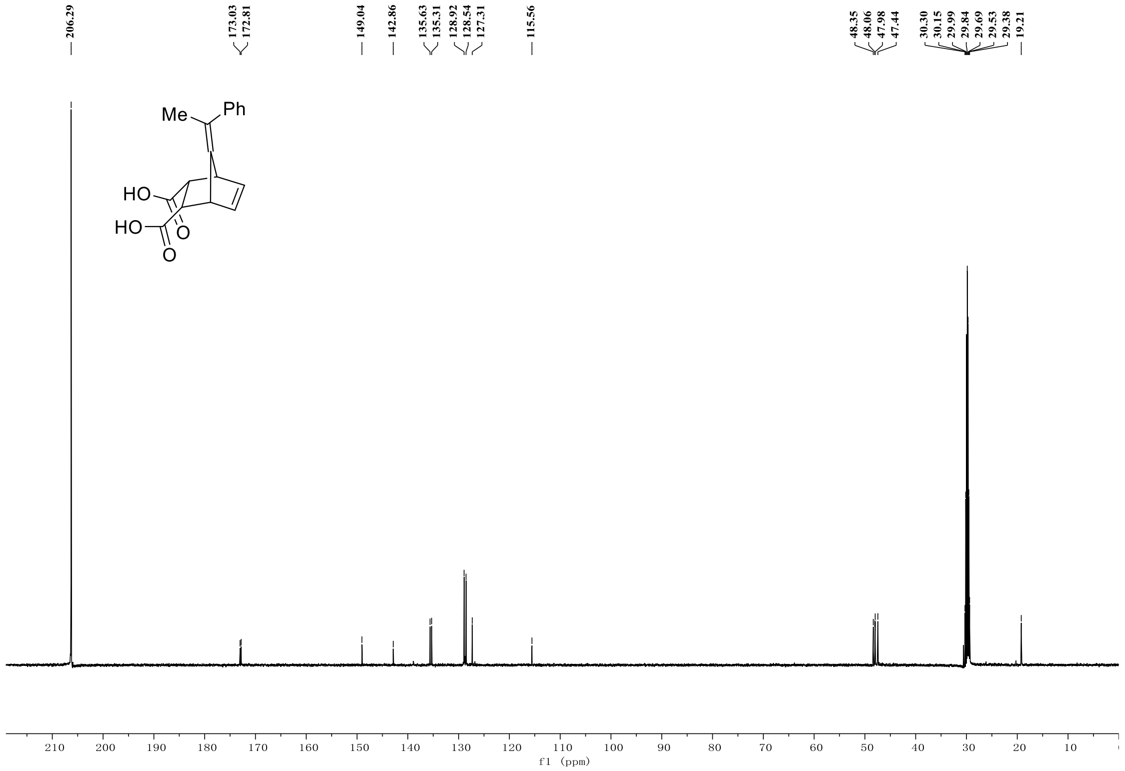

^{13}C NMR was recorded on Bruker 126 MHz; Solvent: Acetone-d_6

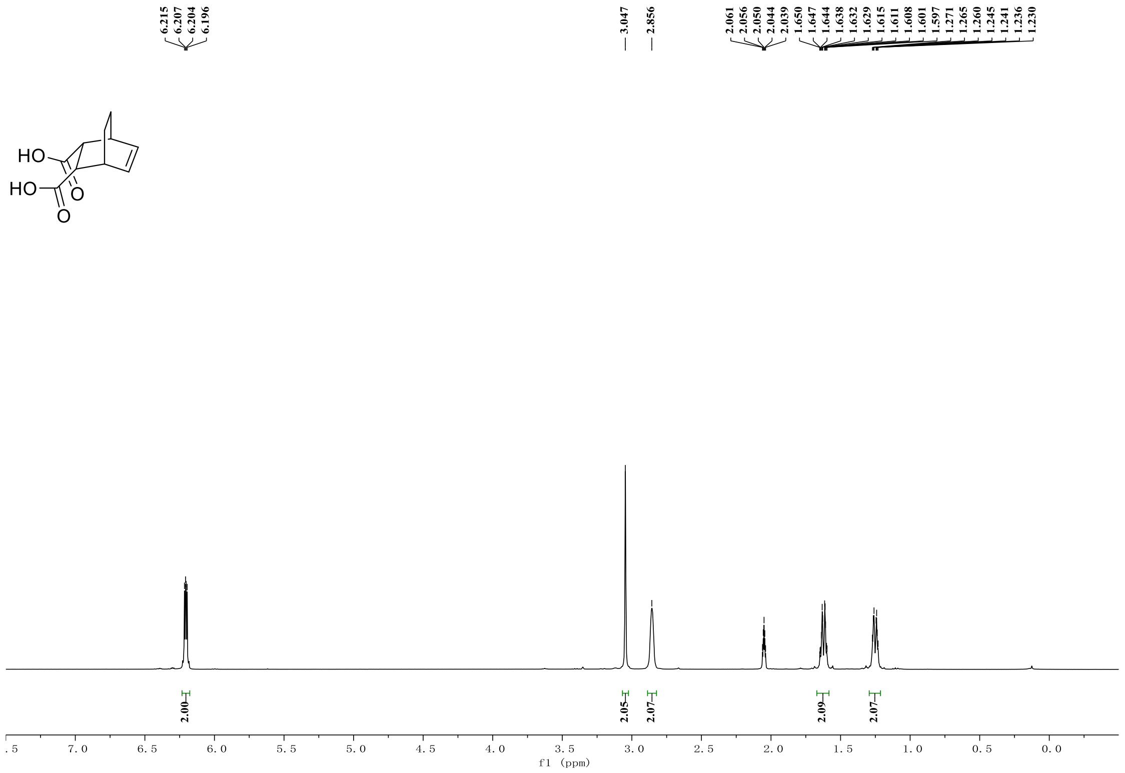

^{1}H NMR was recorded on Bruker 400 MHz; Solvent: Acetone-d_6

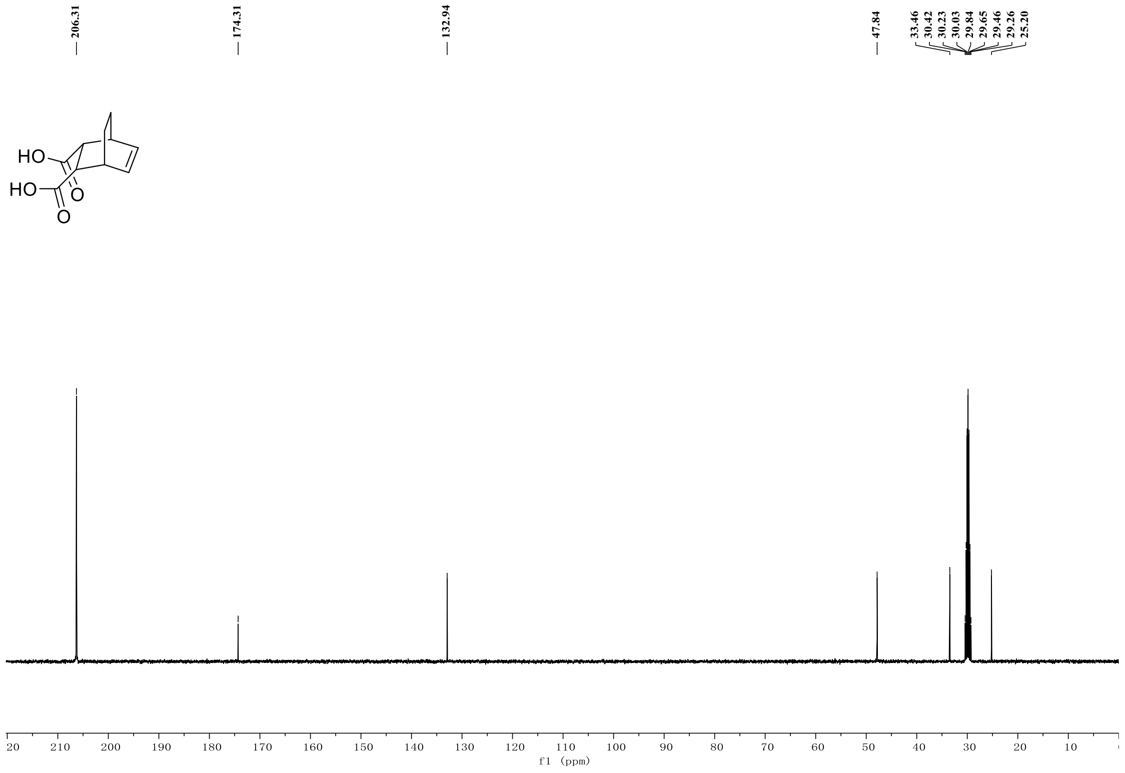

^{13}C NMR was recorded on Bruker 101 MHz; Solvent: Acetone-d_6

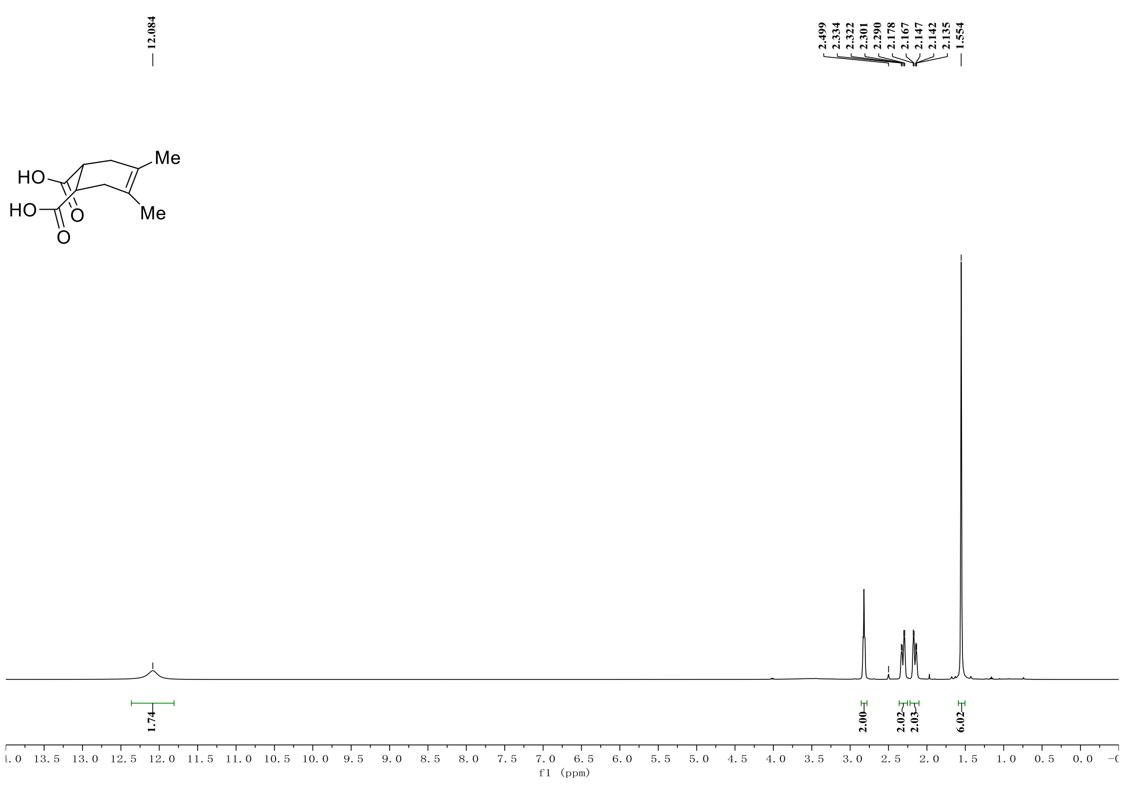

^{1}H NMR was recorded on Bruker 500 MHz; Solvent: DMSO-d_6

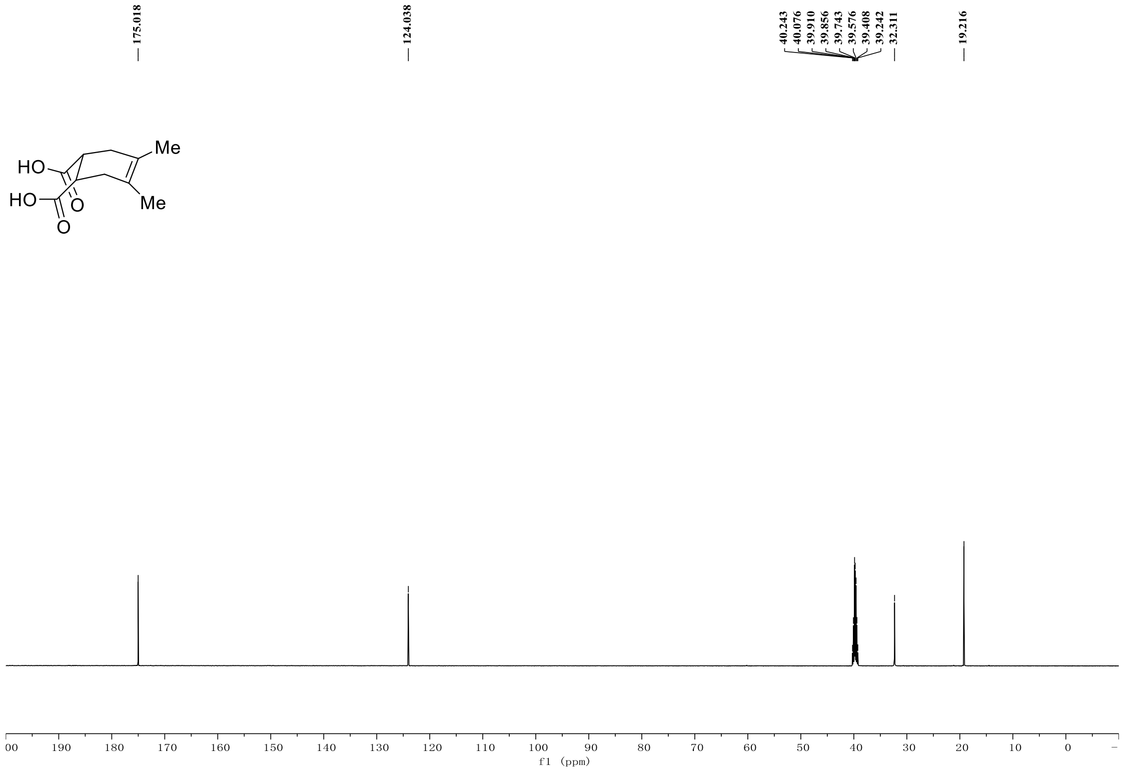

^{13}C NMR was recorded on Bruker 126 MHz; Solvent: DMSO-d_6

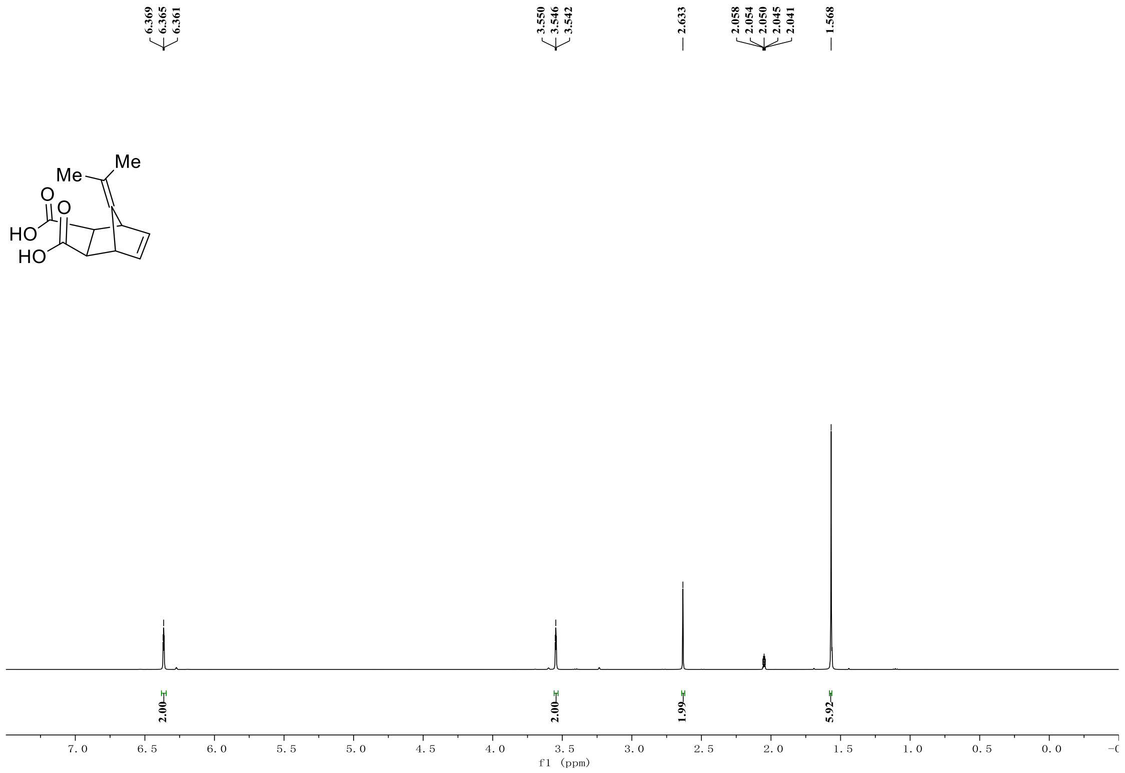

^{1}H NMR was recorded on Bruker 500 MHz; Solvent: Acetone-d_6

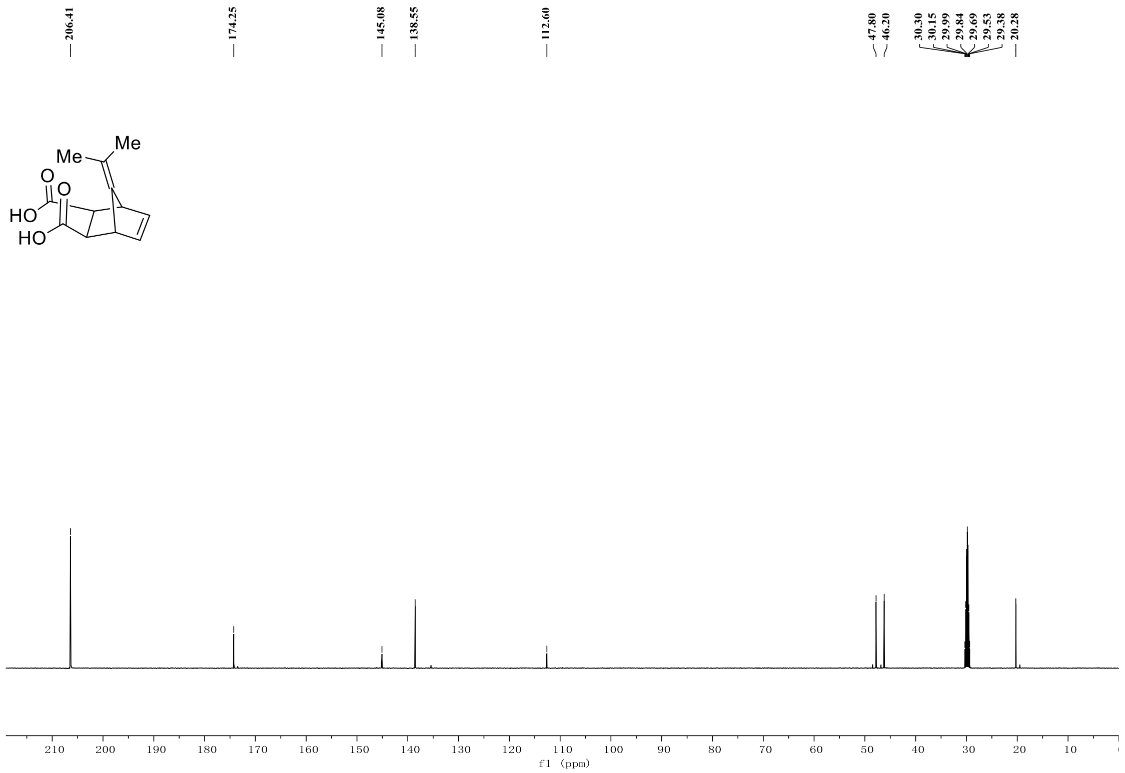

^{13}C NMR was recorded on Bruker 126 MHz; Solvent: Acetone-d_6

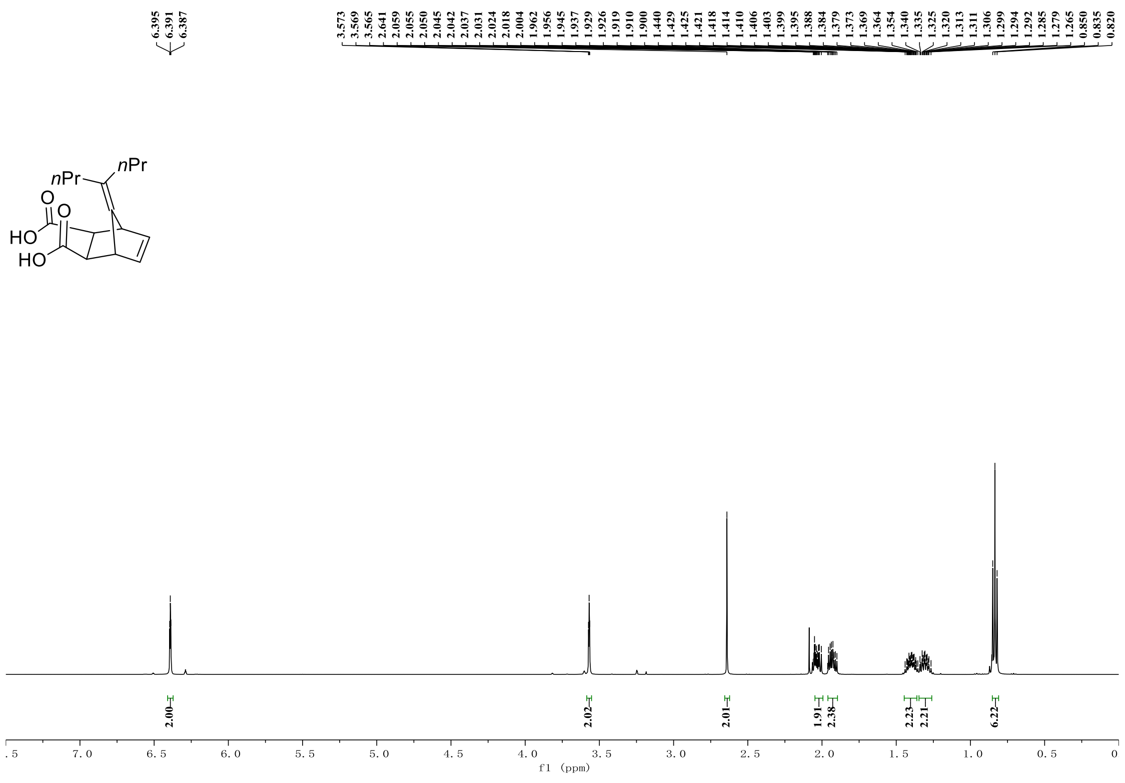

^{1}H NMR was recorded on Bruker 500 MHz; Solvent: Acetone-d_6

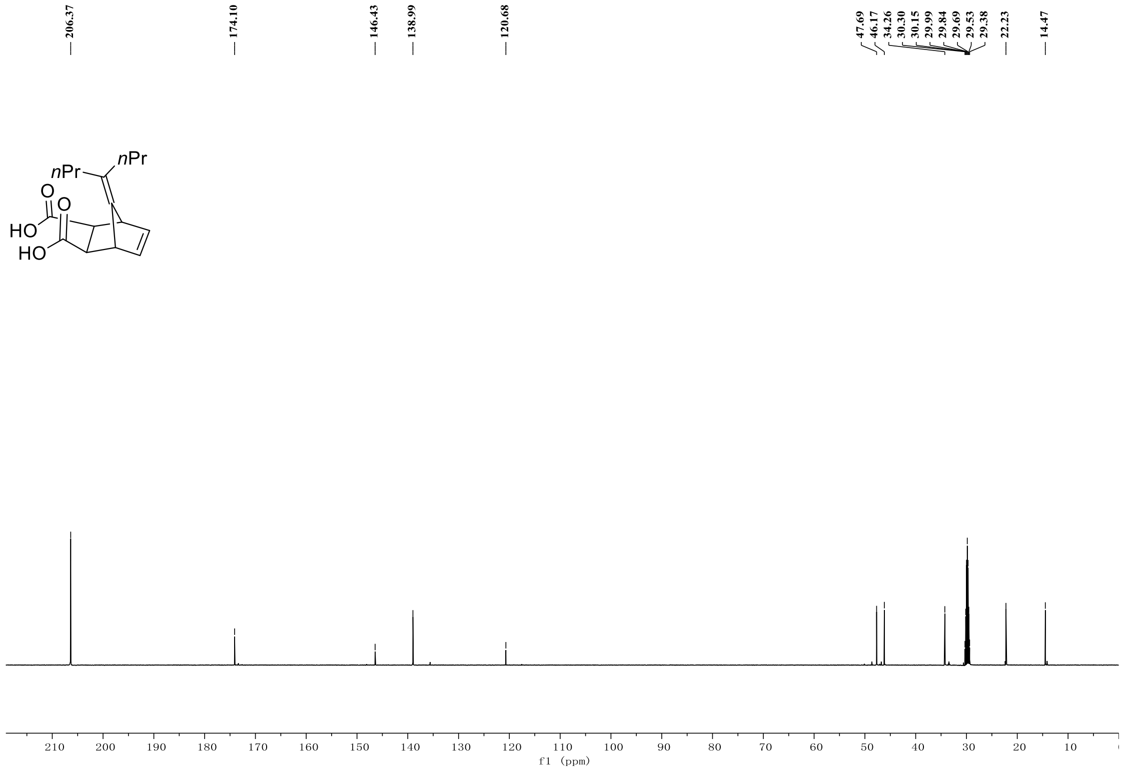

^{13}C NMR was recorded on Bruker 126 MHz; Solvent: Acetone-d_6

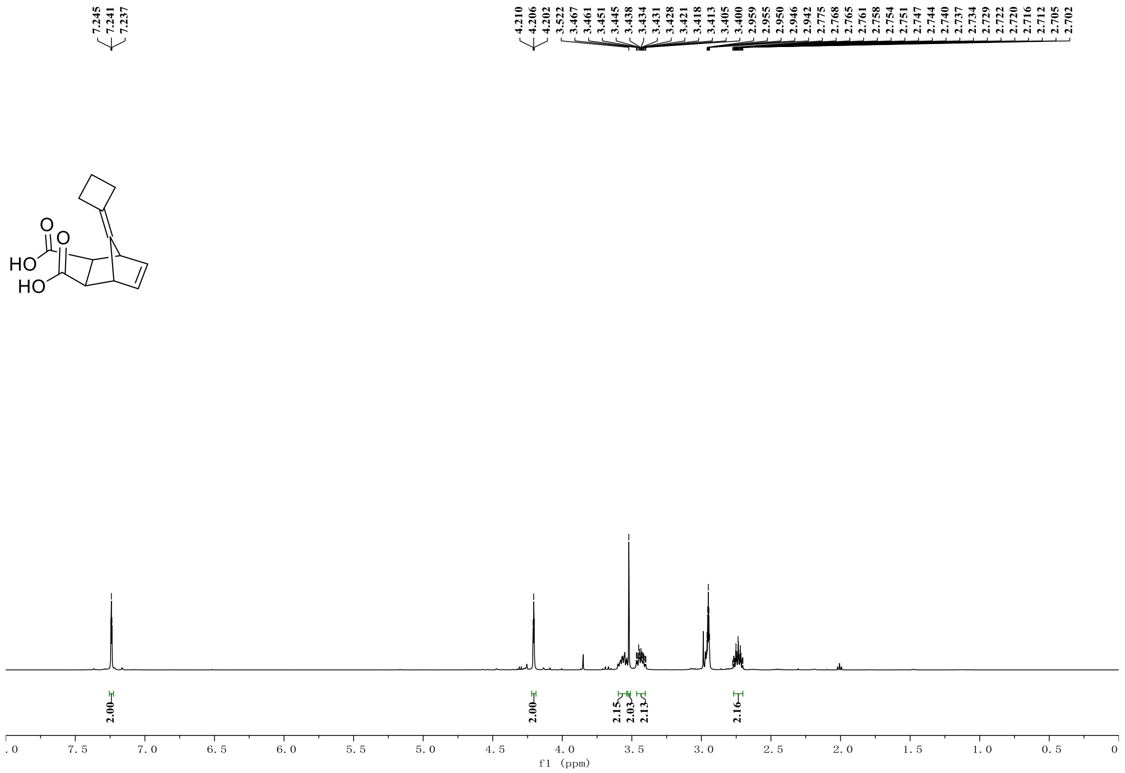

^{1}H NMR was recorded on Bruker 500 MHz; Solvent: Acetone-d_6

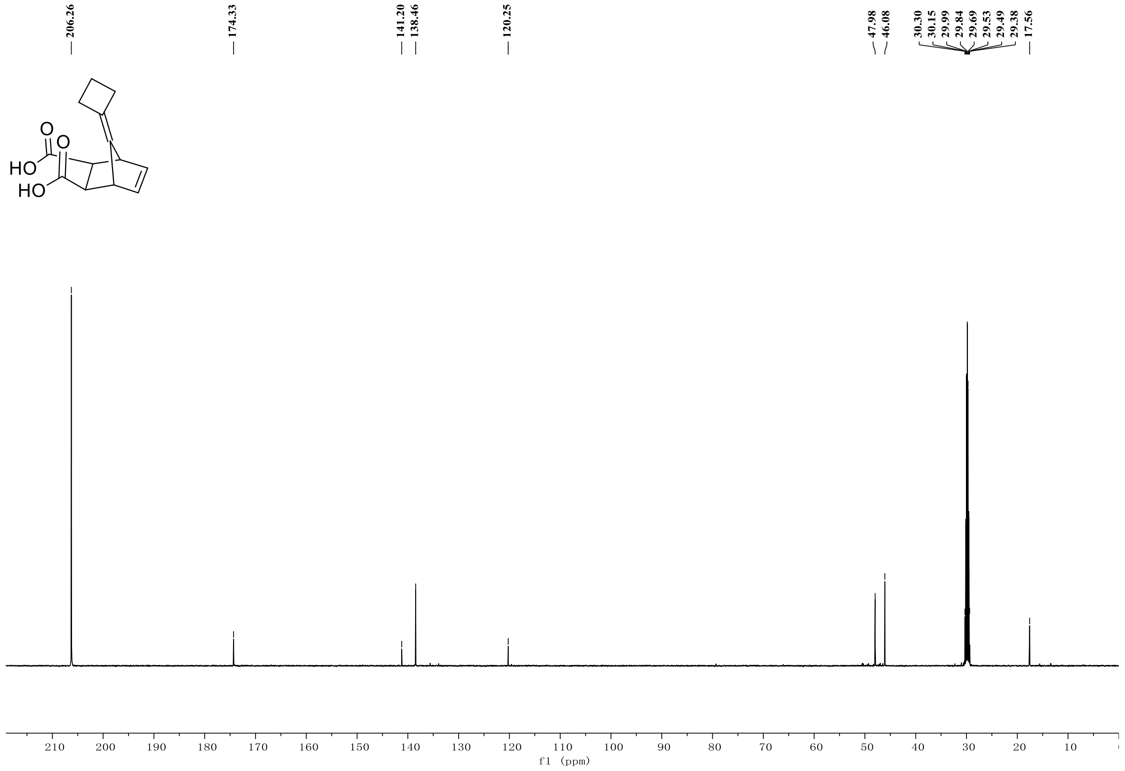

^{13}C NMR was recorded on Bruker 126 MHz; Solvent: Acetone-d_6

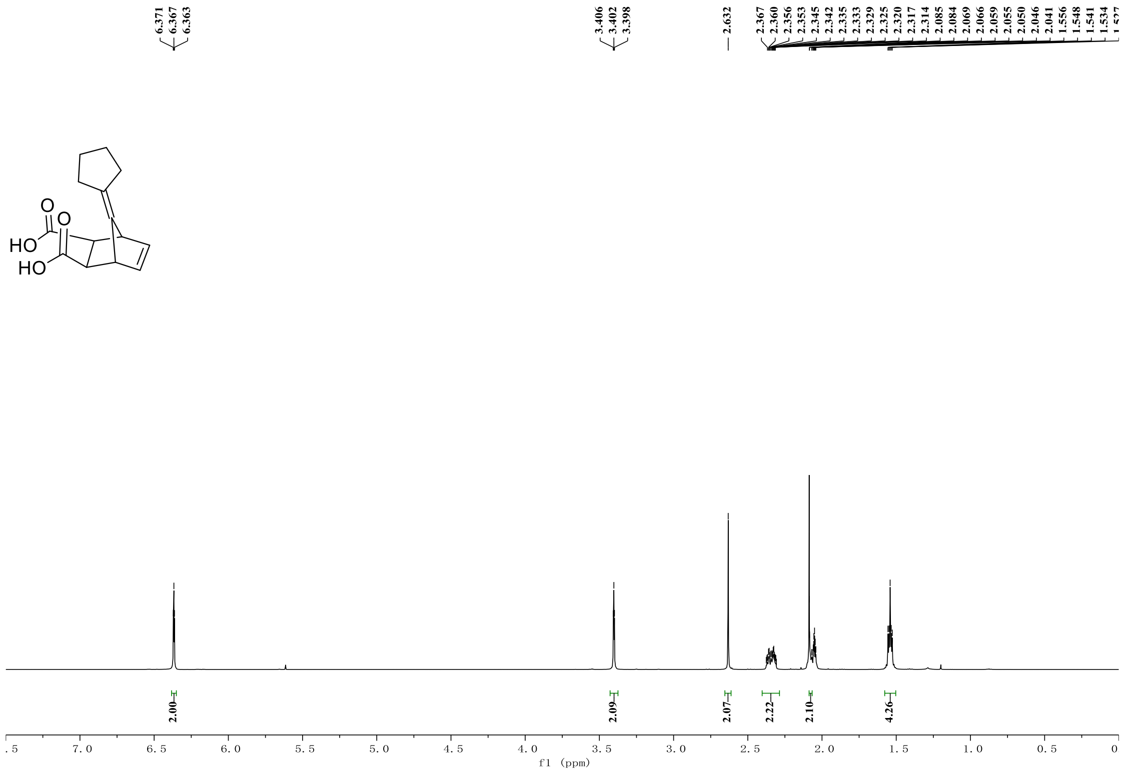

^{1}H NMR was recorded on Bruker 500 MHz; Solvent: Acetone-d_6

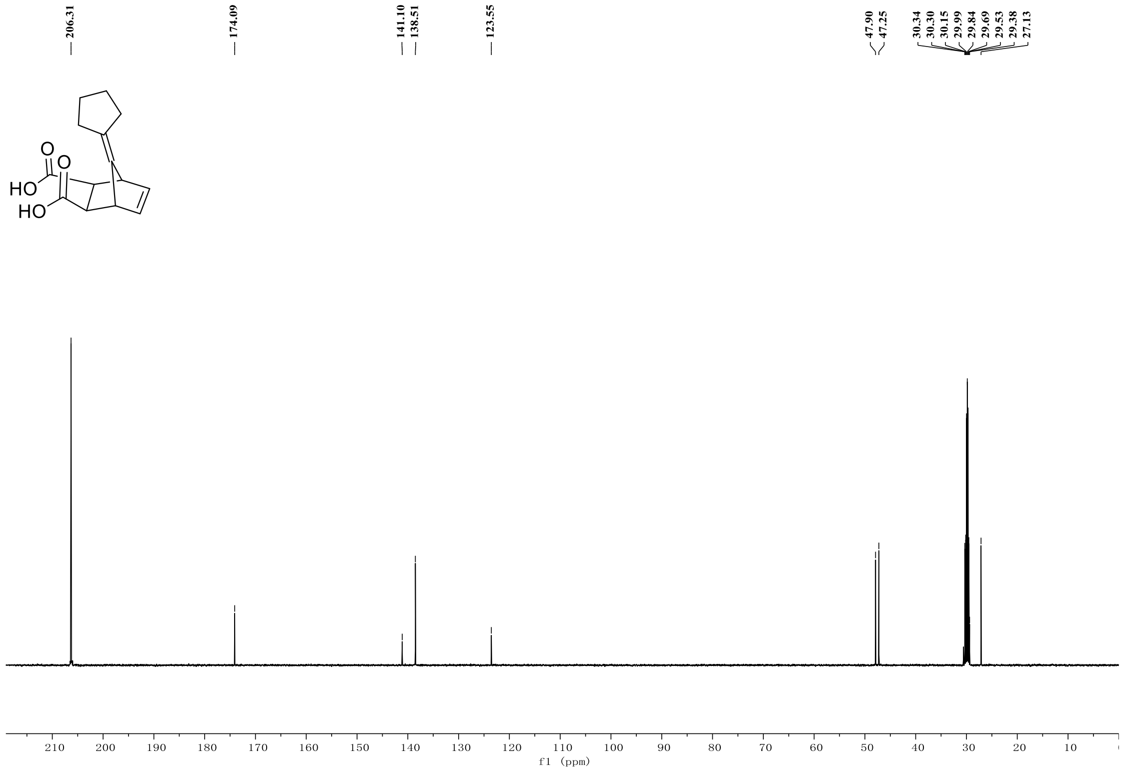

^{13}C NMR was recorded on Bruker 126 MHz; Solvent: Acetone-d_6

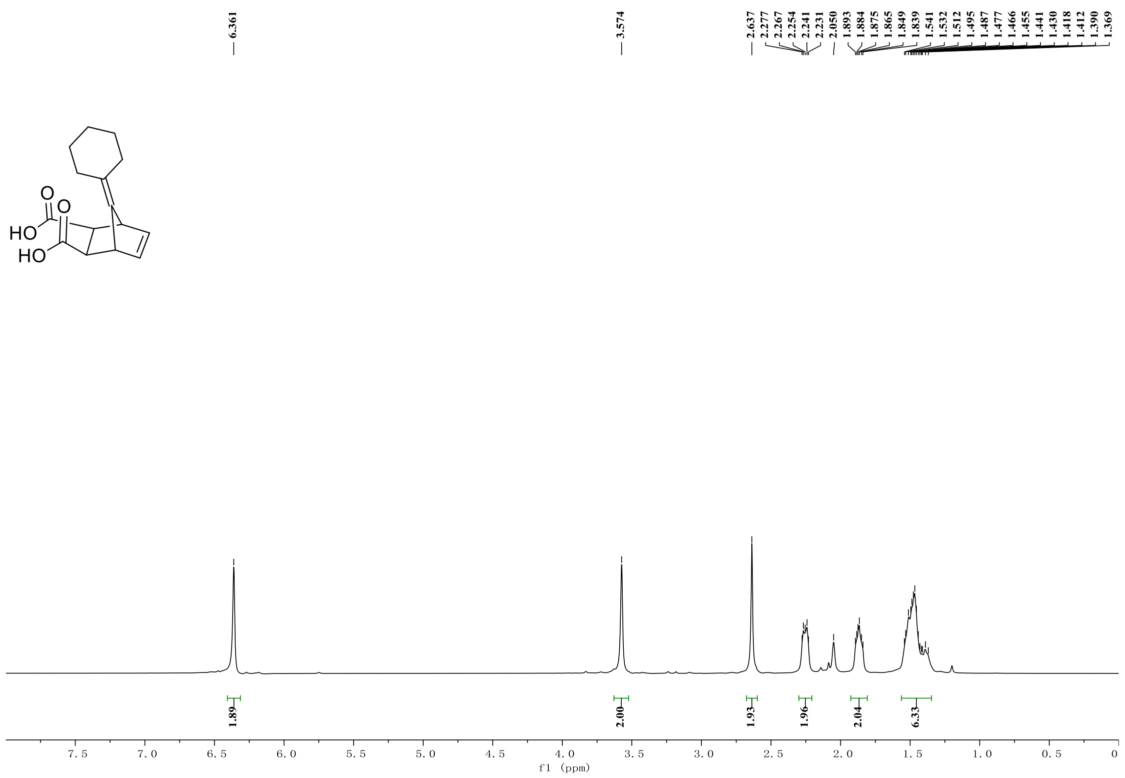

^{1}H NMR was recorded on Bruker 500 MHz; Solvent: Acetone-d_6

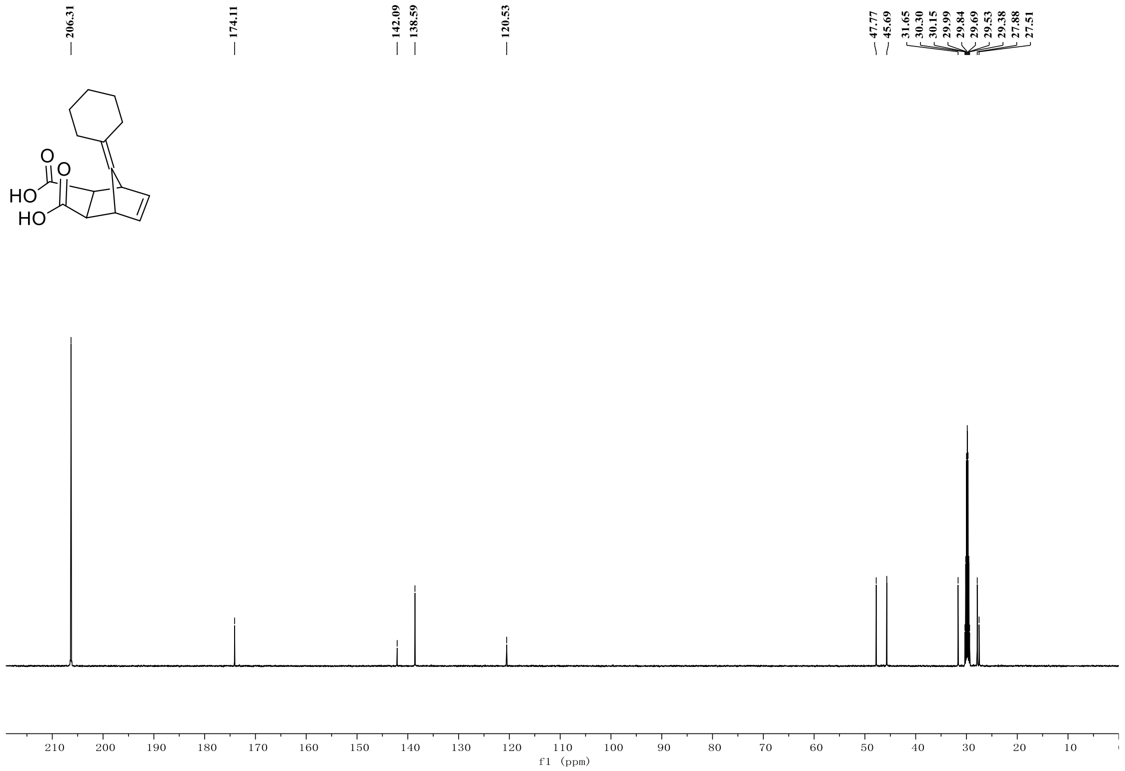

^{13}C NMR was recorded on Bruker 126 MHz; Solvent: Acetone-d_6

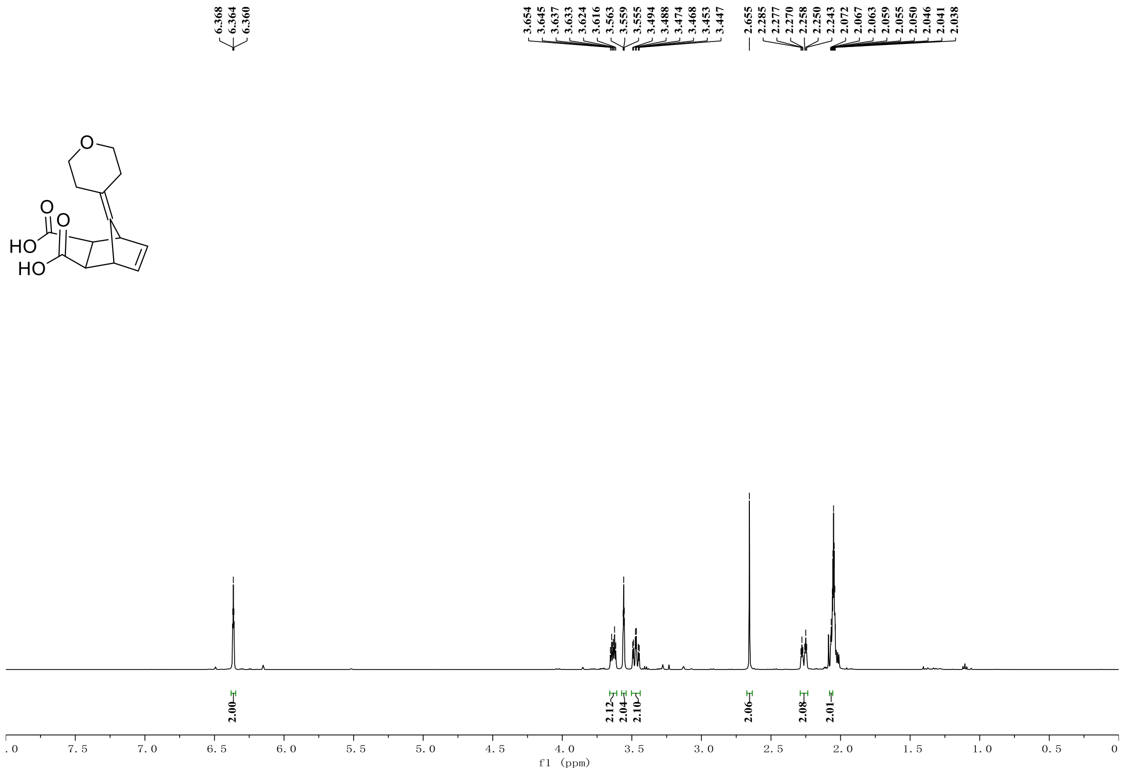

^{1}H NMR was recorded on Bruker 500 MHz; Solvent: Acetone-d_6

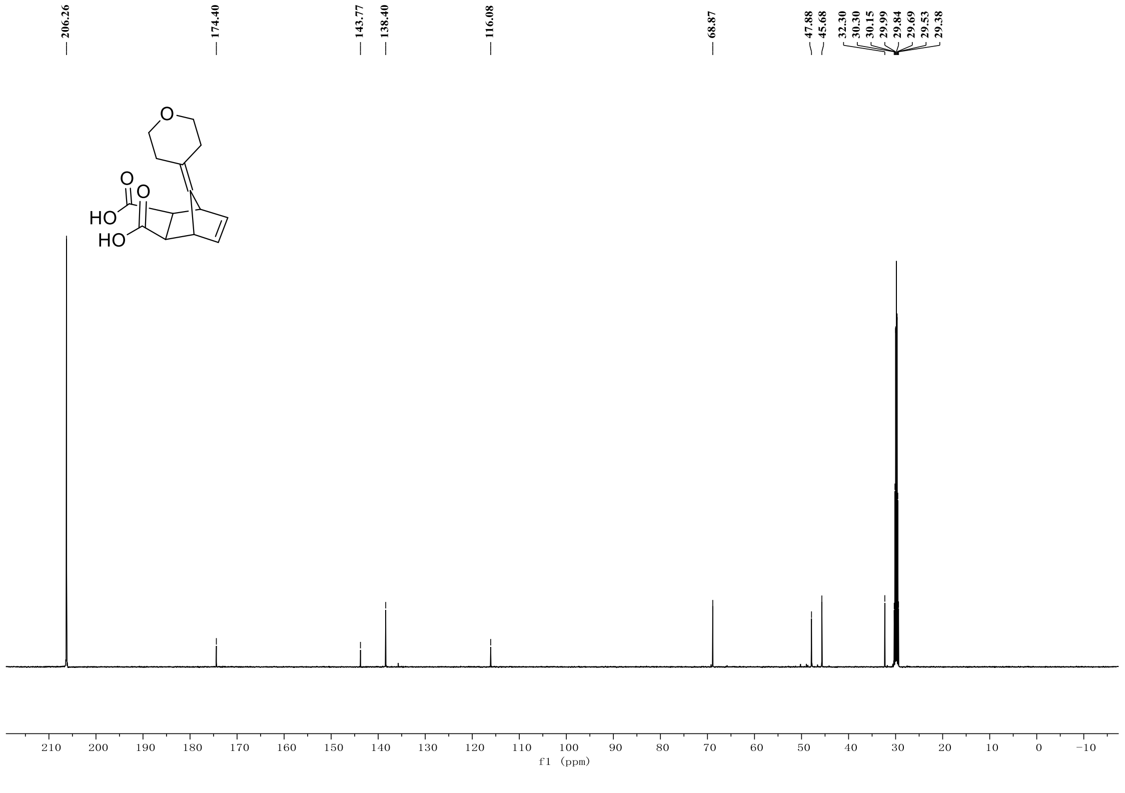

^{13}C NMR was recorded on Bruker 126 MHz; Solvent: Acetone-d_6

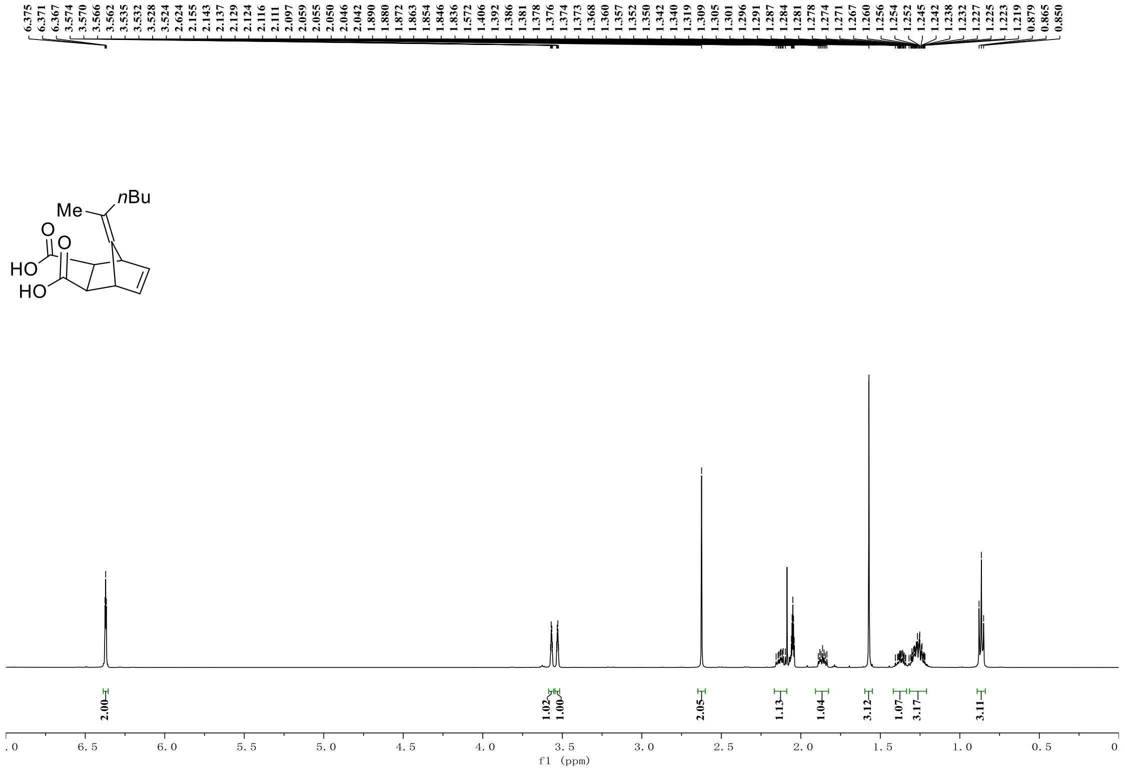

^{1}H NMR was recorded on Bruker 500 MHz; Solvent: Acetone-d_6

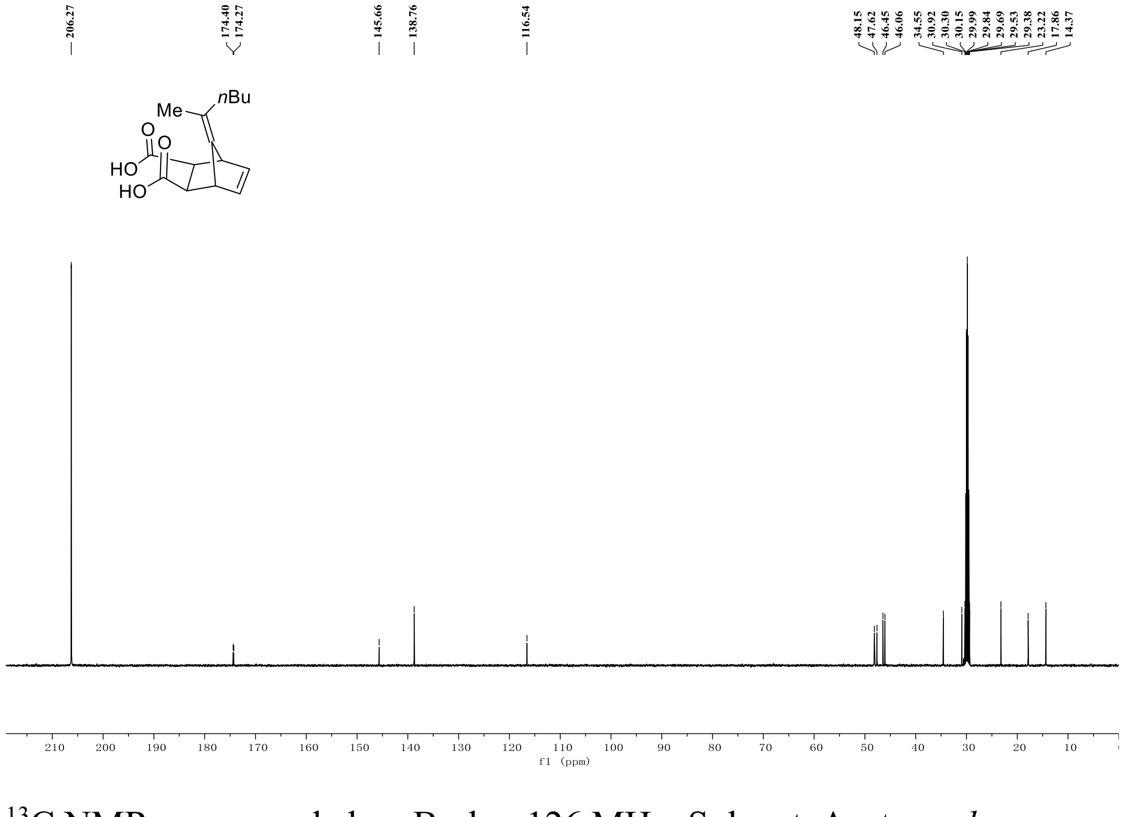

^{13}C NMR was recorded on Bruker 126 MHz; Solvent: Acetone-d_6

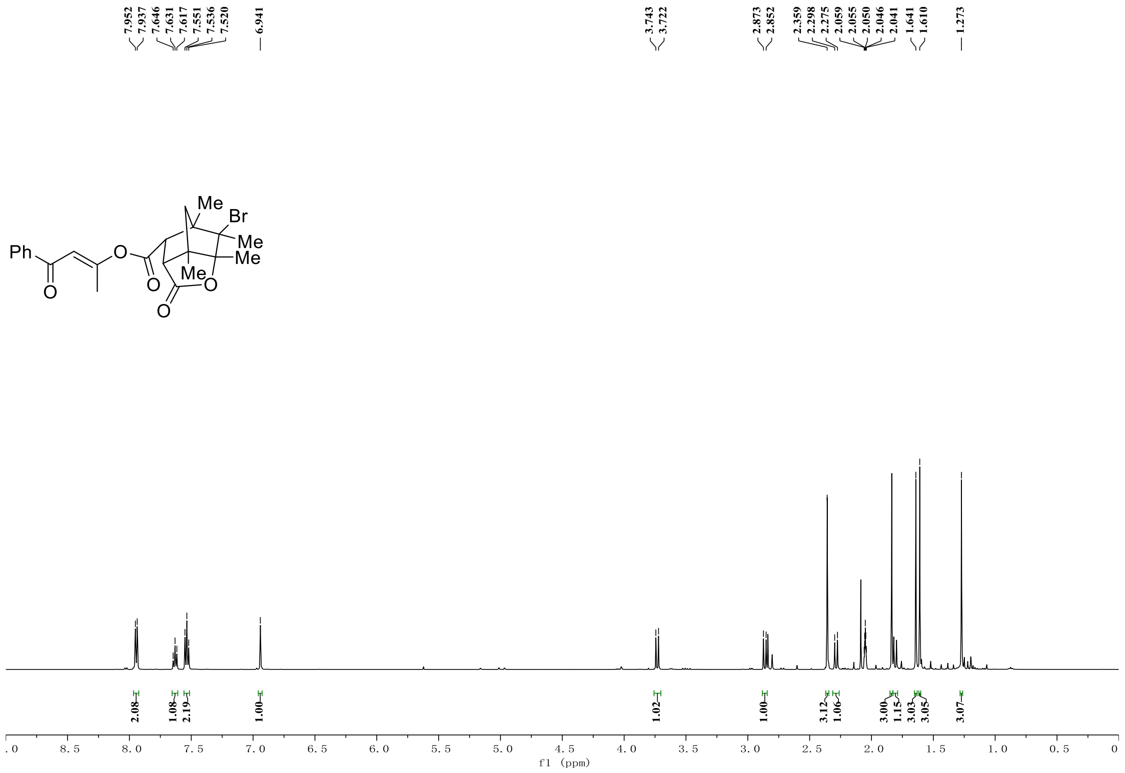

^{1}H NMR was recorded on Bruker 500 MHz; Solvent: Acetone-d_6

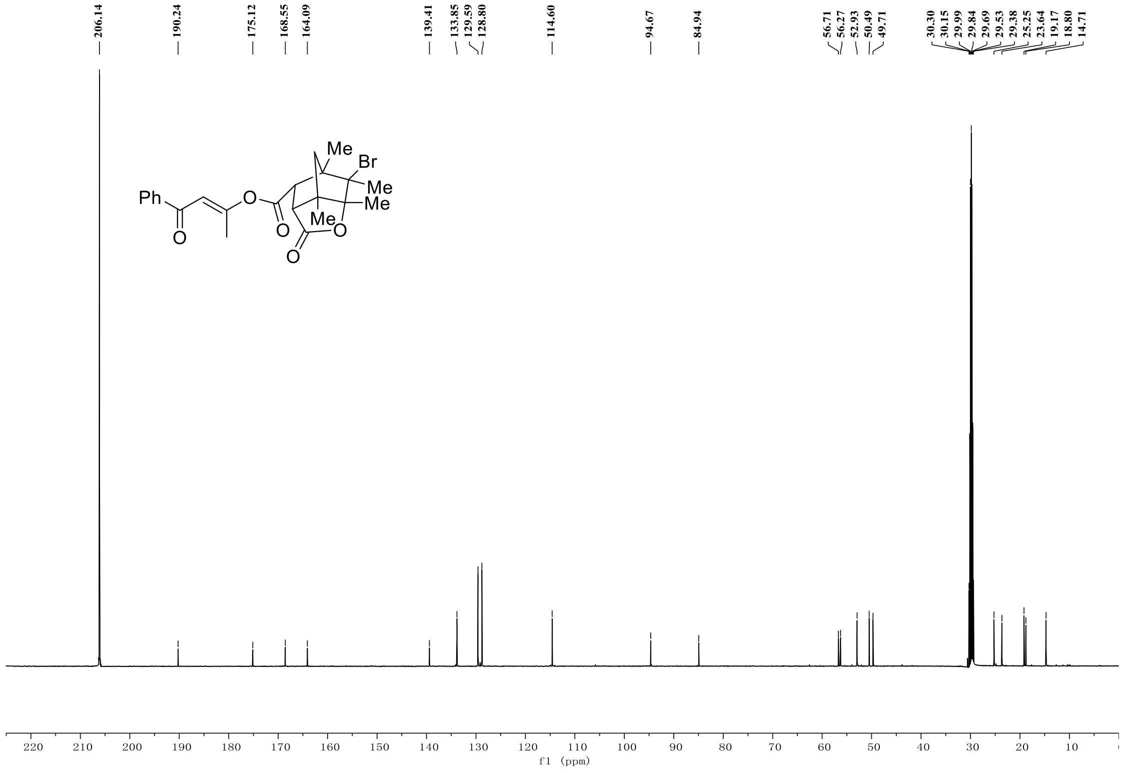

^{13}C NMR was recorded on Bruker 126 MHz; Solvent: Acetone-d_6

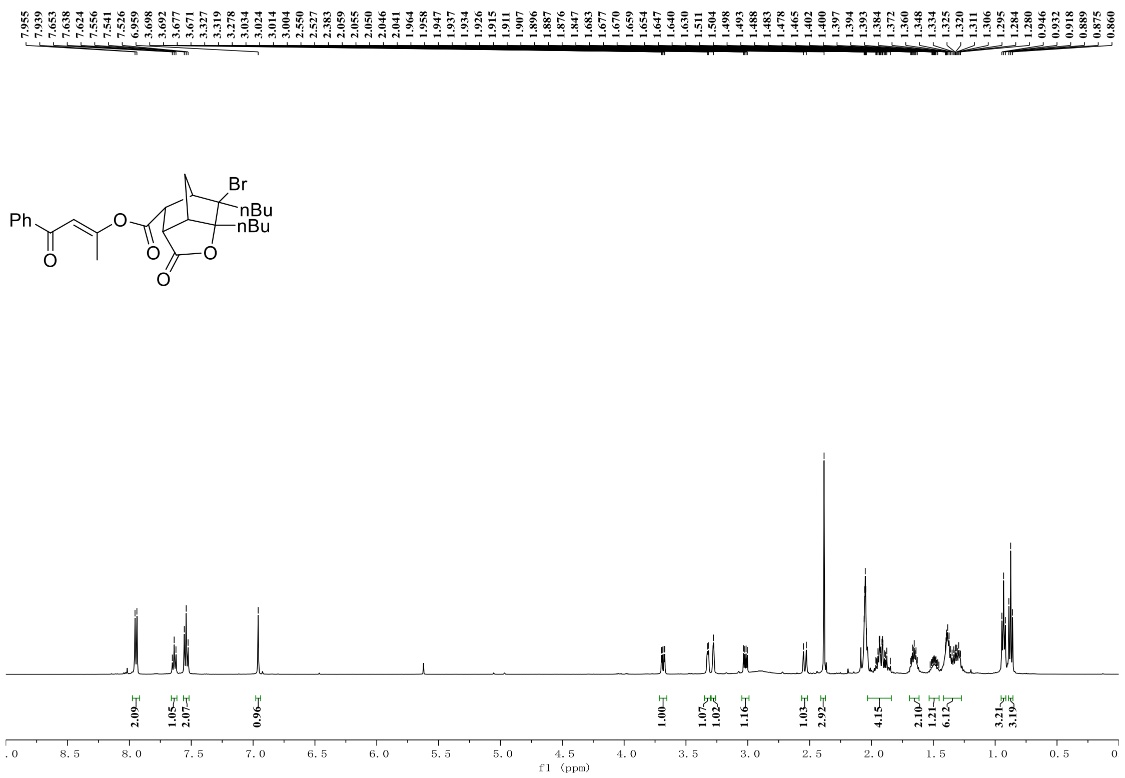

^{1}H NMR was recorded on Bruker 500 MHz; Solvent: Acetone-d_6

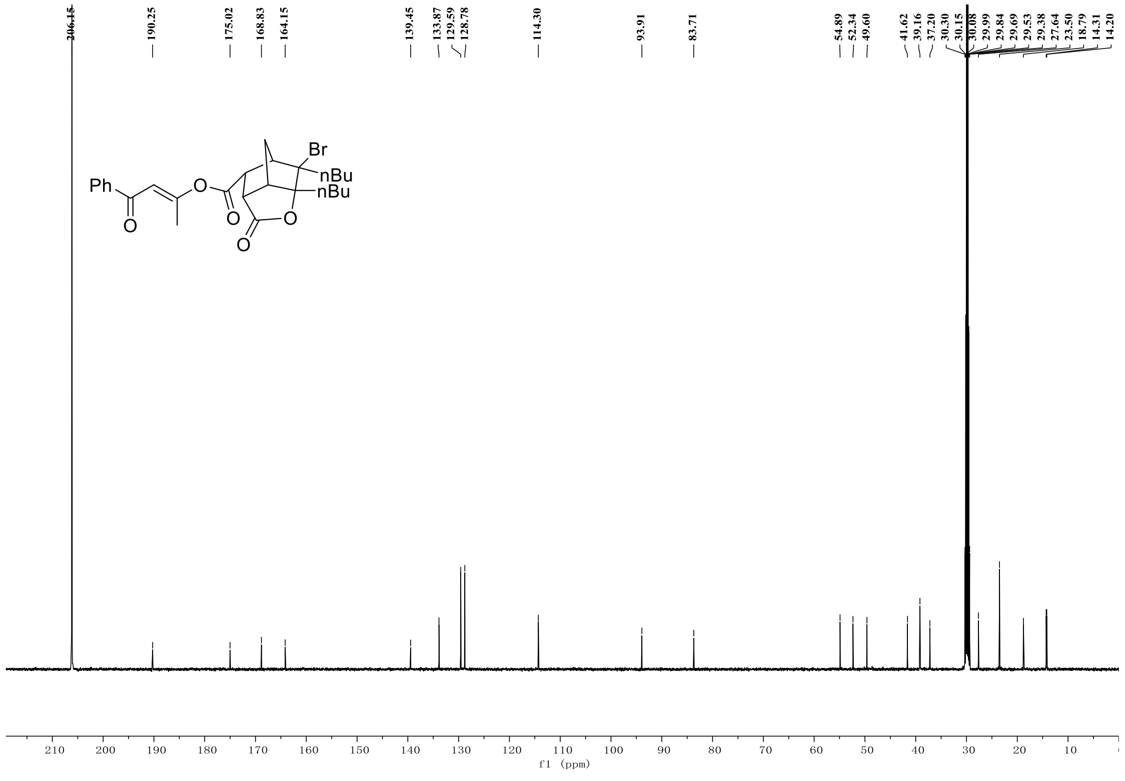

^{13}C NMR was recorded on Bruker 126 MHz; Solvent: Acetone-d_6

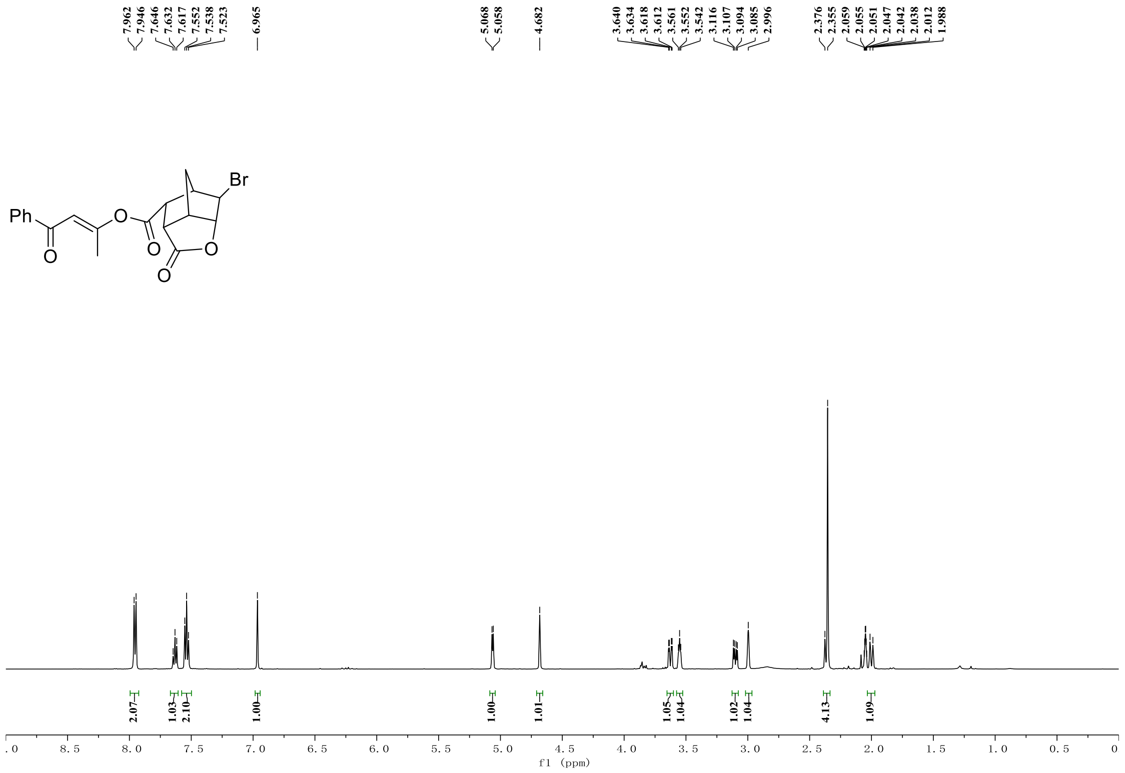

^{1}H NMR was recorded on Bruker 500 MHz; Solvent: Acetone-d_6

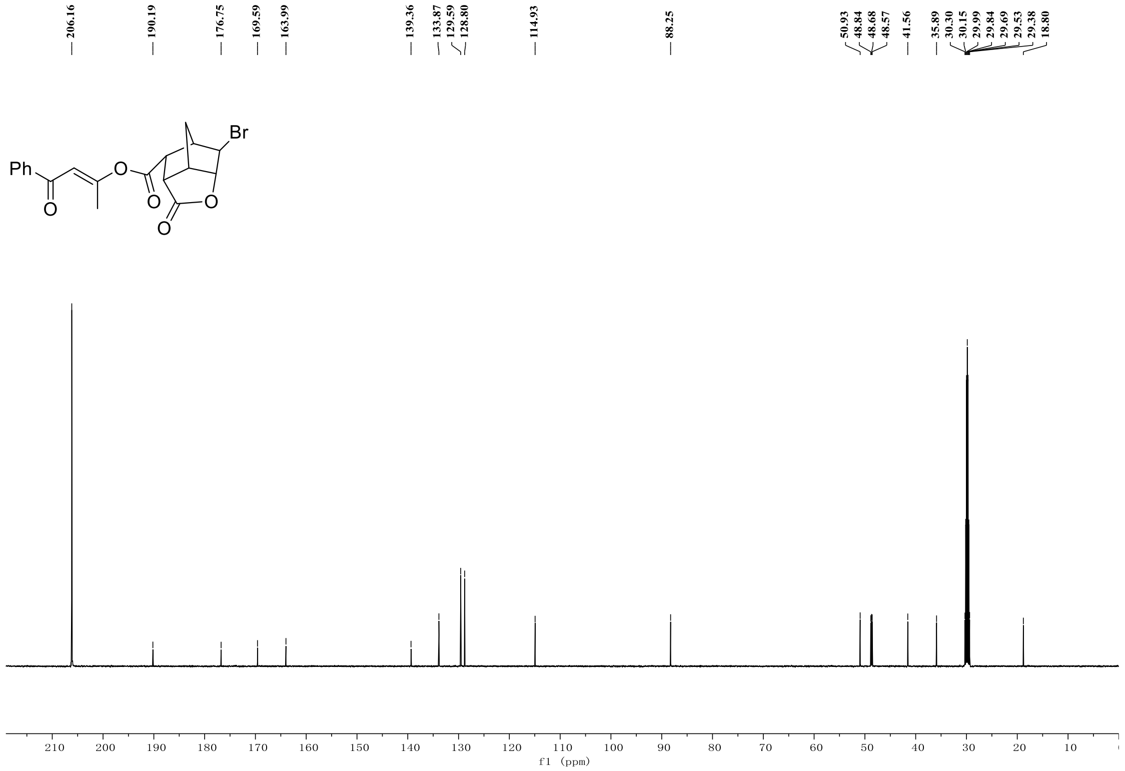

^{13}C NMR was recorded on Bruker 126 MHz; Solvent: Acetone-d_6

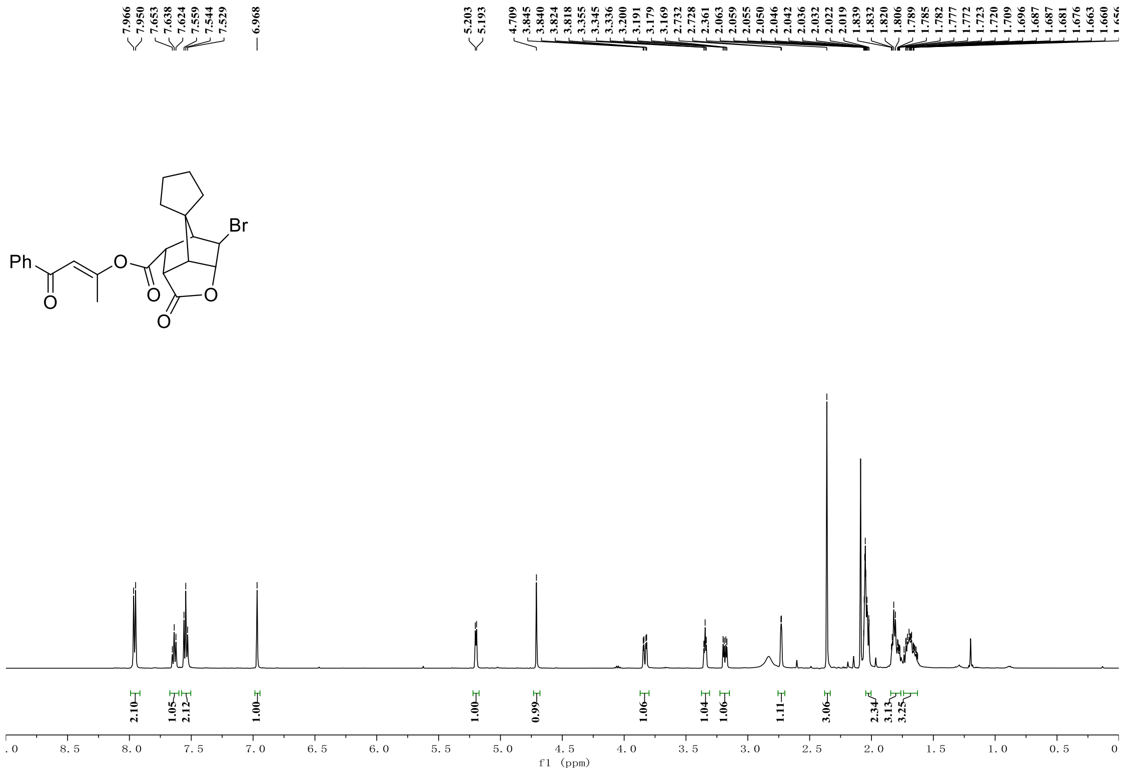

^{1}H NMR was recorded on Bruker 500 MHz; Solvent: Acetone-d_6

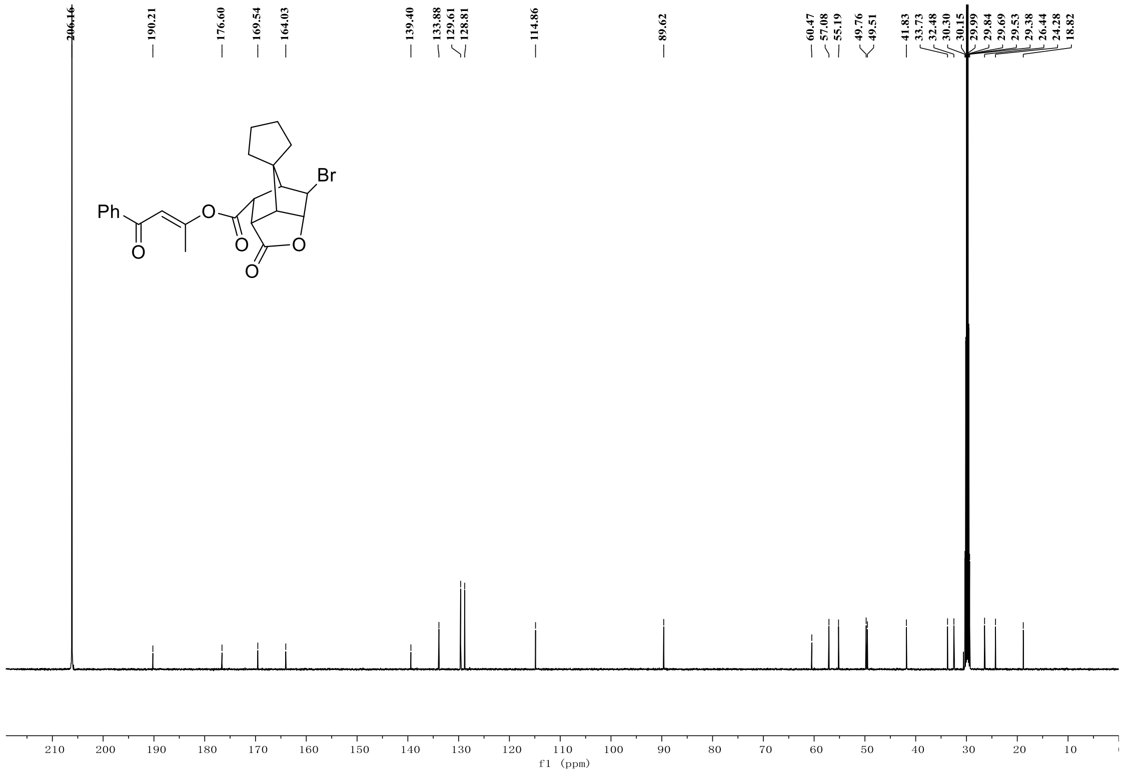

^{13}C NMR was recorded on Bruker 126 MHz; Solvent: Acetone-d_6

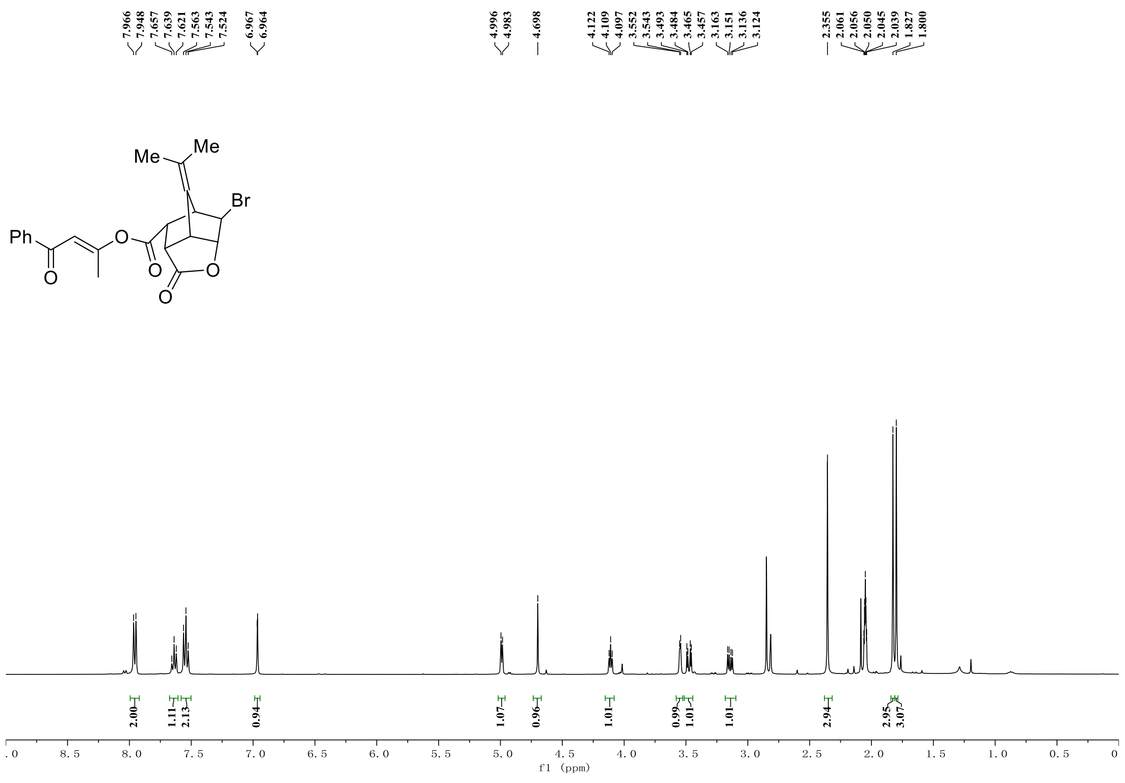

^{1}H NMR was recorded on Bruker 400 MHz; Solvent: Acetone-d_6

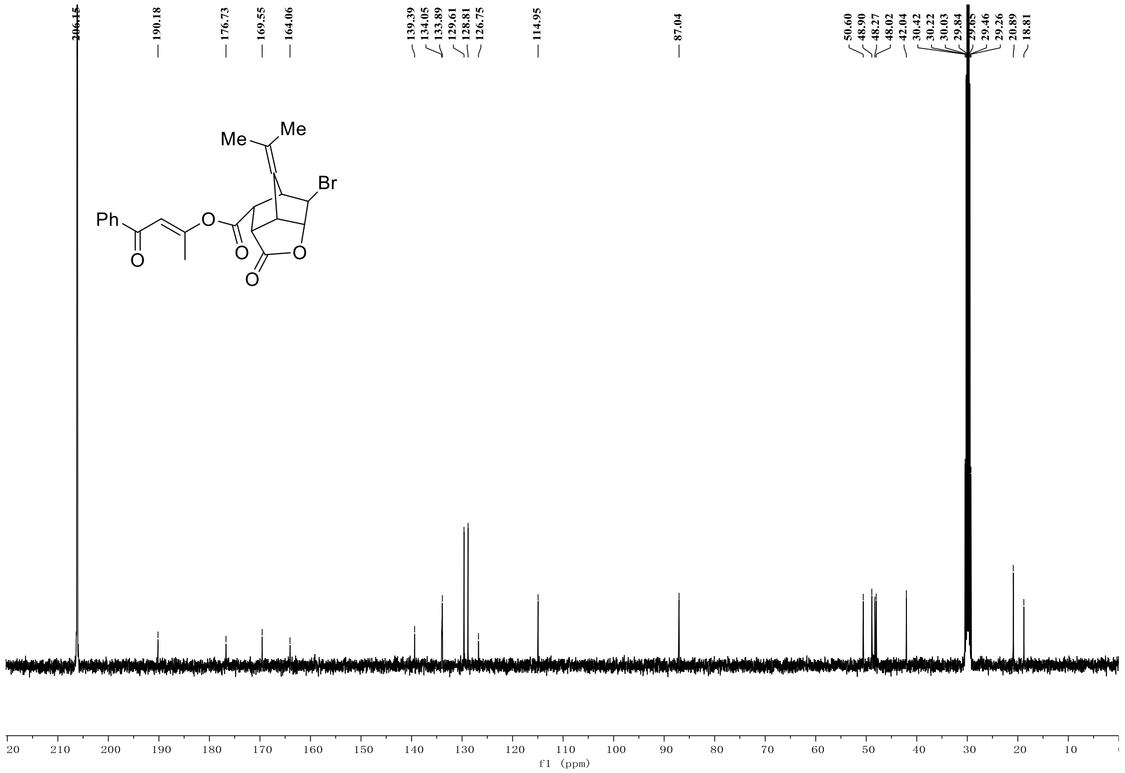

^{13}C NMR was recorded on Bruker 101 MHz; Solvent: Acetone-d_6

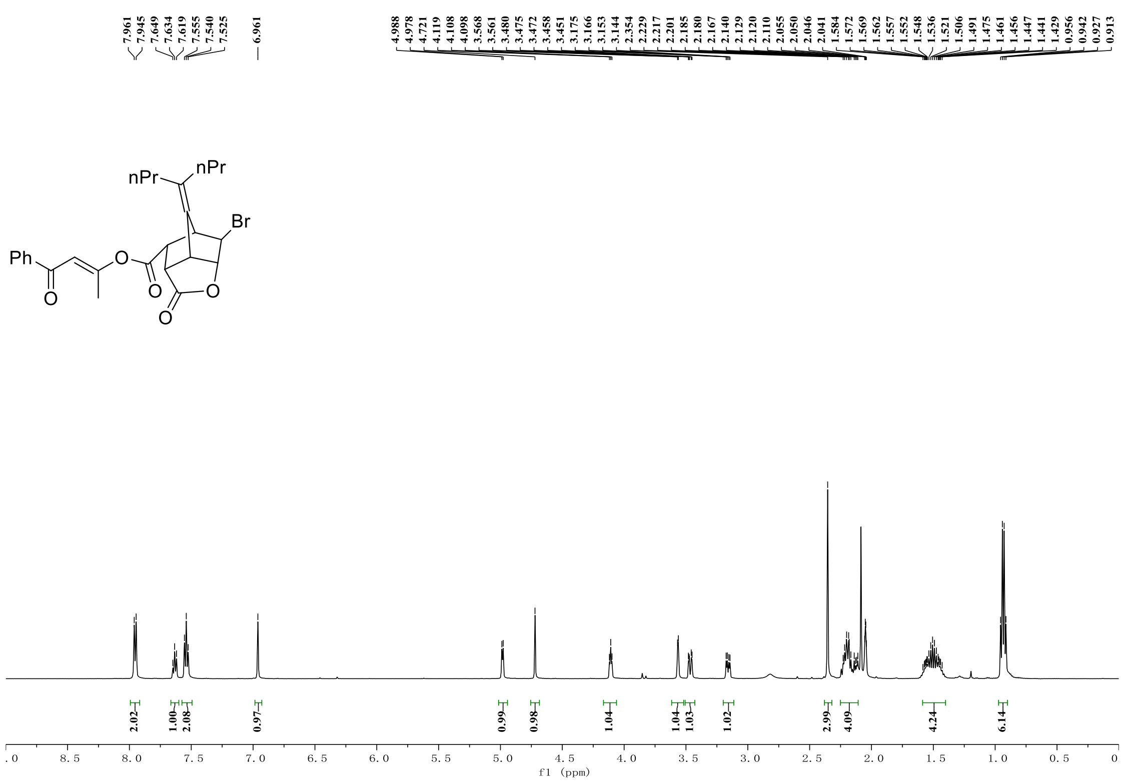

^{1}H NMR was recorded on Bruker 500 MHz; Solvent: Acetone-d_6

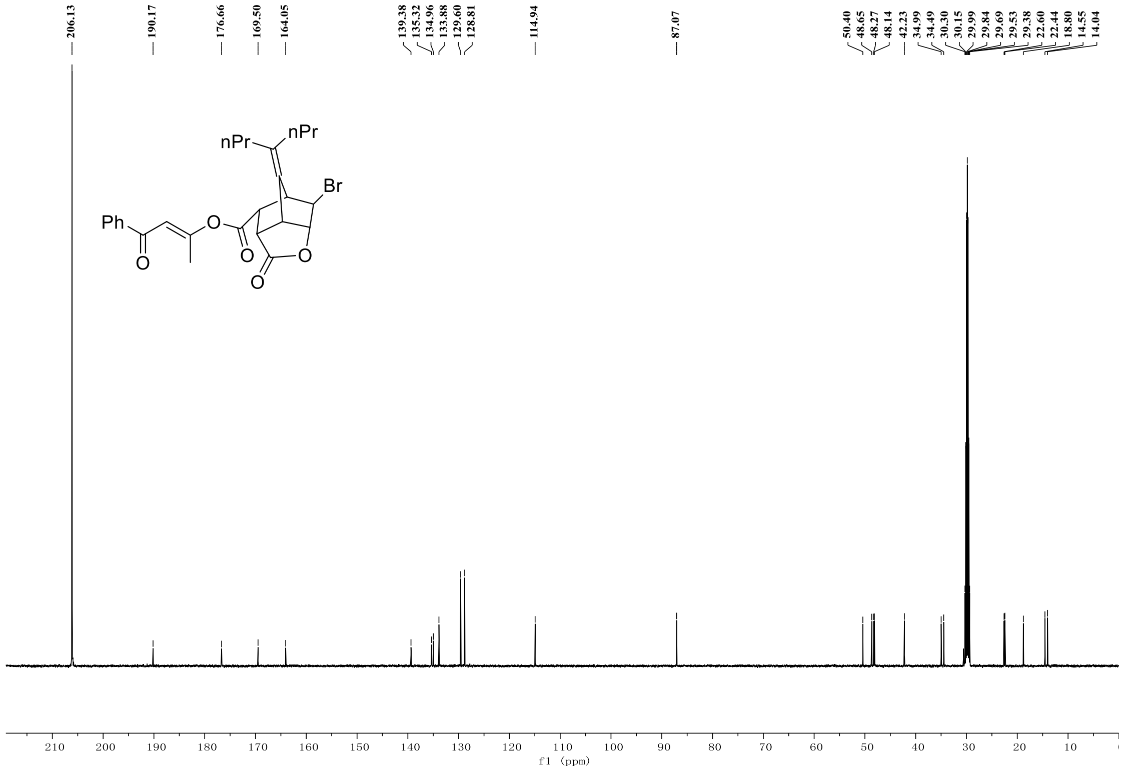

^{13}C NMR was recorded on Bruker 126 MHz; Solvent: DMSO-d_6

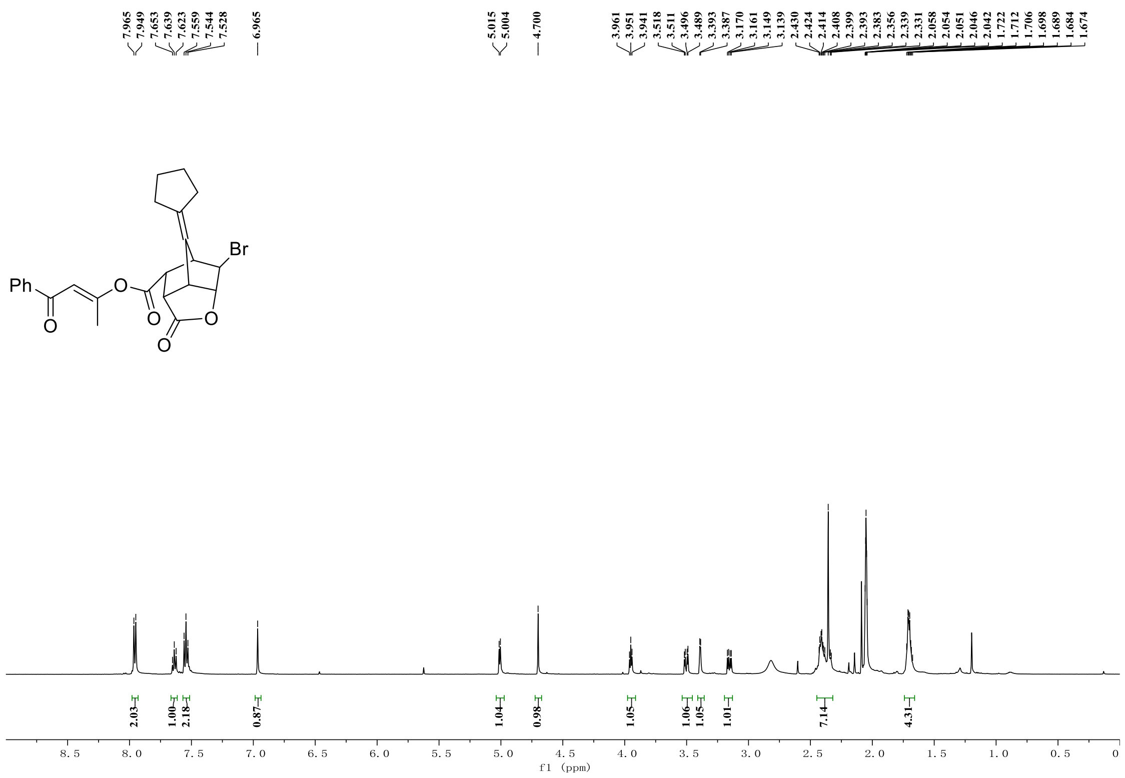

^{1}H NMR was recorded on Bruker 500 MHz; Solvent: Acetone-d_6

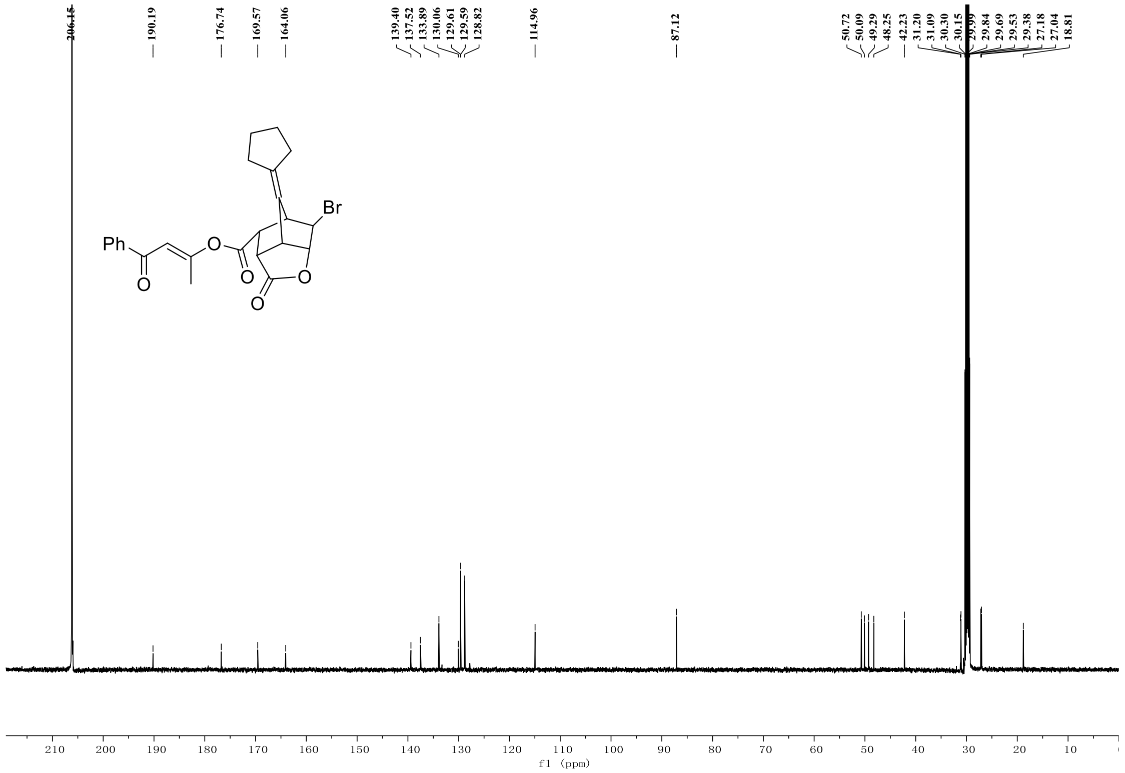

^{13}C NMR was recorded on Bruker 126 MHz; Solvent: Acetone-d_6

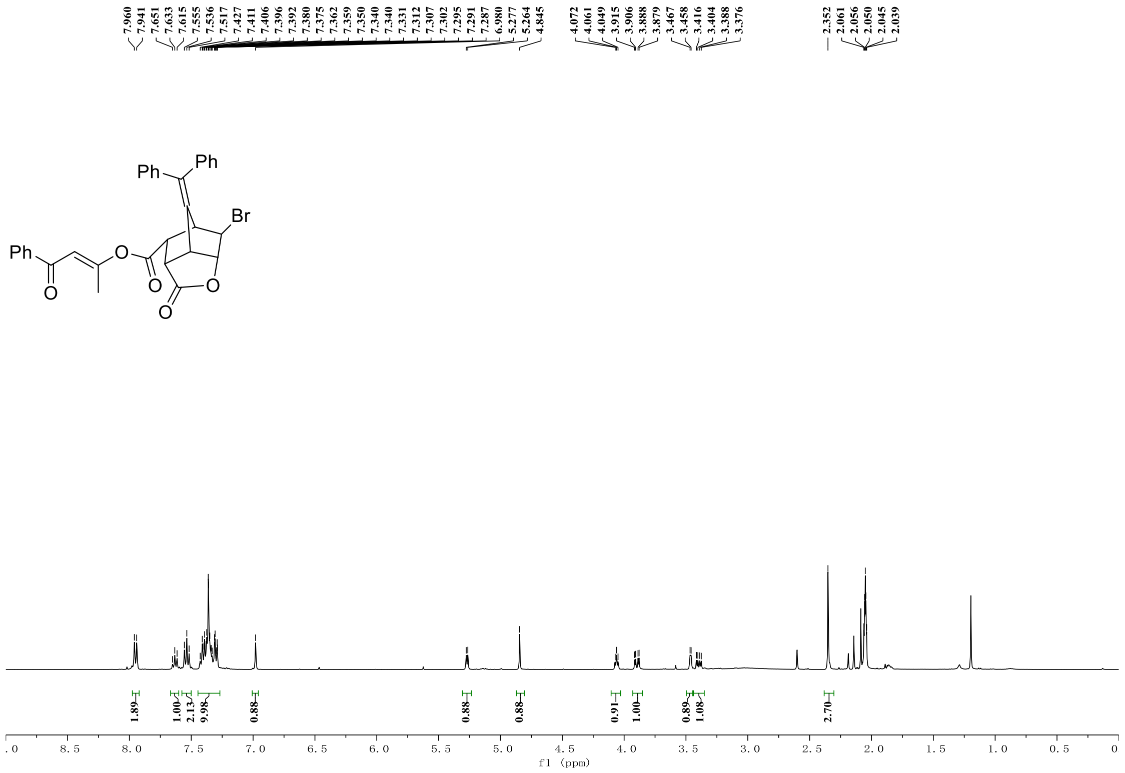

^{1}H NMR was recorded on Bruker 400 MHz; Solvent: Acetone-d_6

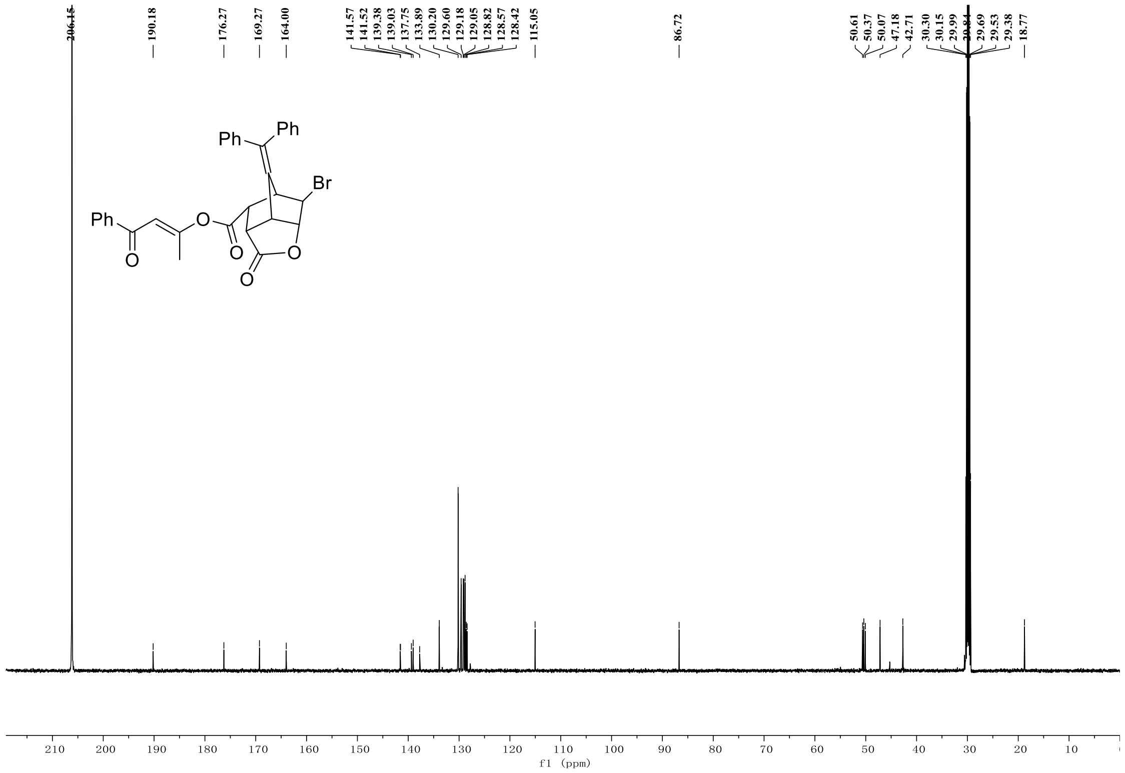

^{13}C NMR was recorded on Bruker 101 MHz; Solvent: Acetone-d_6

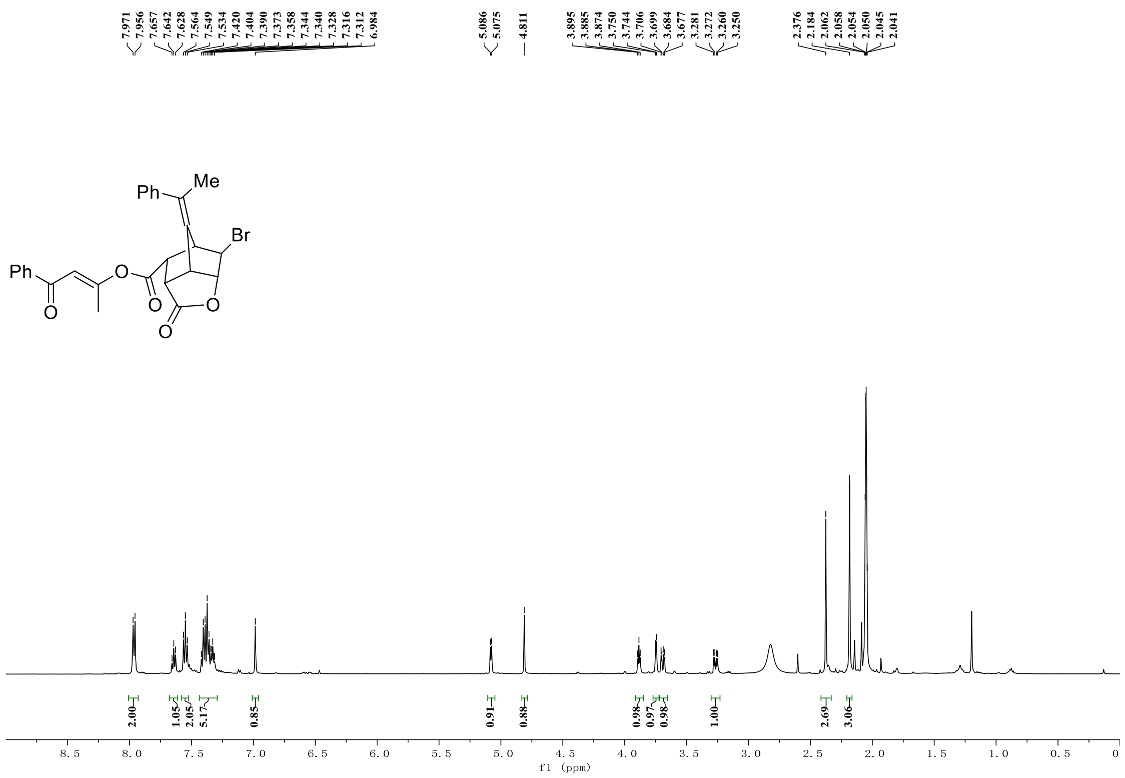

^{1}H NMR was recorded on Bruker 500 MHz; Solvent: Acetone-d_6

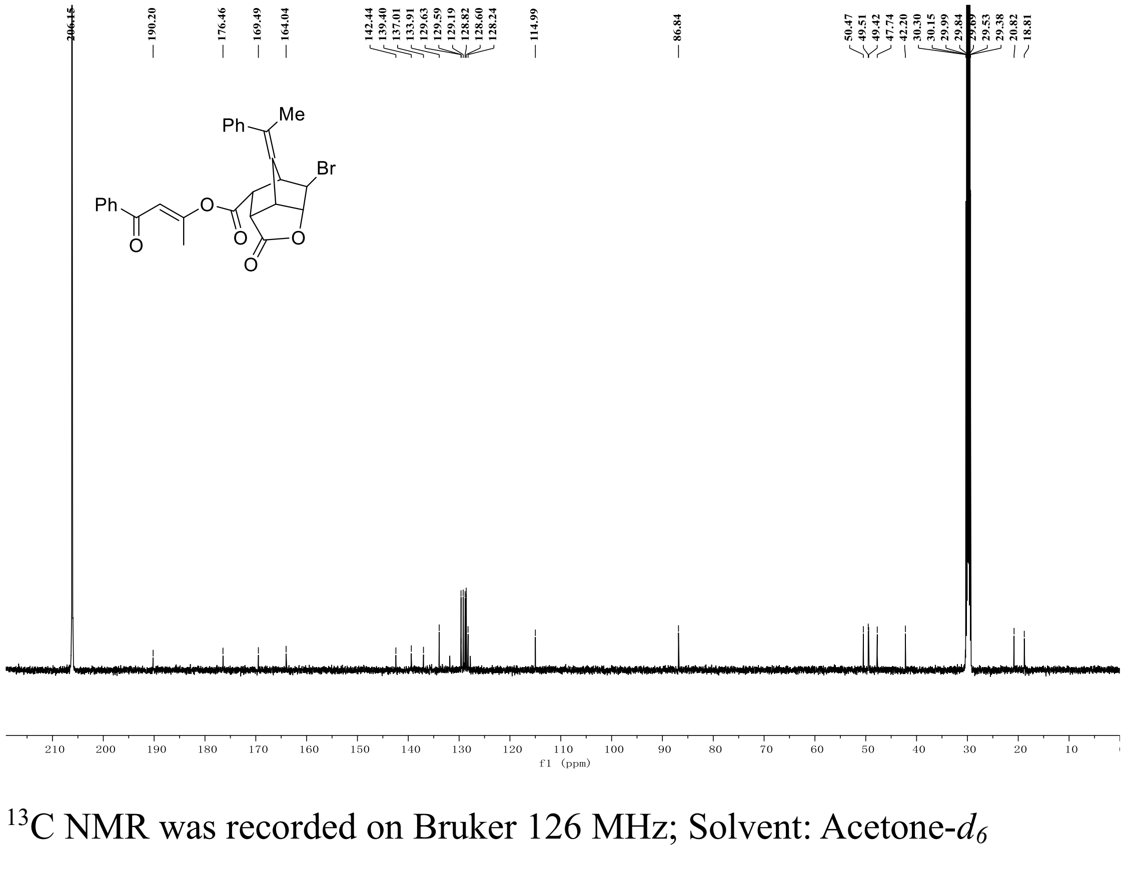

^{13}C NMR was recorded on Bruker 126 MHz; Solvent: Acetone-d_6

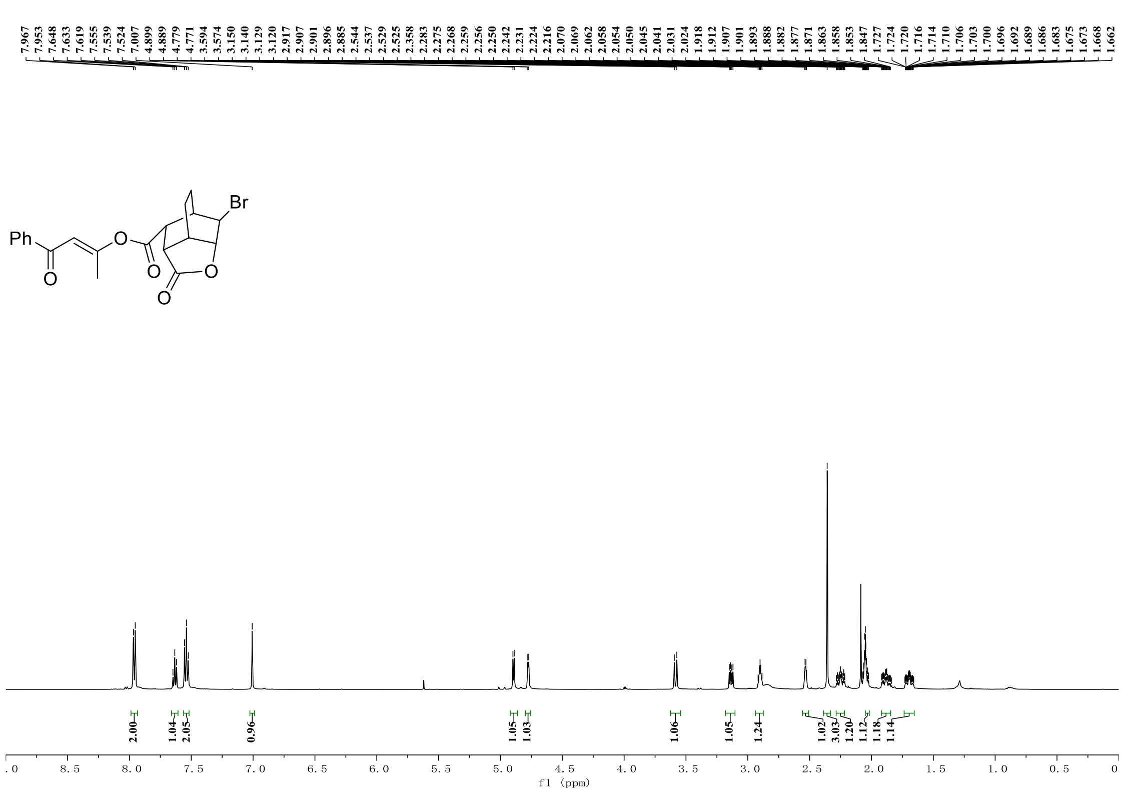

^{1}H NMR was recorded on Bruker 500 MHz; Solvent: Acetone-d_6

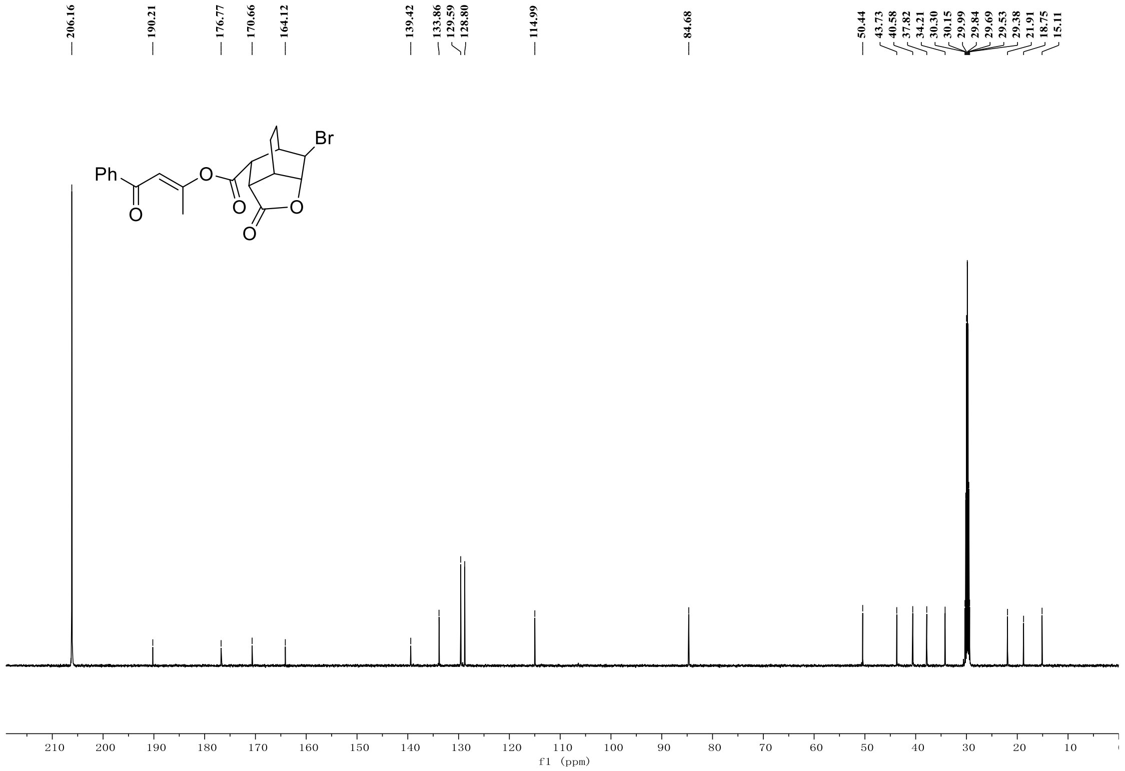

^{13}C NMR was recorded on Bruker 126 MHz; Solvent: Acetone-d_6

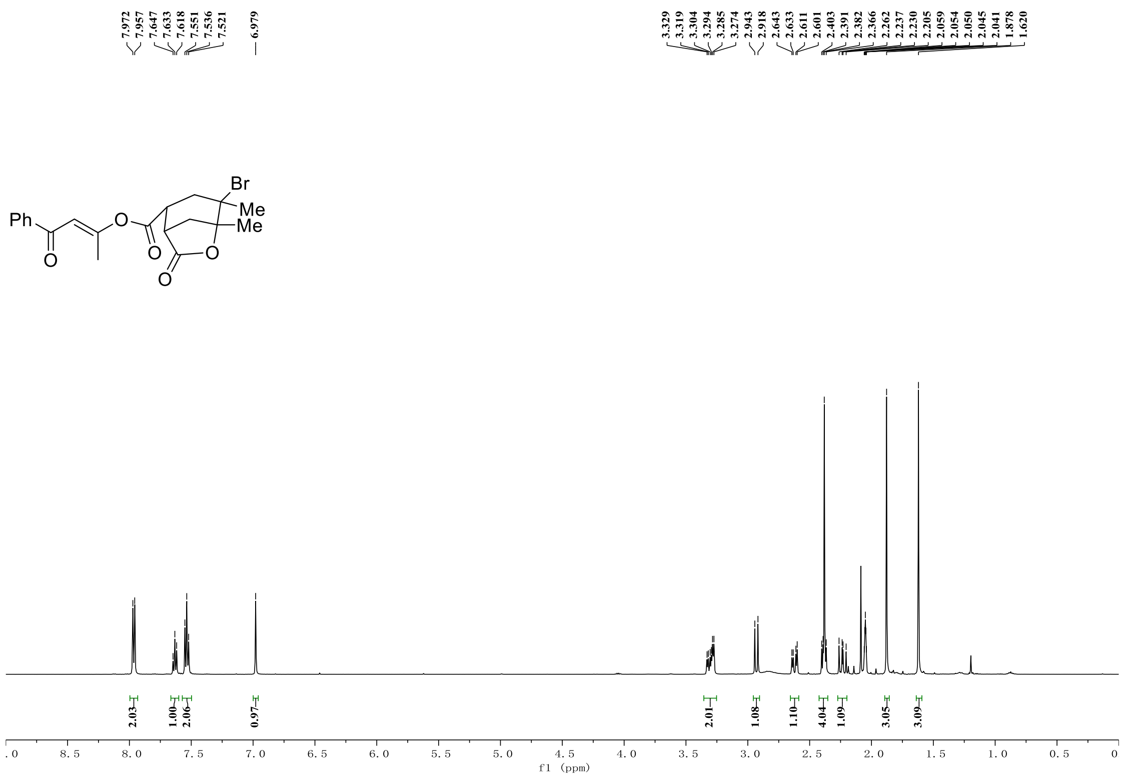

^{1}H NMR was recorded on Bruker 500 MHz; Solvent: Acetone-d_6

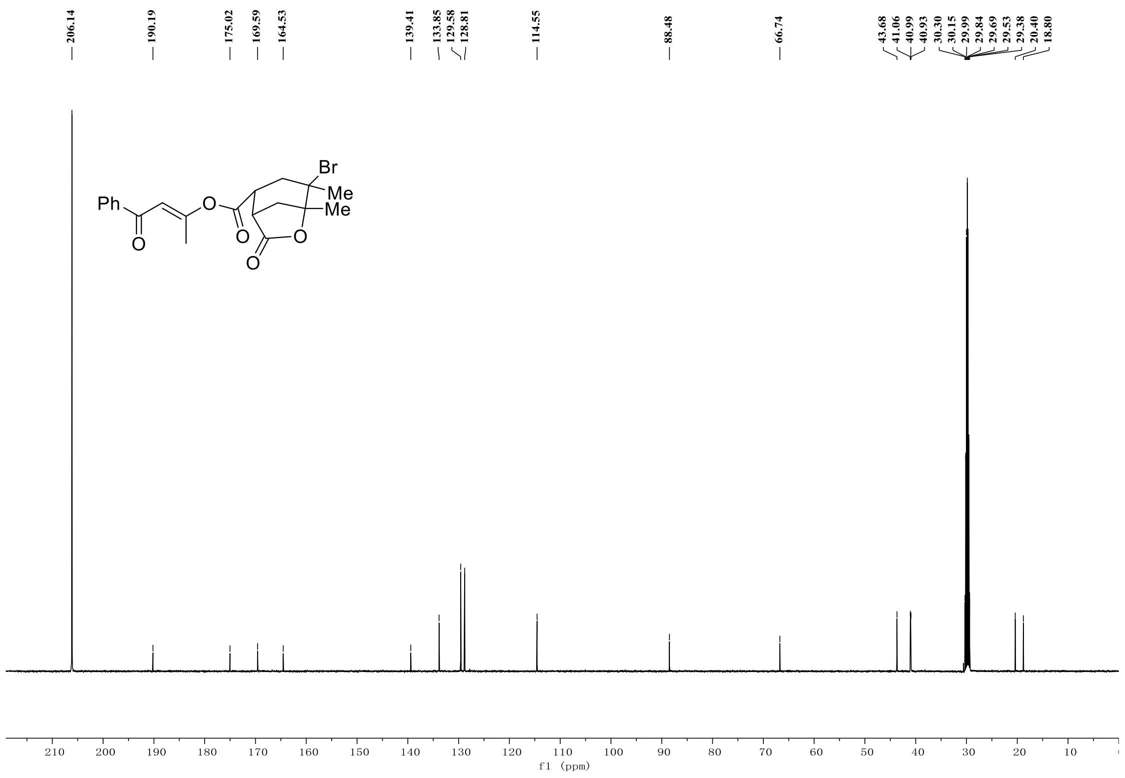

^{13}C NMR was recorded on Bruker 126 MHz; Solvent: Acetone-d_6

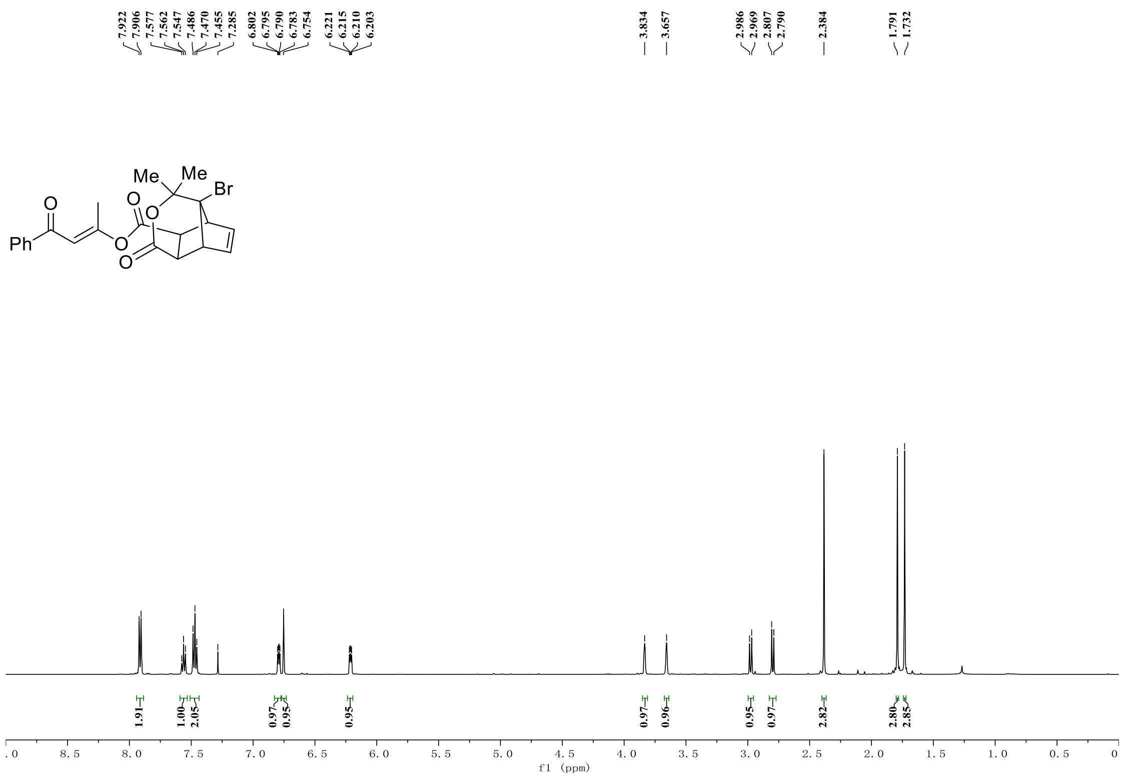

^{1}H NMR was recorded on Bruker 500 MHz; Solvent: $CDCl_3$

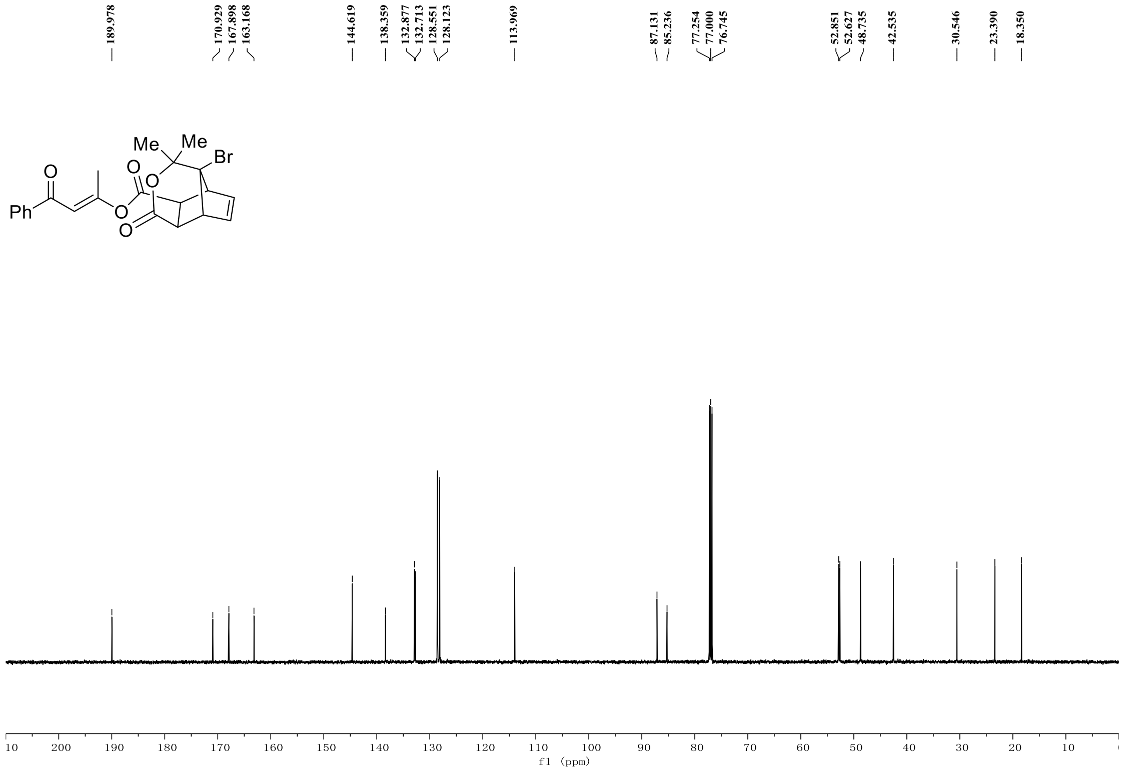

^{13}C NMR was recorded on Bruker 126 MHz; Solvent: $CDCl_3$

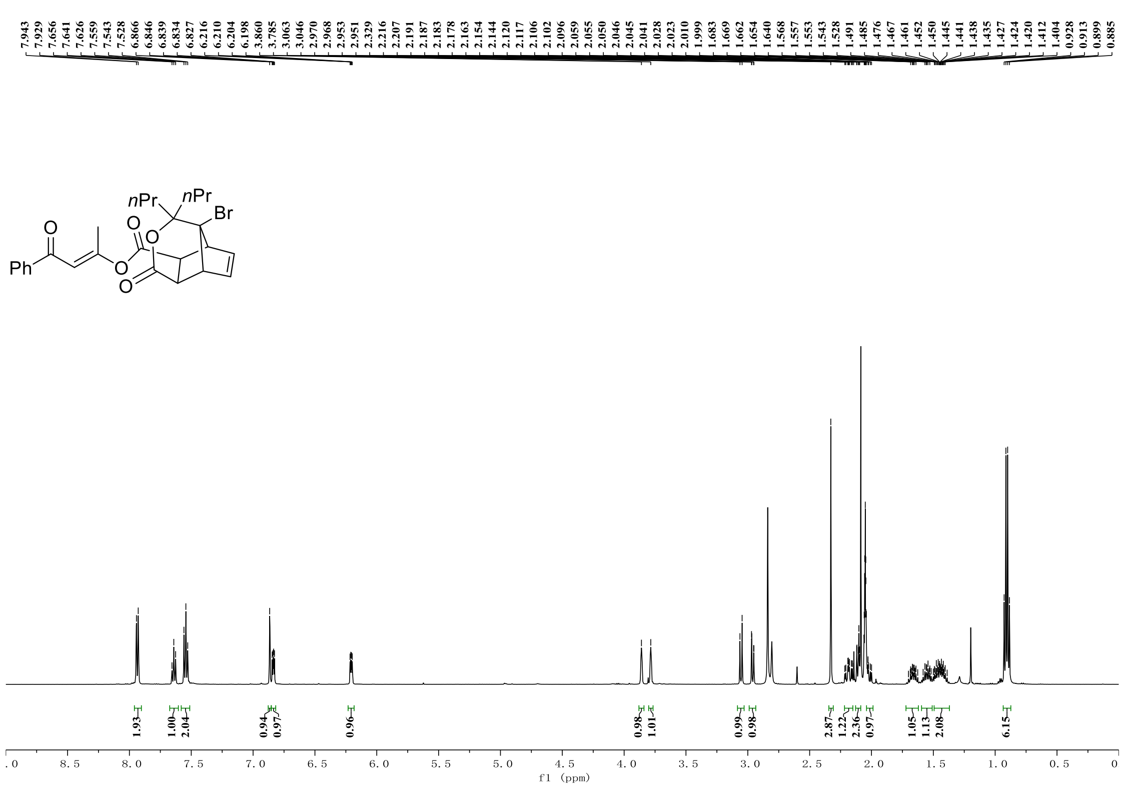

^{1}H NMR was recorded on Bruker 500 MHz; Solvent: Acetone-d_6

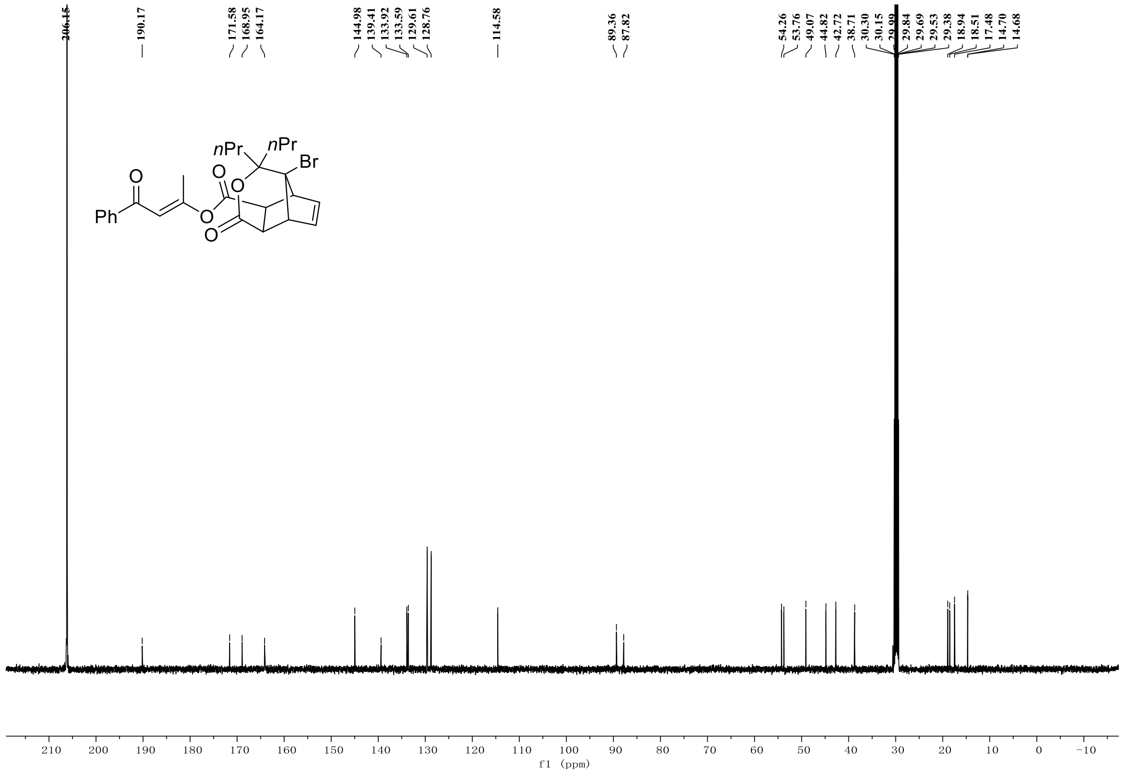

^{13}C NMR was recorded on Bruker 126 MHz; Solvent: Acetone-d_6

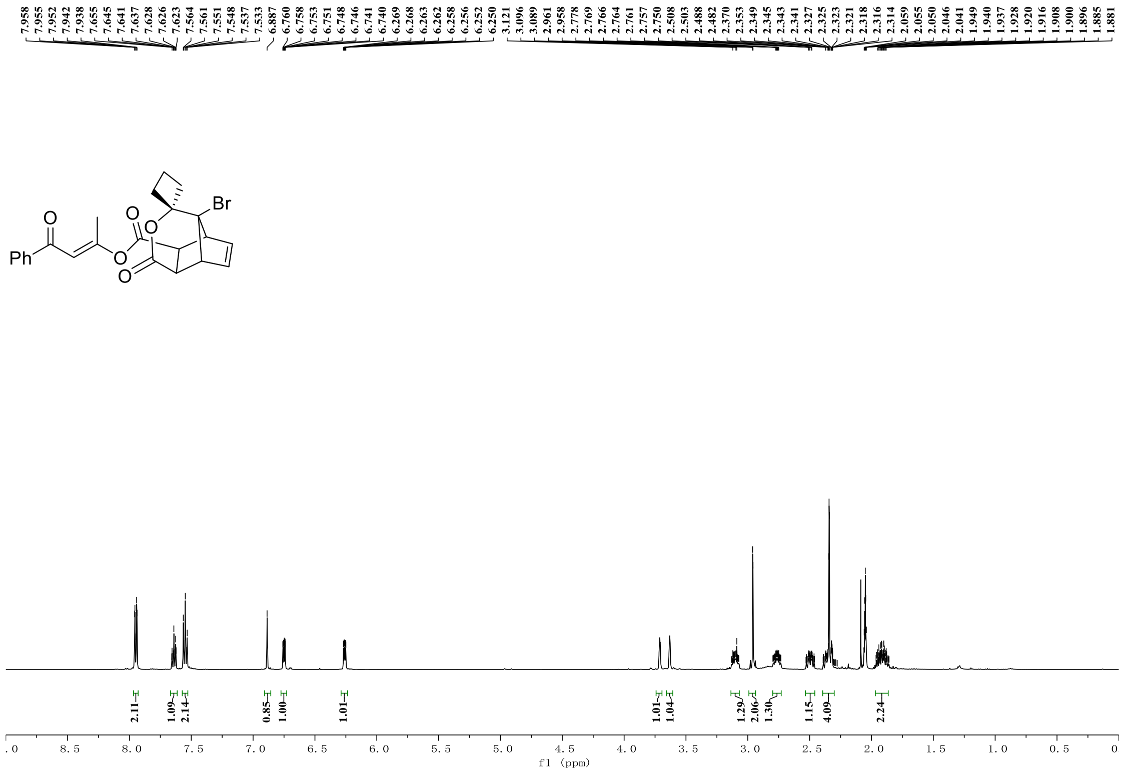

^{1}H NMR was recorded on Bruker 500 MHz; Solvent: Acetone-d_6

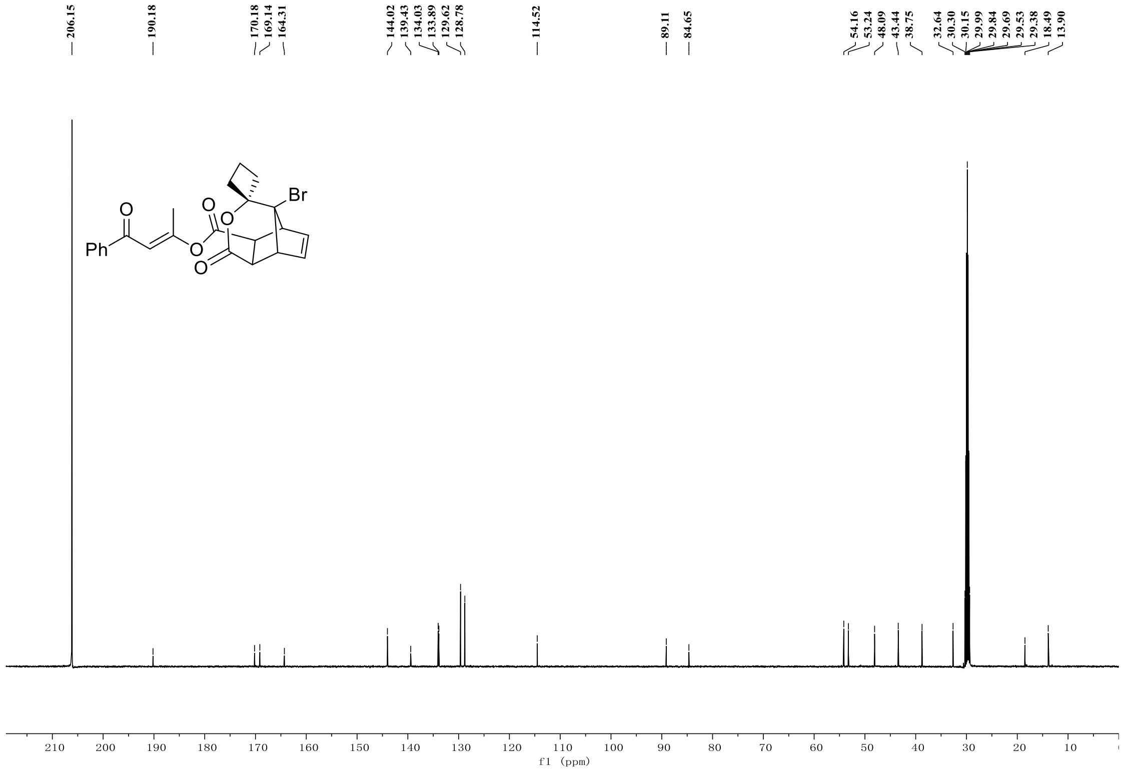

^{13}C NMR was recorded on Bruker 126 MHz; Solvent: Acetone-d_6

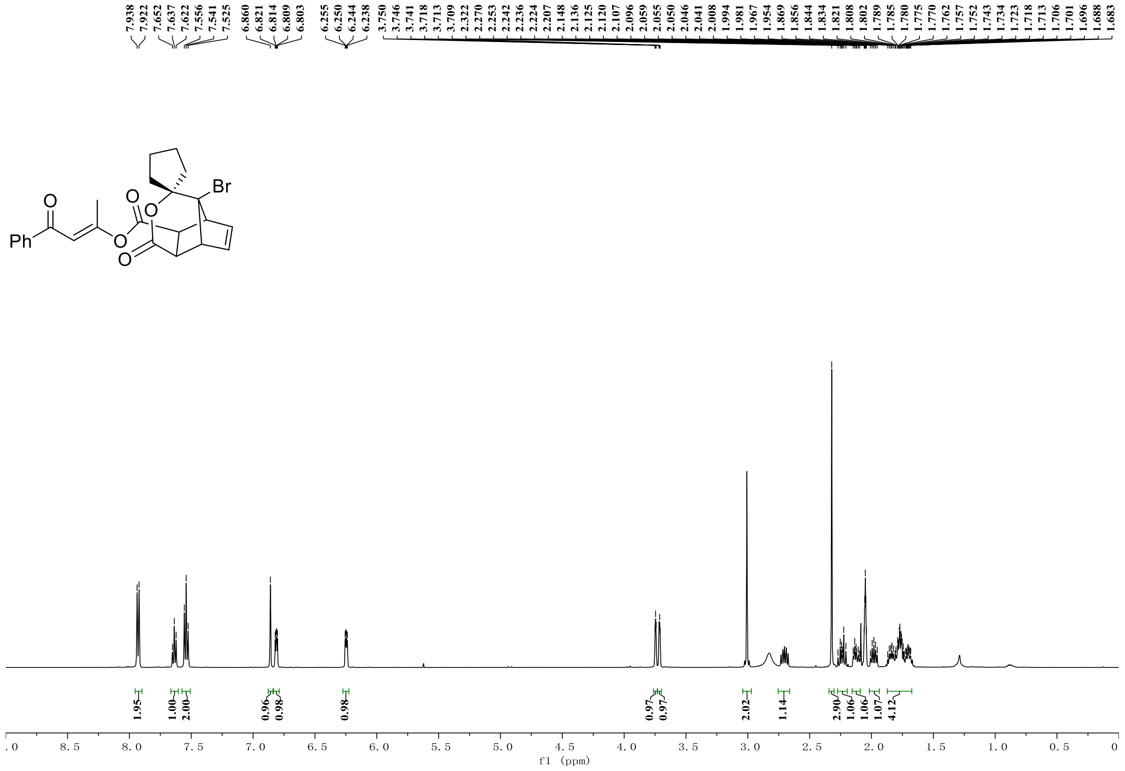

^{1}H NMR was recorded on Bruker 500 MHz; Solvent: Acetone-d_6

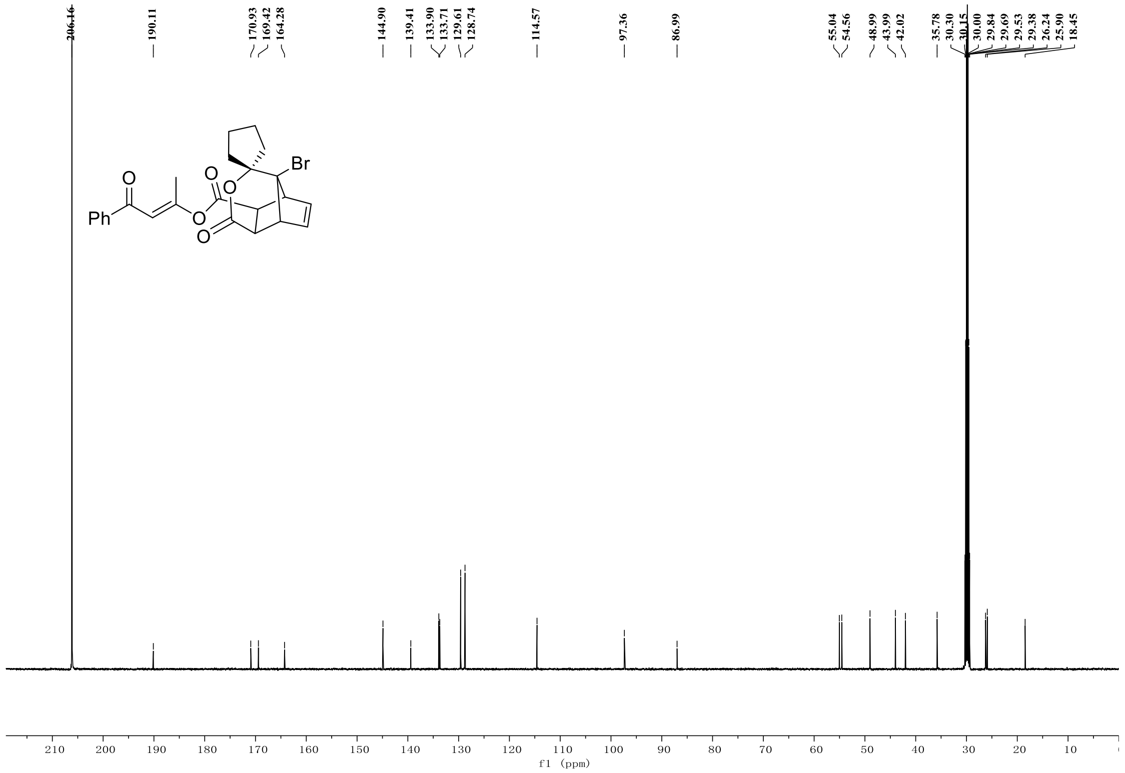

^{13}C NMR was recorded on Bruker 126 MHz; Solvent: Acetone-d_6

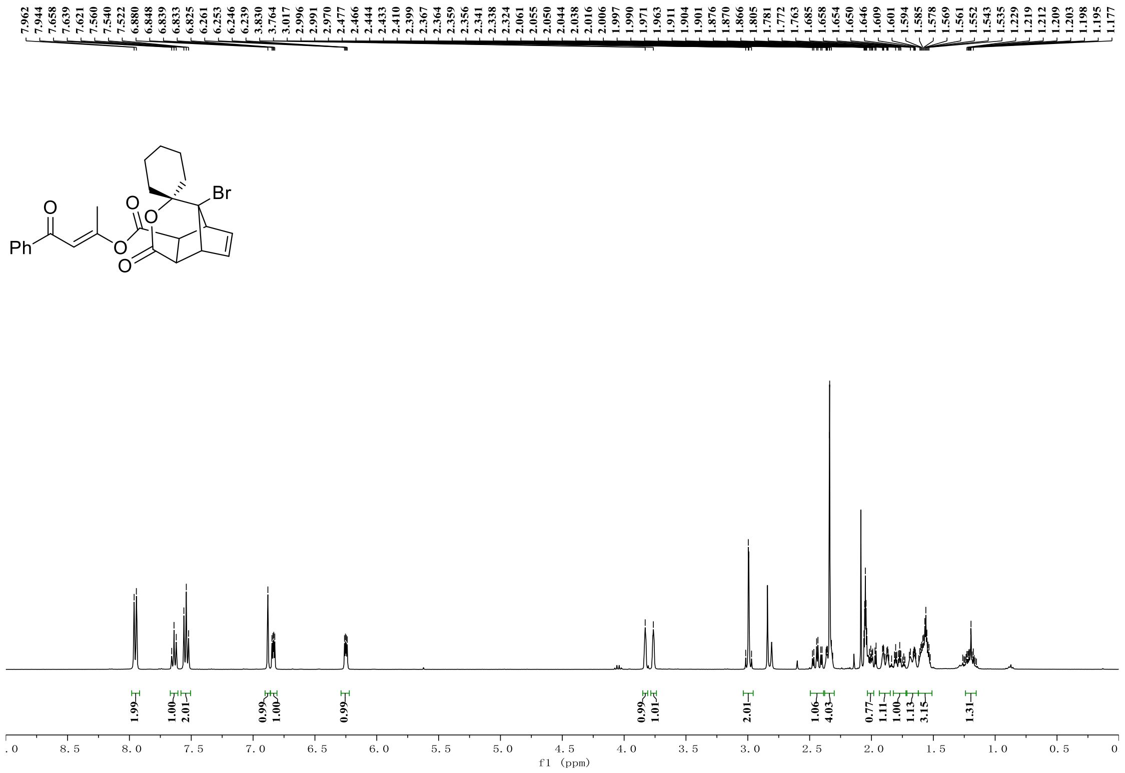

^{1}H NMR was recorded on Bruker 400 MHz; Solvent: Acetone-d_6

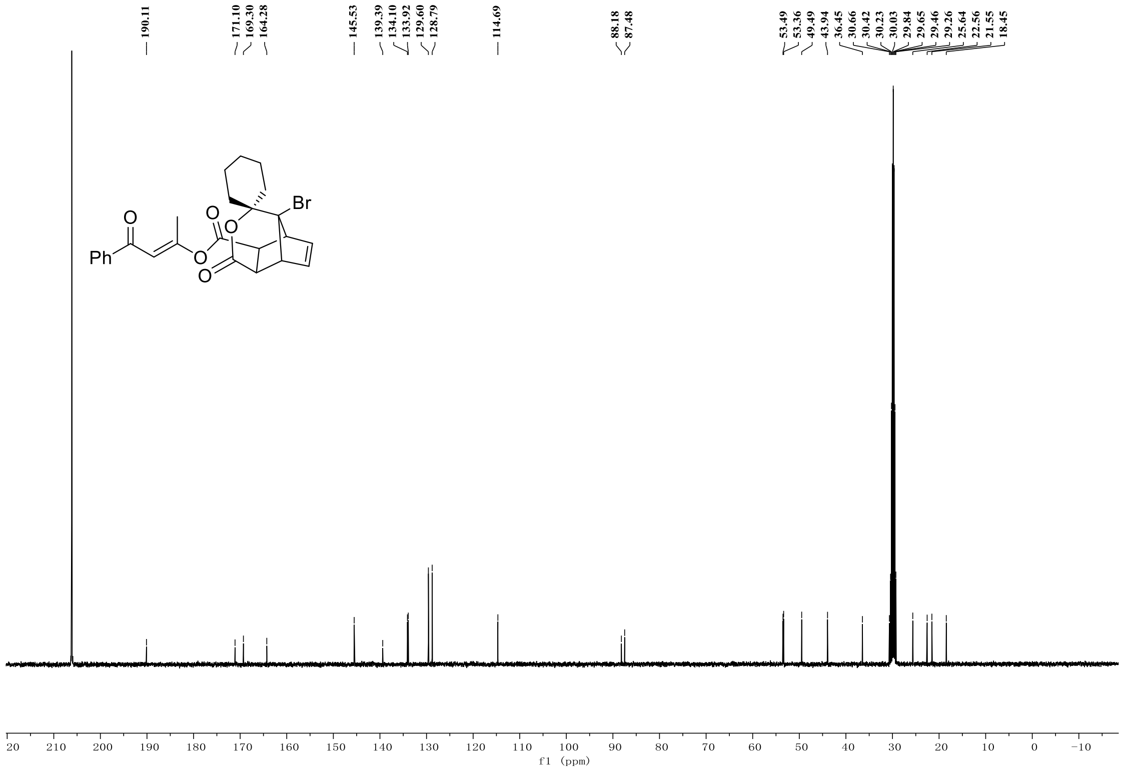

^{13}C NMR was recorded on Bruker 101 MHz; Solvent: Acetone-d_6

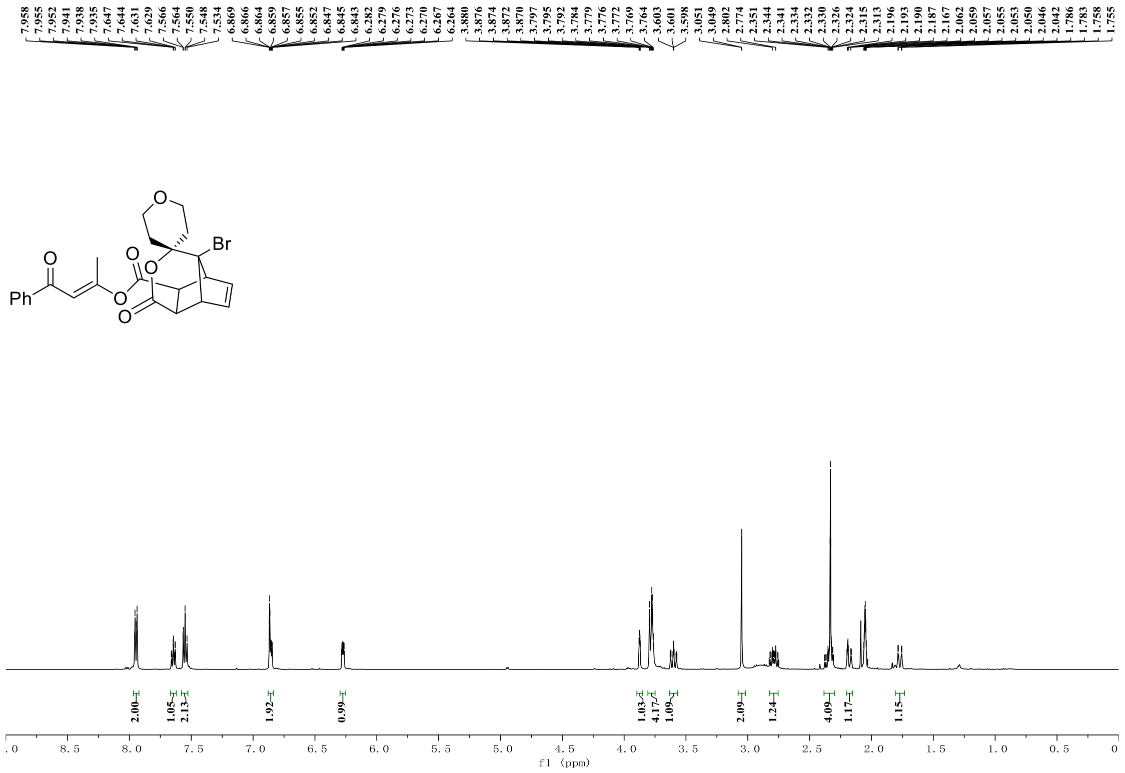

^{1}H NMR was recorded on Bruker 500 MHz; Solvent: Acetone-d_6

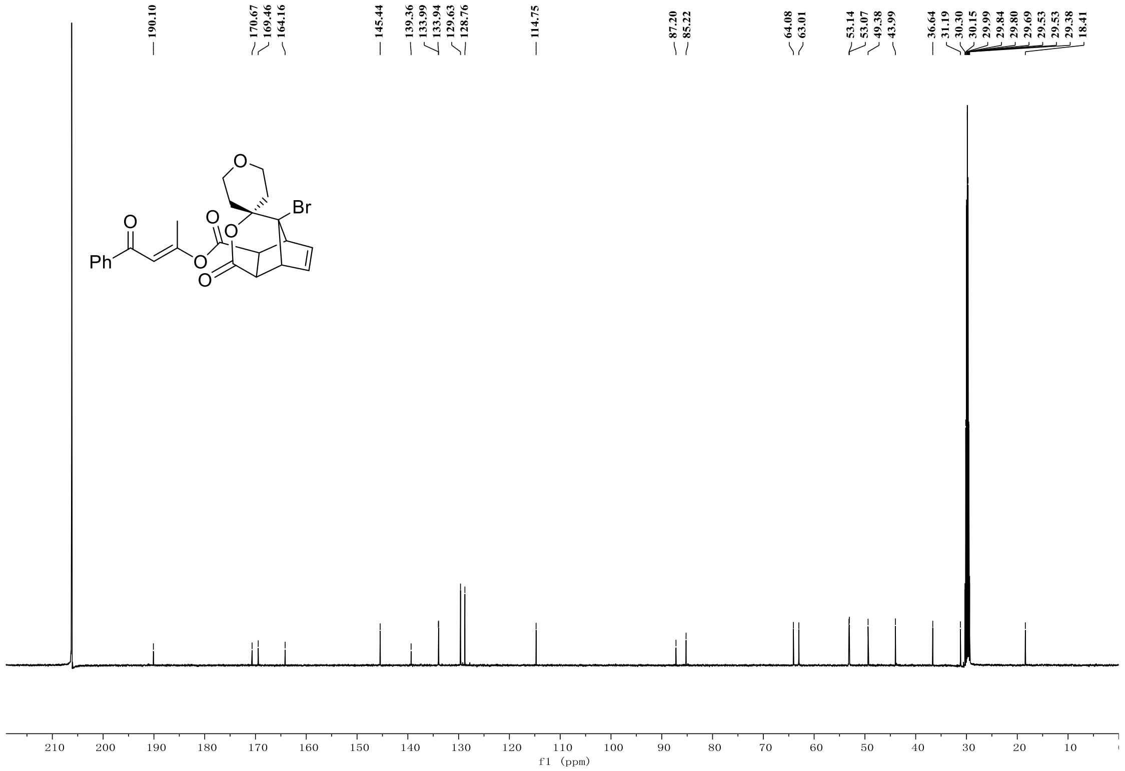

^{13}C NMR was recorded on Bruker 126 MHz; Solvent: Acetone-d_6

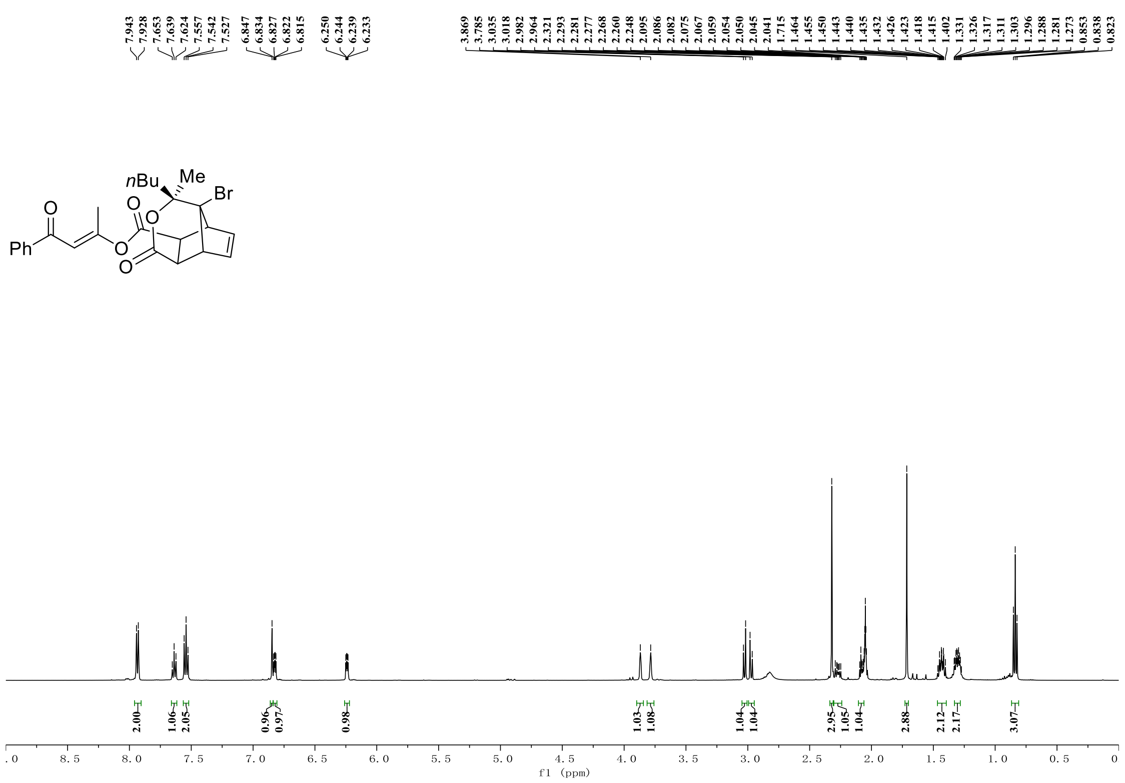

^{1}H NMR was recorded on Bruker 500 MHz; Solvent: Acetone-d_6

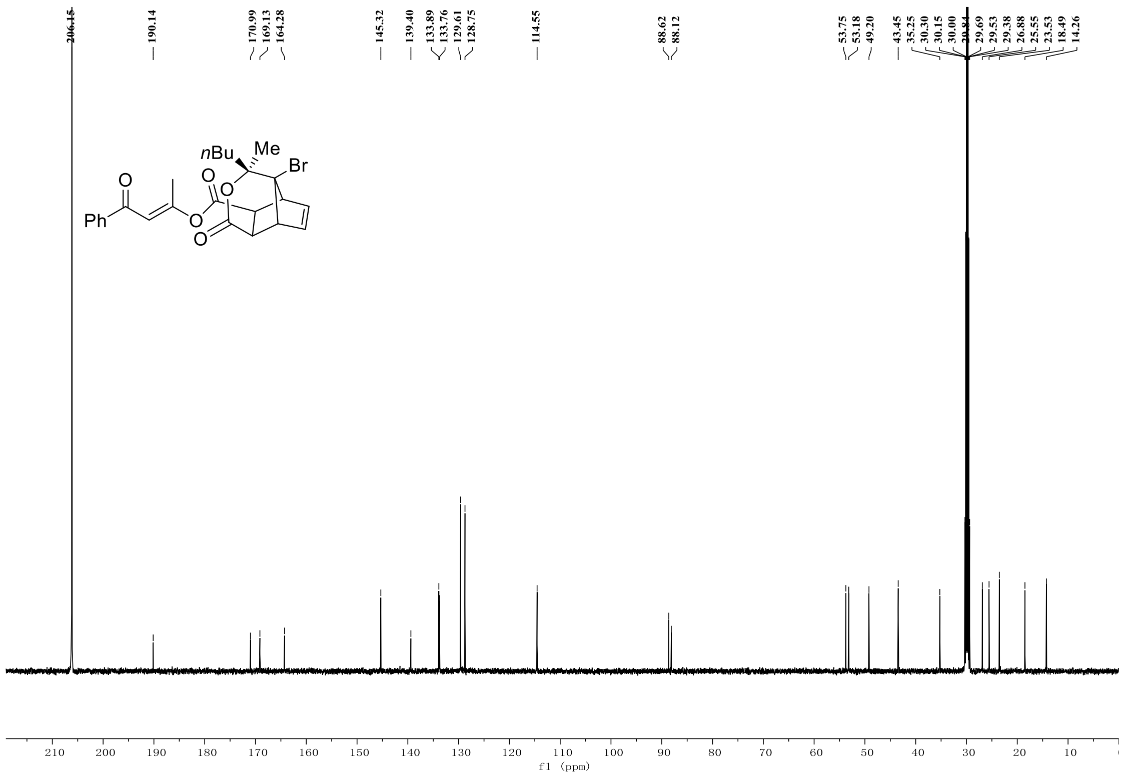

^{13}C NMR was recorded on Bruker 126 MHz; Solvent: Acetone-d_6

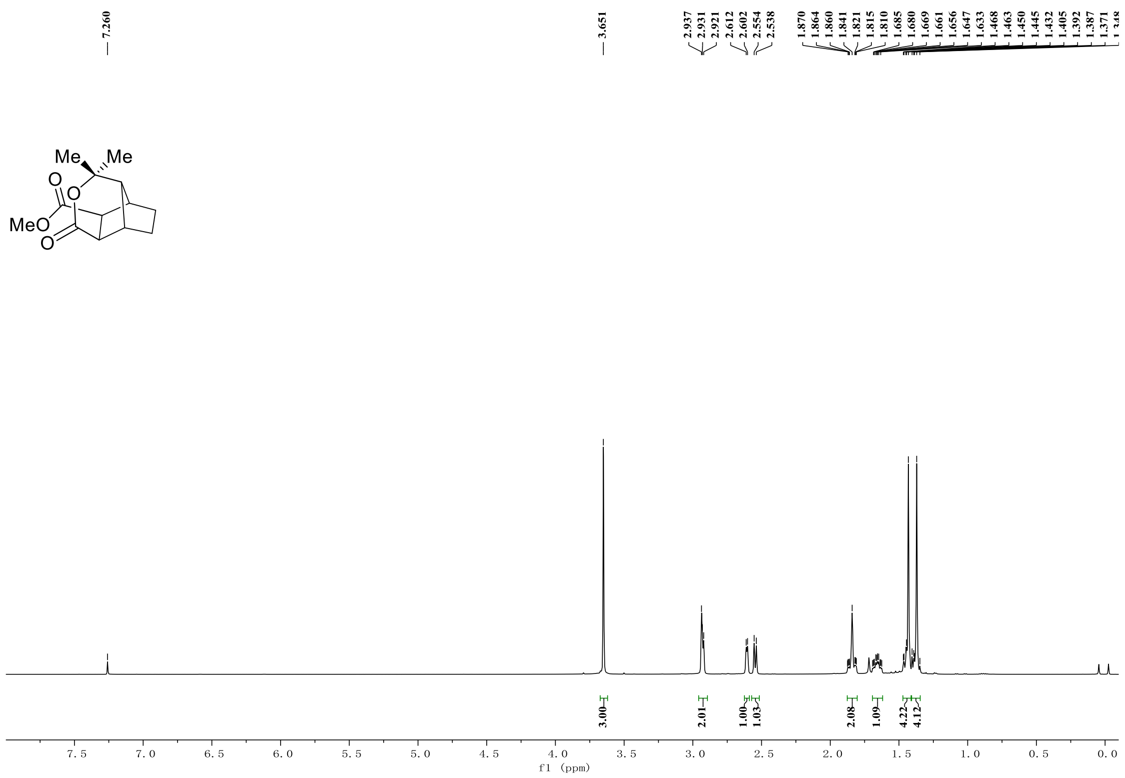

^{1}H NMR was recorded on Bruker 500 MHz; Solvent: $CDCl_3$

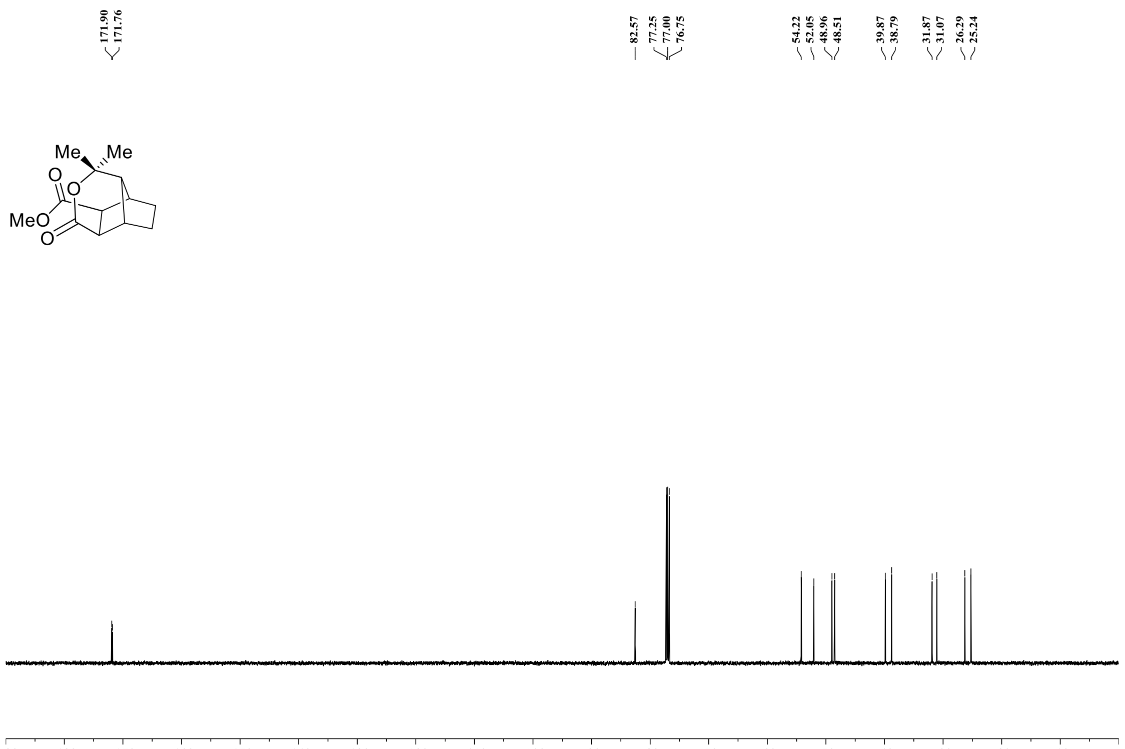

^{13}C NMR was recorded on Bruker 126 MHz; Solvent: $CDCl_3$

12. X-Ray Crystal Data

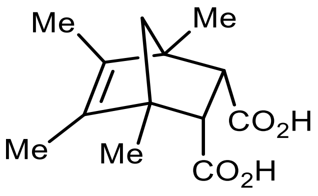

3a

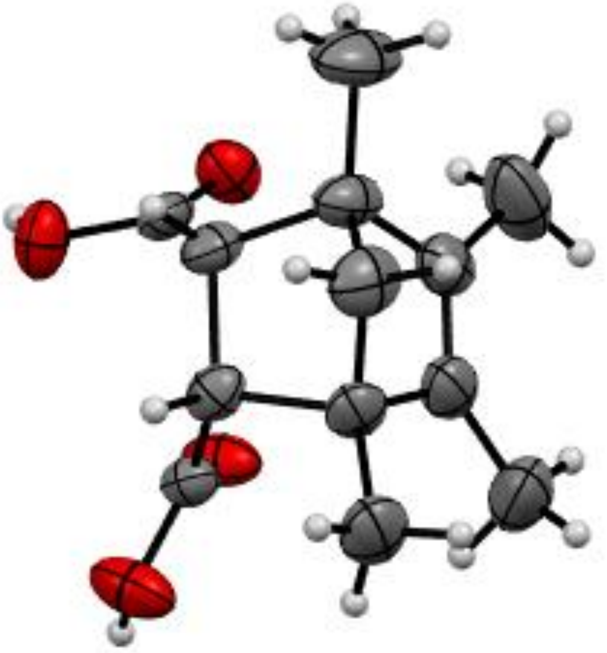

Fig S17. X-ray crystal structure of **3a** (CCDC: 2443944)

Table 1. Crystal data and structure refinement for **3a**.

Identification code	zqy4	
Empirical formula	C13 H18 O4	
Formula weight	238.27	
Temperature	293(2) K	
Wavelength	0.71073 Å	
Crystal system	Orthorhombic	
Space group	$P2_12_12_1$	
Unit cell dimensions	a = 7.1956(8) Å	α= 90°.
	b = 8.2736(10) Å	β= 90°.
	c = 21.672(2) Å	γ = 90°.
Volume	1290.2(2) $Å^3$	
Z	4	
Density (calculated)	1.227 Mg/m^3	
Absorption coefficient	0.090 mm^{-1}	
F(000)	512	
Crystal size	0.400 x 0.300 x 0.200 mm^3	
Theta range for data collection	2.635 to 28.050°.	
Index ranges	-9<=h<=9, -10<=k<=10, -28<=l<=28	
Reflections collected	31409	
Independent reflections	3109 [R(int) = 0.1258]	
Completeness to theta = 25.242°	99.8 %	
Absorption correction	multi-scan	
Max. and min. transmission	0.7456 and 0.5082	
Refinement method	Full-matrix least-squares on F^2	
Data / restraints / parameters	3109 / 0 / 159	
Goodness-of-fit on F^2	1.186	
Final R indices [I>2sigma(I)]	R1 = 0.0934, wR2 = 0.2143	
R indices (all data)	R1 = 0.1024, wR2 = 0.2188	
Absolute structure parameter	2(4)	
Extinction coefficient	n/a	
Largest diff. peak and hole	0.288 and -0.226 e.$Å^{-3}$	

Table 2. Atomic coordinates (x 10^4) and equivalent isotropic displacement parameters ($Å^2$ x 10^3) for **3a**. U(eq) is defined as one third of the trace of the orthogonalized U^{ij} tensor.

	x	y	z	U(eq)
O(1)	7614(6)	2274(6)	2848(2)	62(1)
O(2)	9605(5)	911(5)	2260(2)	49(1)
O(3)	7612(6)	-2296(5)	2347(2)	53(1)
O(4)	9261(6)	-2149(5)	1481(2)	49(1)
C(1)	8043(8)	1391(6)	2360(2)	38(1)
C(2)	6426(8)	1092(6)	1937(2)	38(1)
C(3)	6445(9)	-555(6)	1586(2)	41(1)
C(4)	7929(9)	-1714(6)	1782(3)	41(1)
C(5)	5706(13)	4040(7)	1580(3)	66(2)
C(6)	6364(9)	2364(6)	1385(3)	45(1)
C(7)	5071(11)	1401(8)	951(3)	59(2)
C(8)	6431(11)	-50(7)	893(3)	51(2)
C(9)	5872(14)	-1420(9)	464(3)	82(3)
C(10)	9676(15)	3533(8)	1090(4)	81(3)
C(11)	8179(11)	2259(7)	1038(3)	52(2)
C(12)	8258(10)	841(7)	749(2)	50(2)
C(13)	9764(15)	172(10)	358(3)	88(3)

Table 3. Bond lengths [Å] and angles [°] for **3a**.

O(1)-C(1)	1.321(6)
O(1)-H(1)	0.8200
O(2)-C(1)	1.212(7)
O(3)-C(4)	1.336(6)
O(3)-H(3)	0.9070
O(4)-C(4)	1.214(7)
C(1)-C(2)	1.502(8)
C(2)-C(3)	1.560(7)
C(2)-C(6)	1.594(7)
C(2)-H(2A)	0.9800
C(3)-C(4)	1.496(8)
C(3)-C(8)	1.560(8)
C(3)-H(3A)	0.9800
C(5)-C(6)	1.524(8)
C(5)-H(5A)	0.9600
C(5)-H(5B)	0.9600
C(5)-H(5C)	0.9600
C(6)-C(11)	1.510(9)
C(6)-C(7)	1.545(9)
C(7)-C(8)	1.553(9)
C(7)-H(7A)	0.9700
C(7)-H(7B)	0.9700
C(8)-C(9)	1.521(8)
C(8)-C(12)	1.539(10)
C(9)-H(9A)	0.9600
C(9)-H(9B)	0.9600
C(9)-H(9C)	0.9600
C(10)-C(11)	1.511(11)
C(10)-H(10A)	0.9600
C(10)-H(10B)	0.9600
C(10)-H(10C)	0.9600
C(11)-C(12)	1.331(8)
C(12)-C(13)	1.483(10)
C(13)-H(13A)	0.9600
C(13)-H(13B)	0.9600
C(13)-H(13C)	0.9600

C(1)-O(1)-H(1)	109.5
C(4)-O(3)-H(3)	108.9
O(2)-C(1)-O(1)	122.7(5)
O(2)-C(1)-C(2)	123.8(5)
O(1)-C(1)-C(2)	113.5(5)
C(1)-C(2)-C(3)	115.8(4)
C(1)-C(2)-C(6)	111.8(4)
C(3)-C(2)-C(6)	102.2(4)
C(1)-C(2)-H(2A)	108.9
C(3)-C(2)-H(2A)	108.9
C(6)-C(2)-H(2A)	108.9
C(4)-C(3)-C(8)	116.7(5)
C(4)-C(3)-C(2)	115.3(4)
C(8)-C(3)-C(2)	103.6(4)
C(4)-C(3)-H(3A)	106.9
C(8)-C(3)-H(3A)	106.9
C(2)-C(3)-H(3A)	106.9
O(4)-C(4)-O(3)	121.4(5)
O(4)-C(4)-C(3)	127.0(5)
O(3)-C(4)-C(3)	111.6(5)
C(6)-C(5)-H(5A)	109.5
C(6)-C(5)-H(5B)	109.5
H(5A)-C(5)-H(5B)	109.5
C(6)-C(5)-H(5C)	109.5
H(5A)-C(5)-H(5C)	109.5
H(5B)-C(5)-H(5C)	109.5
C(11)-C(6)-C(5)	117.3(6)
C(11)-C(6)-C(7)	100.8(5)
C(5)-C(6)-C(7)	116.8(6)
C(11)-C(6)-C(2)	108.2(4)
C(5)-C(6)-C(2)	113.7(5)
C(7)-C(6)-C(2)	97.7(4)
C(6)-C(7)-C(8)	93.9(5)
C(6)-C(7)-H(7A)	112.9
C(8)-C(7)-H(7A)	112.9
C(6)-C(7)-H(7B)	112.9
C(8)-C(7)-H(7B)	112.9

H(7A)-C(7)-H(7B)	110.4
C(9)-C(8)-C(12)	117.3(6)
C(9)-C(8)-C(7)	117.3(6)
C(12)-C(8)-C(7)	100.6(5)
C(9)-C(8)-C(3)	113.0(5)
C(12)-C(8)-C(3)	108.6(5)
C(7)-C(8)-C(3)	97.7(5)
C(8)-C(9)-H(9A)	109.5
C(8)-C(9)-H(9B)	109.5
H(9A)-C(9)-H(9B)	109.5
C(8)-C(9)-H(9C)	109.5
H(9A)-C(9)-H(9C)	109.5
H(9B)-C(9)-H(9C)	109.5
C(11)-C(10)-H(10A)	109.5
C(11)-C(10)-H(10B)	109.5
H(10A)-C(10)-H(10B)	109.5
C(11)-C(10)-H(10C)	109.5
H(10A)-C(10)-H(10C)	109.5
H(10B)-C(10)-H(10C)	109.5
C(12)-C(11)-C(6)	108.8(6)
C(12)-C(11)-C(10)	128.3(7)
C(6)-C(11)-C(10)	122.6(5)
C(11)-C(12)-C(13)	129.0(7)
C(11)-C(12)-C(8)	106.9(6)
C(13)-C(12)-C(8)	124.2(6)
C(12)-C(13)-H(13A)	109.5
C(12)-C(13)-H(13B)	109.5
H(13A)-C(13)-H(13B)	109.5
C(12)-C(13)-H(13C)	109.5
H(13A)-C(13)-H(13C)	109.5
H(13B)-C(13)-H(13C)	109.5

Symmetry transformations used to generate equivalent atoms:

Table 4. Anisotropic displacement parameters ($Å^2 \times 10^3$) for **3a**. The anisotropic displacement factor exponent takes the form: $-2\pi^2[h^2 a^{*2}U^{11} + ... + 2 h k a^* b^* U^{12}]$

	U^{11}	U^{22}	U^{33}	U^{23}	U^{13}	U^{12}
O(1)	48(3)	83(3)	54(2)	-31(2)	1(2)	2(2)
O(2)	35(2)	53(2)	58(2)	-21(2)	-6(2)	-1(2)
O(3)	53(3)	52(2)	53(2)	20(2)	5(2)	6(2)
O(4)	58(3)	45(2)	43(2)	1(2)	0(2)	15(2)
C(1)	44(3)	30(2)	41(3)	-4(2)	-1(2)	-2(2)
C(2)	35(3)	32(2)	48(3)	2(2)	1(2)	3(2)
C(3)	44(3)	29(2)	49(3)	-1(2)	-11(3)	-5(2)
C(4)	59(4)	22(2)	43(3)	2(2)	-7(3)	-7(2)
C(5)	97(6)	37(3)	64(4)	5(3)	11(4)	12(4)
C(6)	59(4)	27(2)	48(3)	1(2)	2(3)	10(2)
C(7)	68(5)	47(3)	61(4)	10(3)	-23(3)	8(3)
C(8)	76(5)	33(3)	45(3)	-1(2)	-22(3)	4(3)
C(9)	124(8)	55(4)	67(4)	-12(3)	-45(5)	-4(5)
C(10)	118(8)	48(4)	76(5)	5(3)	35(5)	-8(5)
C(11)	79(5)	35(3)	43(3)	6(2)	7(3)	8(3)
C(12)	76(5)	38(3)	36(3)	4(2)	-5(3)	12(3)
C(13)	140(9)	69(5)	55(4)	6(3)	33(5)	34(6)

Table 5. Hydrogen coordinates (x 10^4) and isotropic displacement parameters (Å^2 x 10^3) for **3a**.

	x	y	z	U(eq)
H(1)	8541	2399	3062	93
H(3)	8533	-2998	2446	79
H(2A)	5271	1175	2175	46
H(3A)	5250	-1080	1669	49
H(5A)	4465	3968	1741	99
H(5B)	5718	4748	1229	99
H(5C)	6520	4457	1892	99
H(7A)	4853	1944	560	70
H(7B)	3900	1110	1142	70
H(9A)	6834	-2223	458	123
H(9B)	5693	-1004	55	123
H(9C)	4736	-1897	608	123
H(10A)	9215	4541	933	121
H(10B)	10742	3206	855	121
H(10C)	10021	3661	1515	121
H(13A)	9253	-183	-28	132
H(13B)	10332	-726	565	132
H(13C)	10680	993	284	132

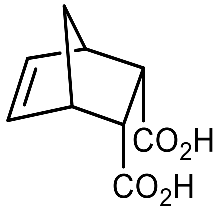

3c

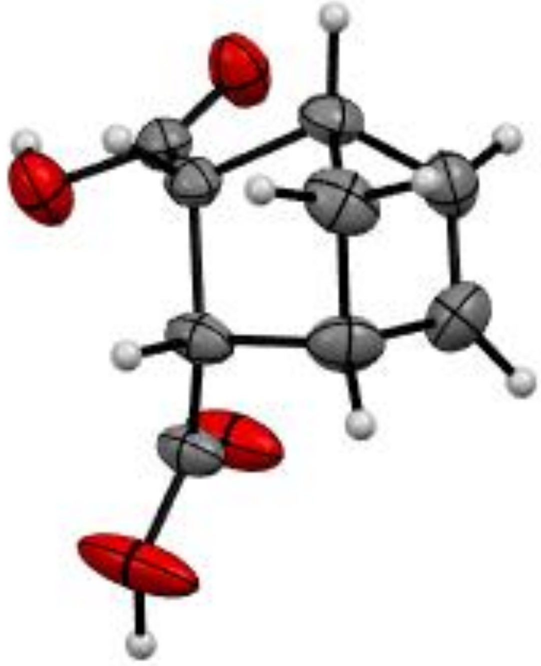

Fig S18. X-ray crystal structure of **3c** (CCDC: 2443943)

Table 1. Crystal data and structure refinement for **3c**.

Identification code	zqy3	
Empirical formula	C9 H10 O4	
Formula weight	182.17	
Temperature	296(2) K	
Wavelength	0.71073 Å	
Crystal system	Monoclinic	
Space group	$P2_1/c$	
Unit cell dimensions	a = 11.010(2) Å	α= 90°.
	b = 7.4001(13) Å	β= 98.369(6)°.
	c = 10.4884(17) Å	γ = 90°.
Volume	845.5(3) $Å^3$	
Z	4	
Density (calculated)	1.431 Mg/m^3	
Absorption coefficient	0.113 mm^{-1}	
F(000)	384	
Crystal size	0.500 x 0.400 x 0.300 mm^3	
Theta range for data collection	3.328 to 27.896°.	
Index ranges	-14<=h<=14, -9<=k<=9, -13<=l<=13	
Reflections collected	15832	
Independent reflections	2014 [R(int) = 0.0396]	
Completeness to theta = 25.242°	99.7 %	
Absorption correction	multi-scan	
Max. and min. transmission	0.7456 and 0.6269	
Refinement method	Full-matrix least-squares on F^2	
Data / restraints / parameters	2014 / 0 / 126	
Goodness-of-fit on F^2	1.036	
Final R indices [I>2sigma(I)]	R1 = 0.0493, wR2 = 0.1272	
R indices (all data)	R1 = 0.0545, wR2 = 0.1314	
Extinction coefficient	n/a	
Largest diff. peak and hole	0.383 and -0.425 e.$Å^{-3}$	

Table 2. Atomic coordinates (x 10^4) and equivalent isotropic displacement parameters ($Å^2$ x 10^3) for **3c**. U(eq) is defined as one third of the trace of the orthogonalized U^{ij} tensor.

	x	y	z	U(eq)
O(1)	9549(1)	7926(2)	4966(1)	44(1)
O(2)	8904(1)	10054(2)	6206(1)	47(1)
O(3)	6376(1)	8917(2)	4891(1)	56(1)
O(4)	5314(2)	8948(3)	6512(1)	80(1)
C(1)	7768(2)	4108(2)	7141(2)	45(1)
C(2)	8640(1)	5015(2)	6318(2)	35(1)
C(3)	7952(2)	4703(2)	4986(2)	40(1)
C(4)	6768(2)	4770(2)	5072(2)	46(1)
C(5)	6637(2)	5115(2)	6465(2)	42(1)
C(6)	8448(1)	7028(2)	6672(1)	30(1)
C(7)	8992(1)	8359(2)	5835(1)	31(1)
C(8)	7052(1)	7124(2)	6790(1)	34(1)
C(9)	6239(1)	8432(2)	5968(1)	36(1)

Table 3. Bond lengths [Å] and angles [°] for **3c**.

O(1)-C(7)	1.2141(18)
O(2)-C(7)	1.3205(18)
O(2)-H(2B)	0.93(3)
O(3)-C(9)	1.2154(18)
O(4)-C(9)	1.2956(18)
O(4)-H(4)	1.01(4)
C(1)-C(5)	1.533(2)
C(1)-C(2)	1.536(2)
C(1)-H(1A)	0.9700
C(1)-H(1B)	0.9700
C(2)-C(3)	1.507(2)
C(2)-C(6)	1.557(2)
C(2)-H(2A)	0.9800
C(3)-C(4)	1.321(2)
C(3)-H(3A)	0.9300
C(4)-C(5)	1.510(3)
C(4)-H(4A)	0.9300
C(5)-C(8)	1.577(2)
C(5)-H(5A)	0.9800
C(6)-C(7)	1.501(2)
C(6)-C(8)	1.5610(19)
C(6)-H(6A)	0.9800
C(8)-C(9)	1.5024(19)
C(8)-H(8A)	0.9800
C(7)-O(2)-H(2B)	106.4(16)
C(9)-O(4)-H(4)	113(2)
C(5)-C(1)-C(2)	93.43(12)
C(5)-C(1)-H(1A)	113.0
C(2)-C(1)-H(1A)	113.0
C(5)-C(1)-H(1B)	113.0
C(2)-C(1)-H(1B)	113.0
H(1A)-C(1)-H(1B)	110.4
C(3)-C(2)-C(1)	100.53(13)
C(3)-C(2)-C(6)	107.46(12)
C(1)-C(2)-C(6)	99.53(12)

C(3)-C(2)-H(2A)	115.7
C(1)-C(2)-H(2A)	115.7
C(6)-C(2)-H(2A)	115.7
C(4)-C(3)-C(2)	107.38(14)
C(4)-C(3)-H(3A)	126.3
C(2)-C(3)-H(3A)	126.3
C(3)-C(4)-C(5)	107.85(15)
C(3)-C(4)-H(4A)	126.1
C(5)-C(4)-H(4A)	126.1
C(4)-C(5)-C(1)	100.38(14)
C(4)-C(5)-C(8)	107.63(13)
C(1)-C(5)-C(8)	99.68(13)
C(4)-C(5)-H(5A)	115.7
C(1)-C(5)-H(5A)	115.7
C(8)-C(5)-H(5A)	115.7
C(7)-C(6)-C(2)	114.08(11)
C(7)-C(6)-C(8)	119.86(11)
C(2)-C(6)-C(8)	103.43(12)
C(7)-C(6)-H(6A)	106.2
C(2)-C(6)-H(6A)	106.2
C(8)-C(6)-H(6A)	106.2
O(1)-C(7)-O(2)	122.55(14)
O(1)-C(7)-C(6)	123.68(13)
O(2)-C(7)-C(6)	113.51(12)
C(9)-C(8)-C(6)	119.64(12)
C(9)-C(8)-C(5)	110.74(13)
C(6)-C(8)-C(5)	101.39(11)
C(9)-C(8)-H(8A)	108.2
C(6)-C(8)-H(8A)	108.2
C(5)-C(8)-H(8A)	108.2
O(3)-C(9)-O(4)	122.32(14)
O(3)-C(9)-C(8)	125.43(13)
O(4)-C(9)-C(8)	112.17(13)

Symmetry transformations used to generate equivalent atoms:

Table 4. Anisotropic displacement parameters ($Å^2$ x 10^3) for **3c**. The anisotropic displacement factor exponent takes the form: $-2\pi^2[h^2 a^{*2}U^{11} + ... + 2 h k a^* b^* U^{12}]$

	U^{11}	U^{22}	U^{33}	U^{23}	U^{13}	U^{12}
O(1)	51(1)	39(1)	48(1)	-7(1)	23(1)	-5(1)
O(2)	58(1)	33(1)	54(1)	-6(1)	22(1)	1(1)
O(3)	51(1)	81(1)	41(1)	23(1)	20(1)	36(1)
O(4)	70(1)	124(2)	51(1)	33(1)	33(1)	67(1)
C(1)	50(1)	39(1)	48(1)	12(1)	13(1)	8(1)
C(2)	31(1)	33(1)	42(1)	3(1)	6(1)	9(1)
C(3)	45(1)	36(1)	40(1)	-7(1)	10(1)	-2(1)
C(4)	41(1)	47(1)	47(1)	-4(1)	0(1)	-9(1)
C(5)	32(1)	47(1)	49(1)	10(1)	13(1)	0(1)
C(6)	28(1)	34(1)	27(1)	0(1)	1(1)	6(1)
C(7)	27(1)	33(1)	32(1)	-3(1)	1(1)	3(1)
C(8)	32(1)	43(1)	28(1)	6(1)	9(1)	11(1)
C(9)	32(1)	45(1)	32(1)	4(1)	10(1)	12(1)

Table 5. Hydrogen coordinates (x 10^4) and isotropic displacement parameters ($Å^2$ x 10^3) for **3c**.

	x	y	z	U(eq)
H(2B)	9400(20)	10730(40)	5740(20)	74(7)
H(4)	4720(40)	9730(50)	5930(40)	139(14)
H(1A)	7714	2810	7021	54
H(1B)	7959	4401	8050	54
H(2A)	9492	4586	6462	43
H(3A)	8297	4500	4240	48
H(4A)	6128	4630	4396	55
H(5A)	5851	4760	6722	51
H(6A)	8887	7196	7546	36
H(8A)	6972	7358	7694	41

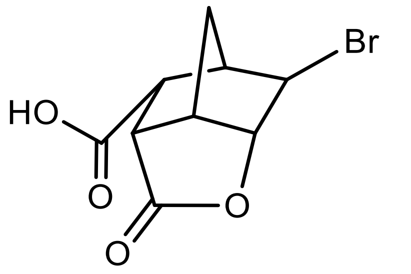

6c

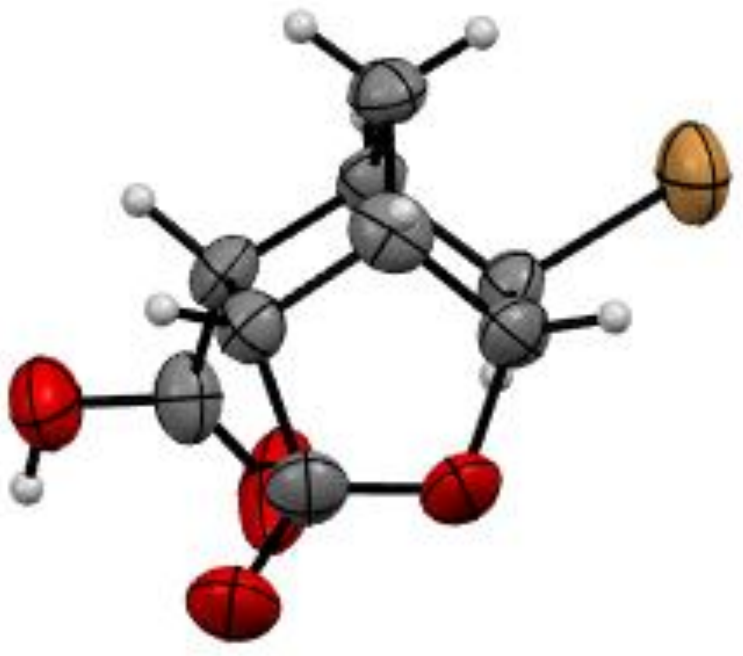

Fig S19. X-ray crystal structure of **6c** (CCDC: 2416060)

Table 1. Crystal data and structure refinement for **6c**.

Identification code	ZQY2	
Empirical formula	C9 H9 Br O4	
Formula weight	261.07	
Temperature	273(2) K	
Wavelength	0.71073 Å	
Crystal system	Orthorhombic	
Space group	$P2_12_12_1$	
Unit cell dimensions	a = 6.1862(5) Å	α= 90°.
	b = 8.1872(8) Å	β= 90°.
	c = 18.4834(17) Å	γ = 90°.
Volume	936.14(15) $Å^3$	
Z	4	
Density (calculated)	1.852 Mg/m^3	
Absorption coefficient	4.375 mm^{-1}	
F(000)	520	
Crystal size	0.500 x 0.400 x 0.200 mm^3	
Theta range for data collection	2.721 to 27.921°.	
Index ranges	-8<=h<=7, -10<=k<=10, -24<=l<=24	
Reflections collected	10978	
Independent reflections	2230 [R(int) = 0.0492]	
Completeness to theta = 25.242°	99.3 %	
Absorption correction	multi-scan	
Max. and min. transmission	0.7456 and 0.4135	
Refinement method	Full-matrix least-squares on F^2	
Data / restraints / parameters	2230 / 0 / 132	
Goodness-of-fit on F^2	1.097	
Final R indices [I>2sigma(I)]	R1 = 0.0340, wR2 = 0.0873	
R indices (all data)	R1 = 0.0391, wR2 = 0.0889	
Absolute structure parameter	0.044(5)	
Extinction coefficient	0.037(5)	
Largest diff. peak and hole	0.708 and -0.624 e.$Å^{-3}$	

Table 2. Atomic coordinates (x 10^4) and equivalent isotropic displacement parameters (Å^2 x 10^3) for **6c**. U(eq) is defined as one third of the trace of the orthogonalized U^{ij} tensor.

	x	y	z	U(eq)
Br(1)	7643(1)	4050(1)	5280(1)	67(1)
O(1)	7852(6)	4173(4)	3082(2)	61(1)
O(2)	7959(7)	5899(5)	2143(2)	72(1)
O(3)	9856(5)	7662(6)	3512(3)	107(2)
C(1)	7530(7)	5118(4)	4338(2)	41(1)
C(2)	6580(7)	4028(5)	3739(2)	47(1)
C(3)	5927(6)	6545(5)	4366(2)	39(1)
C(4)	3783(6)	5651(6)	4278(2)	47(1)
C(5)	4410(7)	4825(5)	3574(2)	46(1)
C(6)	6005(6)	7521(5)	3651(2)	41(1)
C(7)	5232(6)	6214(5)	3083(2)	40(1)
C(8)	7124(8)	5468(5)	2699(2)	52(1)
C(9)	8151(7)	8330(5)	3477(2)	50(1)
O(4)	7981(7)	9815(4)	3241(2)	71(1)

Table 3. Bond lengths [Å] and angles [°] for **6c**.

Br(1)-C(1)	1.951(4)
O(1)-C(8)	1.352(5)
O(1)-C(2)	1.453(5)
O(2)-C(8)	1.204(5)
O(3)-C(9)	1.190(6)
C(1)-C(3)	1.533(5)
C(1)-C(2)	1.539(5)
C(1)-H(1A)	0.9800
C(2)-C(5)	1.523(6)
C(2)-H(2A)	0.9800
C(3)-C(4)	1.524(6)
C(3)-C(6)	1.546(5)
C(3)-H(3A)	0.9800
C(4)-C(5)	1.516(6)
C(4)-H(4A)	0.9700
C(4)-H(4B)	0.9700
C(5)-C(7)	1.541(6)
C(5)-H(5A)	0.9800
C(6)-C(9)	1.517(6)
C(6)-C(7)	1.573(5)
C(6)-H(6A)	0.9800
C(7)-C(8)	1.499(6)
C(7)-H(7A)	0.9800
C(9)-O(4)	1.297(5)
O(4)-H(4C)	0.89(7)
C(8)-O(1)-C(2)	108.7(3)
C(3)-C(1)-C(2)	102.7(3)
C(3)-C(1)-Br(1)	109.5(2)
C(2)-C(1)-Br(1)	113.3(3)
C(3)-C(1)-H(1A)	110.4
C(2)-C(1)-H(1A)	110.4
Br(1)-C(1)-H(1A)	110.4
O(1)-C(2)-C(5)	106.0(3)
O(1)-C(2)-C(1)	110.3(3)
C(5)-C(2)-C(1)	103.4(3)

O(1)-C(2)-H(2A)	112.2
C(5)-C(2)-H(2A)	112.2
C(1)-C(2)-H(2A)	112.2
C(4)-C(3)-C(1)	101.2(3)
C(4)-C(3)-C(6)	100.6(3)
C(1)-C(3)-C(6)	110.1(3)
C(4)-C(3)-H(3A)	114.5
C(1)-C(3)-H(3A)	114.5
C(6)-C(3)-H(3A)	114.5
C(5)-C(4)-C(3)	94.8(3)
C(5)-C(4)-H(4A)	112.8
C(3)-C(4)-H(4A)	112.8
C(5)-C(4)-H(4B)	112.8
C(3)-C(4)-H(4B)	112.8
H(4A)-C(4)-H(4B)	110.2
C(4)-C(5)-C(2)	104.2(3)
C(4)-C(5)-C(7)	105.1(3)
C(2)-C(5)-C(7)	98.2(3)
C(4)-C(5)-H(5A)	115.7
C(2)-C(5)-H(5A)	115.7
C(7)-C(5)-H(5A)	115.7
C(9)-C(6)-C(3)	115.7(3)
C(9)-C(6)-C(7)	115.0(3)
C(3)-C(6)-C(7)	102.1(3)
C(9)-C(6)-H(6A)	107.9
C(3)-C(6)-H(6A)	107.9
C(7)-C(6)-H(6A)	107.9
C(8)-C(7)-C(5)	103.6(3)
C(8)-C(7)-C(6)	110.8(3)
C(5)-C(7)-C(6)	102.1(3)
C(8)-C(7)-H(7A)	113.1
C(5)-C(7)-H(7A)	113.1
C(6)-C(7)-H(7A)	113.1
O(2)-C(8)-O(1)	122.2(4)
O(2)-C(8)-C(7)	128.3(4)
O(1)-C(8)-C(7)	109.4(4)
O(3)-C(9)-O(4)	121.4(5)
O(3)-C(9)-C(6)	124.3(4)

O(4)-C(9)-C(6)	114.2(4)
C(9)-O(4)-H(4C)	111(4)

Symmetry transformations used to generate equivalent atoms:

Table 4. Anisotropic displacement parameters ($Å^2 \times 10^3$) for **6c**. The anisotropic displacement factor exponent takes the form: $-2\pi^2[h^2 a^{*2}U^{11} + ... + 2 h k a^* b^* U^{12}]$

	U^{11}	U^{22}	U^{33}	U^{23}	U^{13}	U^{12}
Br(1)	79(1)	67(1)	55(1)	16(1)	-17(1)	8(1)
O(1)	79(2)	50(2)	55(2)	-4(1)	20(2)	23(2)
O(2)	90(3)	79(2)	49(2)	0(2)	22(2)	17(2)
O(3)	41(2)	82(3)	198(5)	68(4)	-10(3)	-3(2)
C(1)	36(2)	42(2)	44(2)	4(1)	-3(2)	5(2)
C(2)	61(2)	33(2)	48(2)	-6(2)	11(2)	4(2)
C(3)	44(2)	38(2)	34(2)	-6(1)	-2(2)	7(2)
C(4)	35(2)	58(2)	48(2)	2(2)	5(2)	4(2)
C(5)	45(2)	50(2)	44(2)	-6(2)	-6(2)	-13(2)
C(6)	37(2)	38(2)	47(2)	-4(2)	-4(2)	10(2)
C(7)	40(2)	44(2)	36(2)	-3(2)	-6(1)	1(2)
C(8)	64(3)	51(2)	40(2)	-9(2)	5(2)	6(2)
C(9)	56(2)	42(2)	50(2)	5(2)	-13(2)	-3(2)
O(4)	75(3)	51(2)	86(2)	23(2)	9(2)	3(2)

Table 5. Hydrogen coordinates (x 10^4) and isotropic displacement parameters (Å^2 x 10^3) for **6c**.

	x	y	z	U(eq)
H(1A)	8969	5509	4199	49
H(2A)	6421	2890	3895	57
H(3A)	6029	7216	4804	47
H(4A)	3506	4879	4665	56
H(4B)	2567	6389	4223	56
H(5A)	3312	4108	3361	56
H(6A)	4902	8378	3676	49
H(7A)	4123	6630	2752	48
H(4C)	9250(110)	10180(70)	3080(40)	74(19)

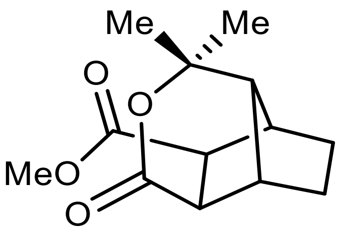

14

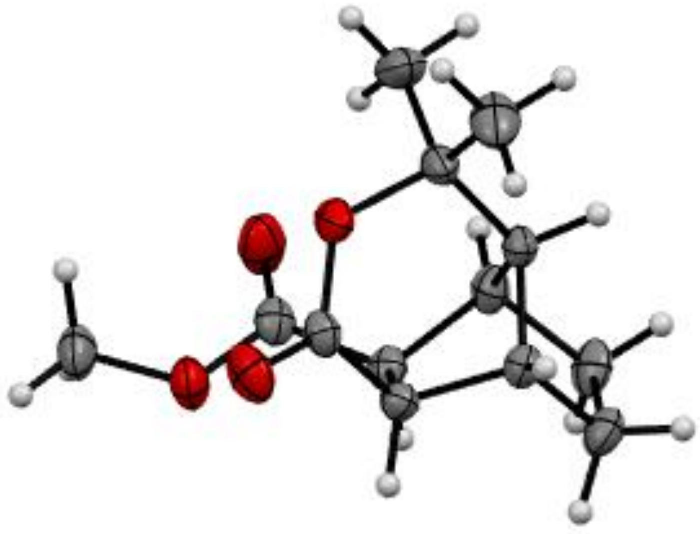

Fig S20. X-ray crystal structure of **14** (CCDC: 2411170)

Table 1. Crystal data and structure refinement for **14**.

Identification code	zqy1(low)	
Empirical formula	C13 H18 O4	
Formula weight	238.27	
Temperature	196(2) K	
Wavelength	0.71073 Å	
Crystal system	Orthorhombic	
Space group	Pbca	
Unit cell dimensions	a = 9.3103(10) Å	α= 90°.
	b = 12.2251(16) Å	β= 90°.
	c = 21.345(3) Å	γ = 90°.
Volume	2429.5(5) Å^3	
Z	8	
Density (calculated)	1.303 Mg/m^3	
Absorption coefficient	0.096 mm^{-1}	
F(000)	1024	
Crystal size	0.400 x 0.300 x 0.200 mm^3	
Theta range for data collection	3.333 to 28.293°.	
Index ranges	-12<=h<=12, -16<=k<=16, -28<=l<=28	
Reflections collected	52312	
Independent reflections	3023 [R(int) = 0.0509]	
Completeness to theta = 25.242°	99.9 %	
Absorption correction	multi-scan	
Max. and min. transmission	0.7457 and 0.6898	
Refinement method	Full-matrix least-squares on F^2	
Data / restraints / parameters	3023 / 0 / 157	
Goodness-of-fit on F^2	1.040	
Final R indices [I>2sigma(I)]	R1 = 0.0411, wR2 = 0.1239	
R indices (all data)	R1 = 0.0474, wR2 = 0.1296	
Extinction coefficient	n/a	
Largest diff. peak and hole	0.329 and -0.279 e.Å^{-3}	

Table 2. Atomic coordinates (x 10^4) and equivalent isotropic displacement parameters (Å^2 x 10^3) for **14**. U(eq) is defined as one third of the trace of the orthogonalized U^{ij} tensor.

	x	y	z	U(eq)
O(1)	5551(1)	3369(1)	4163(1)	35(1)
O(2)	5972(1)	3170(1)	3140(1)	47(1)
O(3)	8507(1)	5029(1)	3923(1)	33(1)
O(4)	7711(1)	4925(1)	4887(1)	40(1)
C(1)	10854(2)	5424(1)	3542(1)	46(1)
C(2)	9561(1)	4671(1)	3448(1)	30(1)
C(3)	8840(2)	4883(1)	2825(1)	44(1)
C(4)	10013(1)	3477(1)	3534(1)	24(1)
C(5)	10175(1)	3131(1)	4224(1)	25(1)
C(6)	8591(1)	3256(1)	4427(1)	24(1)
C(7)	8236(1)	4457(1)	4446(1)	26(1)
C(8)	10550(1)	1906(1)	4178(1)	33(1)
C(9)	9867(1)	1554(1)	3547(1)	35(1)
C(10)	8954(1)	2553(1)	3371(1)	27(1)
C(11)	7793(1)	2647(1)	3886(1)	24(1)
C(12)	6373(1)	3108(1)	3673(1)	28(1)
C(13)	4119(1)	3751(1)	4021(1)	38(1)

Table 3. Bond lengths [Å] and angles [°] for **14**.

O(1)-C(12)	1.3343(14)
O(1)-C(13)	1.4445(14)
O(2)-C(12)	1.1993(15)
O(3)-C(7)	1.3406(14)
O(3)-C(2)	1.4779(13)
O(4)-C(7)	1.2061(13)
C(1)-C(2)	1.5289(18)
C(1)-H(1A)	0.9800
C(1)-H(1B)	0.9800
C(1)-H(1C)	0.9800
C(2)-C(3)	1.5117(18)
C(2)-C(4)	1.5301(15)
C(3)-H(3A)	0.9800
C(3)-H(3B)	0.9800
C(3)-H(3C)	0.9800
C(4)-C(5)	1.5388(14)
C(4)-C(10)	1.5393(15)
C(4)-H(4)	1.0000
C(5)-C(8)	1.5411(15)
C(5)-C(6)	1.5449(15)
C(5)-H(5)	1.0000
C(6)-C(7)	1.5048(15)
C(6)-C(11)	1.5619(14)
C(6)-H(6)	1.0000
C(8)-C(9)	1.5503(17)
C(8)-H(8A)	0.9900
C(8)-H(8B)	0.9900
C(9)-C(10)	1.5354(15)
C(9)-H(9A)	0.9900
C(9)-H(9B)	0.9900
C(10)-C(11)	1.5451(15)
C(10)-H(10)	1.0000
C(11)-C(12)	1.5076(15)
C(11)-H(11)	1.0000
C(13)-H(13A)	0.9800
C(13)-H(13B)	0.9800

C(13)-H(13C)	0.9800
C(12)-O(1)-C(13)	116.26(10)
C(7)-O(3)-C(2)	122.74(8)
C(2)-C(1)-H(1A)	109.5
C(2)-C(1)-H(1B)	109.5
H(1A)-C(1)-H(1B)	109.5
C(2)-C(1)-H(1C)	109.5
H(1A)-C(1)-H(1C)	109.5
H(1B)-C(1)-H(1C)	109.5
O(3)-C(2)-C(3)	104.95(10)
O(3)-C(2)-C(1)	104.74(10)
C(3)-C(2)-C(1)	111.18(11)
O(3)-C(2)-C(4)	112.56(8)
C(3)-C(2)-C(4)	113.02(10)
C(1)-C(2)-C(4)	110.00(10)
C(2)-C(3)-H(3A)	109.5
C(2)-C(3)-H(3B)	109.5
H(3A)-C(3)-H(3B)	109.5
C(2)-C(3)-H(3C)	109.5
H(3A)-C(3)-H(3C)	109.5
H(3B)-C(3)-H(3C)	109.5
C(2)-C(4)-C(5)	113.80(9)
C(2)-C(4)-C(10)	119.74(9)
C(5)-C(4)-C(10)	94.44(8)
C(2)-C(4)-H(4)	109.3
C(5)-C(4)-H(4)	109.3
C(10)-C(4)-H(4)	109.3
C(4)-C(5)-C(8)	103.21(8)
C(4)-C(5)-C(6)	98.50(8)
C(8)-C(5)-C(6)	109.32(9)
C(4)-C(5)-H(5)	114.7
C(8)-C(5)-H(5)	114.7
C(6)-C(5)-H(5)	114.7
C(7)-C(6)-C(5)	108.30(9)
C(7)-C(6)-C(11)	112.37(8)
C(5)-C(6)-C(11)	101.48(8)
C(7)-C(6)-H(6)	111.4
C(5)-C(6)-H(6)	111.4

C(11)-C(6)-H(6)	111.4
O(4)-C(7)-O(3)	118.55(10)
O(4)-C(7)-C(6)	124.99(11)
O(3)-C(7)-C(6)	116.45(9)
C(5)-C(8)-C(9)	103.41(9)
C(5)-C(8)-H(8A)	111.1
C(9)-C(8)-H(8A)	111.1
C(5)-C(8)-H(8B)	111.1
C(9)-C(8)-H(8B)	111.1
H(8A)-C(8)-H(8B)	109.0
C(10)-C(9)-C(8)	102.64(9)
C(10)-C(9)-H(9A)	111.2
C(8)-C(9)-H(9A)	111.2
C(10)-C(9)-H(9B)	111.2
C(8)-C(9)-H(9B)	111.2
H(9A)-C(9)-H(9B)	109.2
C(9)-C(10)-C(4)	99.98(9)
C(9)-C(10)-C(11)	105.82(9)
C(4)-C(10)-C(11)	103.47(8)
C(9)-C(10)-H(10)	115.3
C(4)-C(10)-H(10)	115.3
C(11)-C(10)-H(10)	115.3
C(12)-C(11)-C(10)	115.27(9)
C(12)-C(11)-C(6)	117.50(9)
C(10)-C(11)-C(6)	103.23(8)
C(12)-C(11)-H(11)	106.7
C(10)-C(11)-H(11)	106.7
C(6)-C(11)-H(11)	106.7
O(2)-C(12)-O(1)	123.33(11)
O(2)-C(12)-C(11)	125.62(10)
O(1)-C(12)-C(11)	110.88(9)
O(1)-C(13)-H(13A)	109.5
O(1)-C(13)-H(13B)	109.5
H(13A)-C(13)-H(13B)	109.5
O(1)-C(13)-H(13C)	109.5
H(13A)-C(13)-H(13C)	109.5
H(13B)-C(13)-H(13C)	109.5

Symmetry transformations used to generate equivalent atoms:

Table 4. Anisotropic displacement parameters ($Å^2 x 10^3$) for **14**. The anisotropic displacement factor exponent takes the form: $-2\pi^2[h^2 a^{*2}U^{11} + ... + 2 h k a^* b^* U^{12}]$

	U^{11}	U^{22}	U^{33}	U^{23}	U^{13}	U^{12}
O(1)	24(1)	43(1)	39(1)	-3(1)	4(1)	6(1)
O(2)	34(1)	70(1)	37(1)	-5(1)	-6(1)	12(1)
O(3)	38(1)	22(1)	38(1)	2(1)	13(1)	10(1)
O(4)	43(1)	38(1)	39(1)	-16(1)	12(1)	1(1)
C(1)	39(1)	27(1)	73(1)	6(1)	13(1)	-3(1)
C(2)	30(1)	25(1)	34(1)	4(1)	12(1)	5(1)
C(3)	53(1)	45(1)	34(1)	15(1)	7(1)	15(1)
C(4)	23(1)	23(1)	26(1)	-1(1)	5(1)	4(1)
C(5)	23(1)	24(1)	28(1)	-1(1)	-1(1)	3(1)
C(6)	26(1)	23(1)	22(1)	1(1)	2(1)	1(1)
C(7)	24(1)	26(1)	28(1)	-6(1)	3(1)	1(1)
C(8)	33(1)	26(1)	41(1)	2(1)	-2(1)	9(1)
C(9)	34(1)	24(1)	48(1)	-10(1)	1(1)	8(1)
C(10)	28(1)	26(1)	28(1)	-8(1)	-1(1)	6(1)
C(11)	23(1)	20(1)	30(1)	-3(1)	0(1)	0(1)
C(12)	24(1)	24(1)	36(1)	-3(1)	0(1)	-1(1)
C(13)	23(1)	37(1)	55(1)	0(1)	6(1)	4(1)

Table 5. Hydrogen coordinates (x 10^4) and isotropic displacement parameters ($Å^2$ x 10^3) for **14**.

	x	y	z	U(eq)
H(1A)	11584	5261	3225	69
H(1B)	11259	5306	3961	69
H(1C)	10548	6188	3501	69
H(3A)	9496	4684	2485	66
H(3B)	8594	5660	2793	66
H(3C)	7964	4442	2795	66
H(4)	10941	3348	3310	29
H(5)	10873	3578	4471	30
H(6)	8410	2899	4841	29
H(8A)	10132	1491	4532	40
H(8B)	11604	1794	4173	40
H(9A)	10612	1409	3227	42
H(9B)	9264	893	3599	42
H(10)	8584	2560	2931	33
H(11)	7601	1888	4038	29
H(13A)	4172	4348	3715	58
H(13B)	3661	4016	4405	58
H(13C)	3553	3148	3846	58
